# Supplementary material for: Structural characterization of human RPA70N association with DNA damage response proteins
Source: eLife. 2023 Sep 5;12:e81639. doi: 10.7554/eLife.81639 (PMC10479964; doi:10.7554/eLife.81639)
Supplement: Figure 1—source data 1. [file elife-81639-fig1-data1.docx]

**Table S1.**

**Data Collection and Refinement Statistics part 1**

| Data Set | RPA70N-HelB | RPA70N-BLMp2 | RPA70N-BLMp1 |
| --- | --- | --- | --- |
| **Data collection** |  |  |  |
| PDB code | 7XUT | 7XUV | 7XV0 |
| Space group | *P 41 21 2* | *P 41 21 2* | *P 21 21 21* |
| *a*, *b*, *c* (Å) | 50.333 50.333 126.481 | 62.4761 62.4761 69.497 | 38.088 53.644 54.501 |
| *α*, *β*, *γ* (°) | 90, 90, 90 | 90, 90, 90 | 90, 90, 90 |
| Resolution (Å) | 22.6 - 1.8 (1.864 - 1.8) | 25.92 - 1.8 (1.864 - 1.8) | 31.22 - 1.5 (1.554 - 1.5) |
| Observed reflections | 193641 (19790) | 162807 (16556) | 111286 (10924) |
| Unique reflections | 15793 (1542) | 13283 (1288) | 18207 (1767) |
| R_merge_ (%) | 6.153 (48.31) | 13.12 (76.71) | 3.1 (8.807) |
| R_pim_ (%) | 1.881 (14) | 3.932 (22.15) | 1.362 (3.812) |
| I/σ(I) | 26.49 (8.87) | 13.60 (5.83) | 34.45 (16.08) |
| CC_1/2_ | 0.998 (0.982) | 0.996 (0.954) | 0.999 (0.996) |
| Completeness (%) | 99.83 (100.00) | 99.92 (100.00) | 98.58 (98.44) |
| Multiplicity | 12.3 (12.8) | 12.3 (12.9) | 6.1 (6.2) |
| **Refinement** |  |  |  |
| R_work_/R_free_ (%) | 19.99/22.09 | 19.31/21.70 | 16.88/20.03 |
| No. protein atoms | 1130 | 1059 | 1050 |
| No. ligand atoms | 0 | 0 | 0 |
| No. solvent atoms | 94 | 96 | 132 |
| Average B-factor (Å^2^) | 35.38 | 26.81 | 18.73 |
| Protein B-factor (Å^2^) | 34.89 | 26.32 | 17.54 |
| Solvent B-factor (Å^2^) | 41.25 | 32.17 | 28.19 |
| Rmsd bond lengths (Å) | 0.008 | 0.007 | 0.008 |
| Rmsd bond angles (°) | 1.15 | 0.92 | 0.92 |
| Ramachandran outliers (%) | 0.00 | 0.00 | 0.00 |
| Ramachandran favored (%) | 97.86 | 97.79 | 97.69 |

Values in parentheses are for the highest-resolution shell.

**Data Collection and Refinement Statistics part 2**

| Data Set | RPA70N-RMI1 | RPA70N-WRN | RPA70N-ATRIP |
| --- | --- | --- | --- |
| **Data collection** |  |  |  |
| PDB code | 7XV1 | 7XV4 | 7XUW |
| Space group | *P 21 21 21* | *P 21 21 21* | *P 21 21 21* |
| *a*, *b*, *c* (Å) | 40.93 50.263 52.308 | 32.936 58.609 111.786 | 39.035 53.11 55.224 |
| *α*, *β*, *γ* (°) | 90, 90, 90 | 90, 90, 90 | 90, 90, 90 |
| Resolution (Å) | 27.13 - 1.6 (1.657 - 1.6) | 31.44 - 1.6 (1.657 - 1.6) | 38.28 - 1.6 (1.657 - 1.6) |
| Observed reflections | 60739 (6230) | 181925 (18864) | 197641 (20518) |
| Unique reflections | 13228 (1355) | 29352 (2870) | 15720 (1555) |
| R_merge_ (%) | 6.908 (34.22) | 9.657 (15.2) | 3.727 (14.91) |
| R_pim_ (%) | 3.173 (15.78) | 4.187 (6.41) | 1.094 (4.257) |
| I/σ(I) | 13.80 (4.84) | 13.82 (8.55) | 43.83 (16.69) |
| CC_1/2_ | 0.997 (0.953) | 0.992 (0.98) | 1 (0.995) |
| Completeness (%) | 89.41 (93.11) | 99.78 (100.00) | 99.97 (100.00) |
| Multiplicity | 4.6 (4.6) | 6.2 (6.6) | 12.6 (13.2) |
| **Refinement** |  |  |  |
| R_work_/R_free_ (%) | 19.08/21.88 | 18.17/21.44 | 19.83/22.28 |
| No. protein atoms | 1016 | 2137 | 1008 |
| No. ligand atoms | 0 | 0 | 0 |
| No. solvent atoms | 110 | 249 | 135 |
| Average B-factor (Å^2^) | 21.27 | 21.86 | 22.85 |
| Protein B-factor (Å^2^) | 20.36 | 21.17 | 21.71 |
| Solvent B-factor (Å^2^) | 29.65 | 27.76 | 31.37 |
| Rmsd bond lengths (Å) | 0.006 | 0.008 | 0.009 |
| Rmsd bond angles (°) | 1.00 | 1.02 | 1.18 |
| Ramachandran outliers (%) | 0.00 | 0.00 | 0.00 |
| Ramachandran favored (%) | 97.64 | 98.18 | 98.40 |

Values in parentheses are for the highest-resolution shell.

**Data Collection and Refinement Statistics part 3**

| Data Set | RPA70N-MRE11 | RPA70N-RAD9 | RPA70N-ETAA1 |
| --- | --- | --- | --- |
| **Data collection** |  |  |  |
| PDB code | 8JZY | 8K00 | 8JZV |
| Space group | *P 21 21 21* | *P 32 2 1* | *P 32 2 1* |
| *a*, *b*, *c* (Å) | 38.9 53.705 55.329 | 50.041 50.041 93.255 | 50.209 50.209 94.526 |
| *α*, *β*, *γ* (°) | 90, 90, 90 | 90, 90,120 | 90, 90, 120 |
| Resolution (Å) | 27.38 - 1.4 (1.43 - 1.4) | 25.26 - 1.5 (1.53 - 1.5) | 39.5 - 1.5 (1.54 - 1.5) |
| Observed reflections | 147295 (8244) | 213852 (13766) | 213063 (12979) |
| Unique reflections | 23471 (1442) | 22322 (1472) | 22757 (1483) |
| R_merge_ (%) | 5.82 (8.074) | 5.98 (55.31) | 5.676 (22.13) |
| R_pim_ (%) | 2.54 (3.673) | 2.095 (19) | 2.001 (7.917) |
| I/σ(I) | 23.61 (15.28) | 20.11 (3.74) | 26.24 (9.76) |
| CC_1/2_ | 0.996 (0.994) | 0.998 (0.927) | 0.997 (0.981) |
| Completeness (%) | 99.82 (99.09) | 99.98 (99.96) | 99.96 (99.82) |
| Multiplicity | 6.3 (5.7) | 9.6 (9.4) | 9.4 (8.8) |
| **Refinement** |  |  |  |
| R_work_/R_free_ (%) | 16.74/19.28 | 18.48/19.91 | 18.45/20.03 |
| No. protein atoms | 1111 | 1009 | 1030 |
| No. ligand atoms | 0 | 0 | 0 |
| No. solvent atoms | 228 | 102 | 141 |
| Average B-factor (Å^2^) | 15.27 | 34.30 | 25.65 |
| Protein B-factor (Å^2^) | 12.90 | 33.53 | 24.30 |
| Solvent B-factor (Å^2^) | 26.83 | 41.88 | 35.49 |
| Rmsd bond lengths (Å) | 0.009 | 0.008 | 0.013 |
| Rmsd bond angles (°) | 1.15 | 1.05 | 1.32 |
| Ramachandran outliers (%) | 0.00 | 0.00 | 0.00 |
| Ramachandran favored (%) | 97.89 | 97.58 | 96.85 |

Values in parentheses are for the highest-resolution shell.
