# Supplementary figures and images for: Structural characterization of human RPA70N association with DNA damage response proteins (part 1 of 2)
Source: eLife. 2023 Sep 5;12:e81639. doi: 10.7554/eLife.81639 (PMC10479964; doi:10.7554/eLife.81639)

Figure 2K  
HeIB

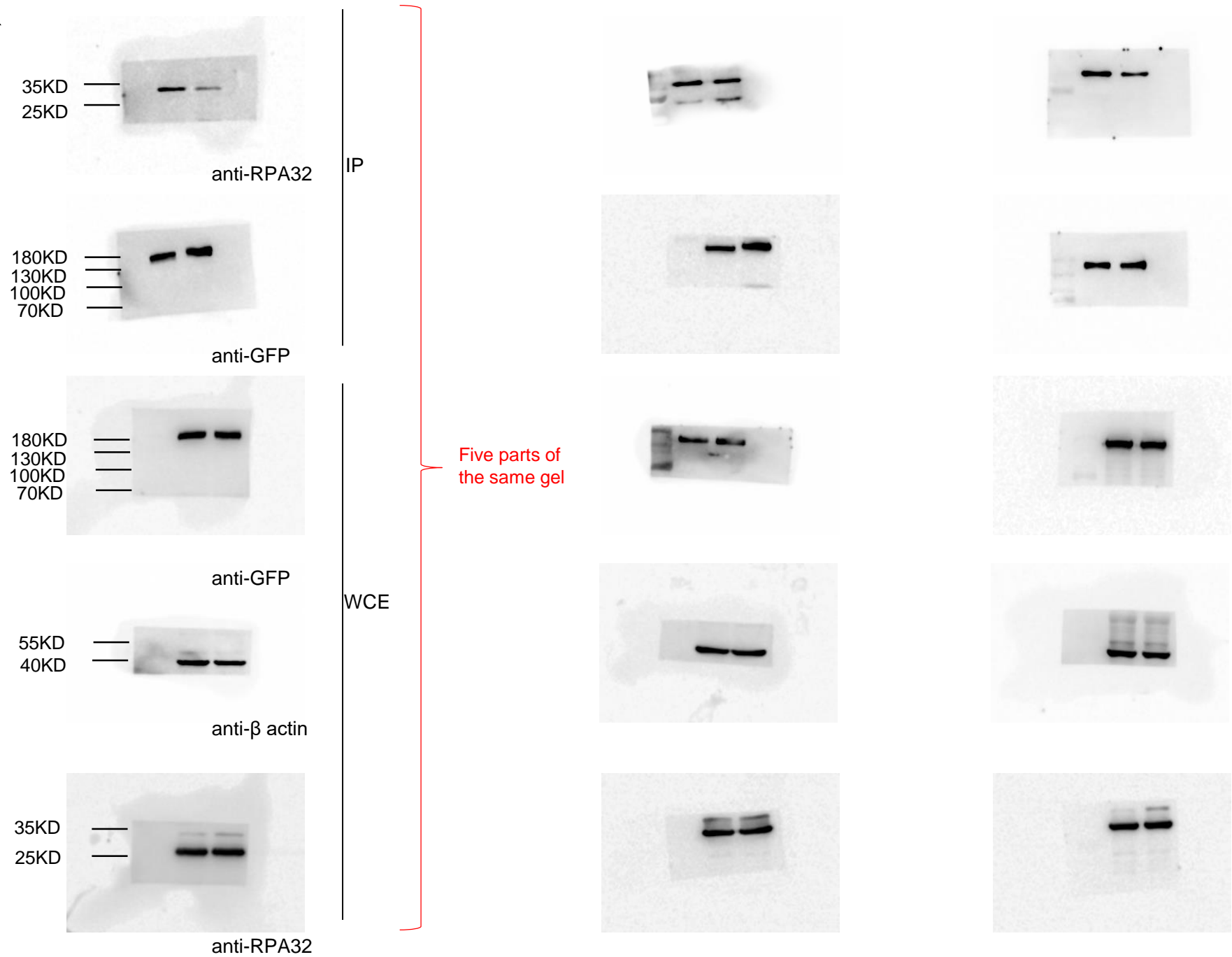

Supplement: Figure 2—source data 1. [file elife-81639-fig2-data1.zip › Figure2-source data/IP-data-Figure 2K.pdf]

Figure 2K  
HeIB

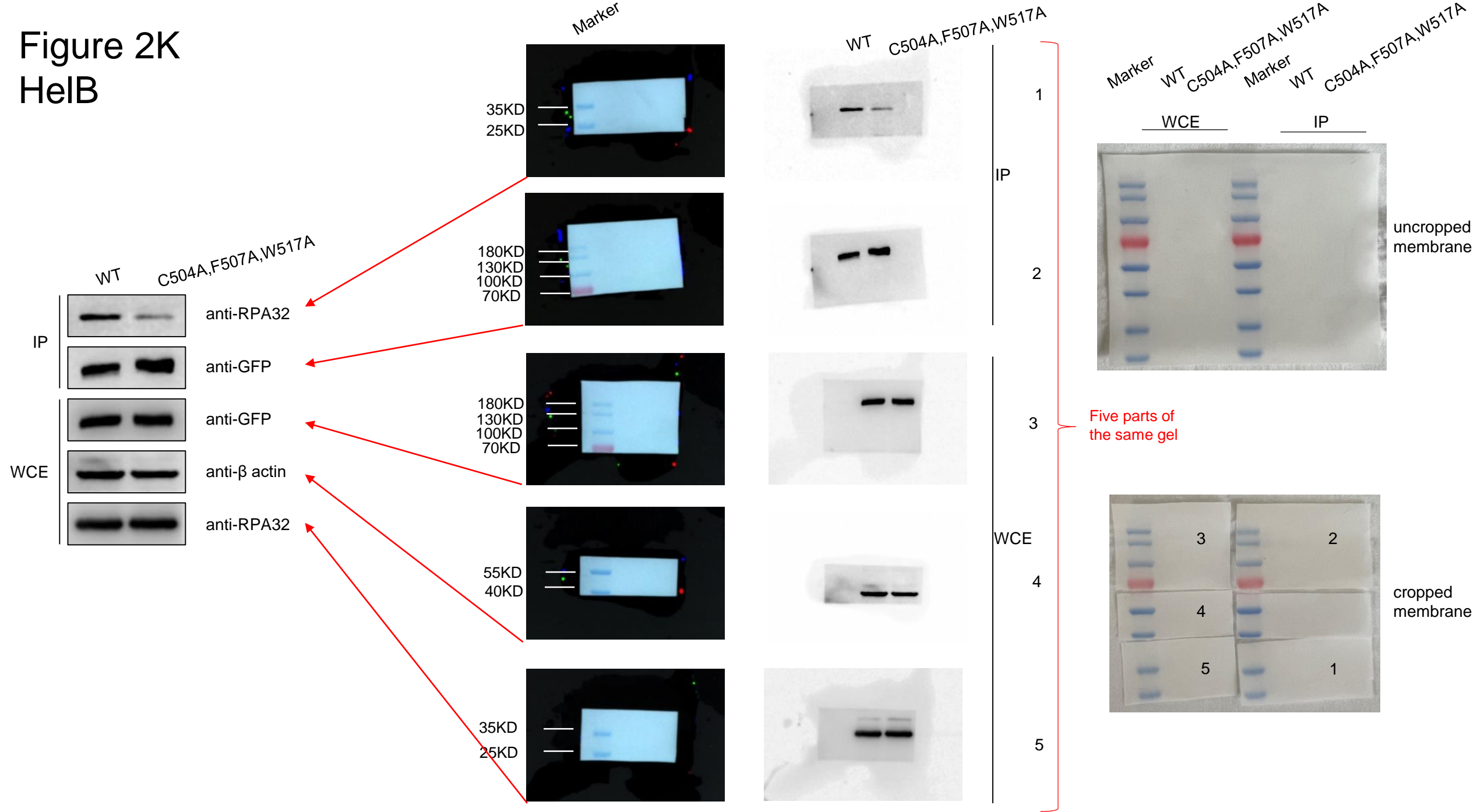

Supplement: Figure 2—source data 1. [file elife-81639-fig2-data1.zip › Figure2-source data/Figure 2K.pdf]

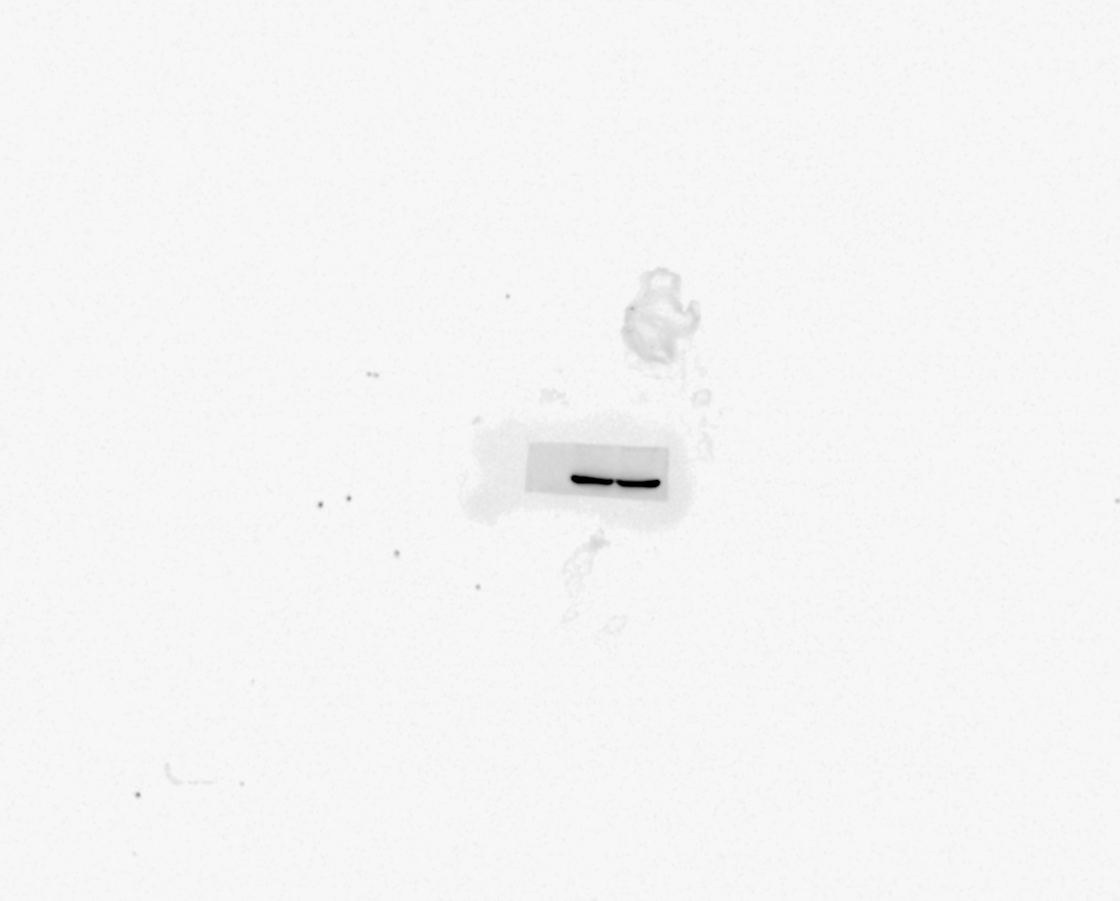

Supplement: Figure 2—source data 1. [file elife-81639-fig2-data1.zip › Figure2-source data/Figure 2K Repeat2/WCE-anti╬▓ actin.tif]

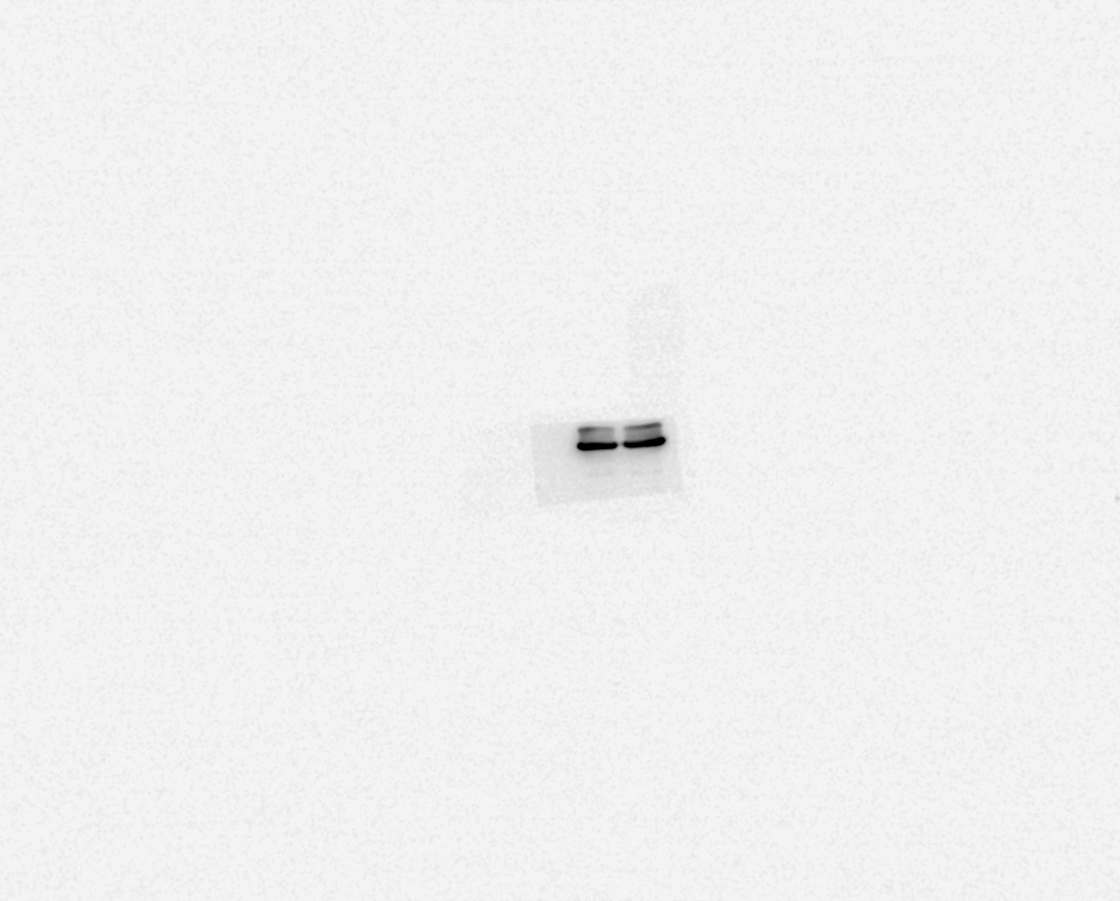

Supplement: Figure 2—source data 1. [file elife-81639-fig2-data1.zip › Figure2-source data/Figure 2K Repeat2/WCE-antiRPA32.tif]

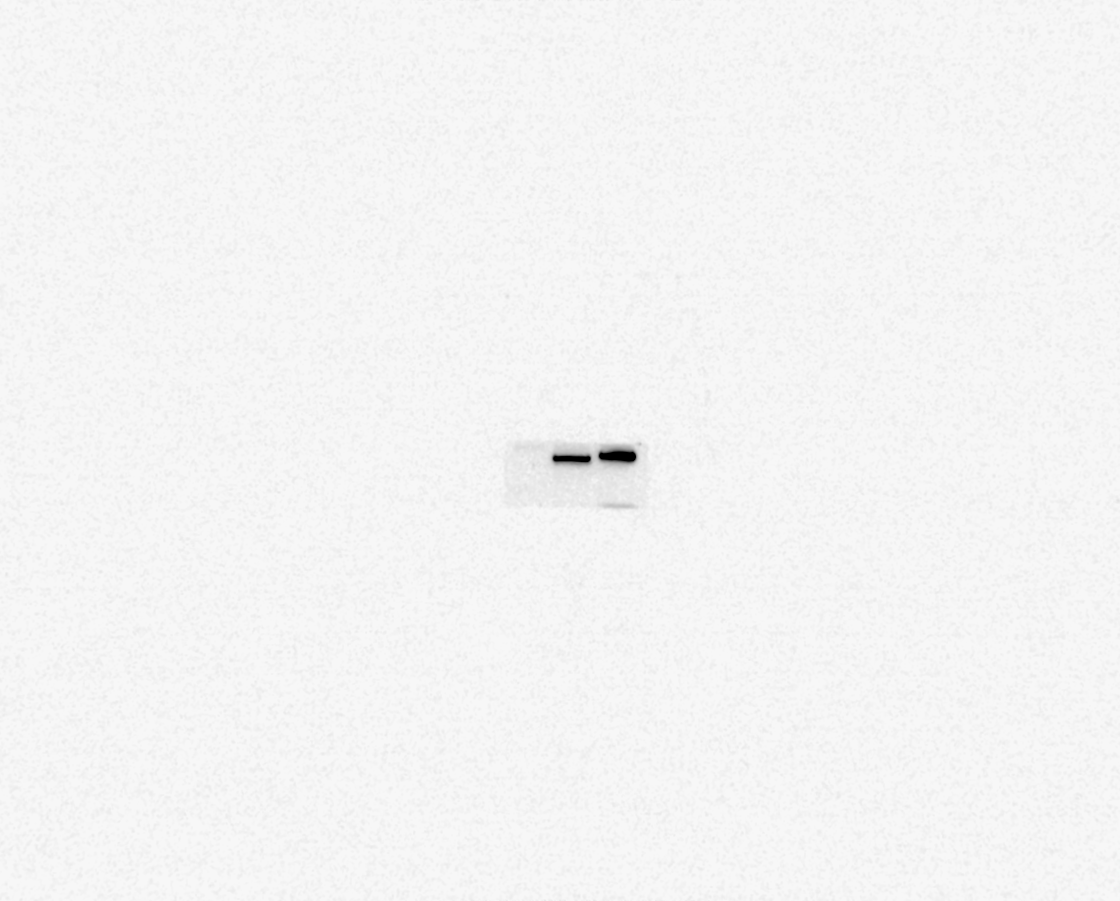

Supplement: Figure 2—source data 1. [file elife-81639-fig2-data1.zip › Figure2-source data/Figure 2K Repeat2/WCE-antiGFP.tif]

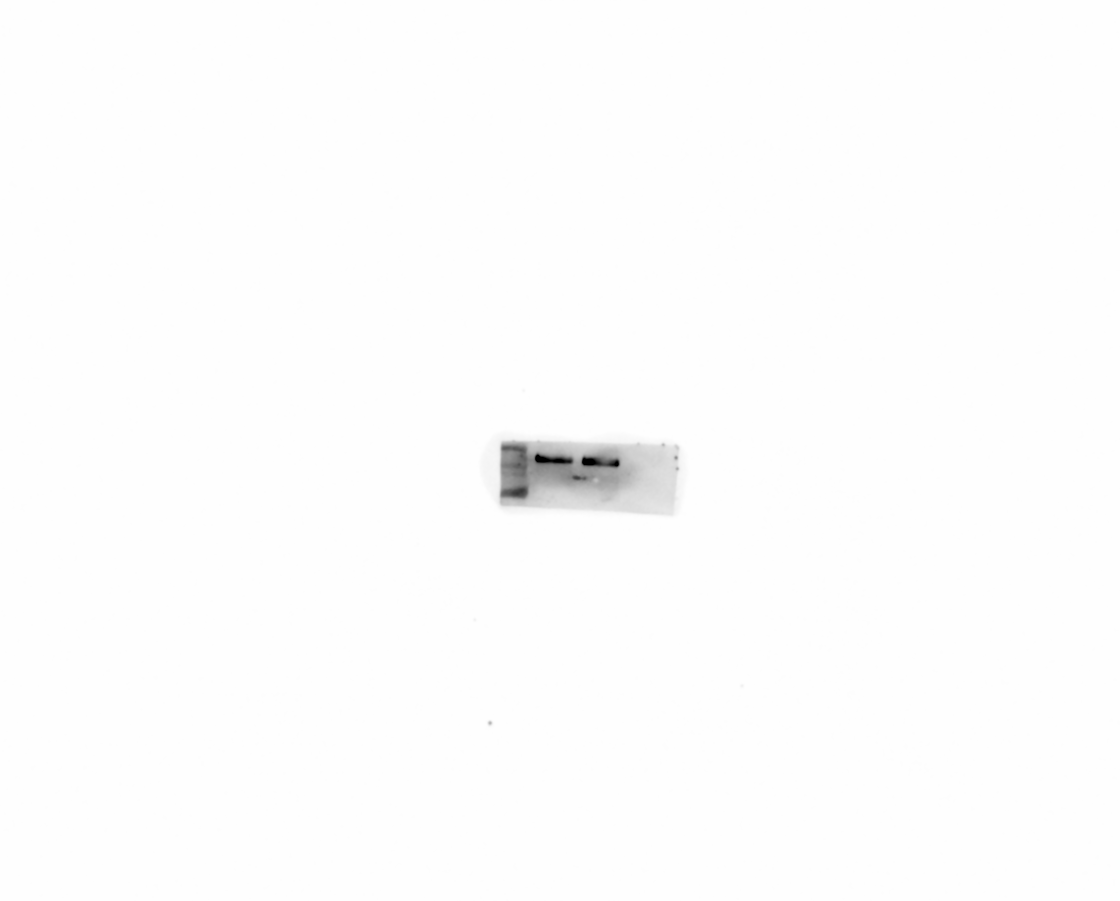

Supplement: Figure 2—source data 1. [file elife-81639-fig2-data1.zip › Figure2-source data/Figure 2K Repeat2/IP-antiGFP.tif]

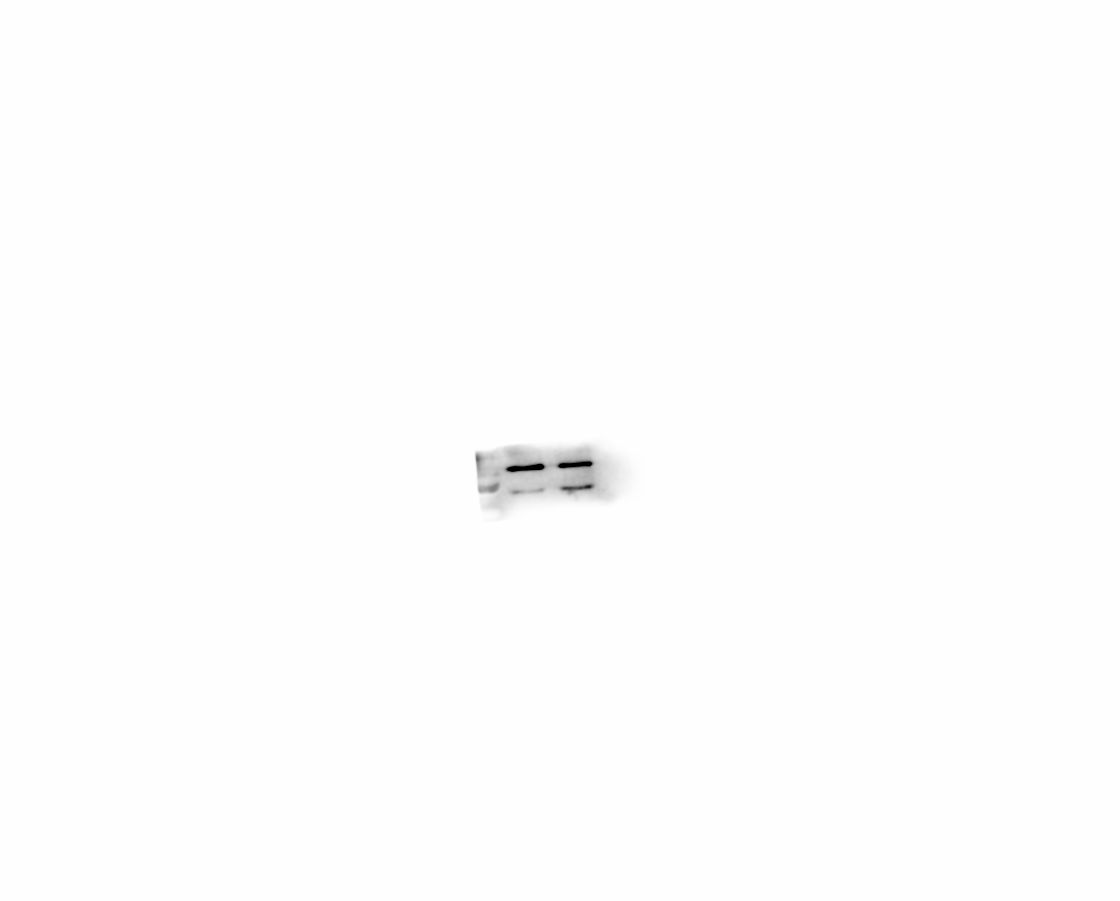

Supplement: Figure 2—source data 1. [file elife-81639-fig2-data1.zip › Figure2-source data/Figure 2K Repeat2/IP-antiRPA32.tif]

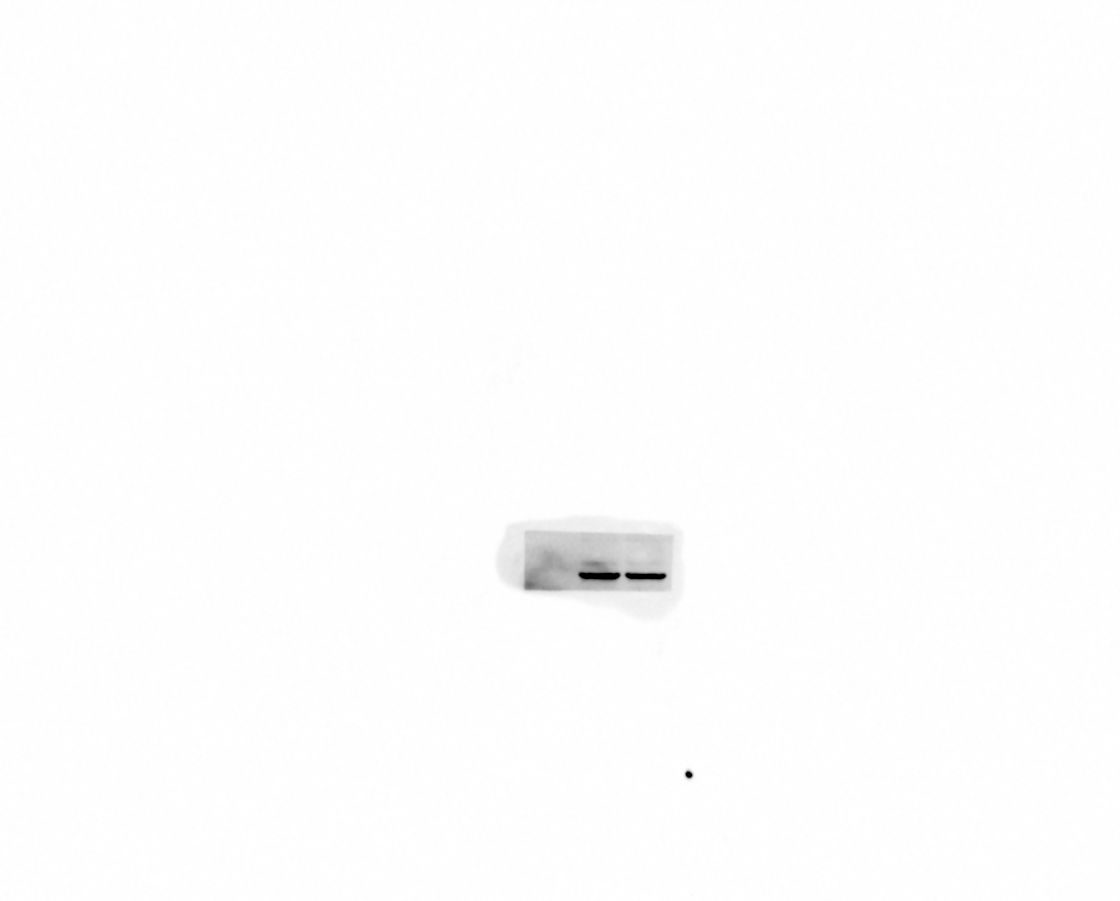

Supplement: Figure 2—source data 1. [file elife-81639-fig2-data1.zip › Figure2-source data/Figure 2K initial trial/WCE-anti╬▓ actin.tif]

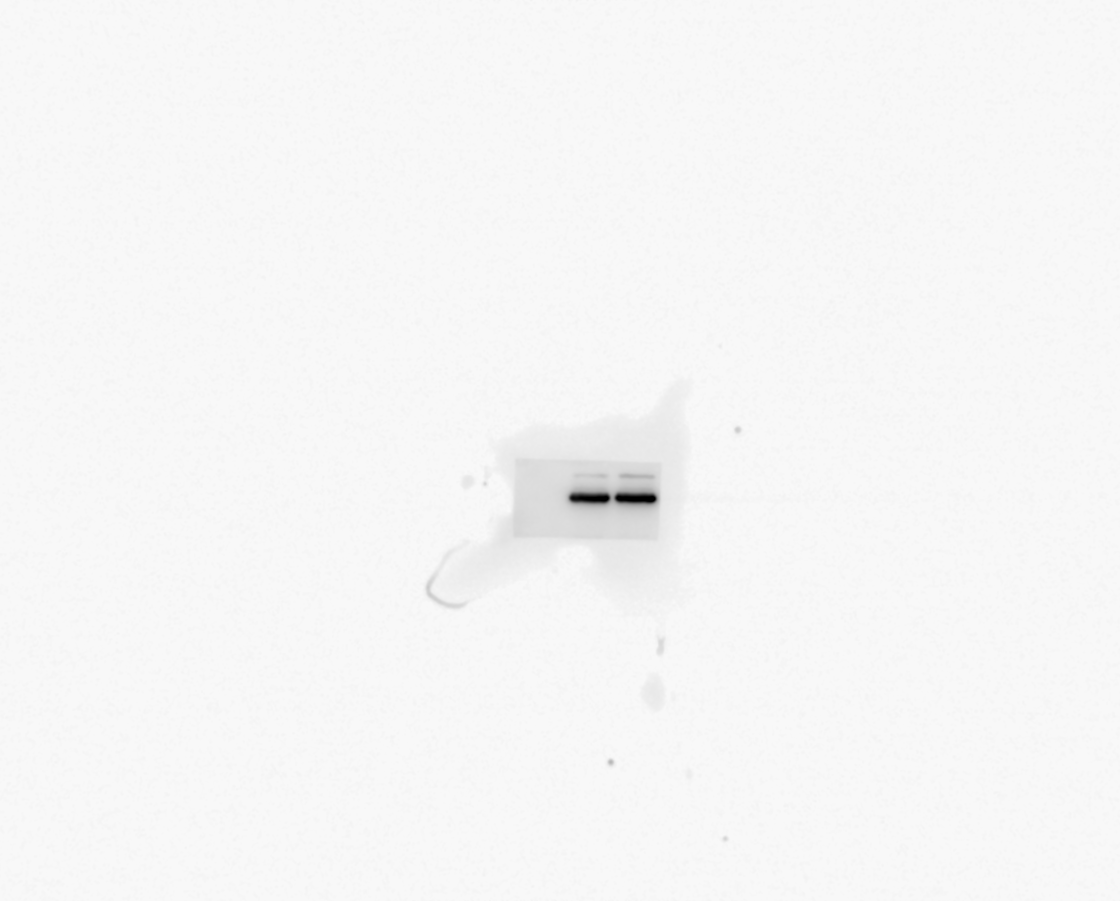

Supplement: Figure 2—source data 1. [file elife-81639-fig2-data1.zip › Figure2-source data/Figure 2K initial trial/WCE-antiRPA32.tif]

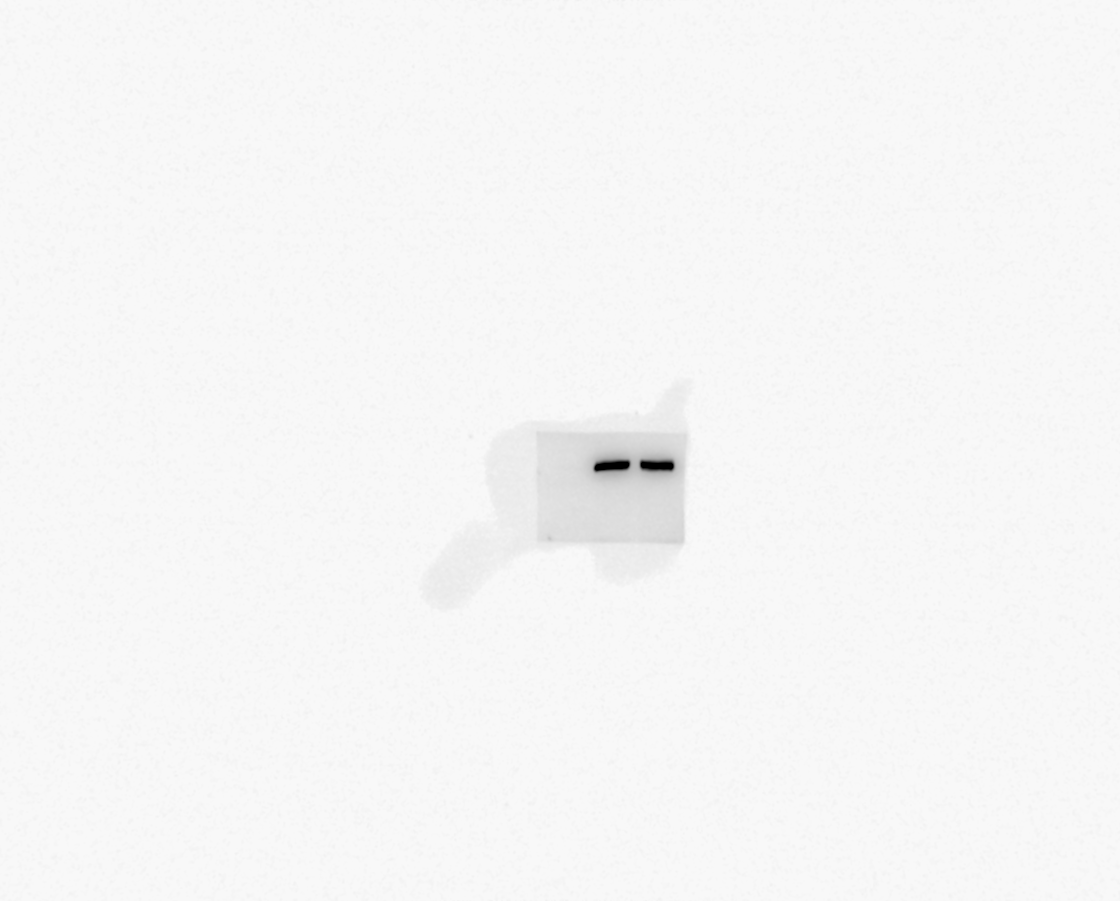

Supplement: Figure 2—source data 1. [file elife-81639-fig2-data1.zip › Figure2-source data/Figure 2K initial trial/WCE-antiGFP.tif]

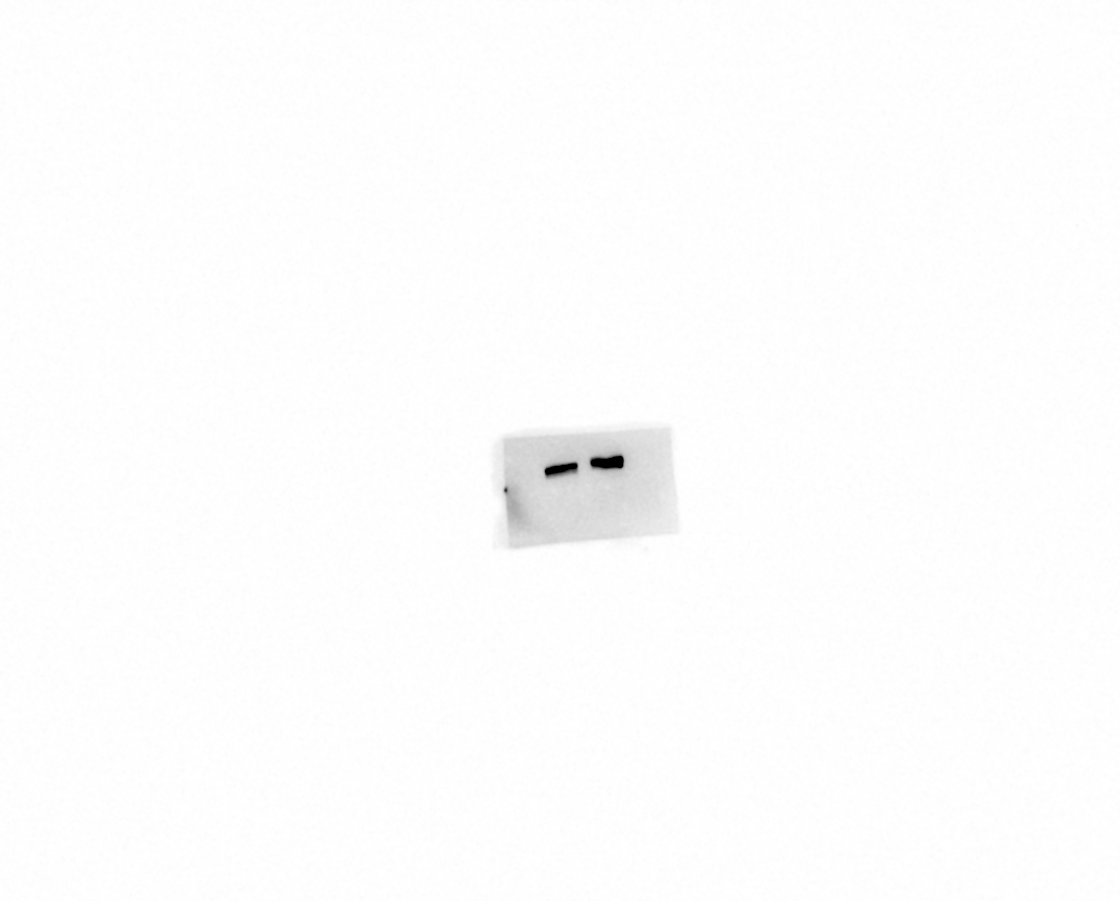

Supplement: Figure 2—source data 1. [file elife-81639-fig2-data1.zip › Figure2-source data/Figure 2K initial trial/IP-antiGFP.tif]

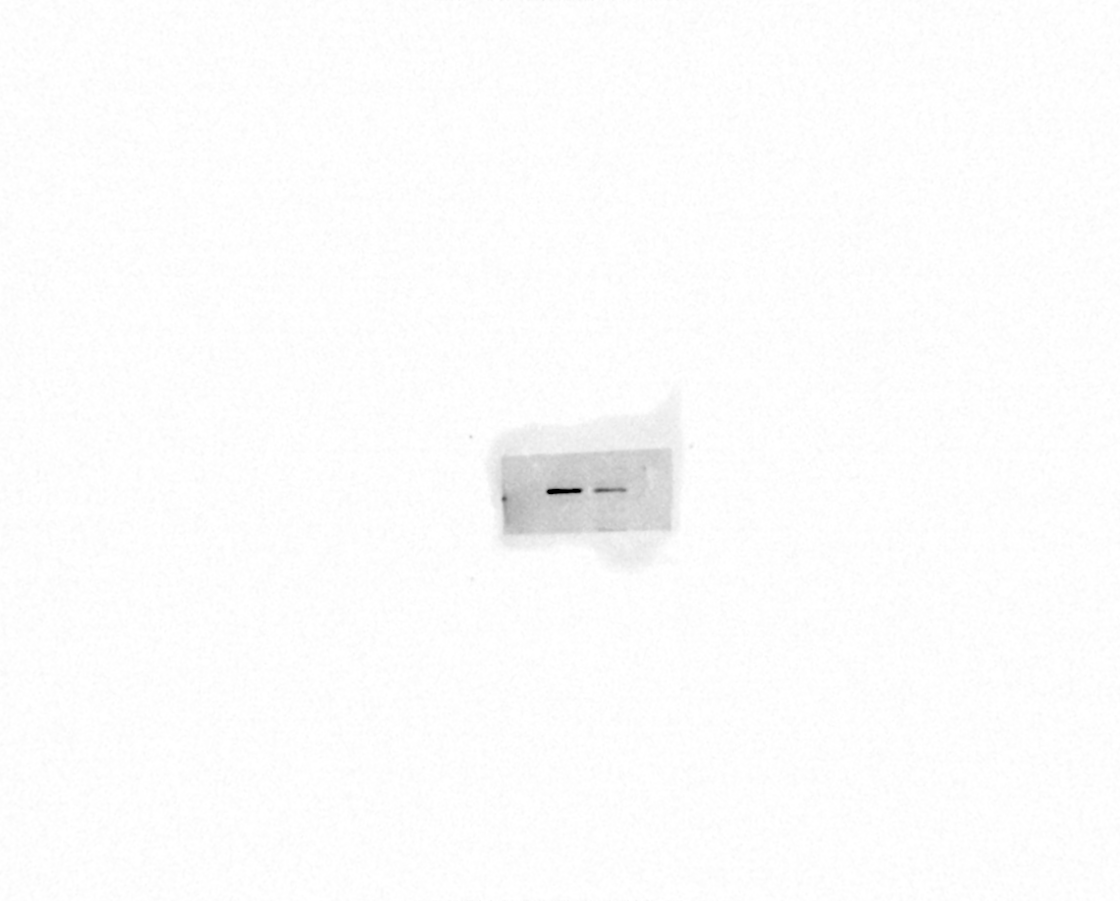

Supplement: Figure 2—source data 1. [file elife-81639-fig2-data1.zip › Figure2-source data/Figure 2K initial trial/IP-antiRPA32.tif]

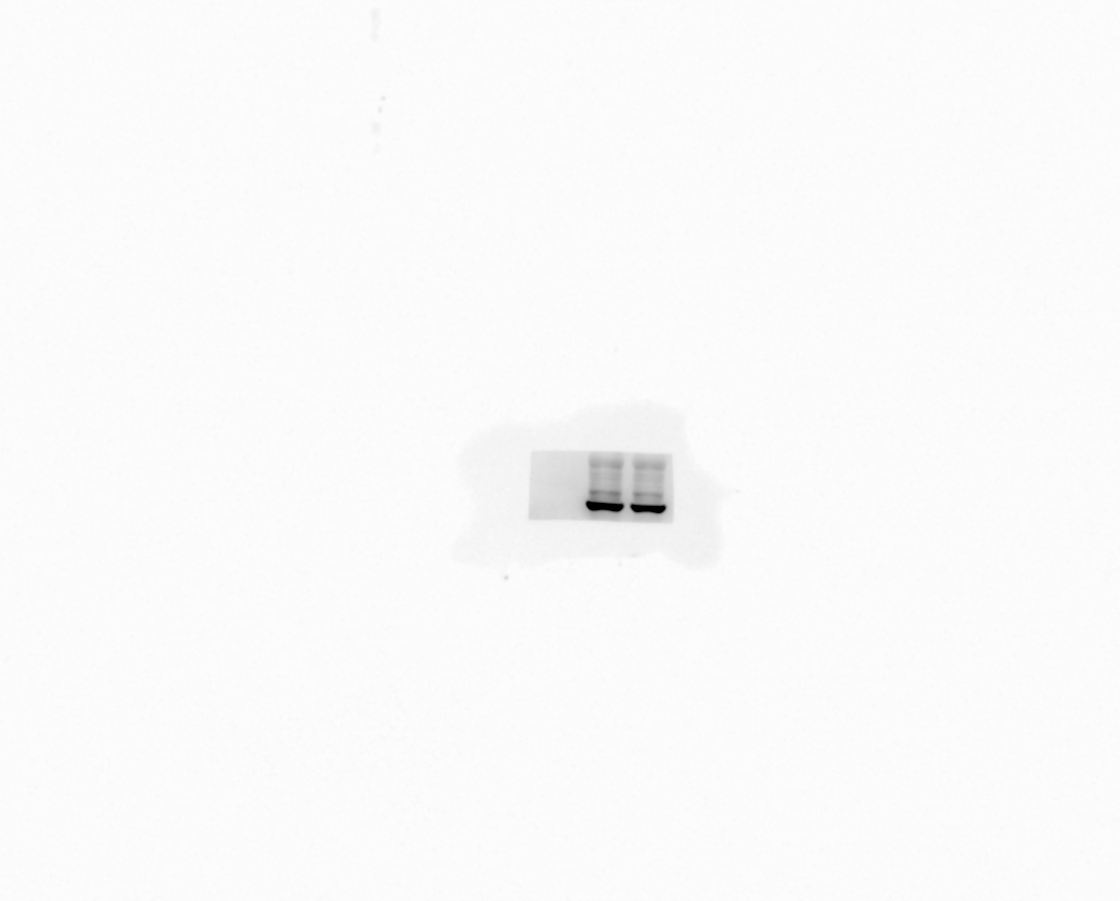

Supplement: Figure 2—source data 1. [file elife-81639-fig2-data1.zip › Figure2-source data/Figure 2K Repeat1/WCE-anti╬▓ actin.tif]

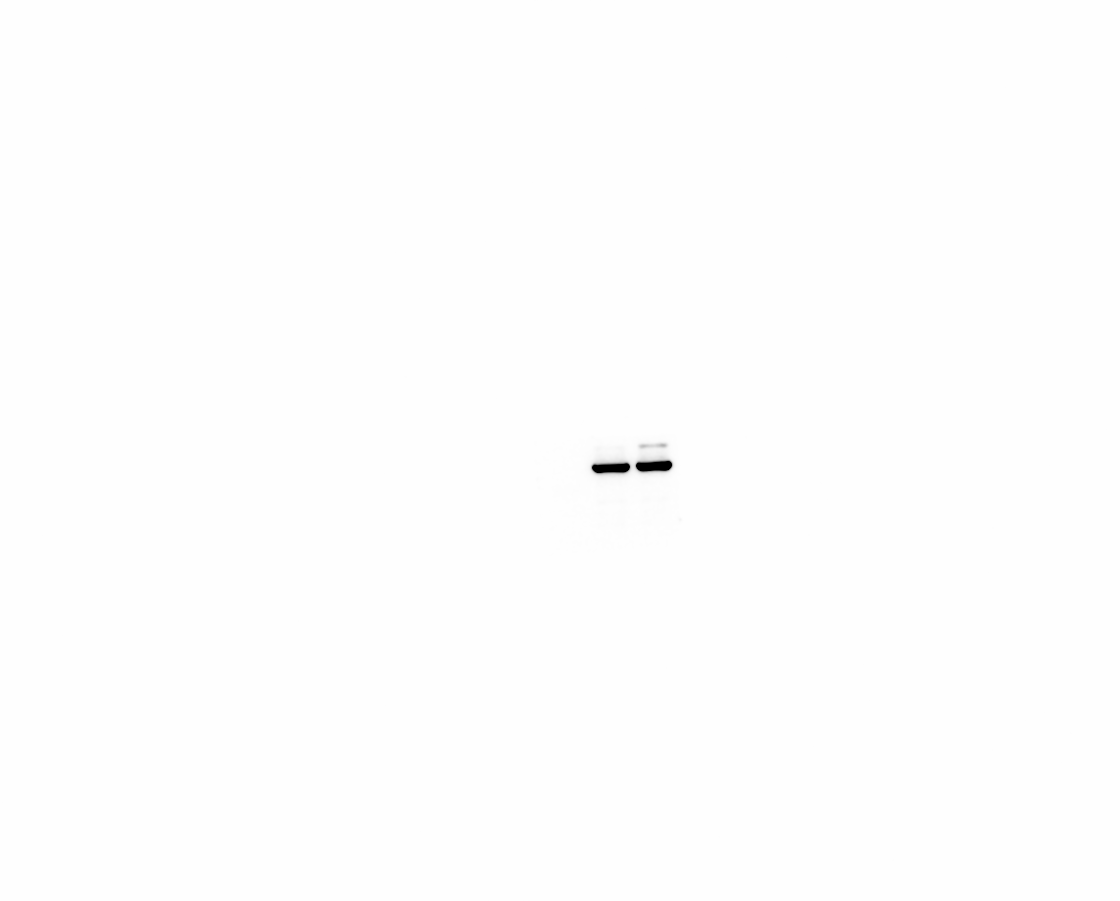

Supplement: Figure 2—source data 1. [file elife-81639-fig2-data1.zip › Figure2-source data/Figure 2K Repeat1/WCE-antiRPA32.tif]

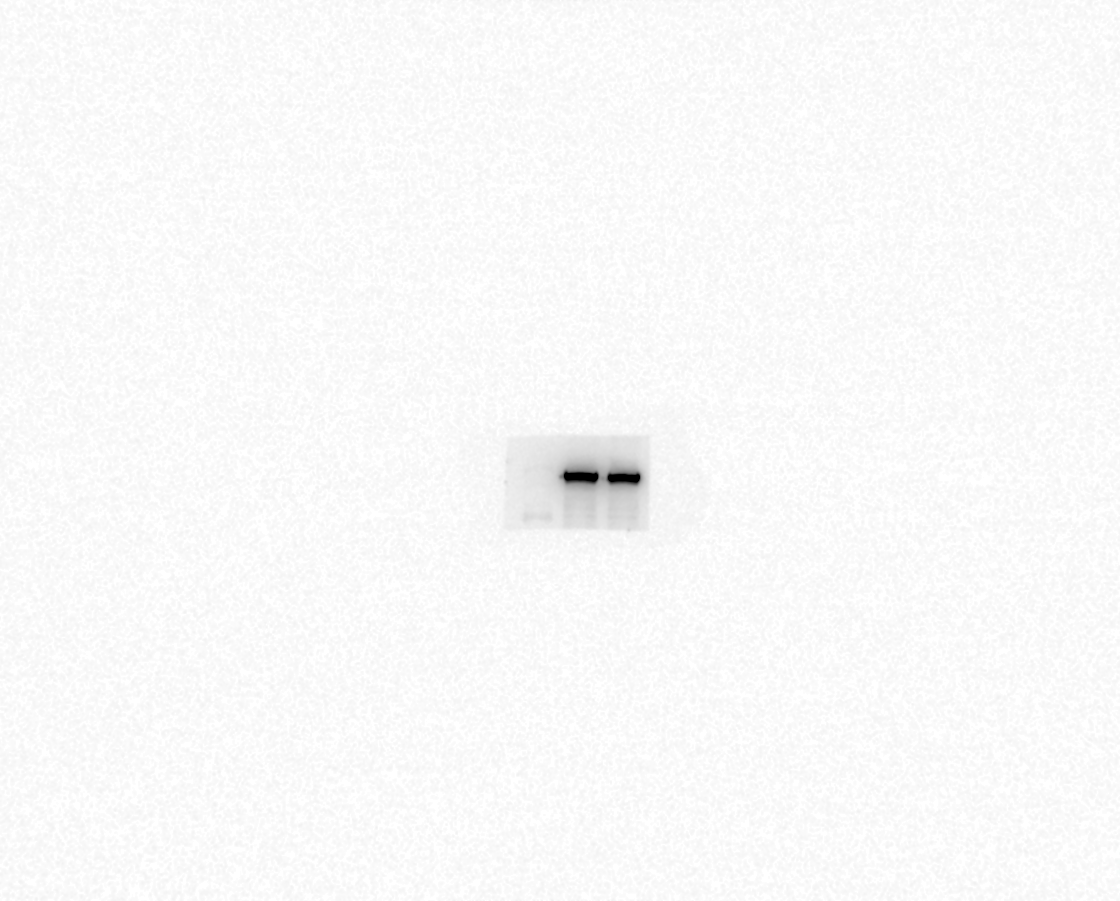

Supplement: Figure 2—source data 1. [file elife-81639-fig2-data1.zip › Figure2-source data/Figure 2K Repeat1/WCE-antiGFP.tif]

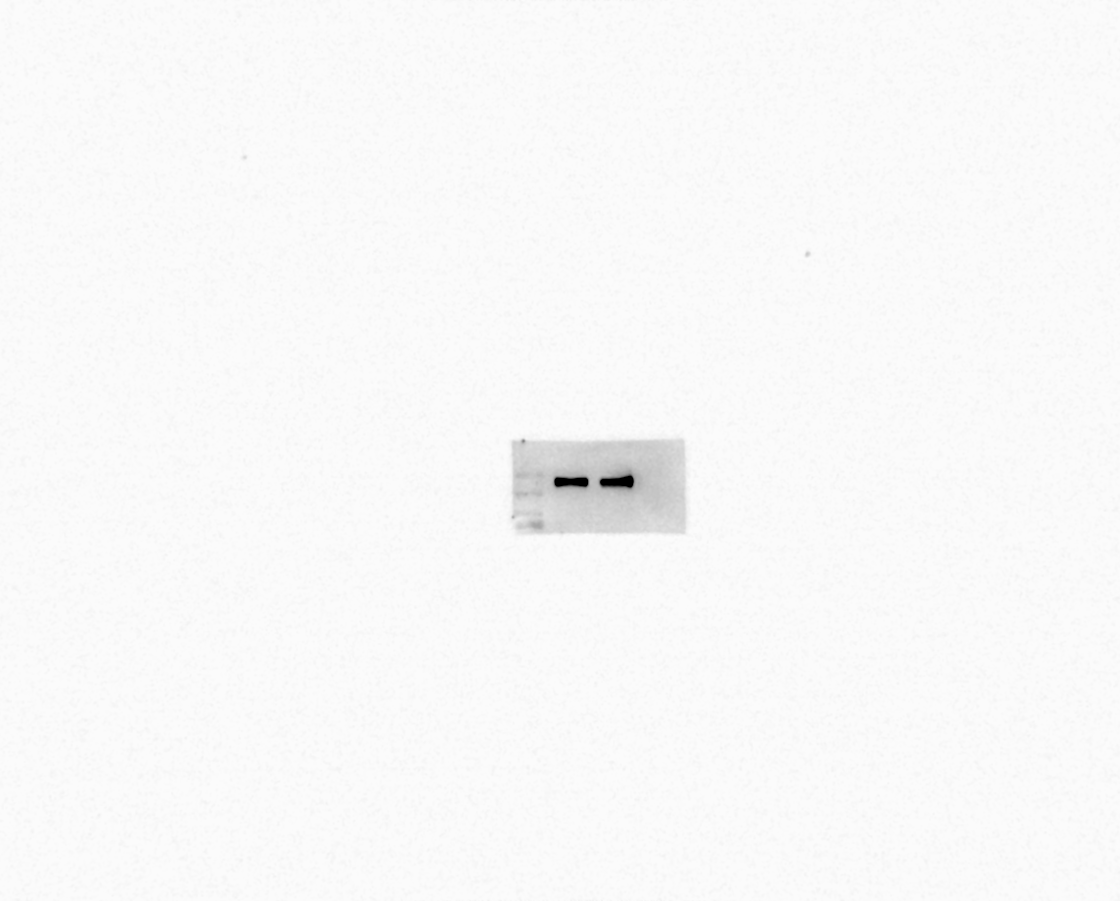

Supplement: Figure 2—source data 1. [file elife-81639-fig2-data1.zip › Figure2-source data/Figure 2K Repeat1/IP-antiGFP.tif]

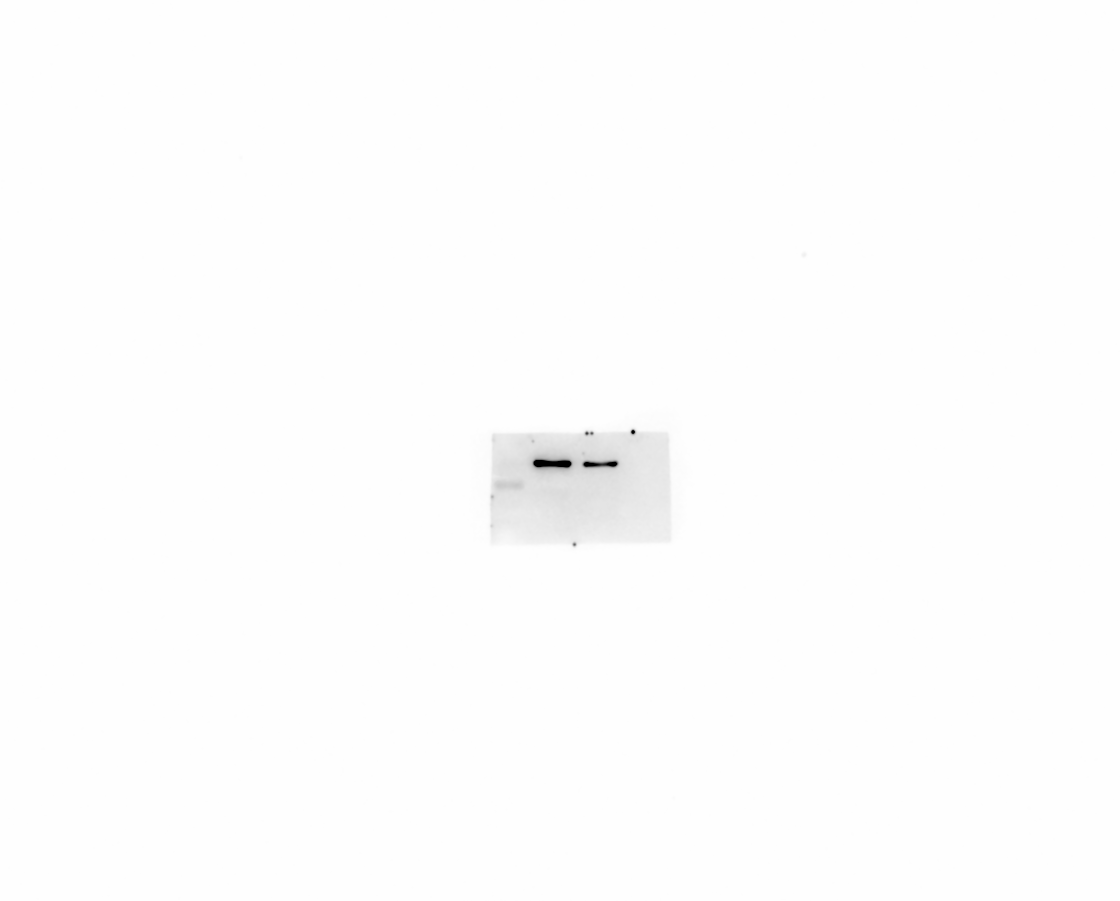

Supplement: Figure 2—source data 1. [file elife-81639-fig2-data1.zip › Figure2-source data/Figure 2K Repeat1/IP-antiRPA32.tif]

Figure 3N  
BLM

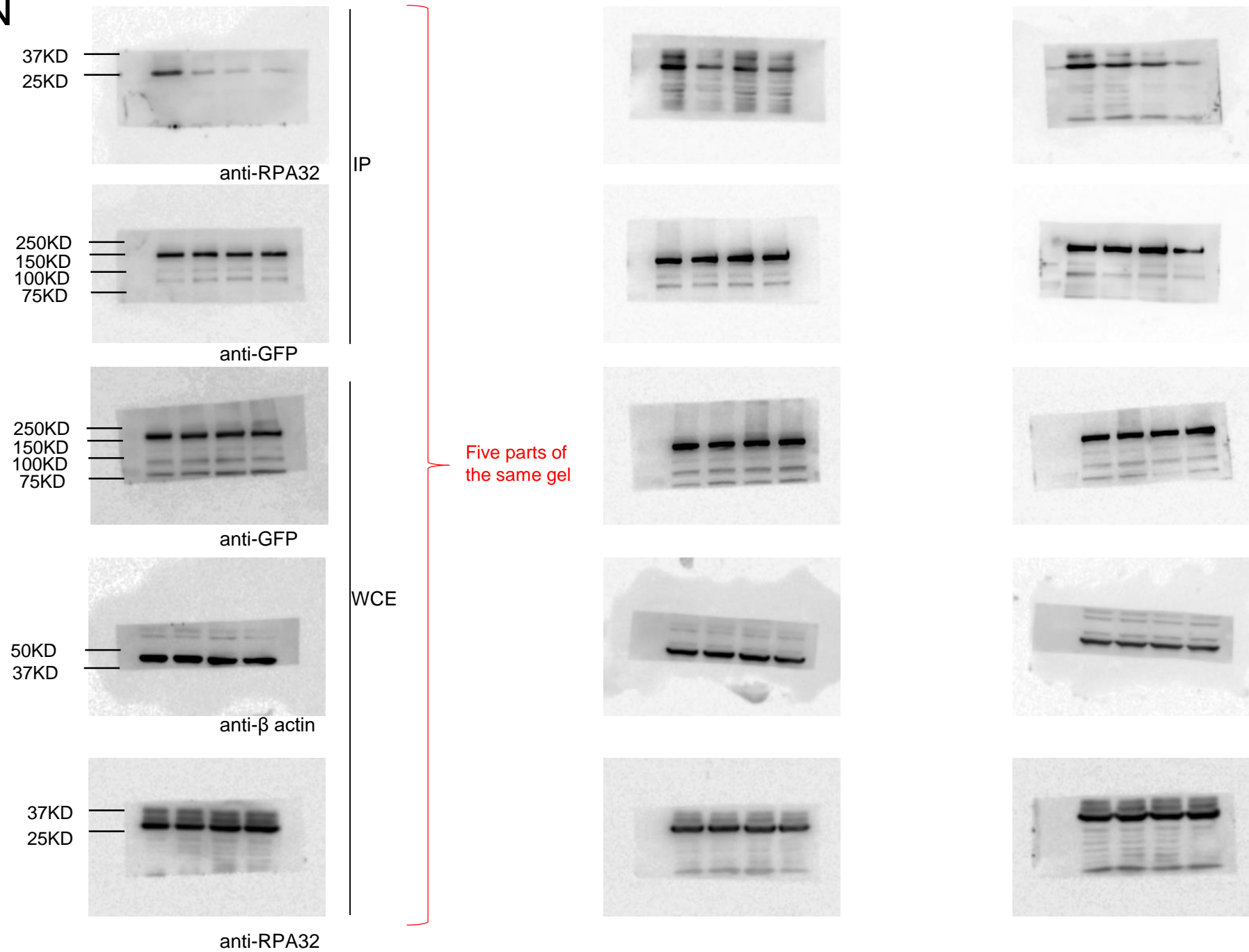

Supplement: Figure 3—source data 1. [file elife-81639-fig3-data1.zip › Figure3-source data/IP-data-Figure 3N.pdf]

Figure 3N  
BLM

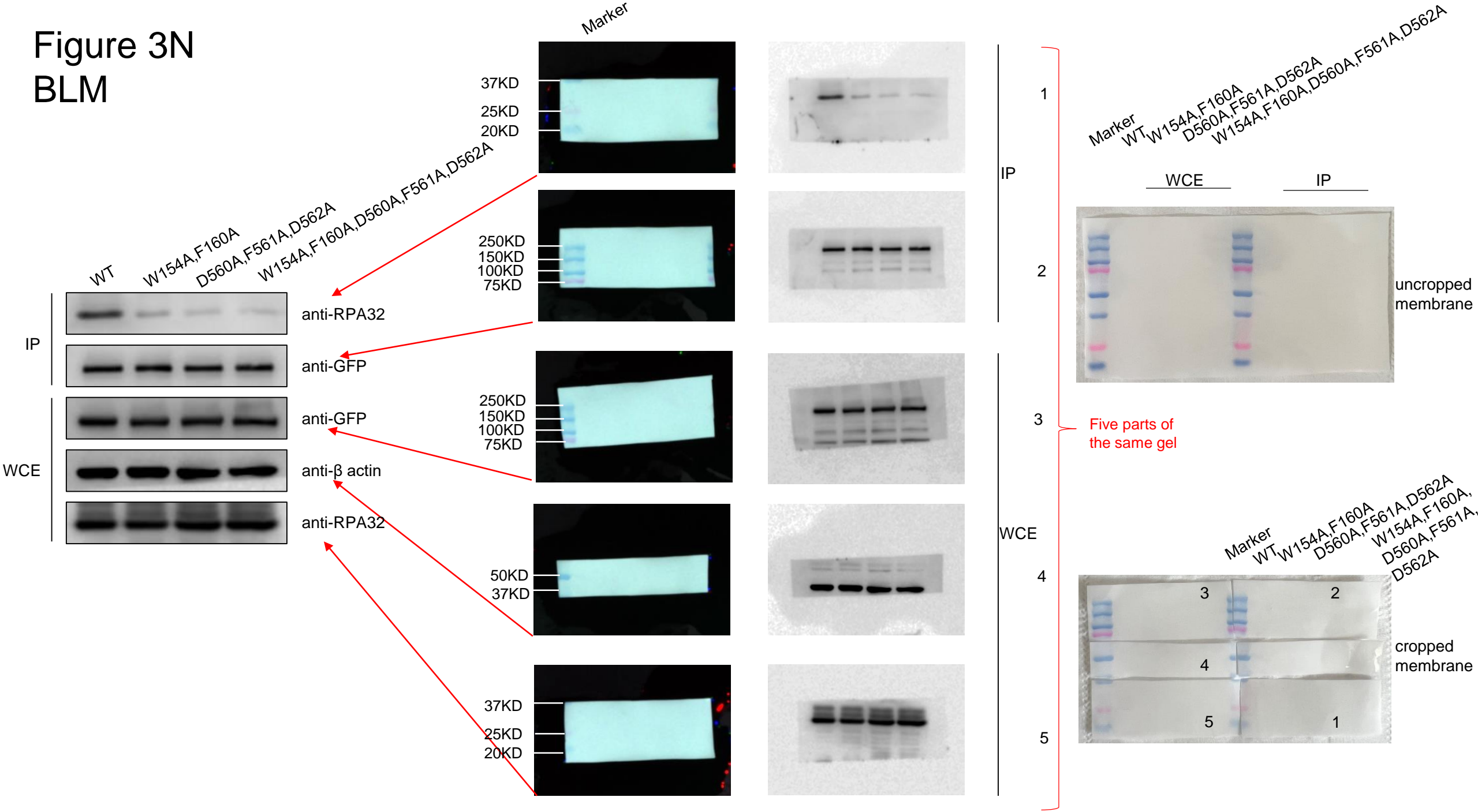

Supplement: Figure 3—source data 1. [file elife-81639-fig3-data1.zip › Figure3-source data/Figure 3N.pdf]

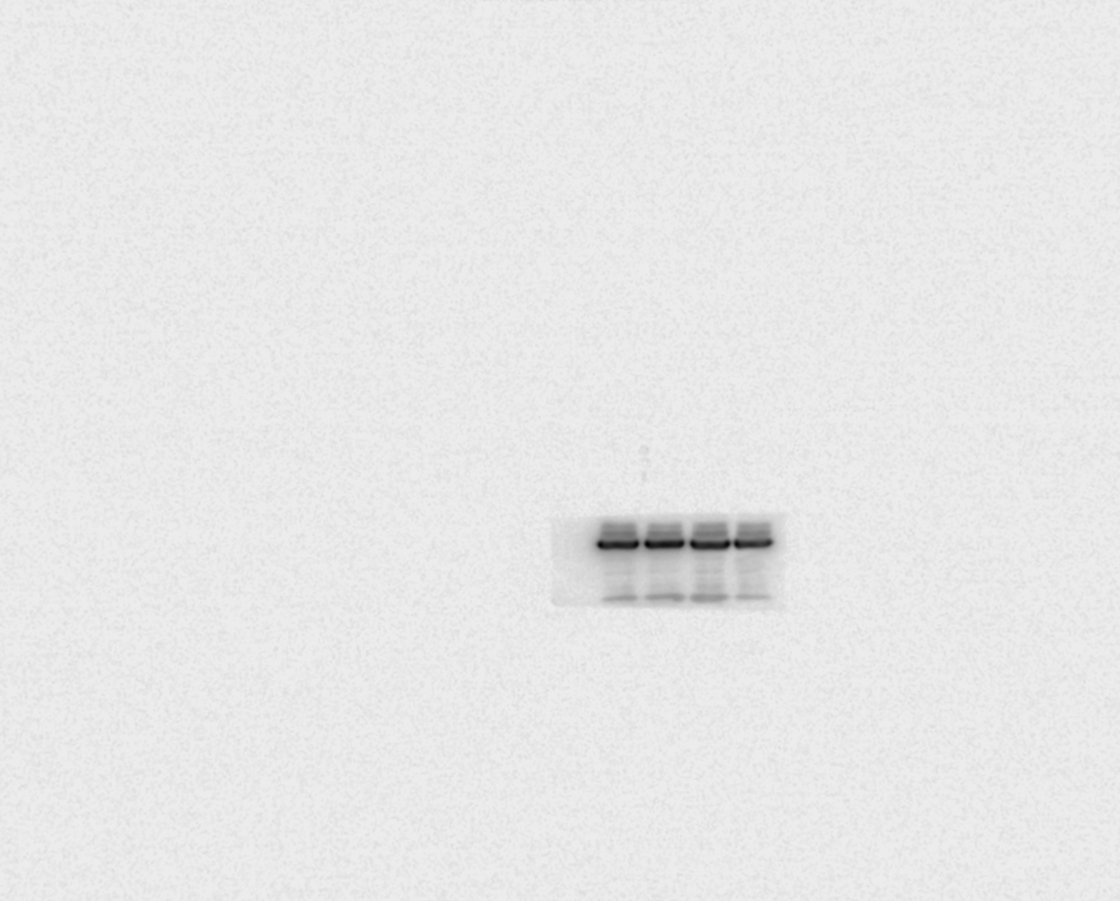

Supplement: Figure 3—source data 1. [file elife-81639-fig3-data1.zip › Figure3-source data/Figure 3N Repeat1/WCE-antiRPA32.tif]

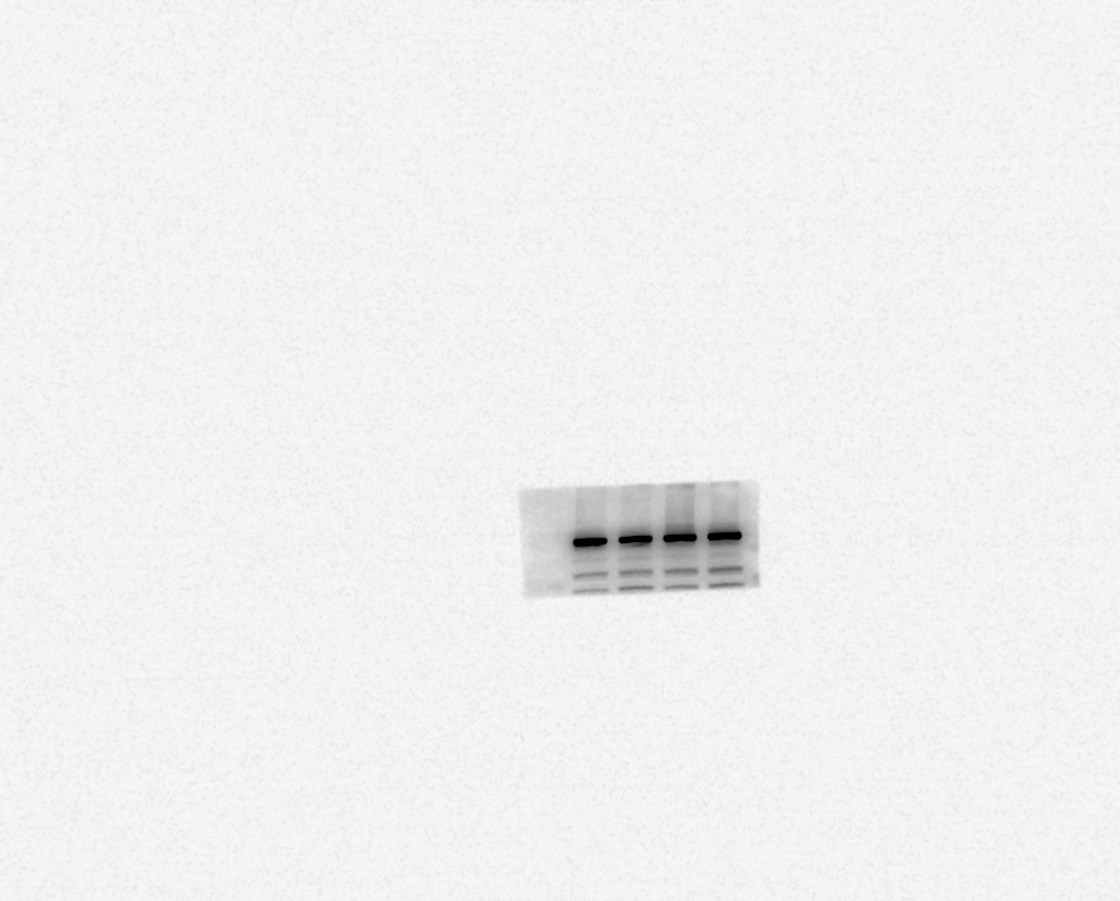

Supplement: Figure 3—source data 1. [file elife-81639-fig3-data1.zip › Figure3-source data/Figure 3N Repeat1/WCE-antiGFP.tif]

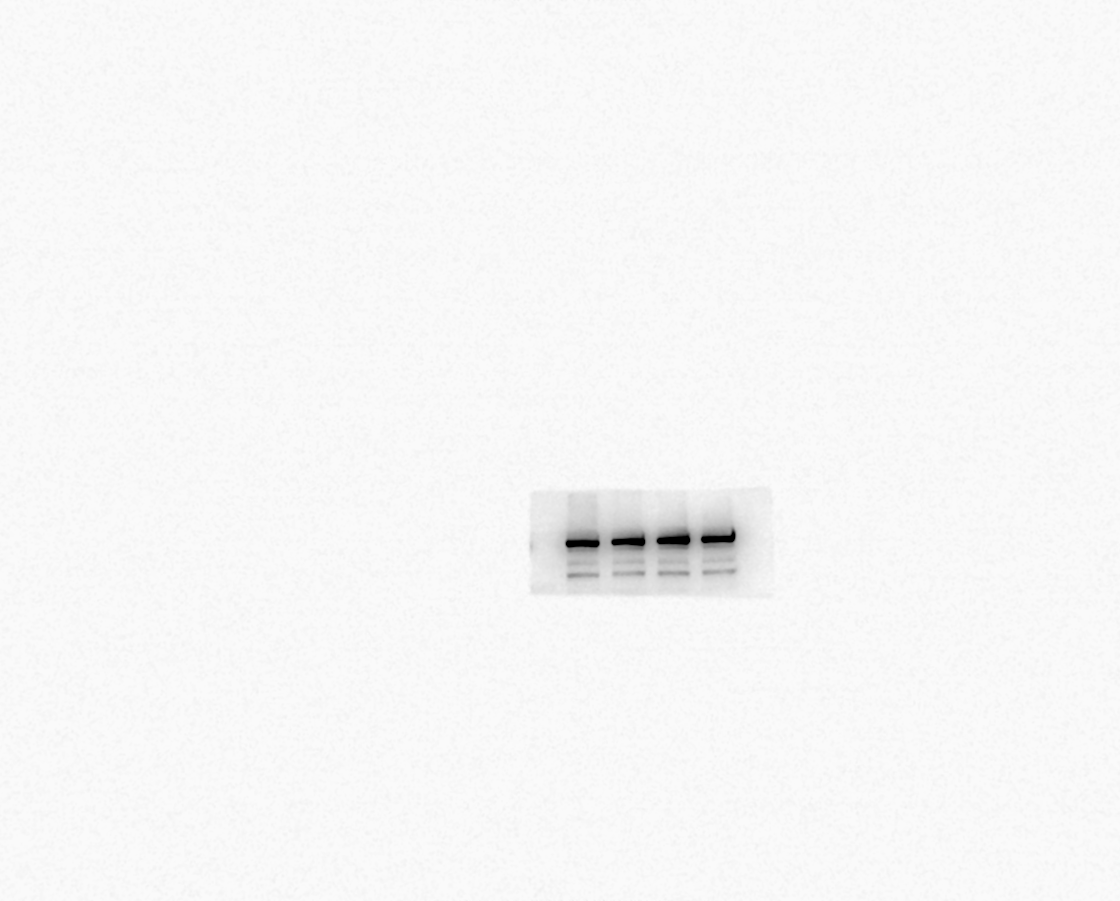

Supplement: Figure 3—source data 1. [file elife-81639-fig3-data1.zip › Figure3-source data/Figure 3N Repeat1/IP-antiGFP.tif]

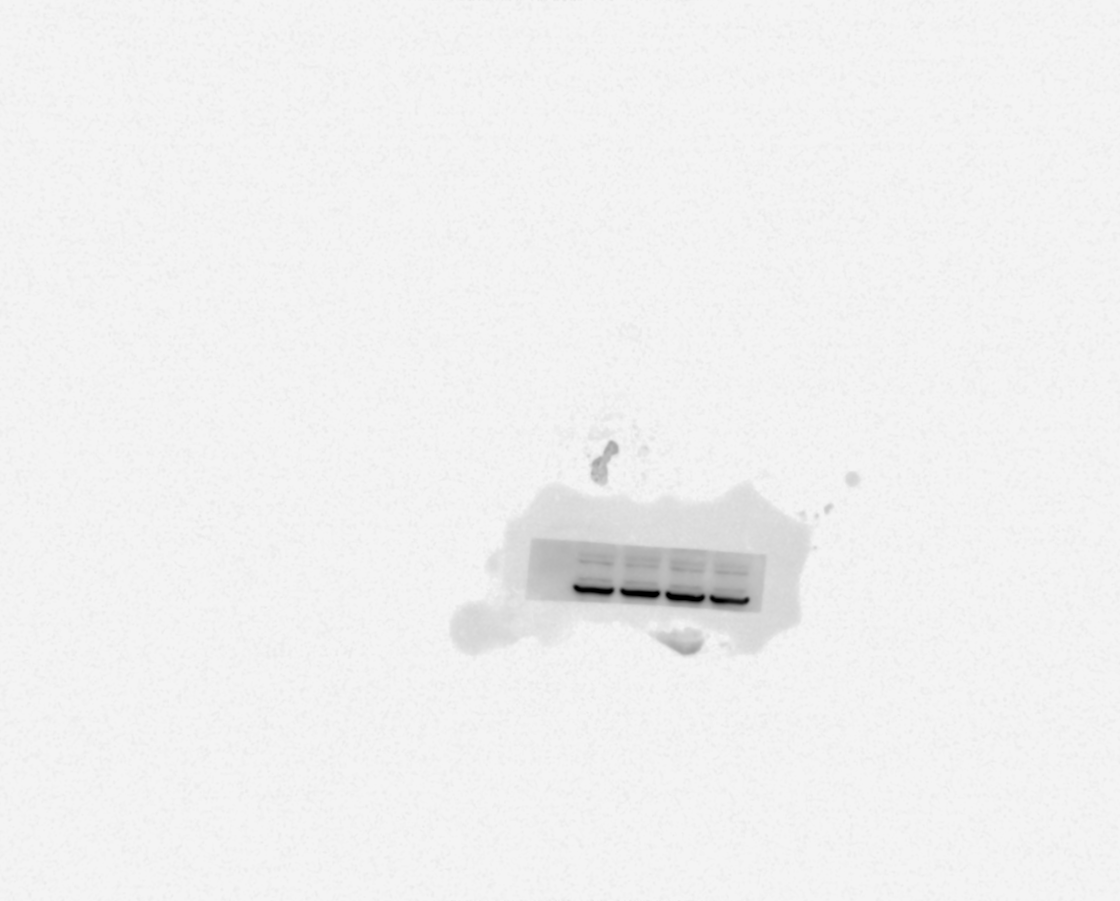

Supplement: Figure 3—source data 1. [file elife-81639-fig3-data1.zip › Figure3-source data/Figure 3N Repeat1/WCE-╬▓ actin.tif]

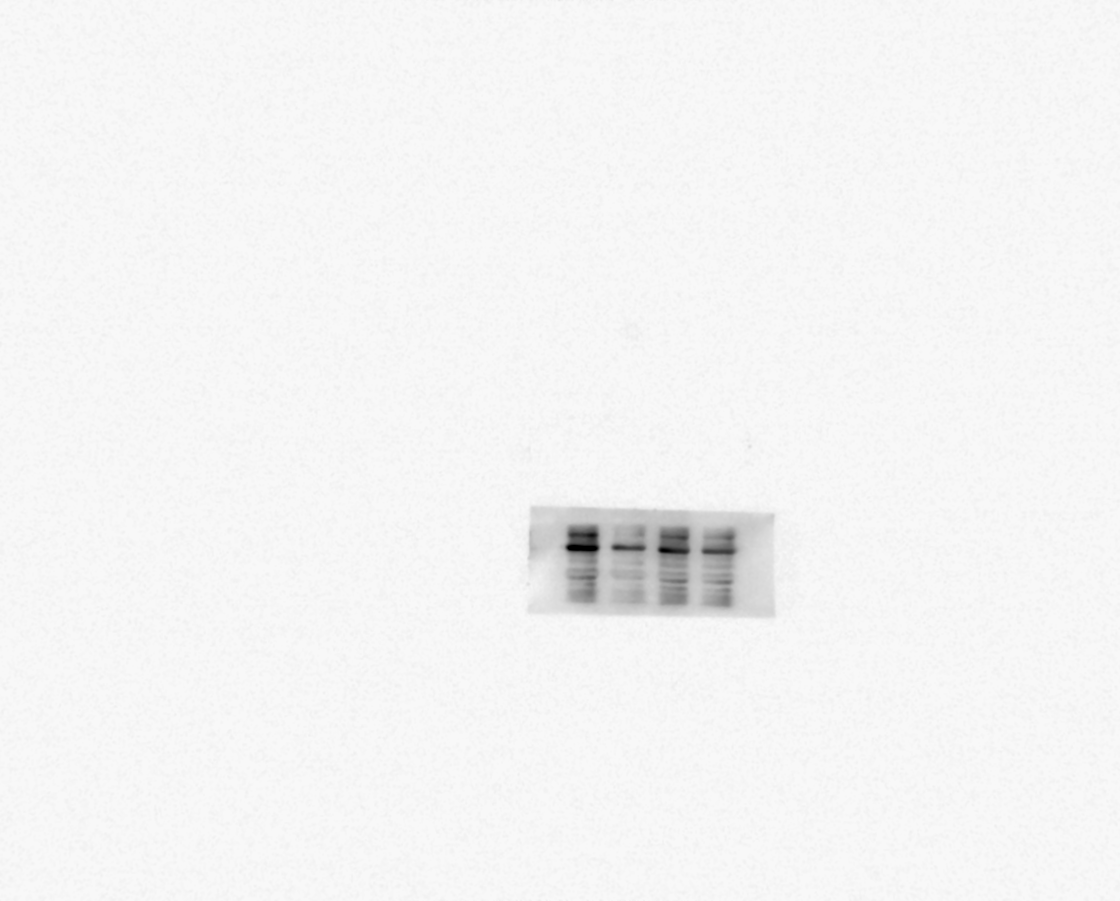

Supplement: Figure 3—source data 1. [file elife-81639-fig3-data1.zip › Figure3-source data/Figure 3N Repeat1/IP-antiPRA32.tif]

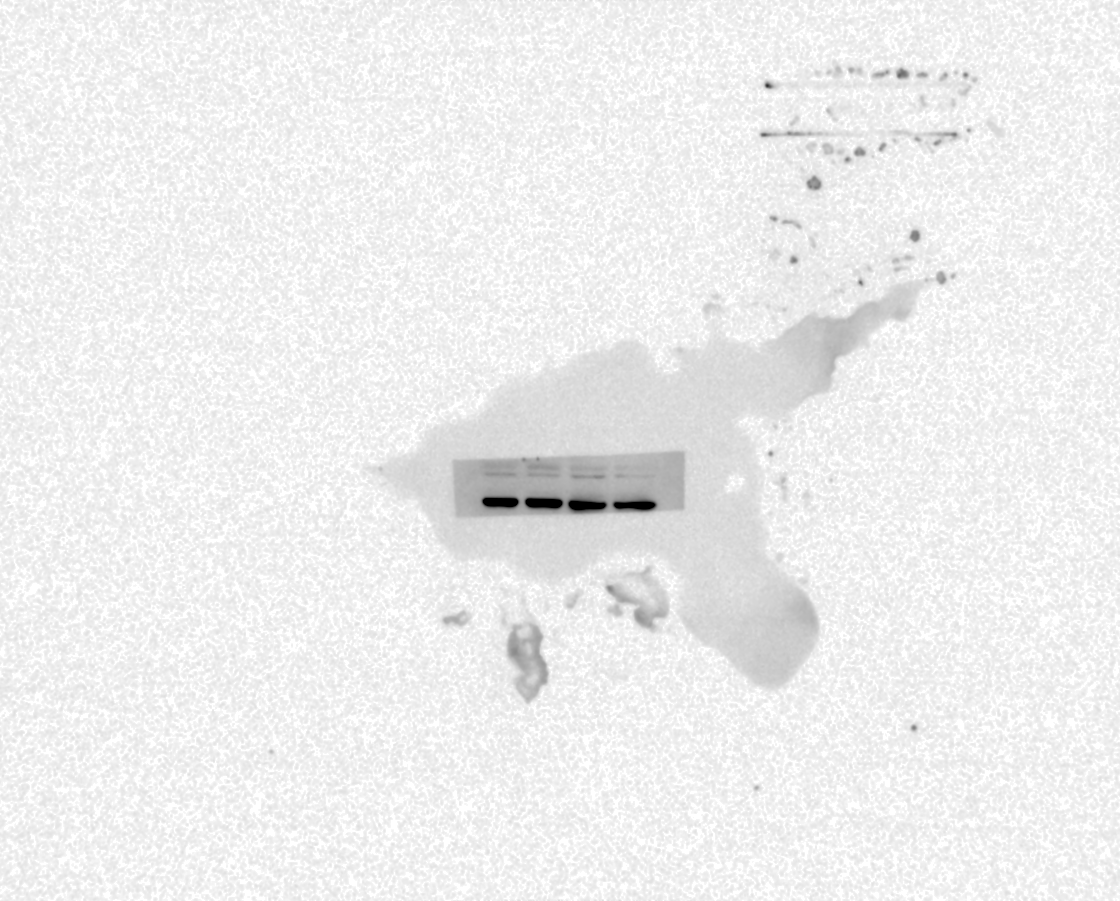

Supplement: Figure 3—source data 1. [file elife-81639-fig3-data1.zip › Figure3-source data/Figure 3N initial trial/WCE-anti╬▓ actin.tif]

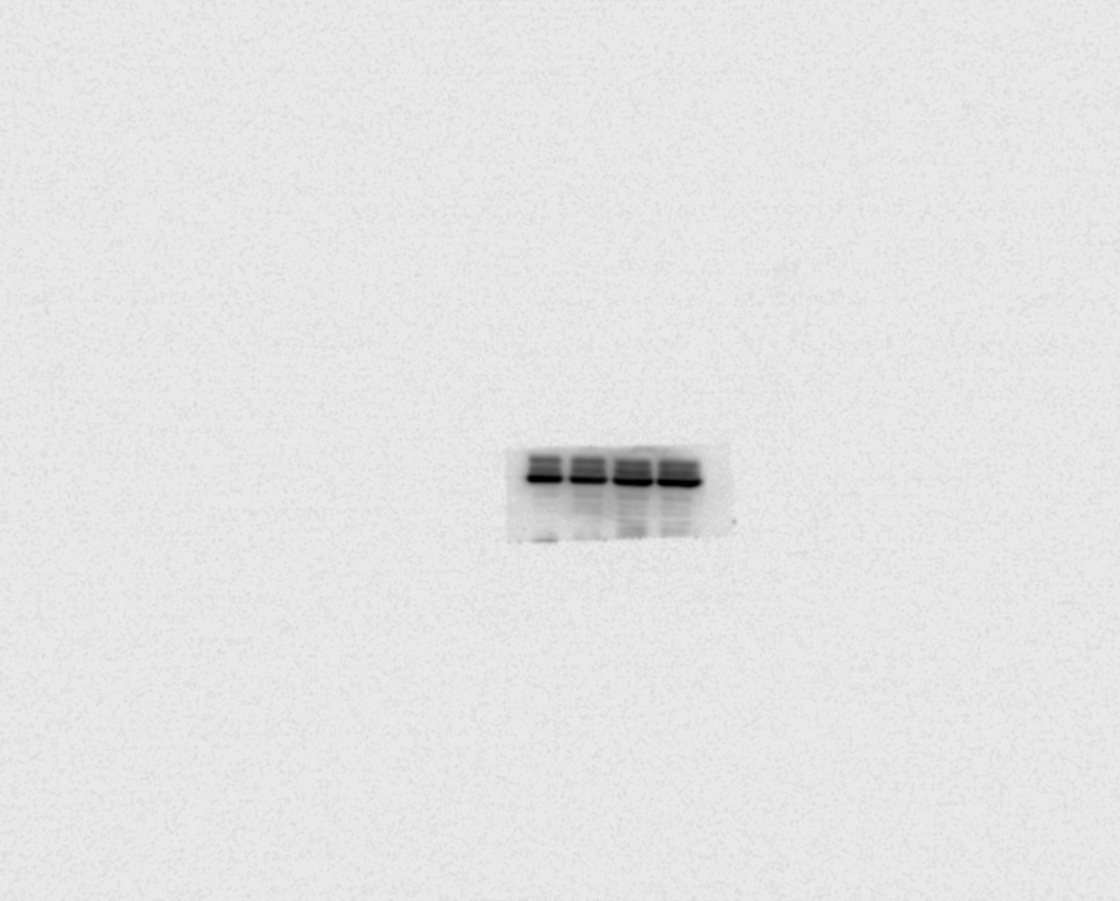

Supplement: Figure 3—source data 1. [file elife-81639-fig3-data1.zip › Figure3-source data/Figure 3N initial trial/WCE-antiRPA32.tif]

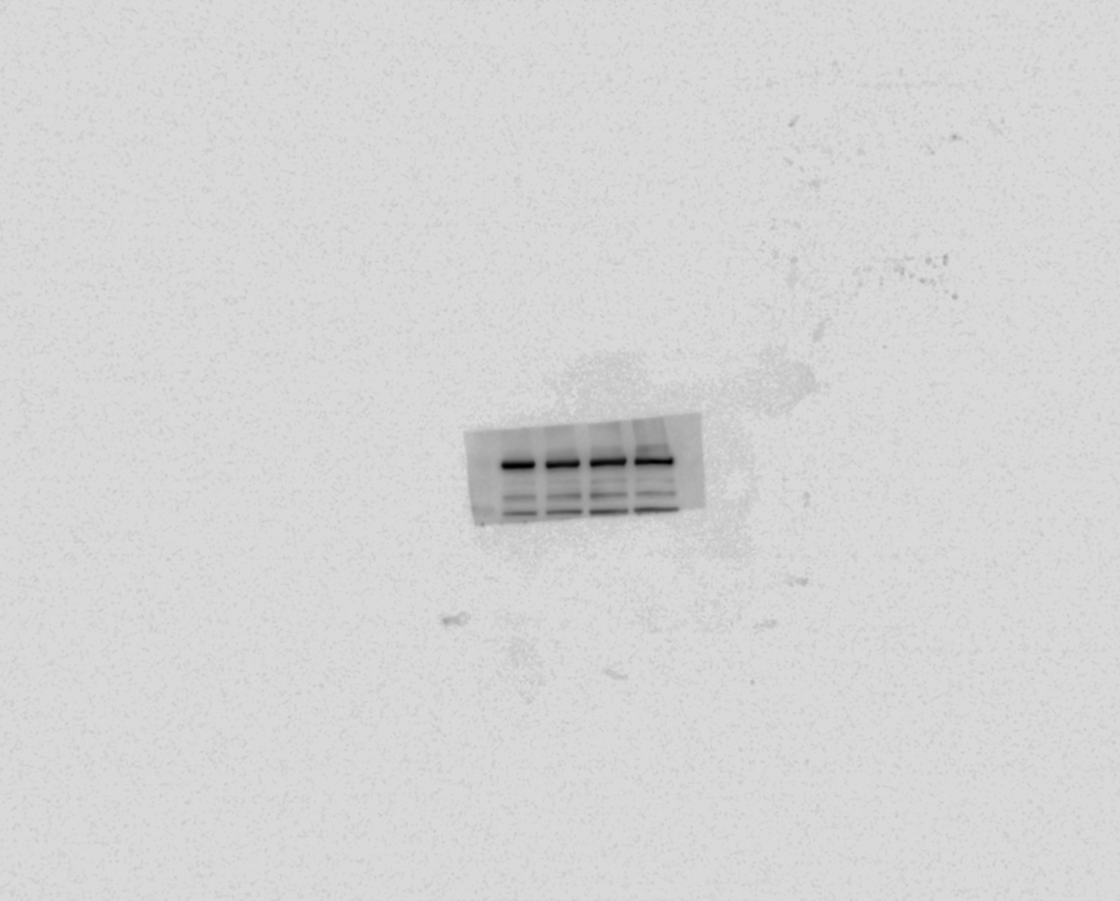

Supplement: Figure 3—source data 1. [file elife-81639-fig3-data1.zip › Figure3-source data/Figure 3N initial trial/WCE-antiGFP.tif]

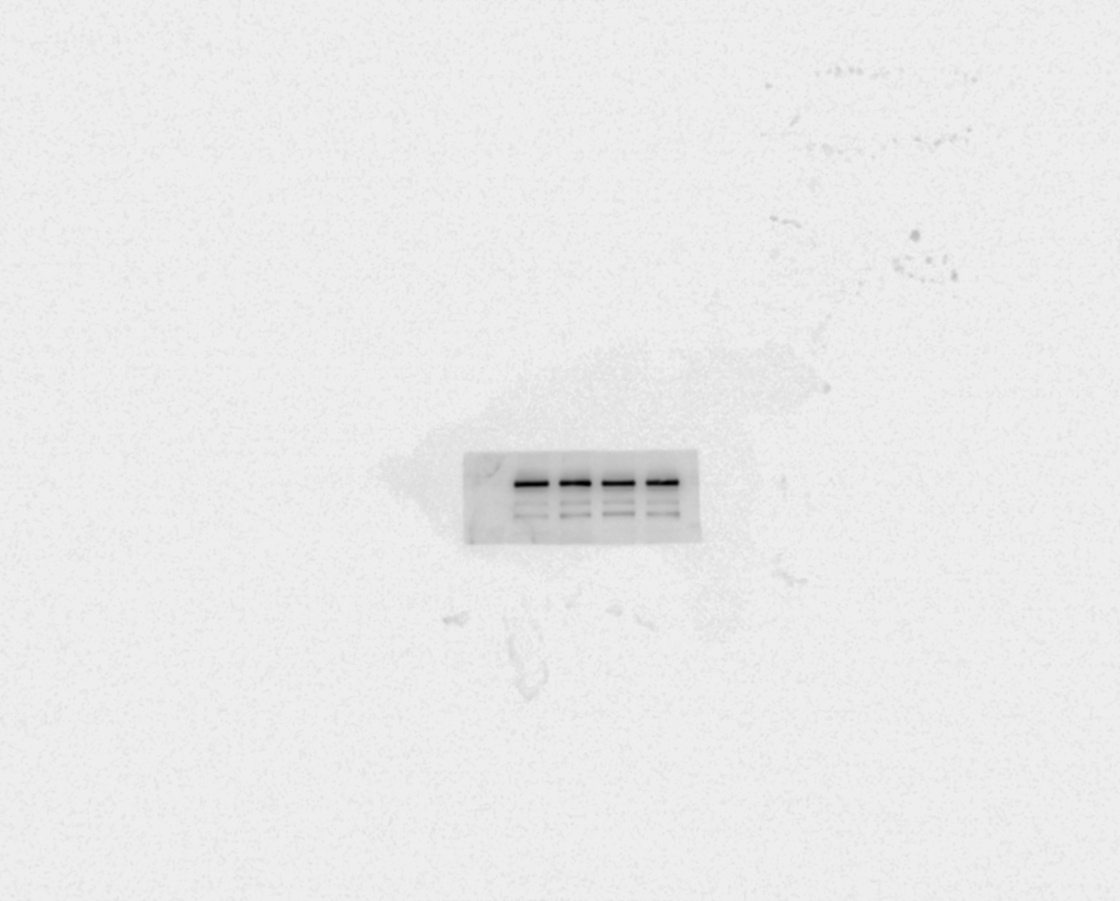

Supplement: Figure 3—source data 1. [file elife-81639-fig3-data1.zip › Figure3-source data/Figure 3N initial trial/IP-antiGFP.tif]

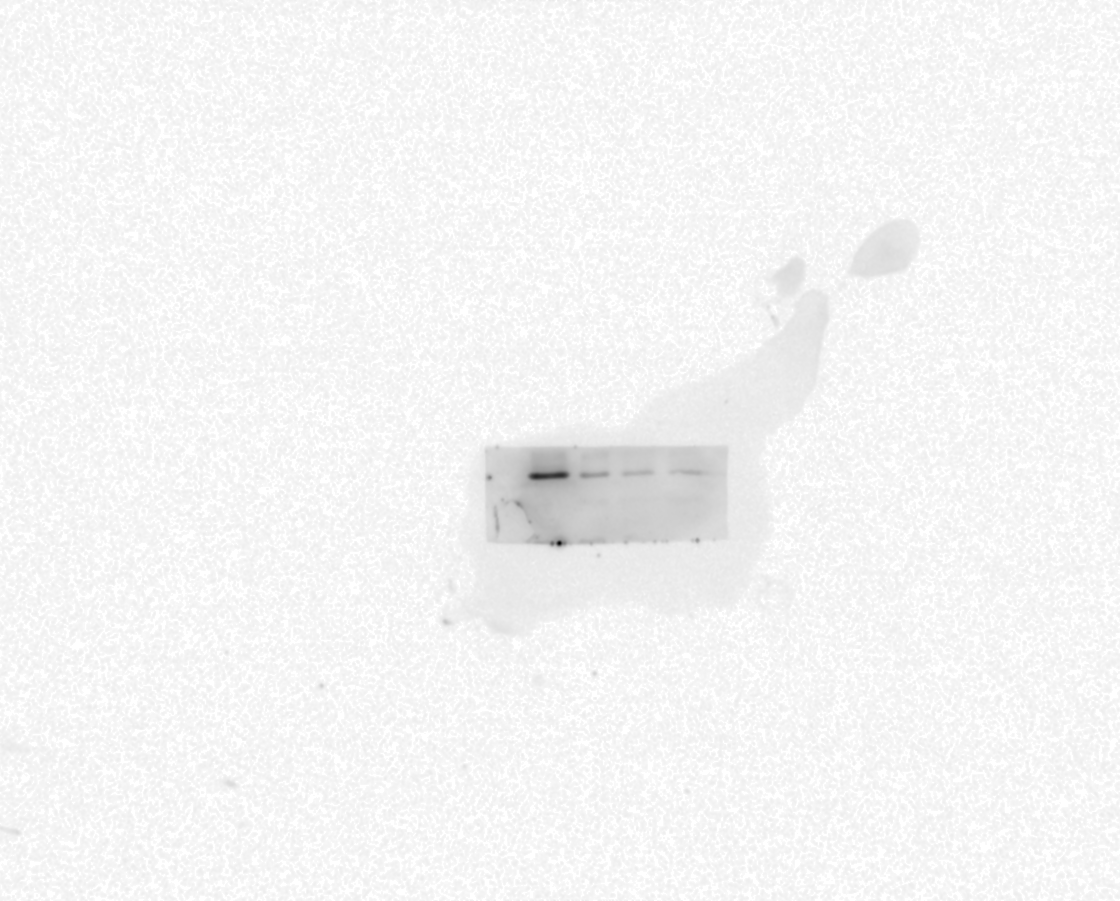

Supplement: Figure 3—source data 1. [file elife-81639-fig3-data1.zip › Figure3-source data/Figure 3N initial trial/IP-antiRPA32.tif]

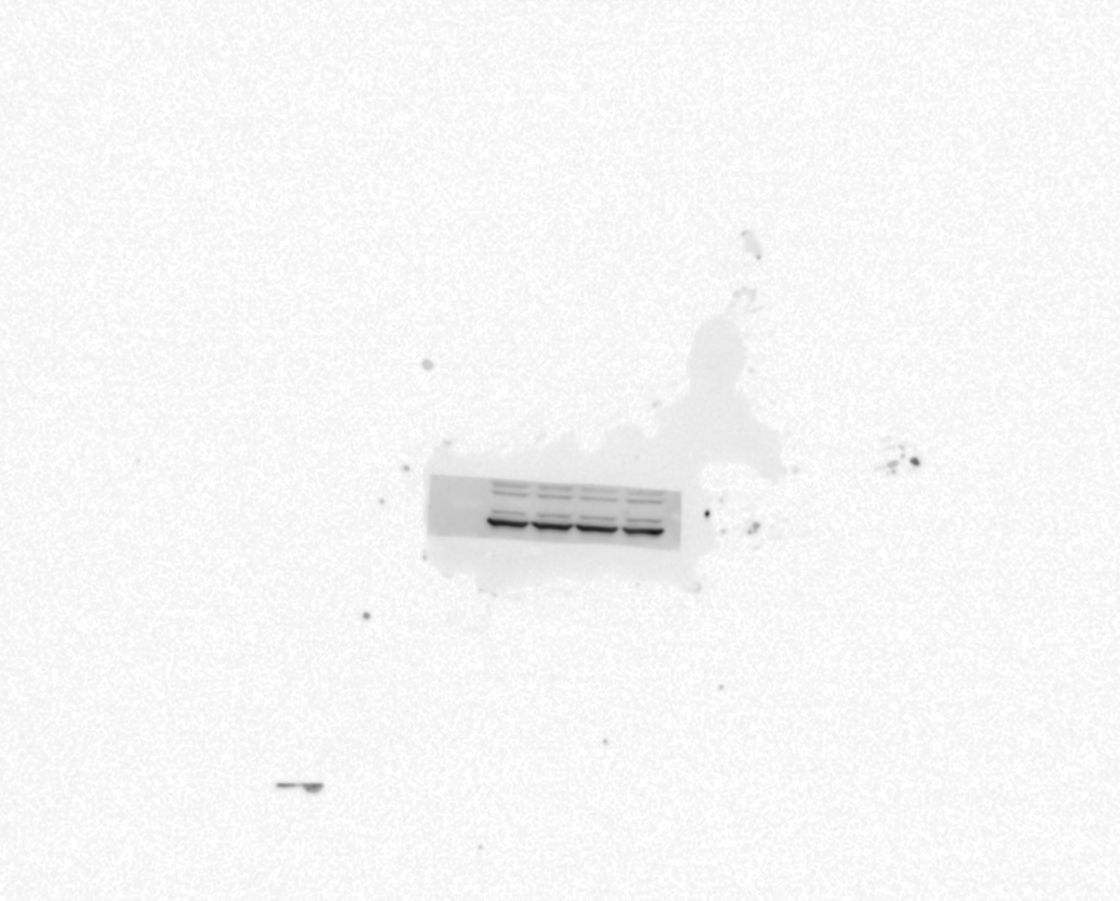

Supplement: Figure 3—source data 1. [file elife-81639-fig3-data1.zip › Figure3-source data/Figure 3N Repeat2/WCE-anti╬▓ actin.tif]

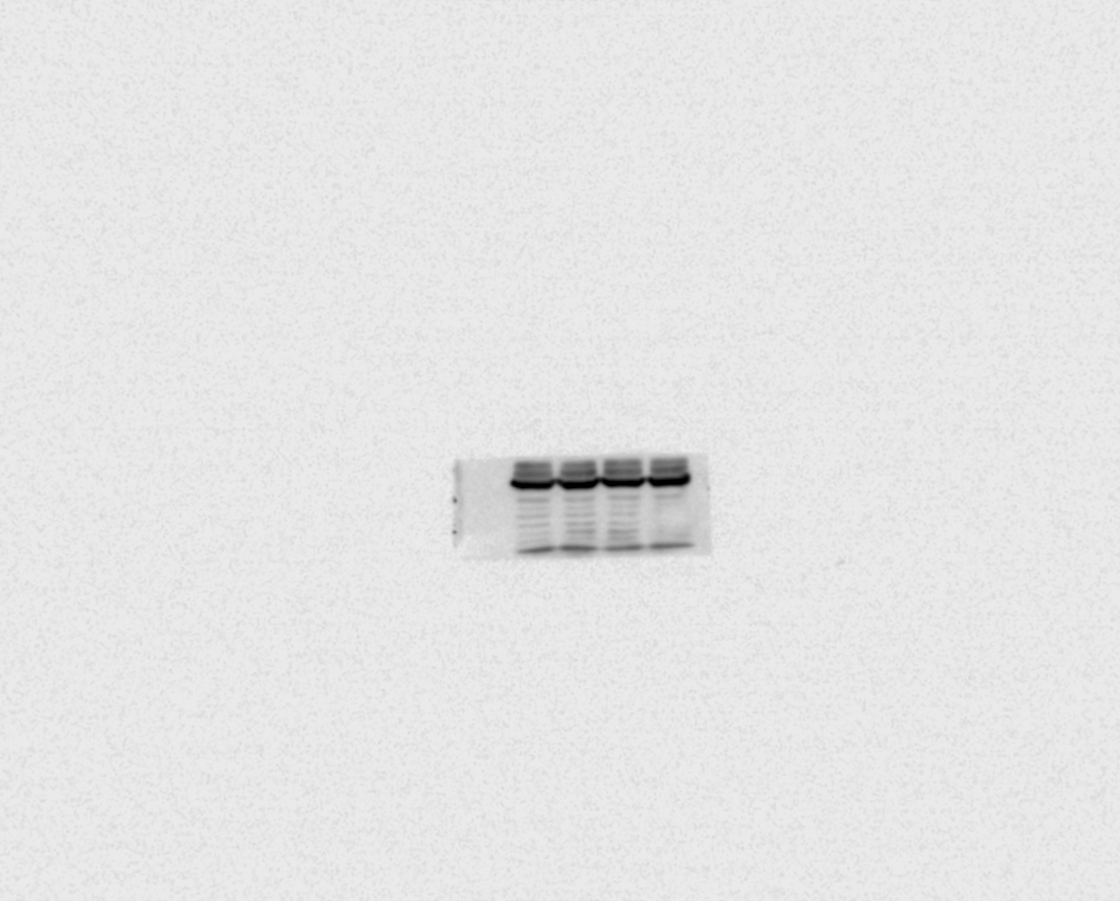

Supplement: Figure 3—source data 1. [file elife-81639-fig3-data1.zip › Figure3-source data/Figure 3N Repeat2/WCE-antiRPA32.tif]

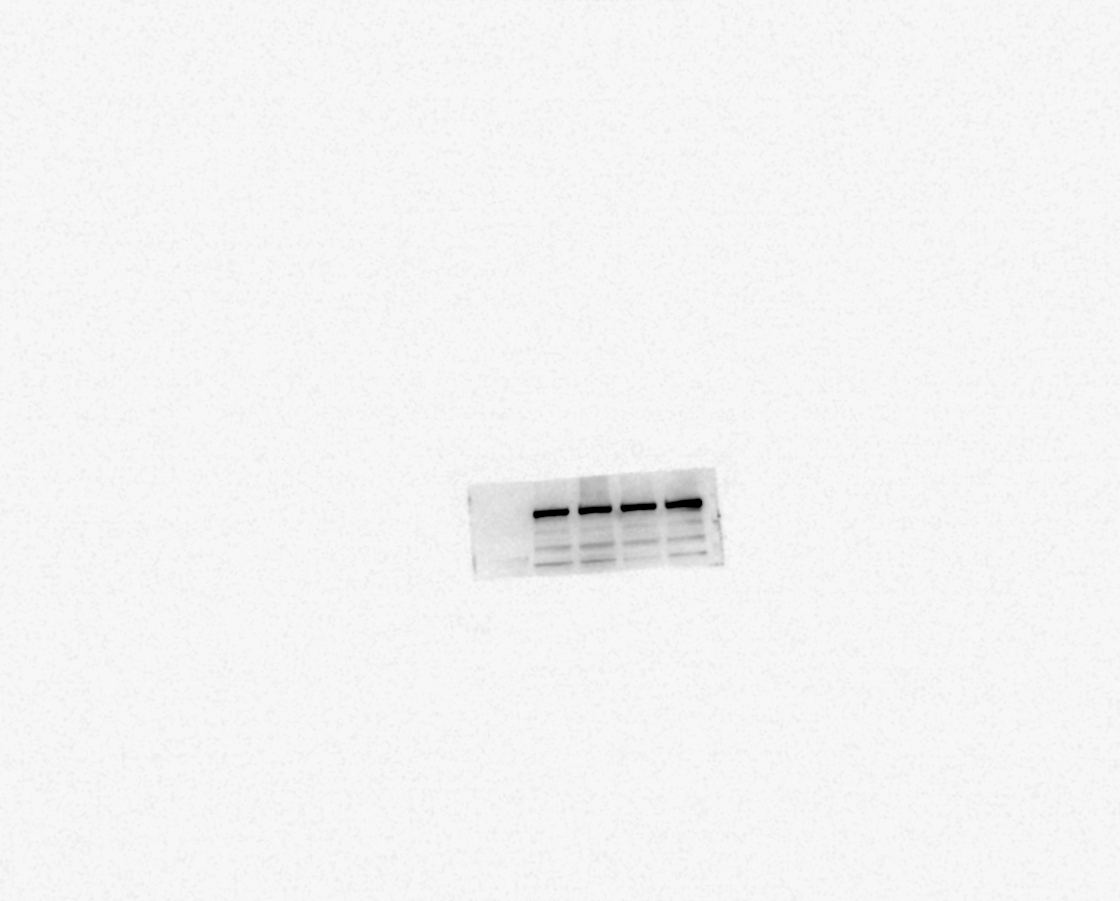

Supplement: Figure 3—source data 1. [file elife-81639-fig3-data1.zip › Figure3-source data/Figure 3N Repeat2/WCE-antiGFP.tif]

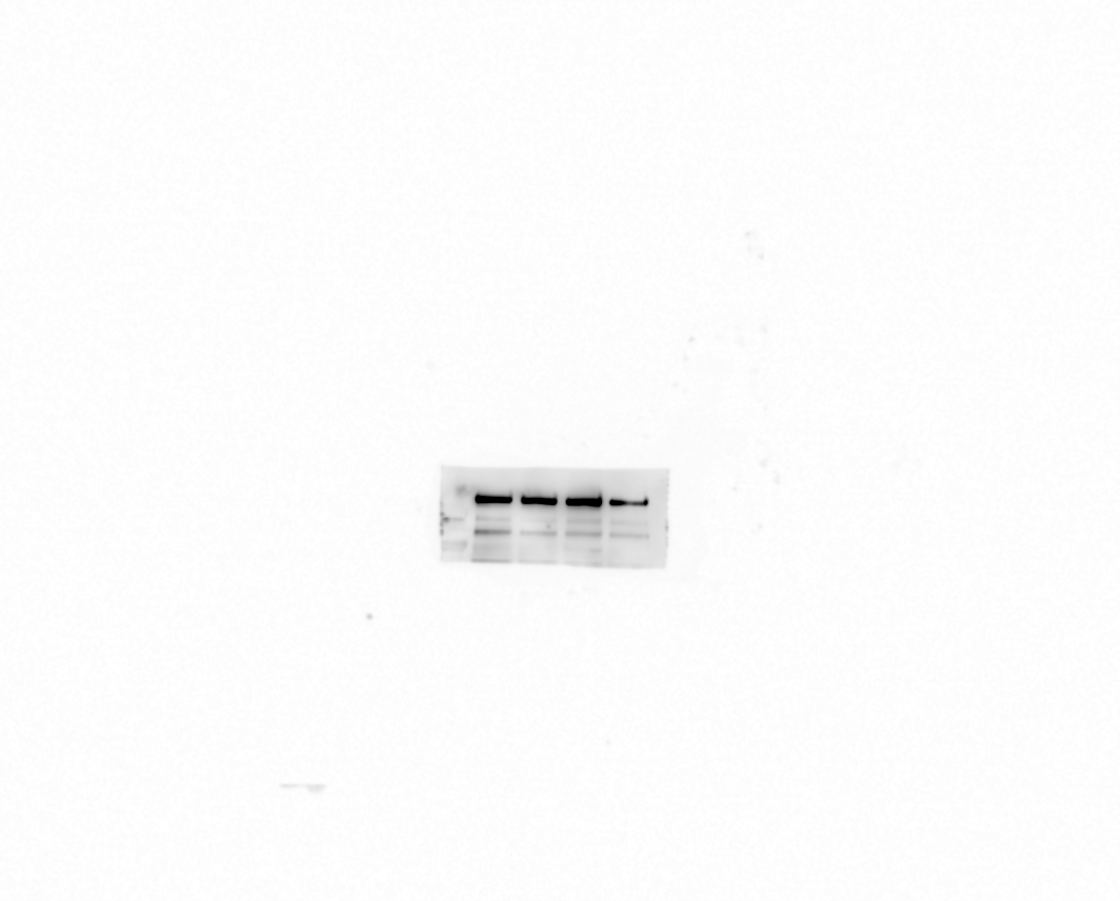

Supplement: Figure 3—source data 1. [file elife-81639-fig3-data1.zip › Figure3-source data/Figure 3N Repeat2/IP-antiGFP.tif]

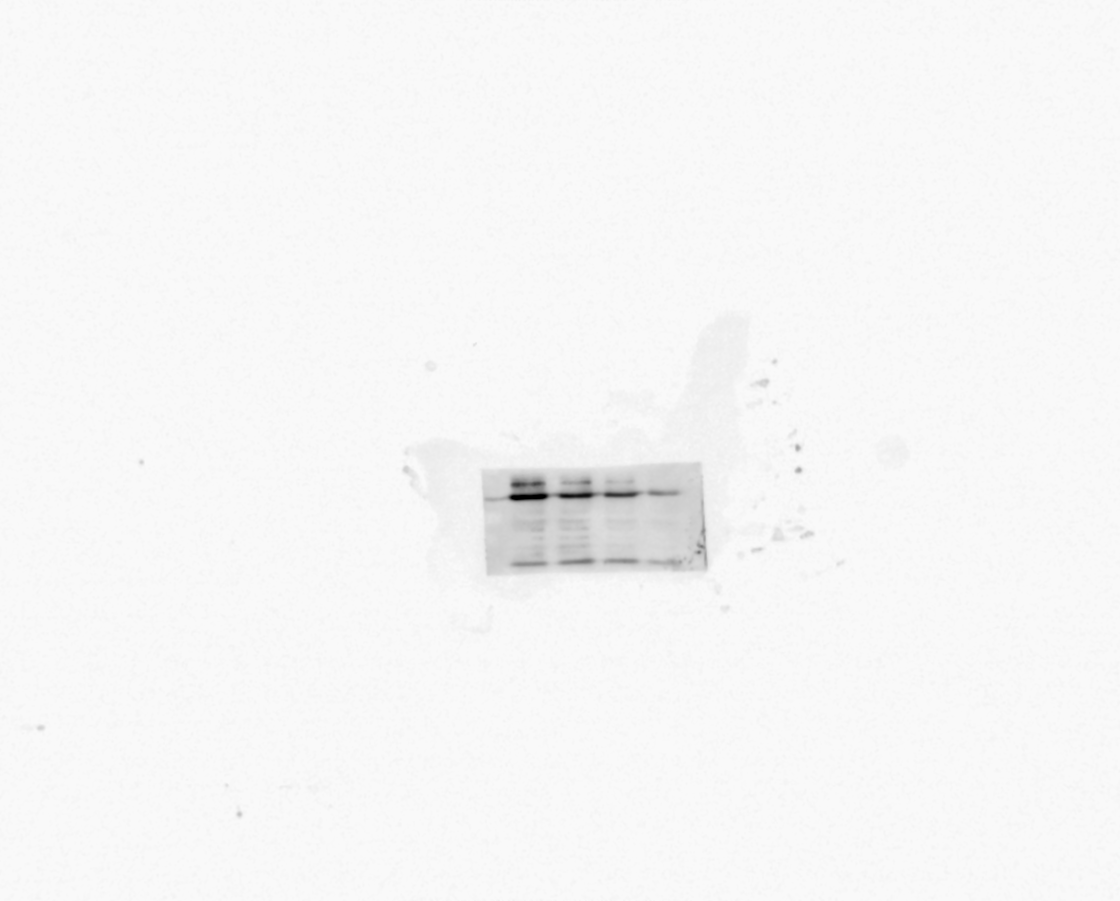

Supplement: Figure 3—source data 1. [file elife-81639-fig3-data1.zip › Figure3-source data/Figure 3N Repeat2/IP-antiRPA32.tif]

# G

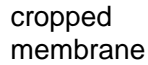

Supplement: Figure 4—source data 1. [file elife-81639-fig4-data1.zip › Figure4-source data/Figure 4G.pdf]

Figure 4G

RMI

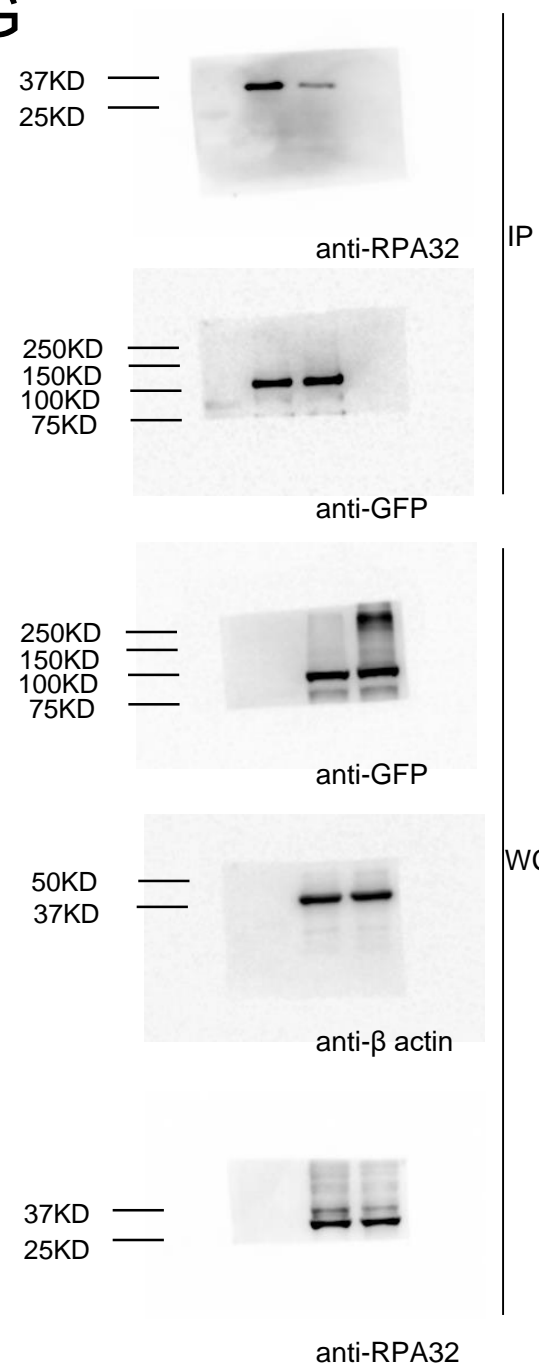

Five parts of  
the same gel

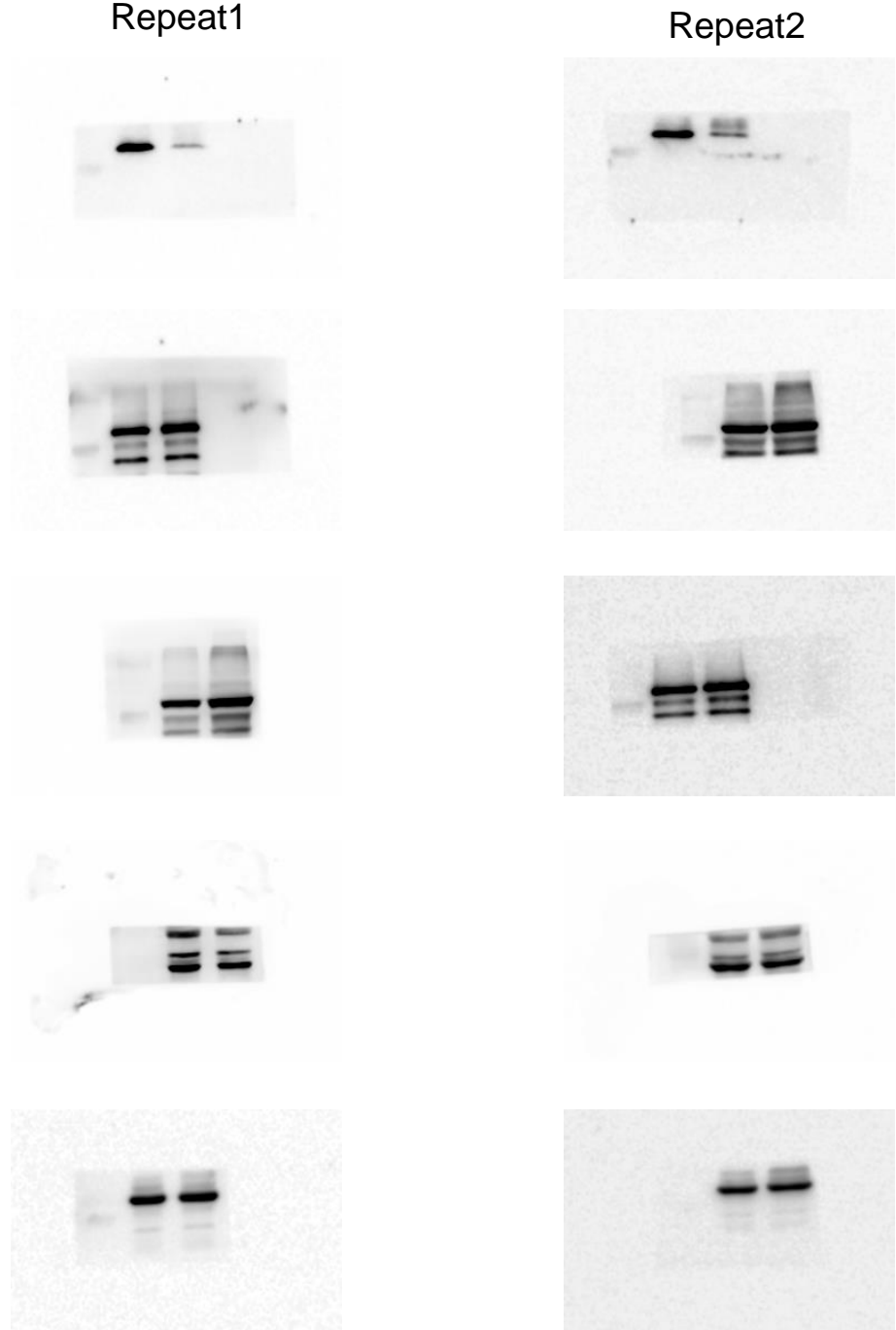

Supplement: Figure 4—source data 1. [file elife-81639-fig4-data1.zip › Figure4-source data/IP-data-Figure 4G.pdf]

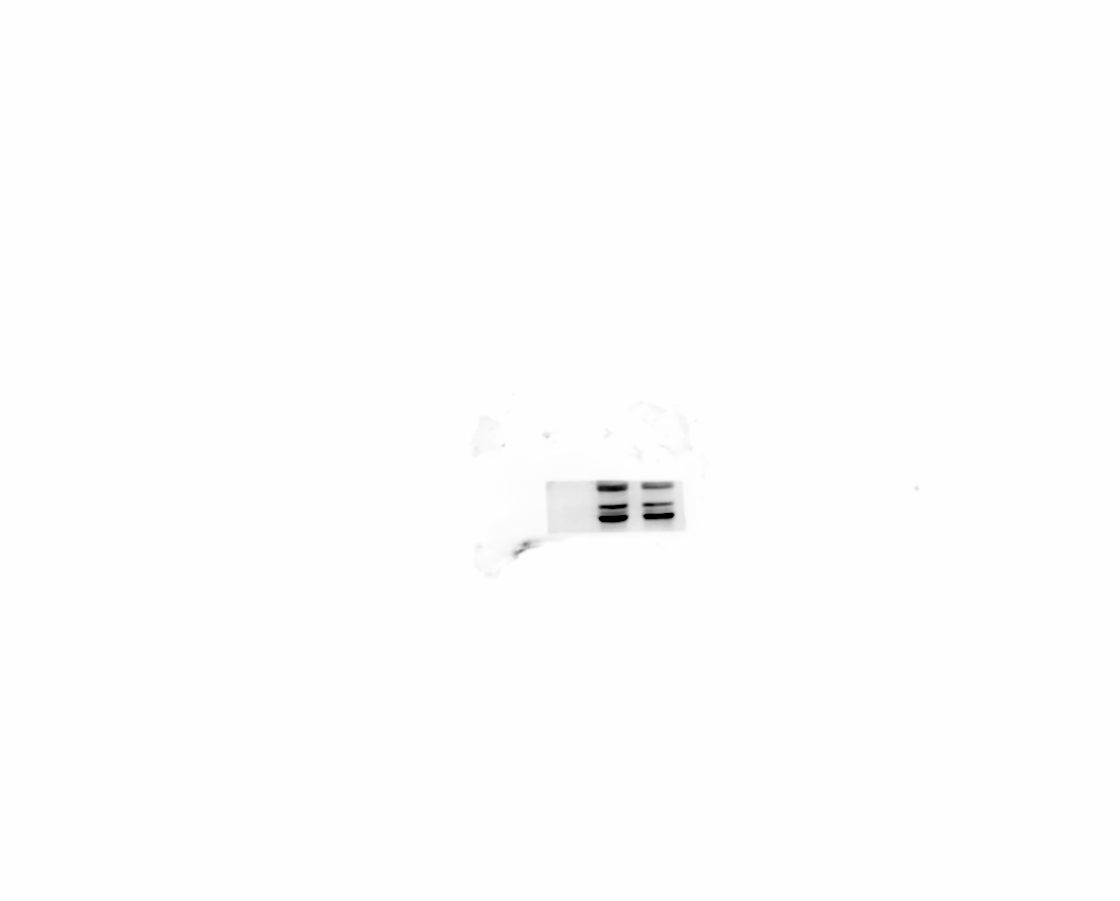

Supplement: Figure 4—source data 1. [file elife-81639-fig4-data1.zip › Figure4-source data/Figure 4G Repeat1/WCE-anti╬▓ actin.tif]

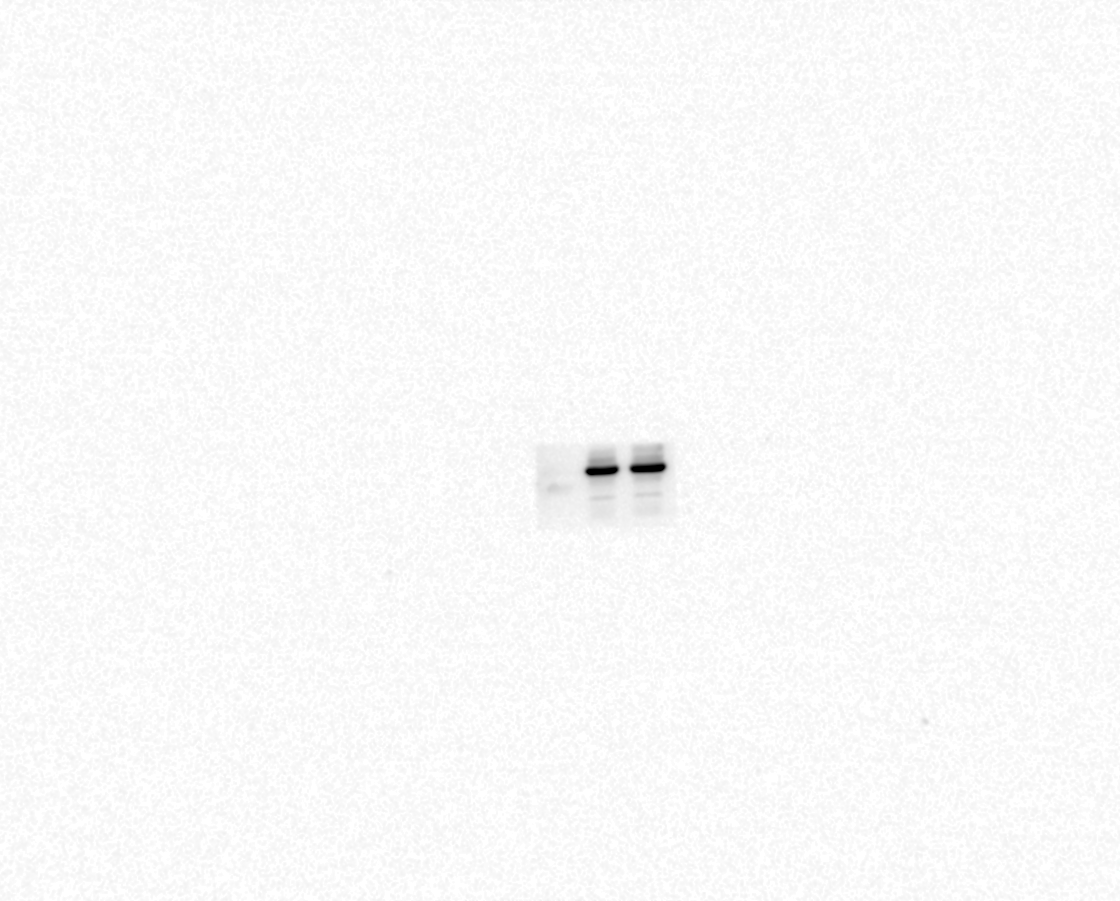

Supplement: Figure 4—source data 1. [file elife-81639-fig4-data1.zip › Figure4-source data/Figure 4G Repeat1/WCE-antiRPA32.tif]

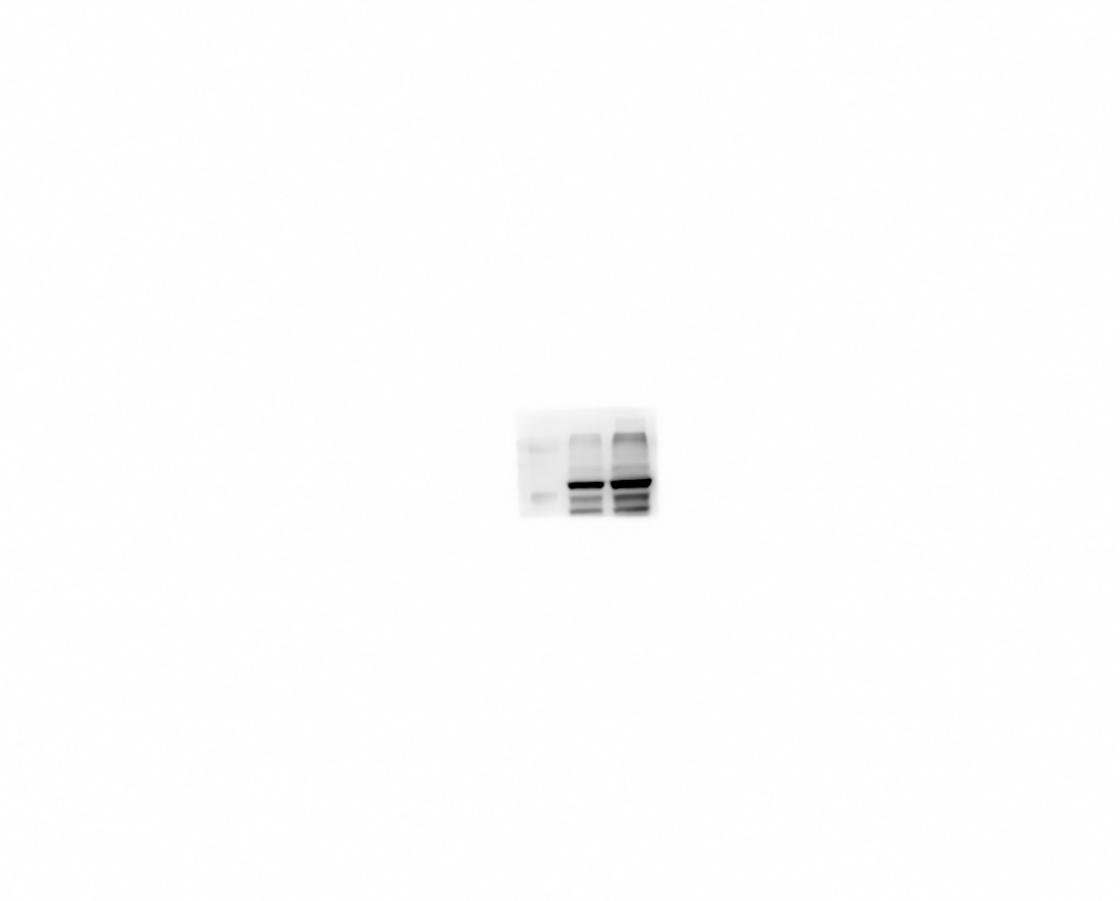

Supplement: Figure 4—source data 1. [file elife-81639-fig4-data1.zip › Figure4-source data/Figure 4G Repeat1/WCE-antiGFP.tif]

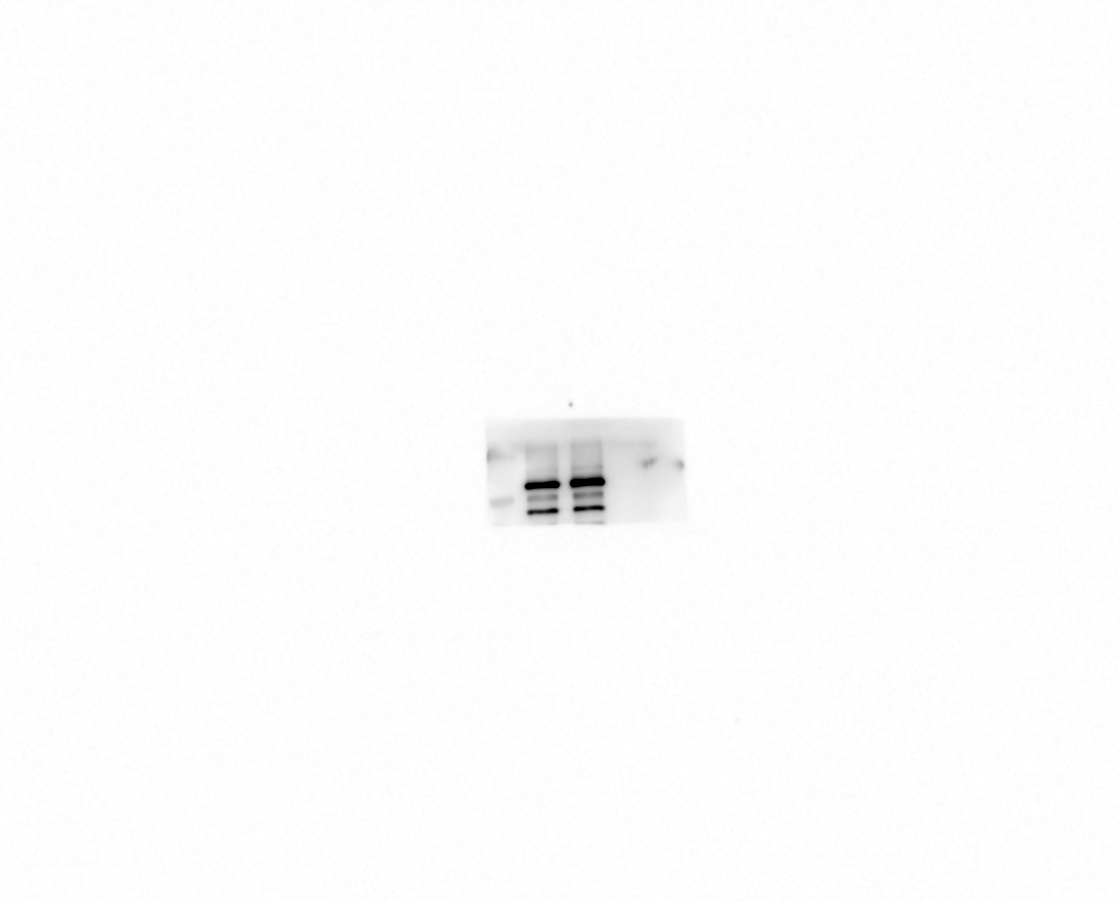

Supplement: Figure 4—source data 1. [file elife-81639-fig4-data1.zip › Figure4-source data/Figure 4G Repeat1/IP-antiGFP.tif]

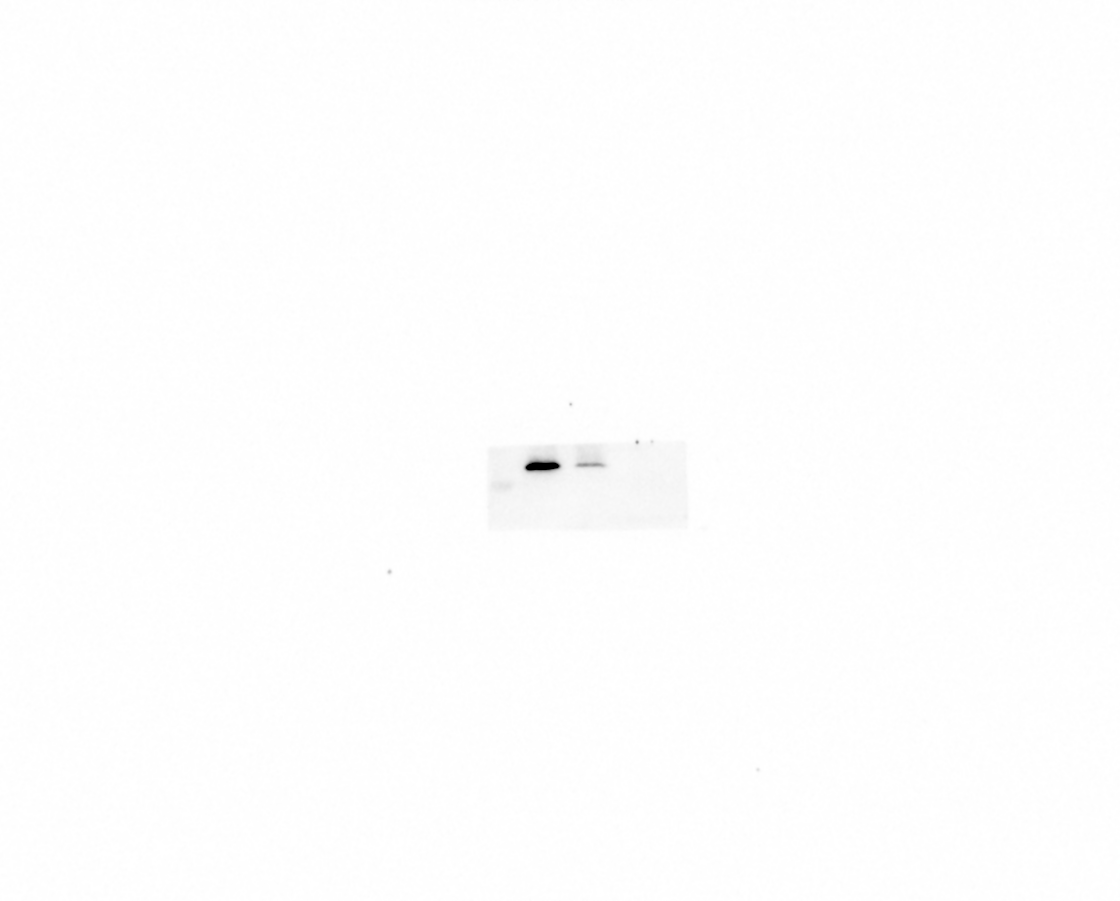

Supplement: Figure 4—source data 1. [file elife-81639-fig4-data1.zip › Figure4-source data/Figure 4G Repeat1/IP-antiRPA32.tif]

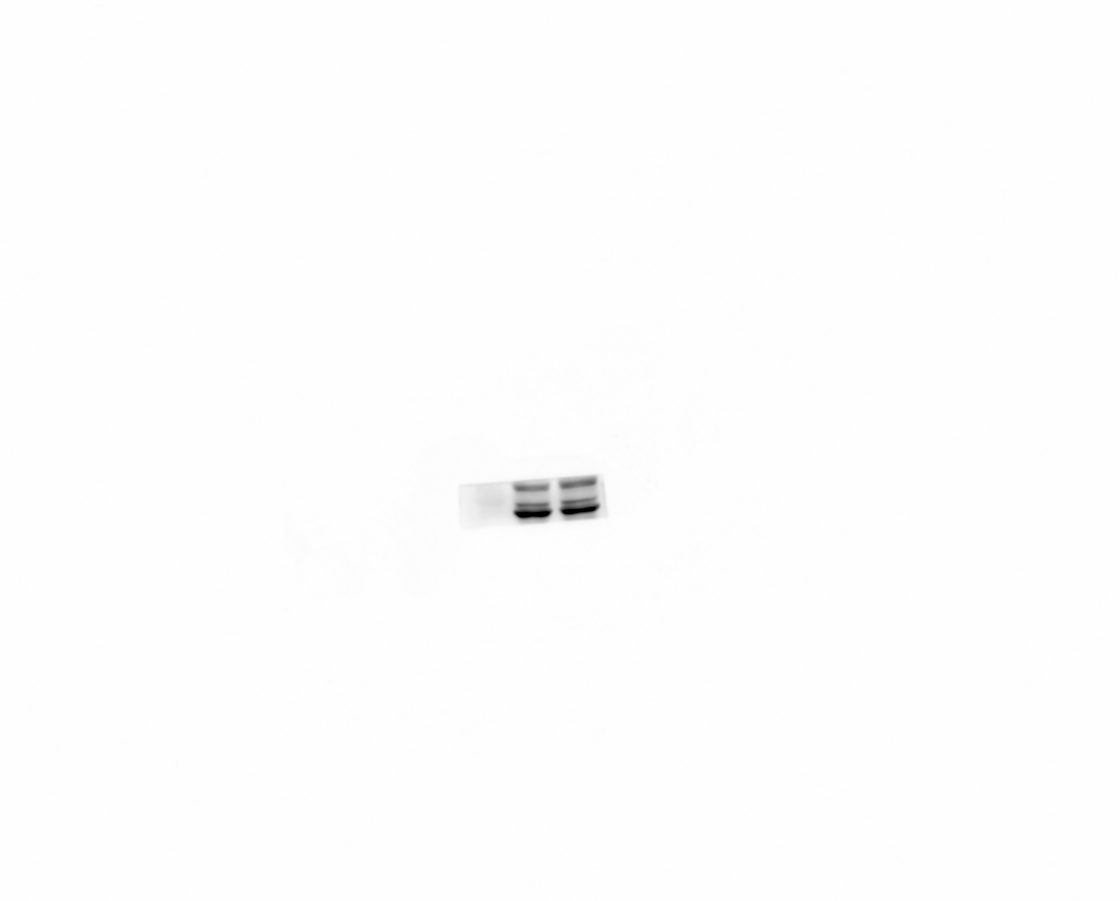

Supplement: Figure 4—source data 1. [file elife-81639-fig4-data1.zip › Figure4-source data/Figure 4G Repeat2/WCE-anti╬▓ actin.tif]

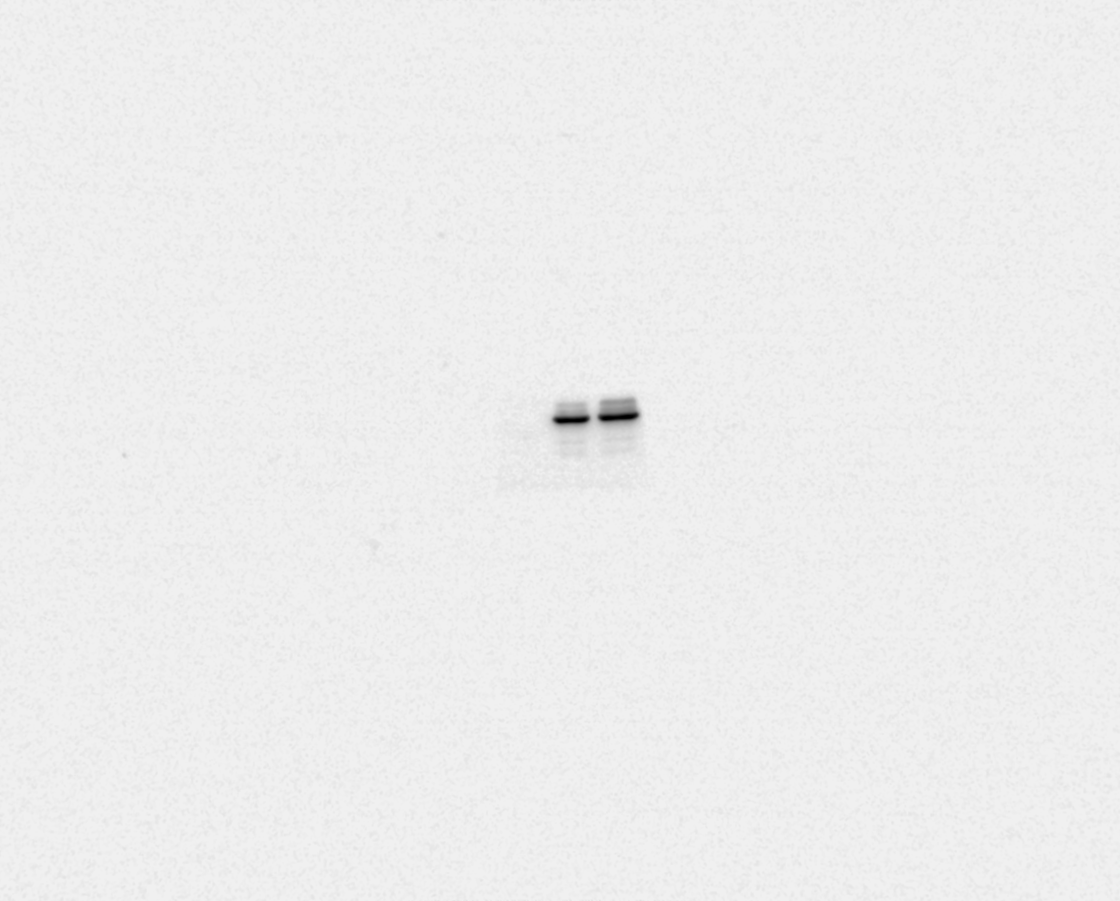

Supplement: Figure 4—source data 1. [file elife-81639-fig4-data1.zip › Figure4-source data/Figure 4G Repeat2/WCE-antiRPA32.tif]

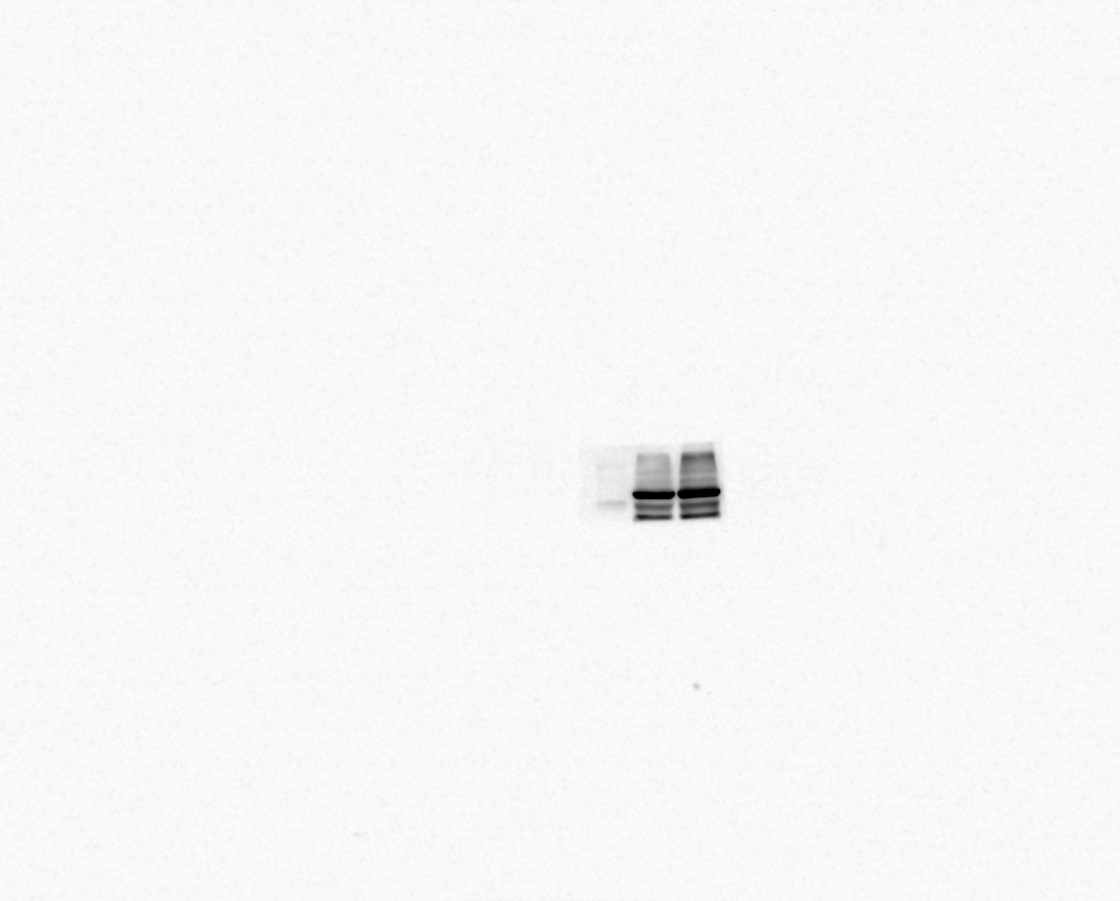

Supplement: Figure 4—source data 1. [file elife-81639-fig4-data1.zip › Figure4-source data/Figure 4G Repeat2/WCE-antiGFP.tif]

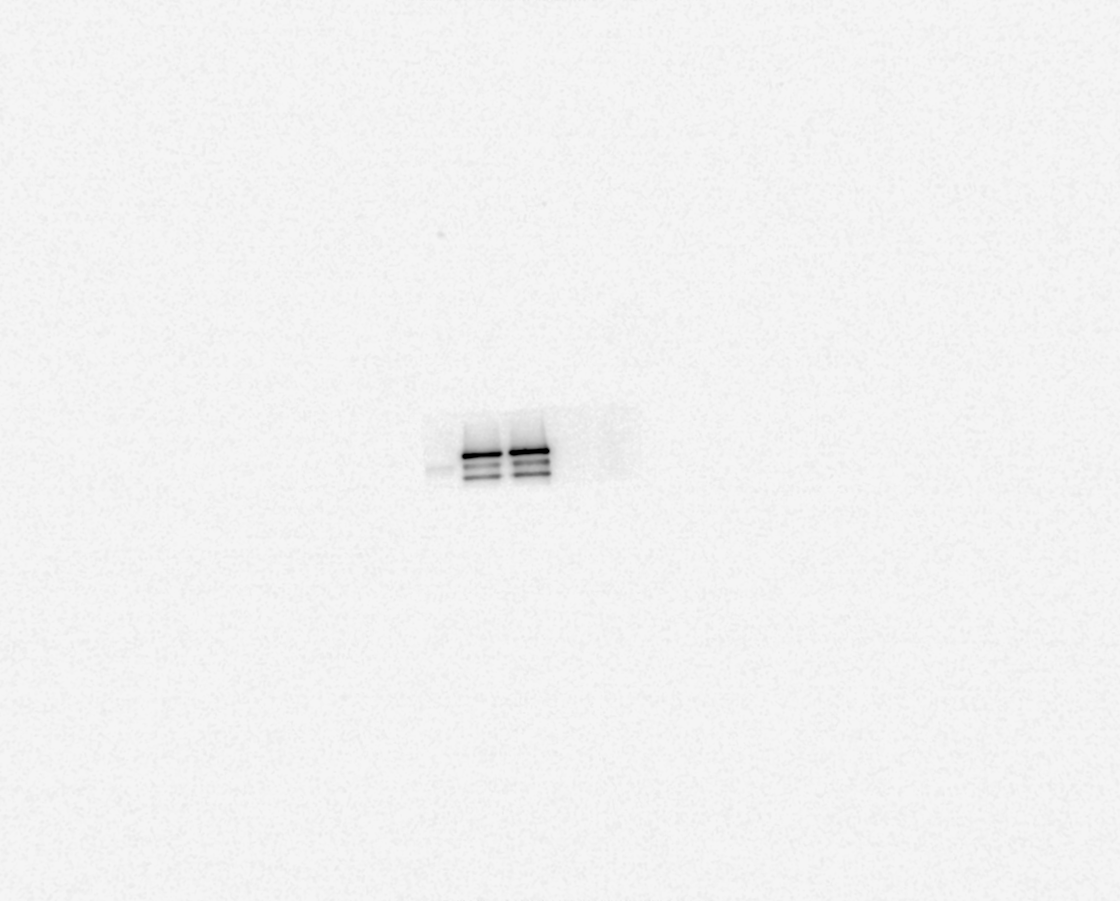

Supplement: Figure 4—source data 1. [file elife-81639-fig4-data1.zip › Figure4-source data/Figure 4G Repeat2/IP-antiGFP.tif]

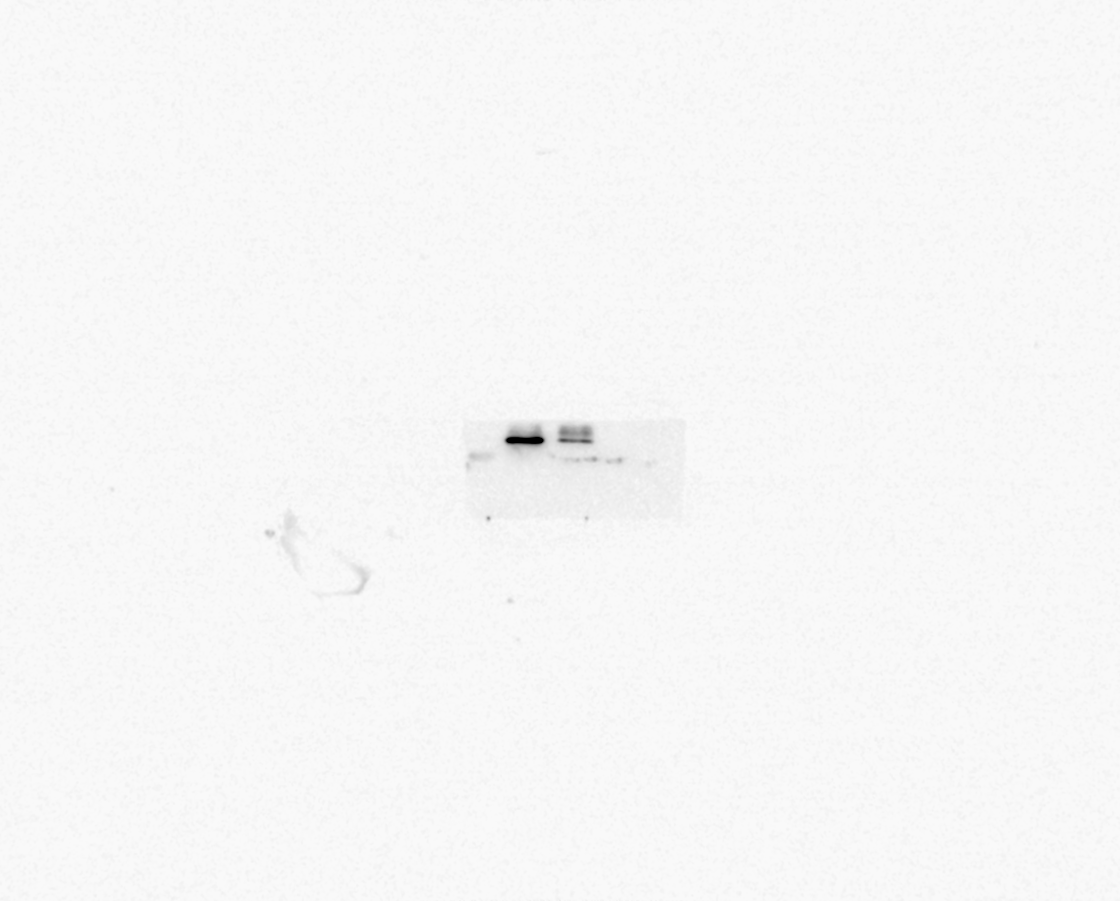

Supplement: Figure 4—source data 1. [file elife-81639-fig4-data1.zip › Figure4-source data/Figure 4G Repeat2/IP-antiRPA32.tif]

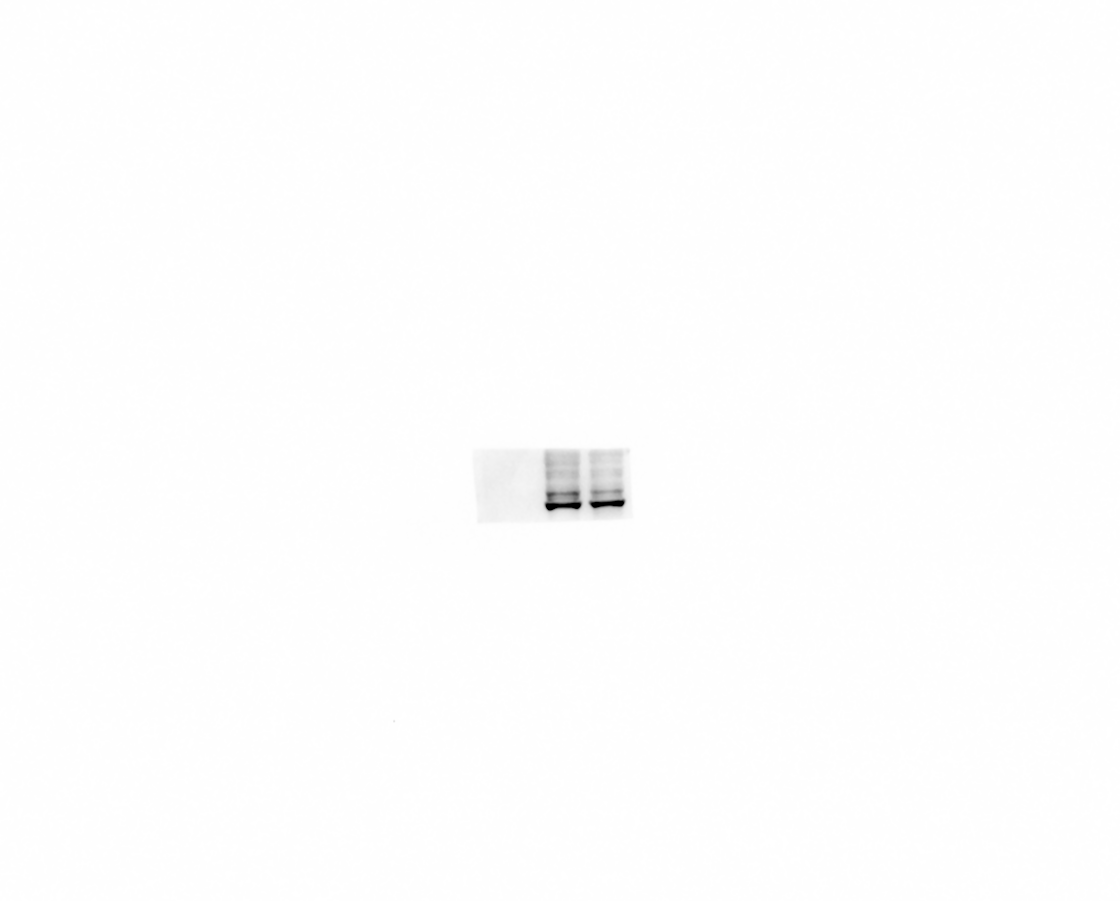

Supplement: Figure 4—source data 1. [file elife-81639-fig4-data1.zip › Figure4-source data/Figure 4G initial trial/WCE-anti╬▓ actin.tif]

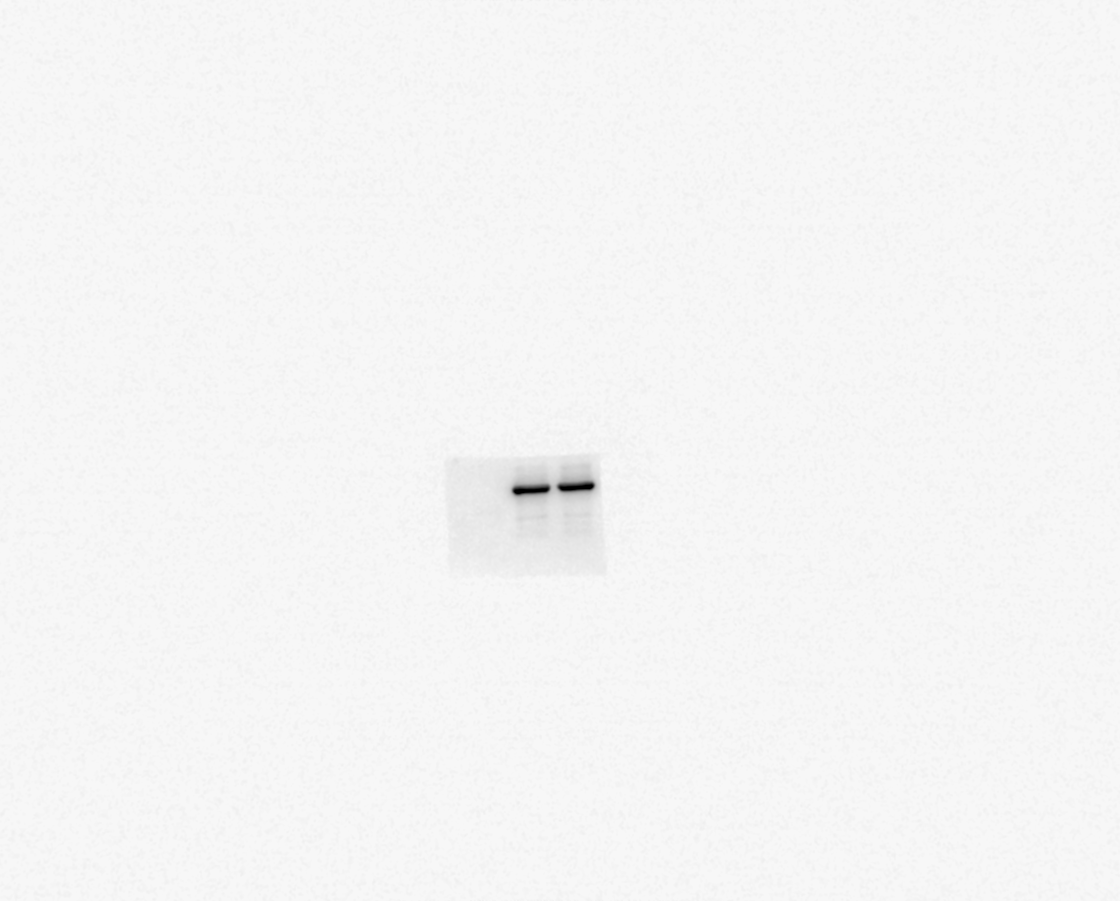

Supplement: Figure 4—source data 1. [file elife-81639-fig4-data1.zip › Figure4-source data/Figure 4G initial trial/WCE-antiRPA32.tif]

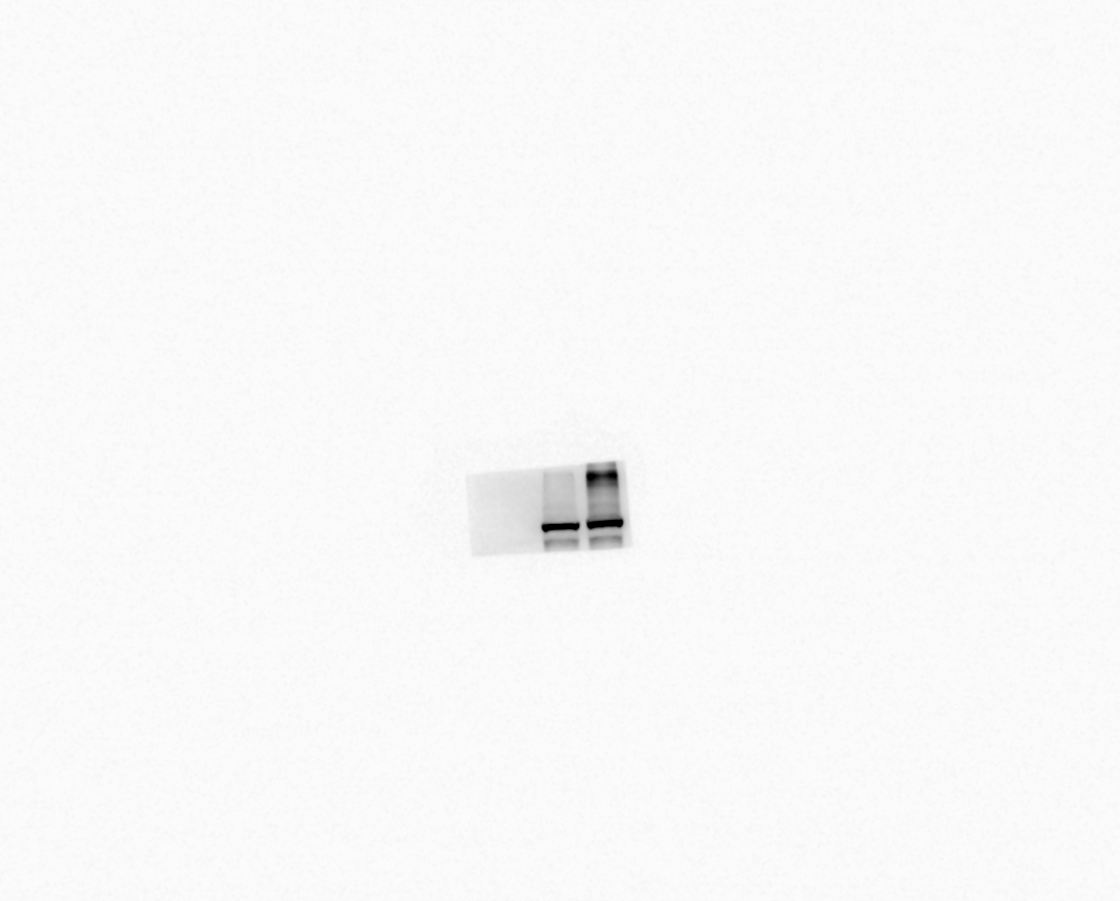

Supplement: Figure 4—source data 1. [file elife-81639-fig4-data1.zip › Figure4-source data/Figure 4G initial trial/WCE-antiGFP.tif]

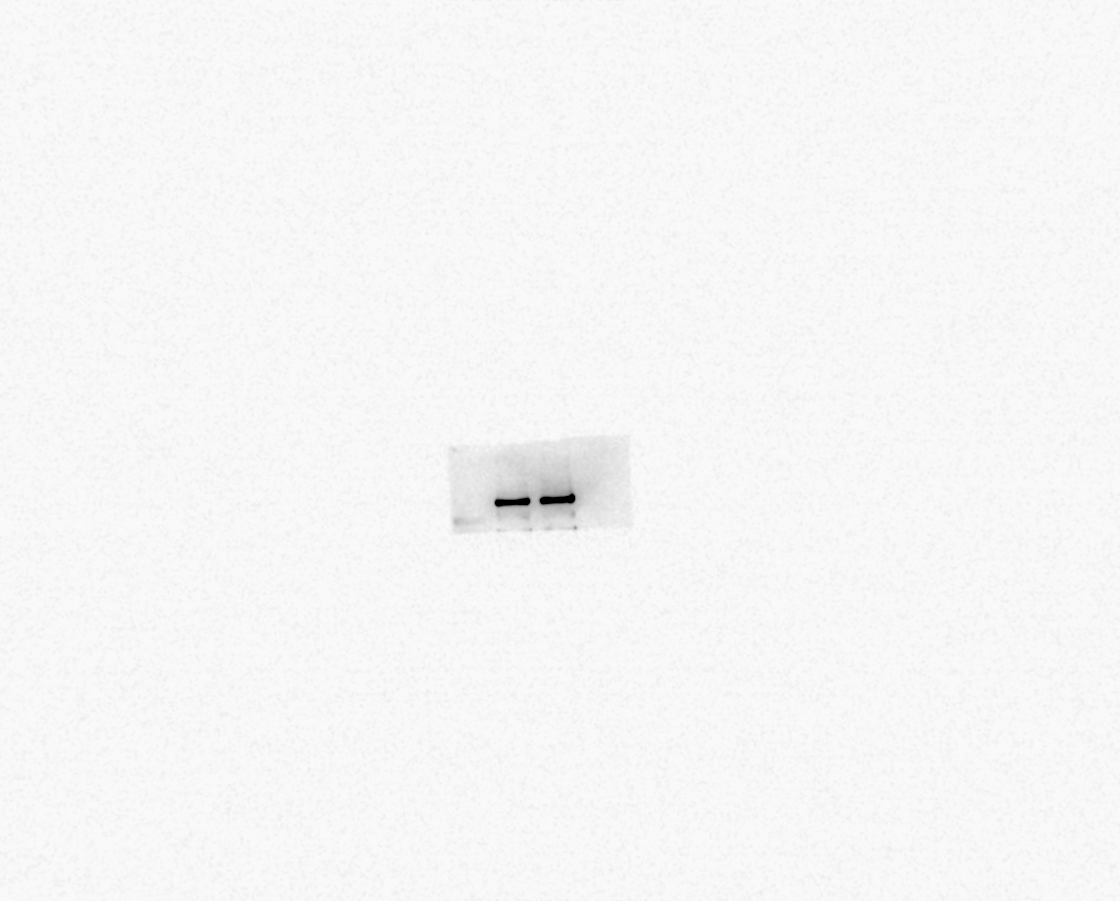

Supplement: Figure 4—source data 1. [file elife-81639-fig4-data1.zip › Figure4-source data/Figure 4G initial trial/IP-antiGFP.tif]

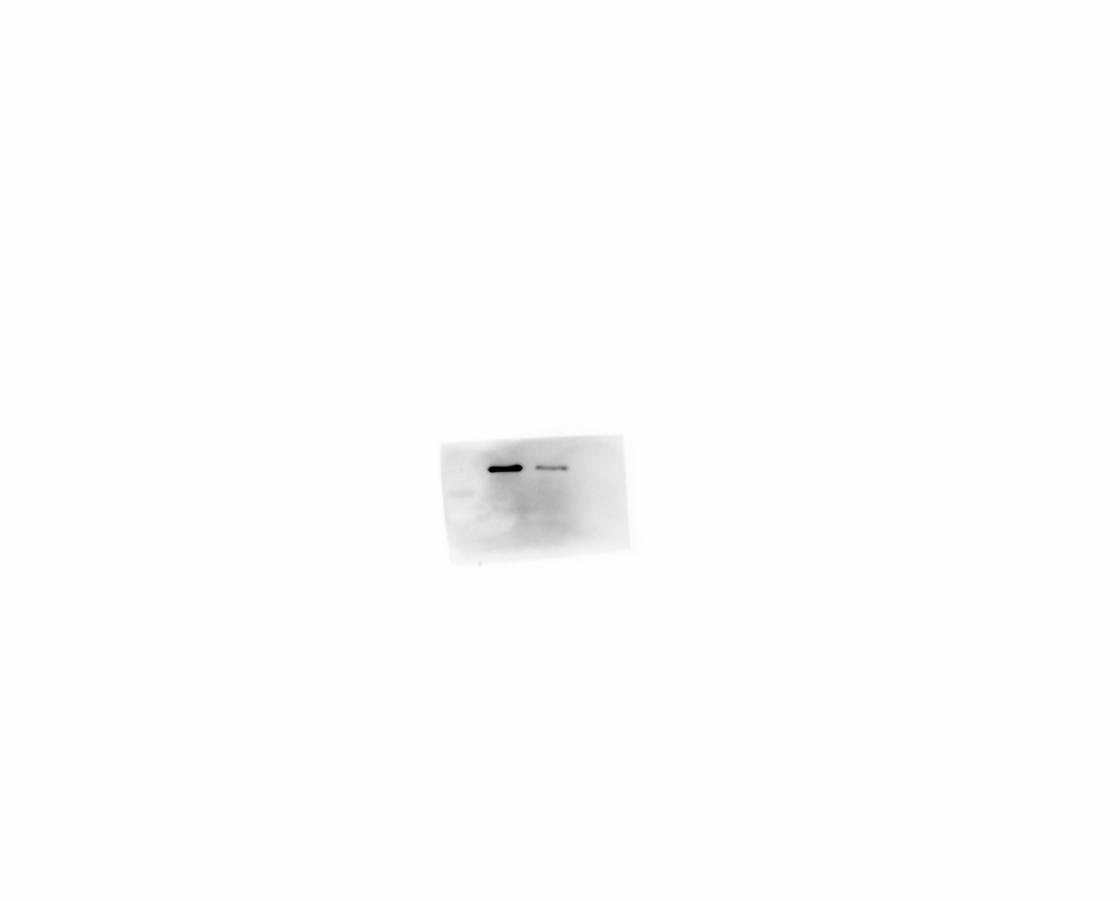

Supplement: Figure 4—source data 1. [file elife-81639-fig4-data1.zip › Figure4-source data/Figure 4G initial trial/IP-antiRPA32.tif]

Figure 5L  
WRN

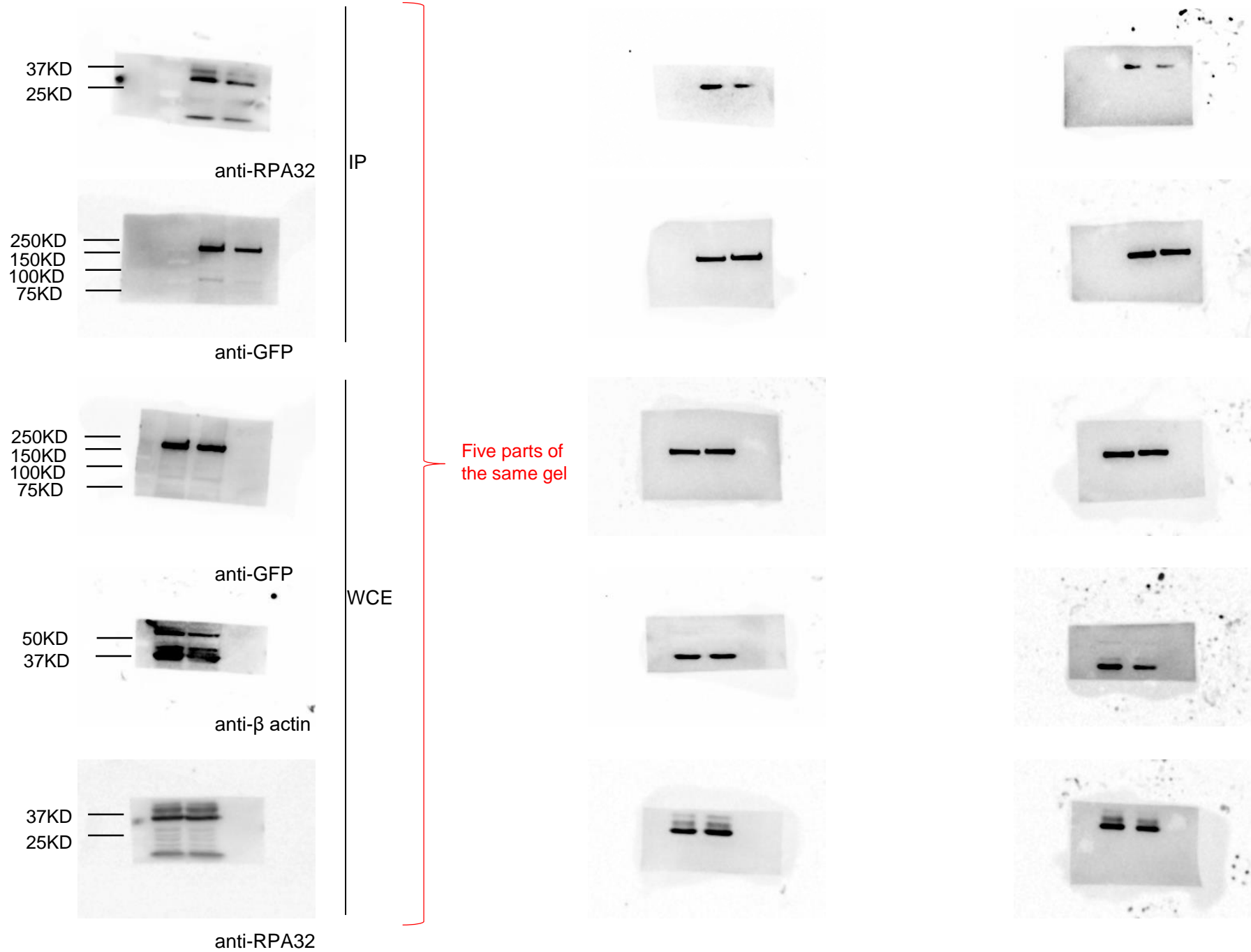

Supplement: Figure 5—source data 1. [file elife-81639-fig5-data1.zip › Figure5-source data/IP-data-Figure 5L.pdf]

Figure 5L  
WRN

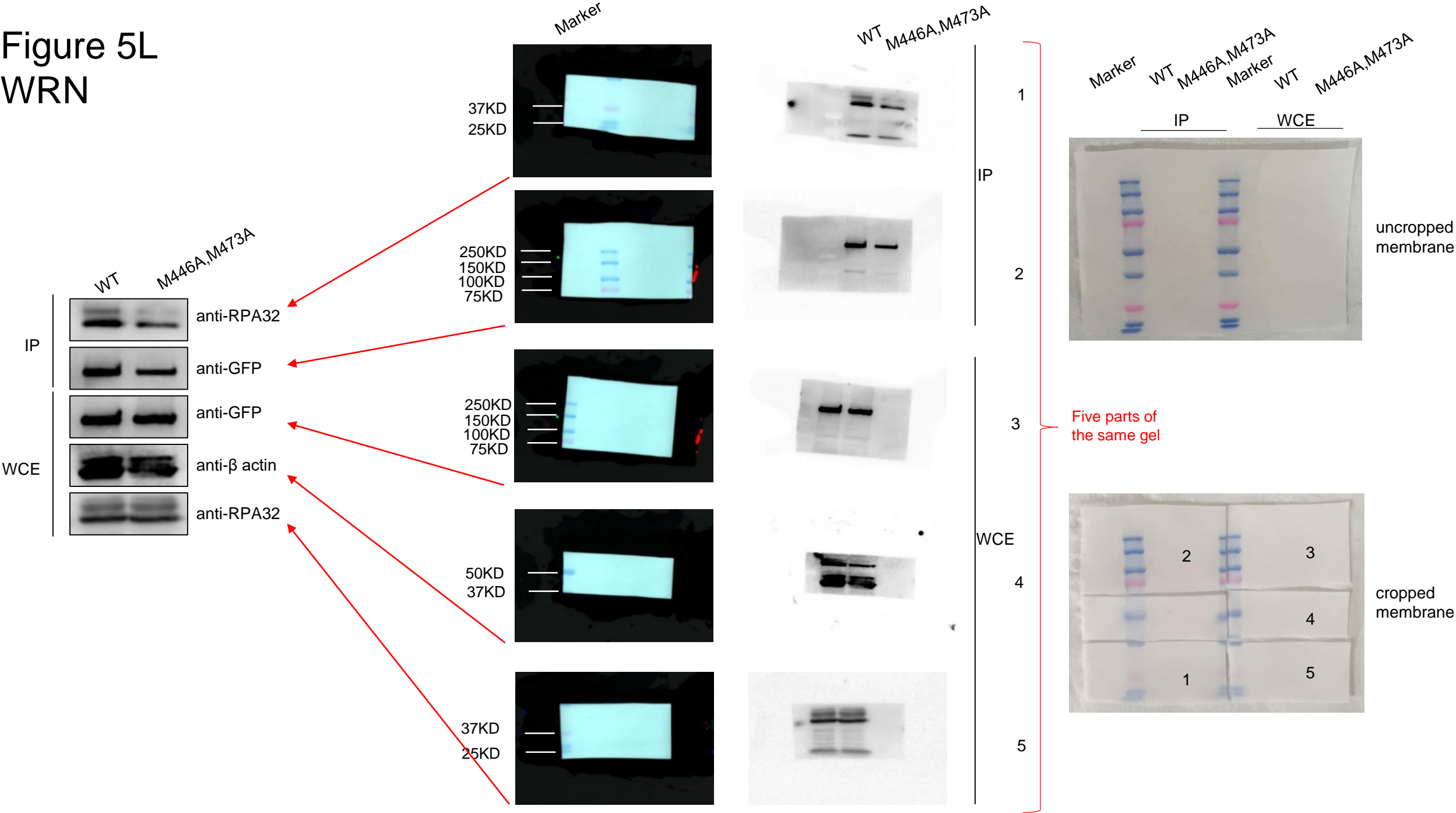

Supplement: Figure 5—source data 1. [file elife-81639-fig5-data1.zip › Figure5-source data/Figure 5L.pdf]

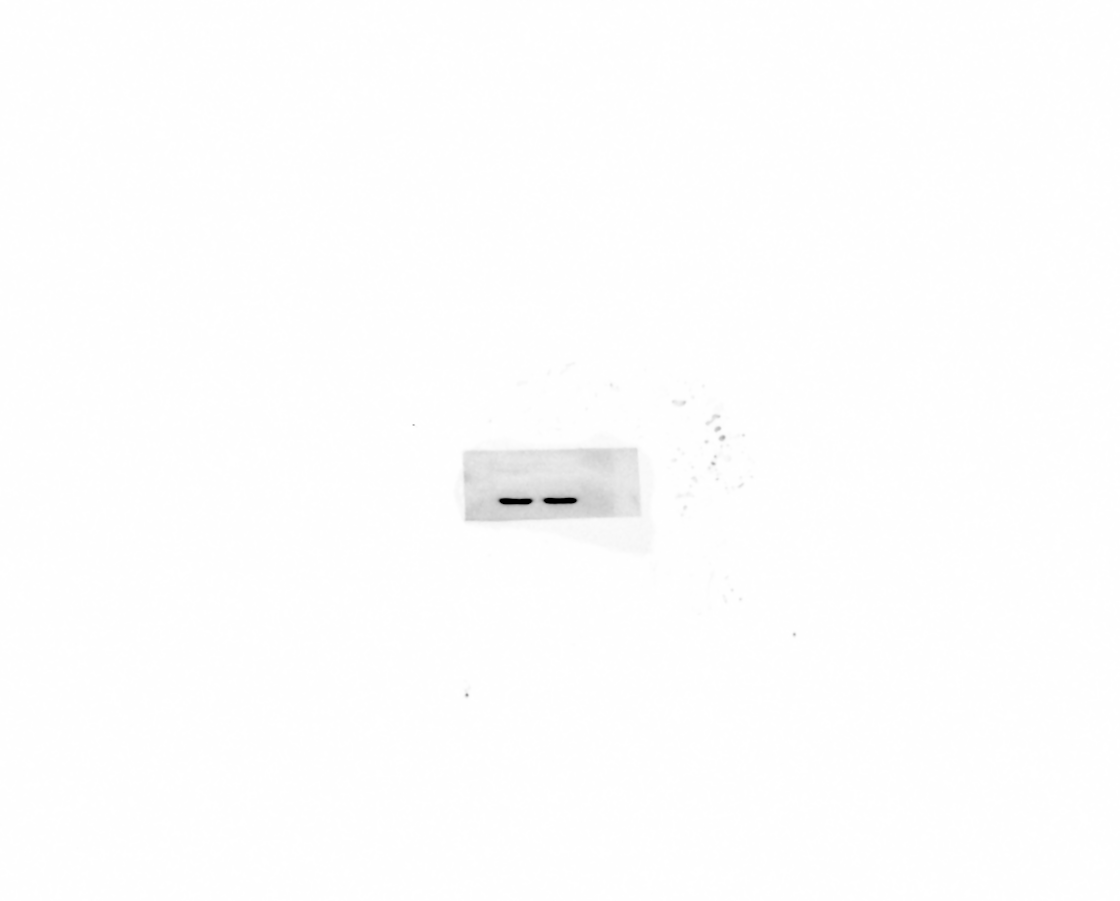

Supplement: Figure 5—source data 1. [file elife-81639-fig5-data1.zip › Figure5-source data/Figure 5L Repeat1/WCE-anti╬▓ actin.tif]

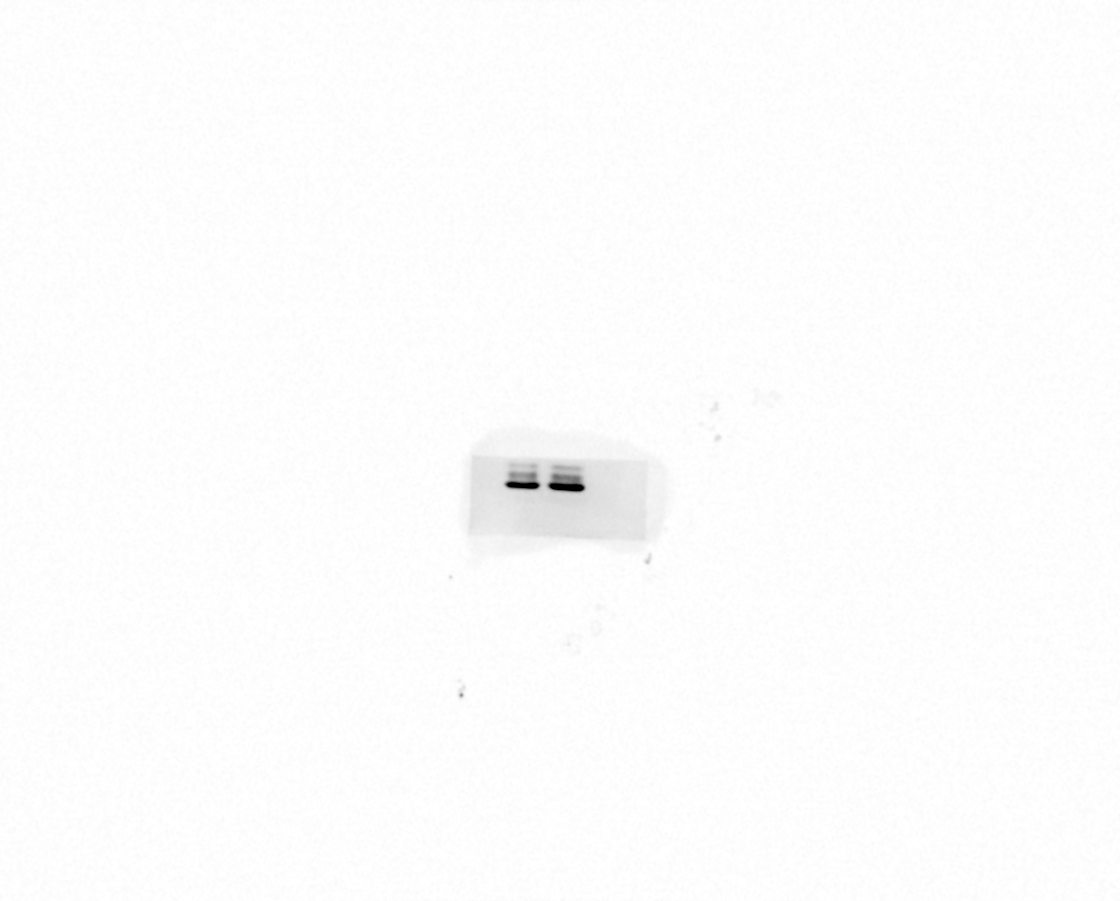

Supplement: Figure 5—source data 1. [file elife-81639-fig5-data1.zip › Figure5-source data/Figure 5L Repeat1/WCE-antiRPA32.tif]

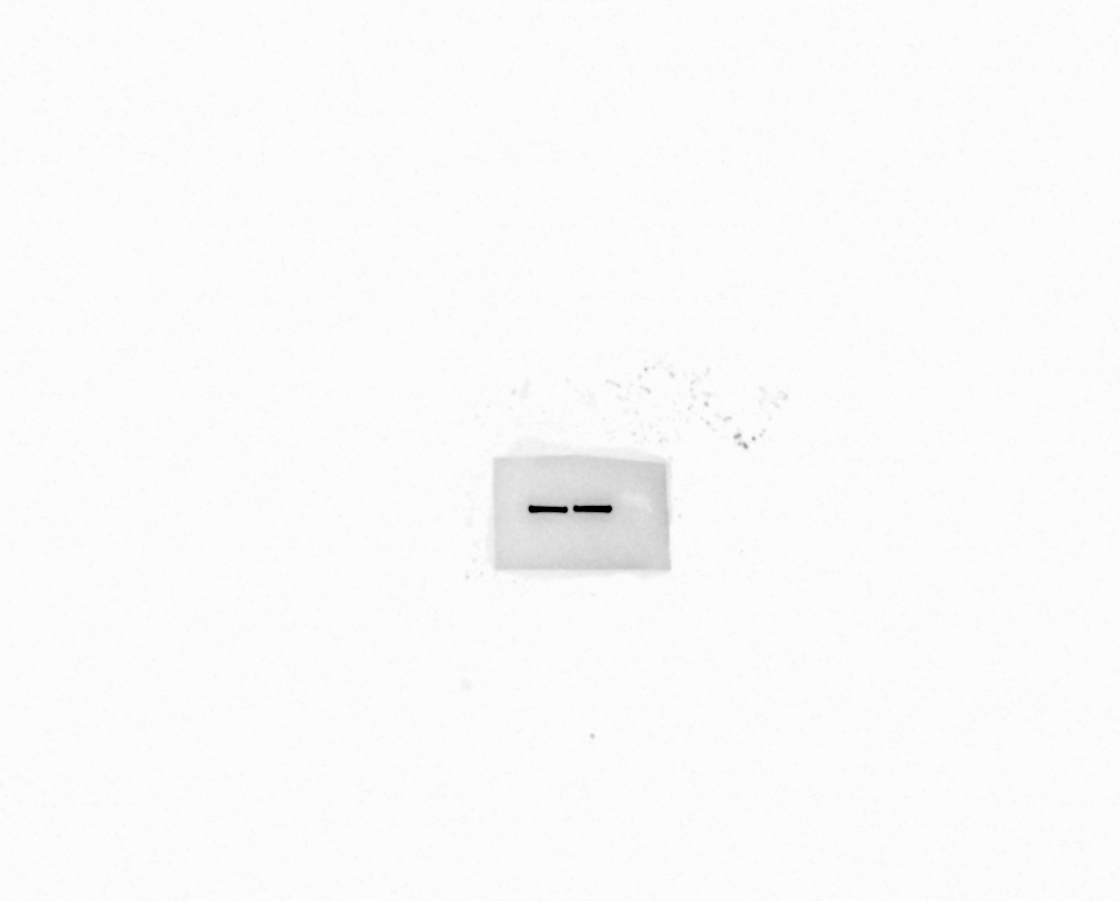

Supplement: Figure 5—source data 1. [file elife-81639-fig5-data1.zip › Figure5-source data/Figure 5L Repeat1/WCE-antiGFP.tif]

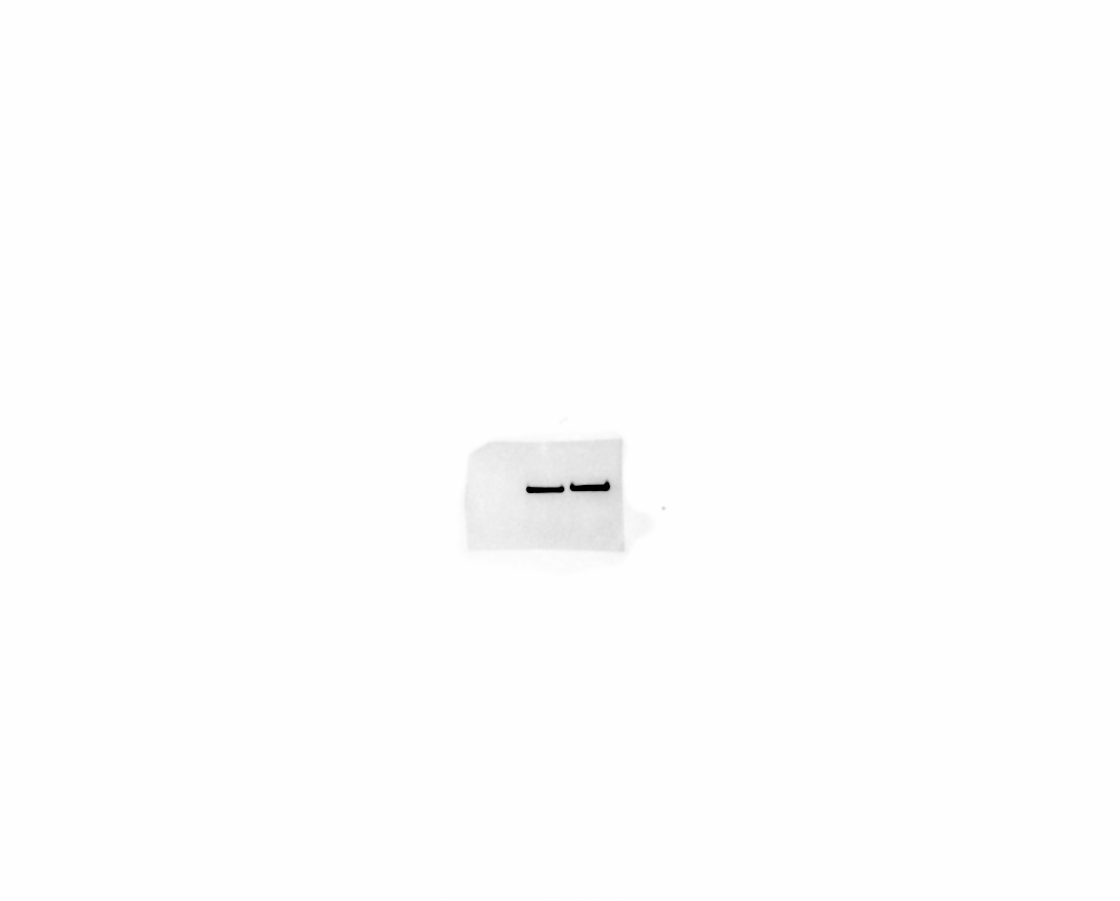

Supplement: Figure 5—source data 1. [file elife-81639-fig5-data1.zip › Figure5-source data/Figure 5L Repeat1/IP-antiGFP.tif]

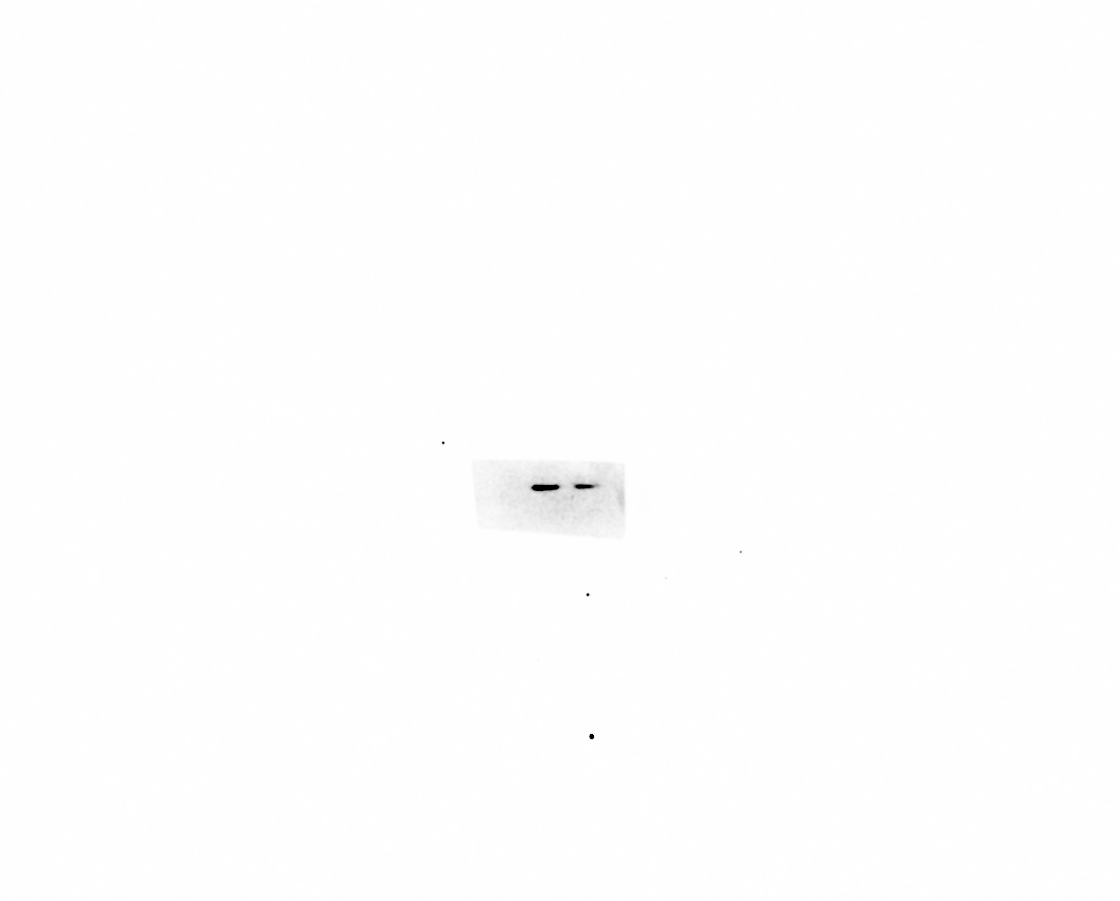

Supplement: Figure 5—source data 1. [file elife-81639-fig5-data1.zip › Figure5-source data/Figure 5L Repeat1/IP-antiRPA32.tif]

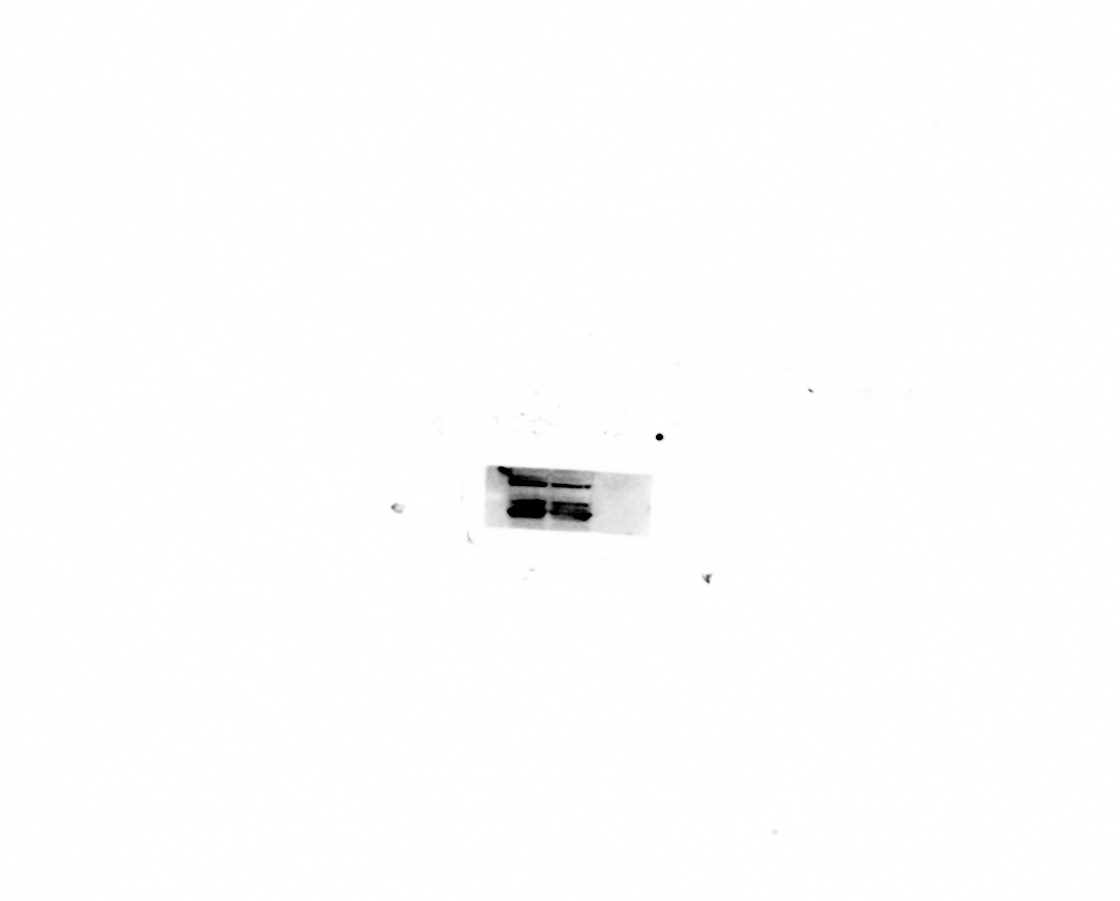

Supplement: Figure 5—source data 1. [file elife-81639-fig5-data1.zip › Figure5-source data/Figure 5L initial trial/WCE-anti╬▓ actin.tif]

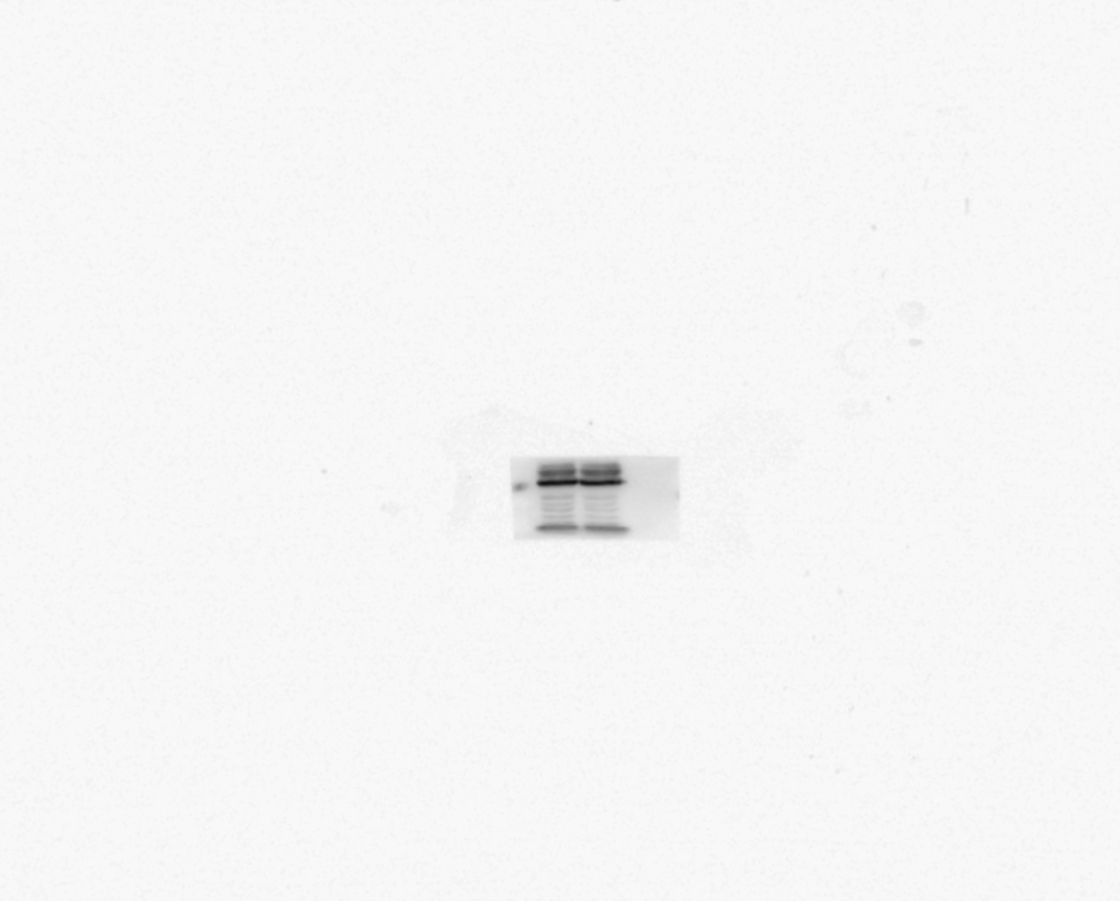

Supplement: Figure 5—source data 1. [file elife-81639-fig5-data1.zip › Figure5-source data/Figure 5L initial trial/WCE-antiRPA32.tif]

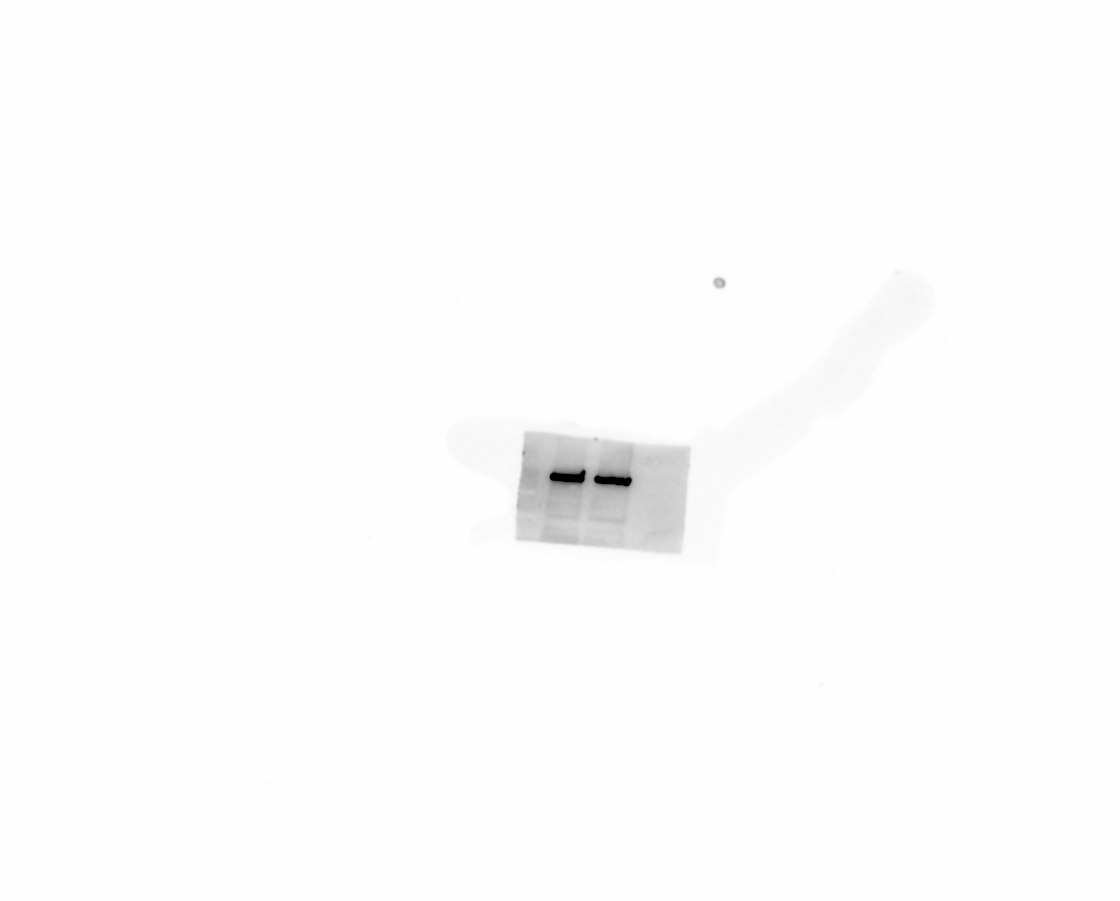

Supplement: Figure 5—source data 1. [file elife-81639-fig5-data1.zip › Figure5-source data/Figure 5L initial trial/WCE-antiGFP.tif]

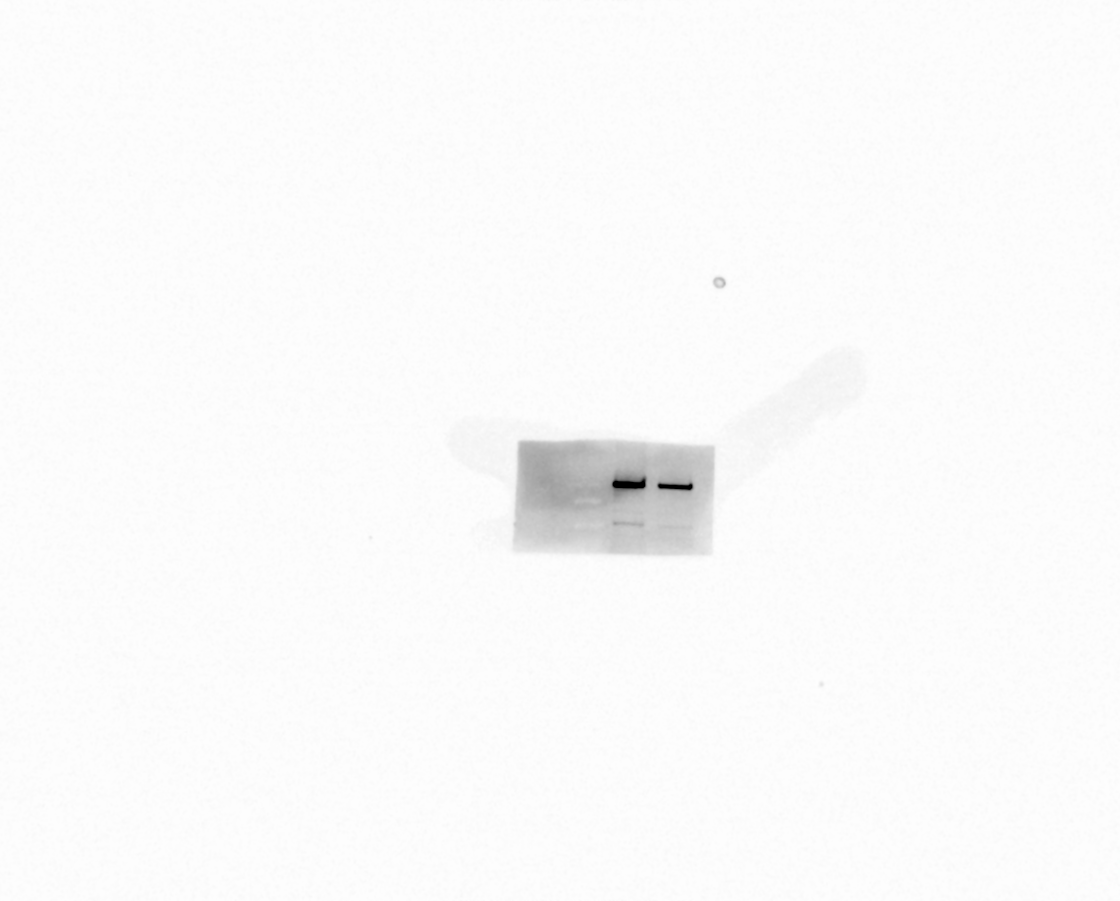

Supplement: Figure 5—source data 1. [file elife-81639-fig5-data1.zip › Figure5-source data/Figure 5L initial trial/IP-antiGFP.tif]

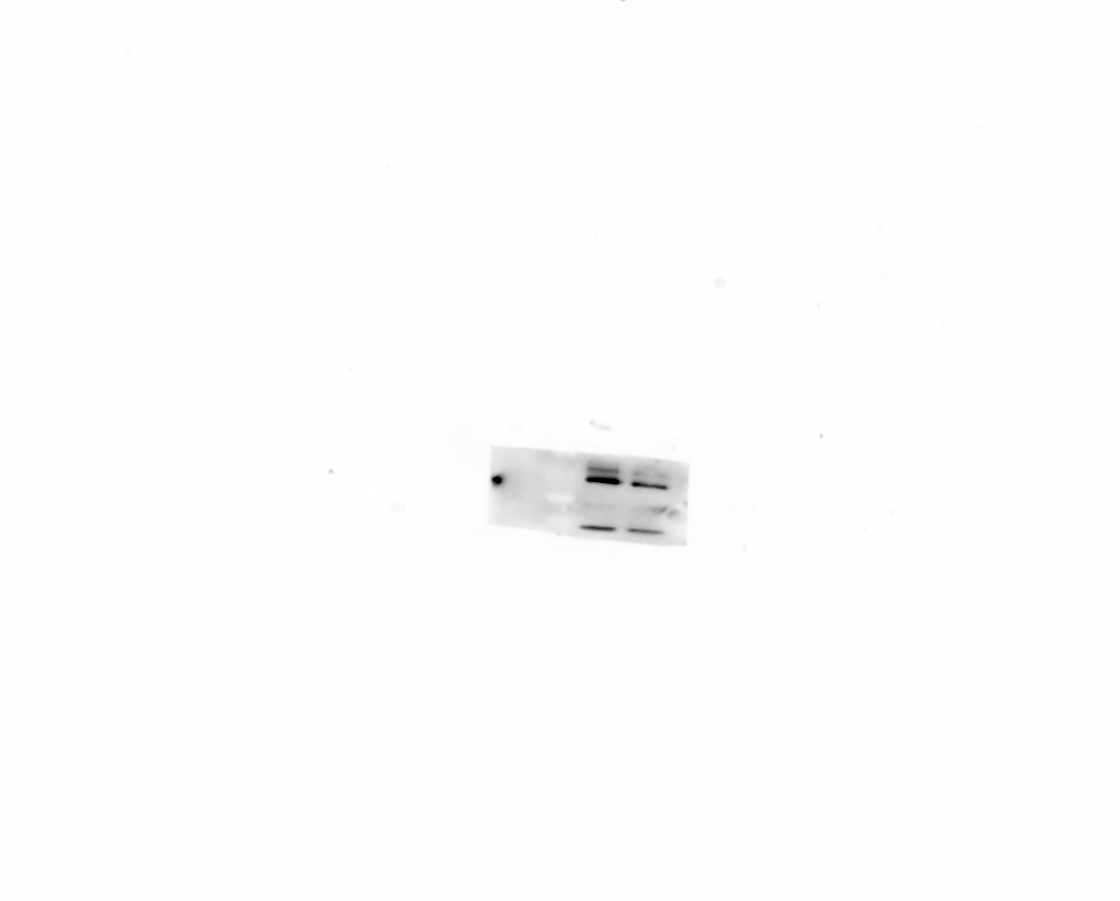

Supplement: Figure 5—source data 1. [file elife-81639-fig5-data1.zip › Figure5-source data/Figure 5L initial trial/IP-antiRPA32.tif]

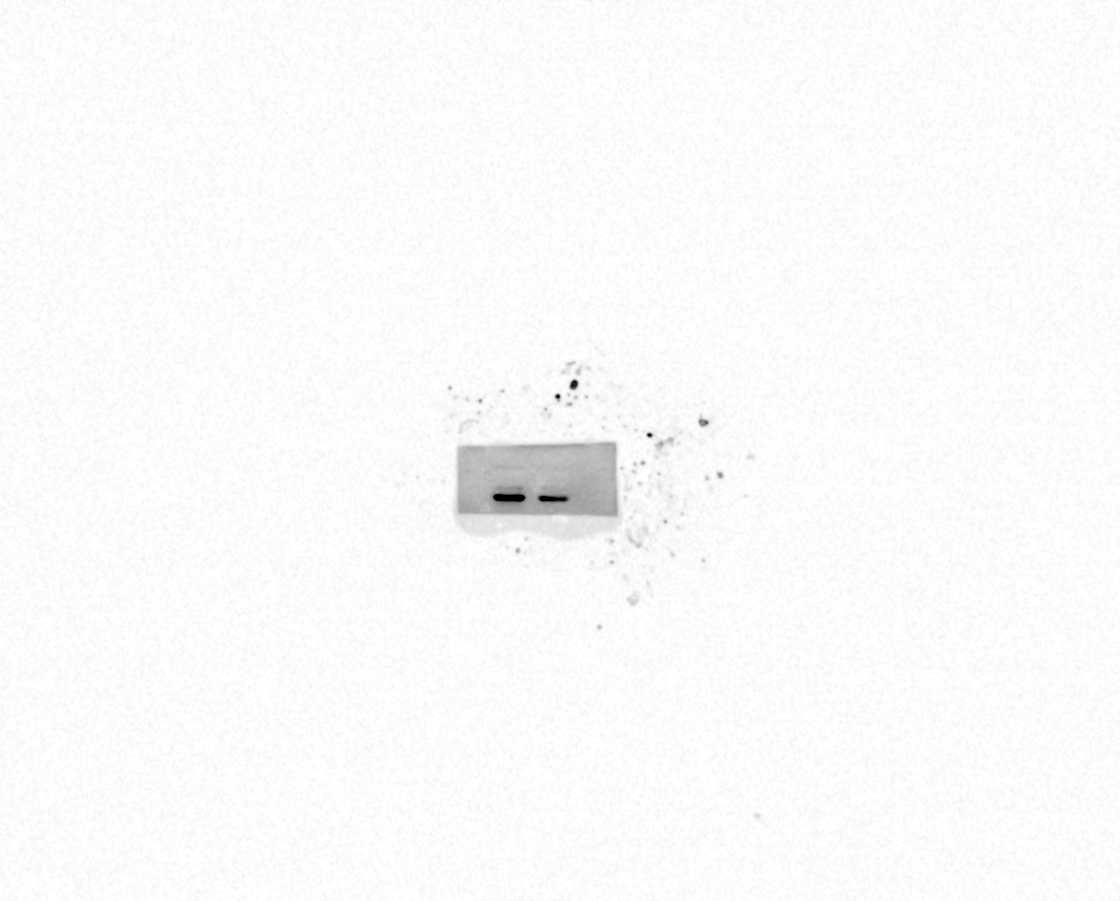

Supplement: Figure 5—source data 1. [file elife-81639-fig5-data1.zip › Figure5-source data/Figure 5L Repeat2/WCE-anti╬▓ actin.tif]

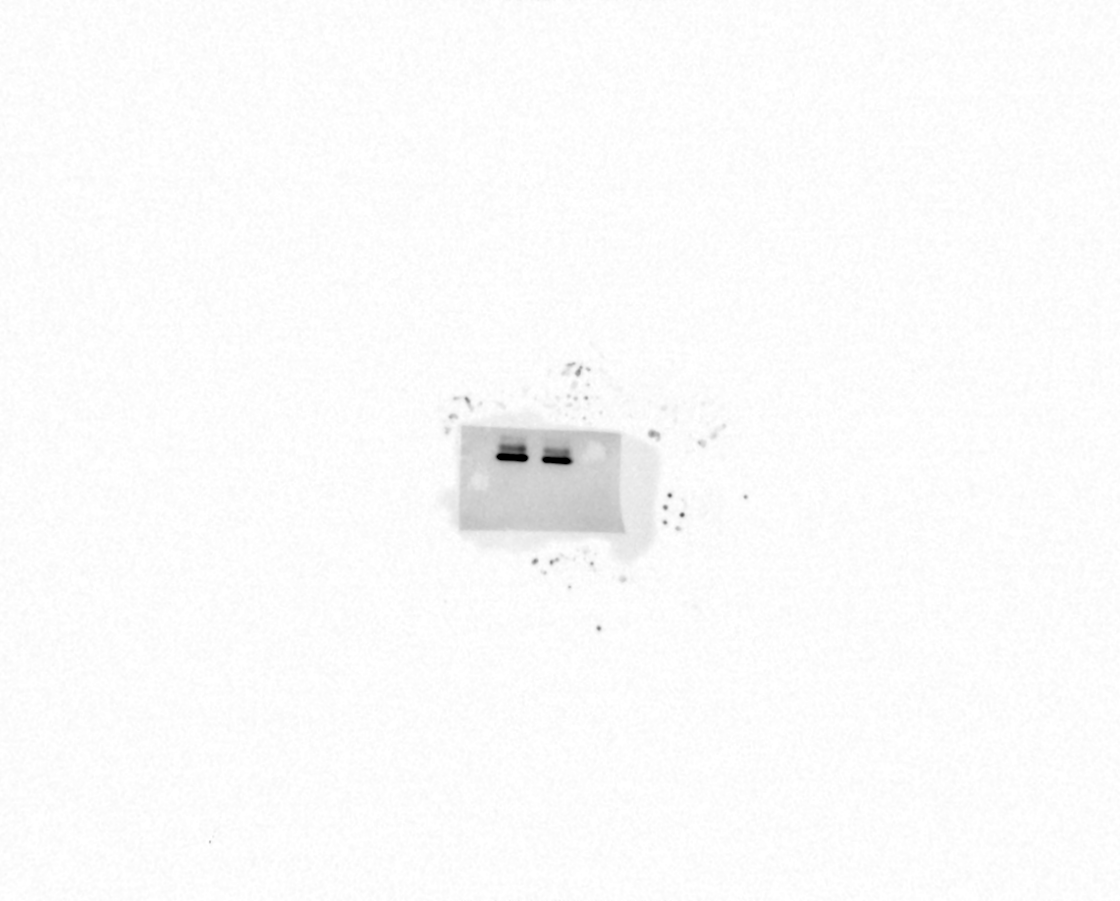

Supplement: Figure 5—source data 1. [file elife-81639-fig5-data1.zip › Figure5-source data/Figure 5L Repeat2/WCE-antiRPA32.tif]

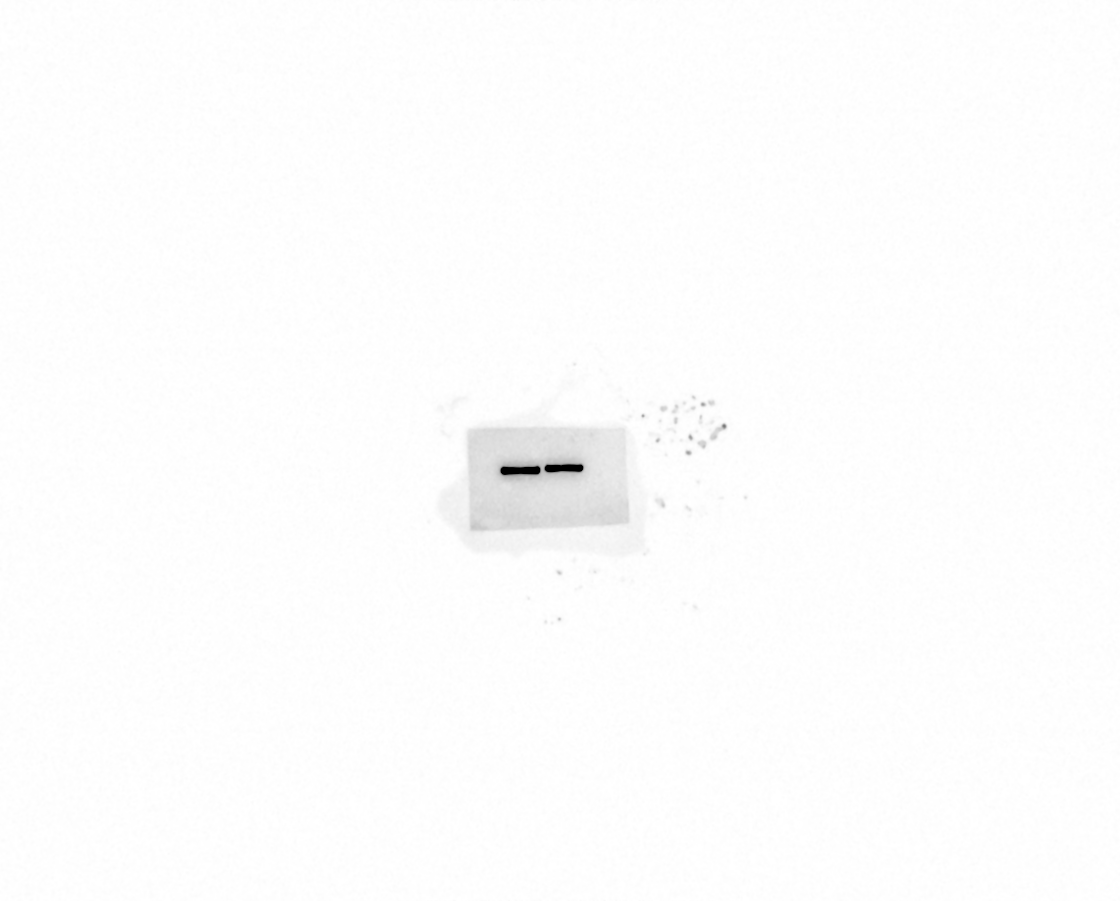

Supplement: Figure 5—source data 1. [file elife-81639-fig5-data1.zip › Figure5-source data/Figure 5L Repeat2/WCE-antiGFP.tif]

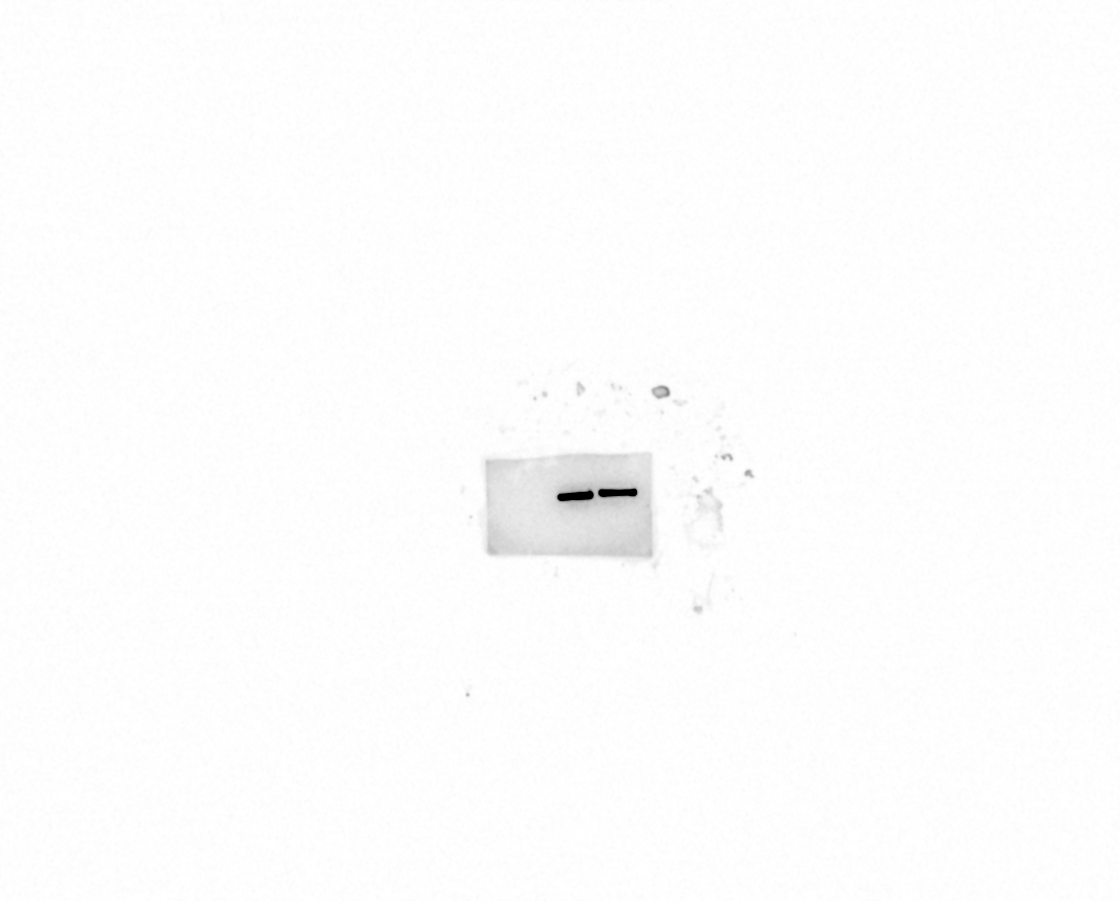

Supplement: Figure 5—source data 1. [file elife-81639-fig5-data1.zip › Figure5-source data/Figure 5L Repeat2/IP-antiGFP.tif]

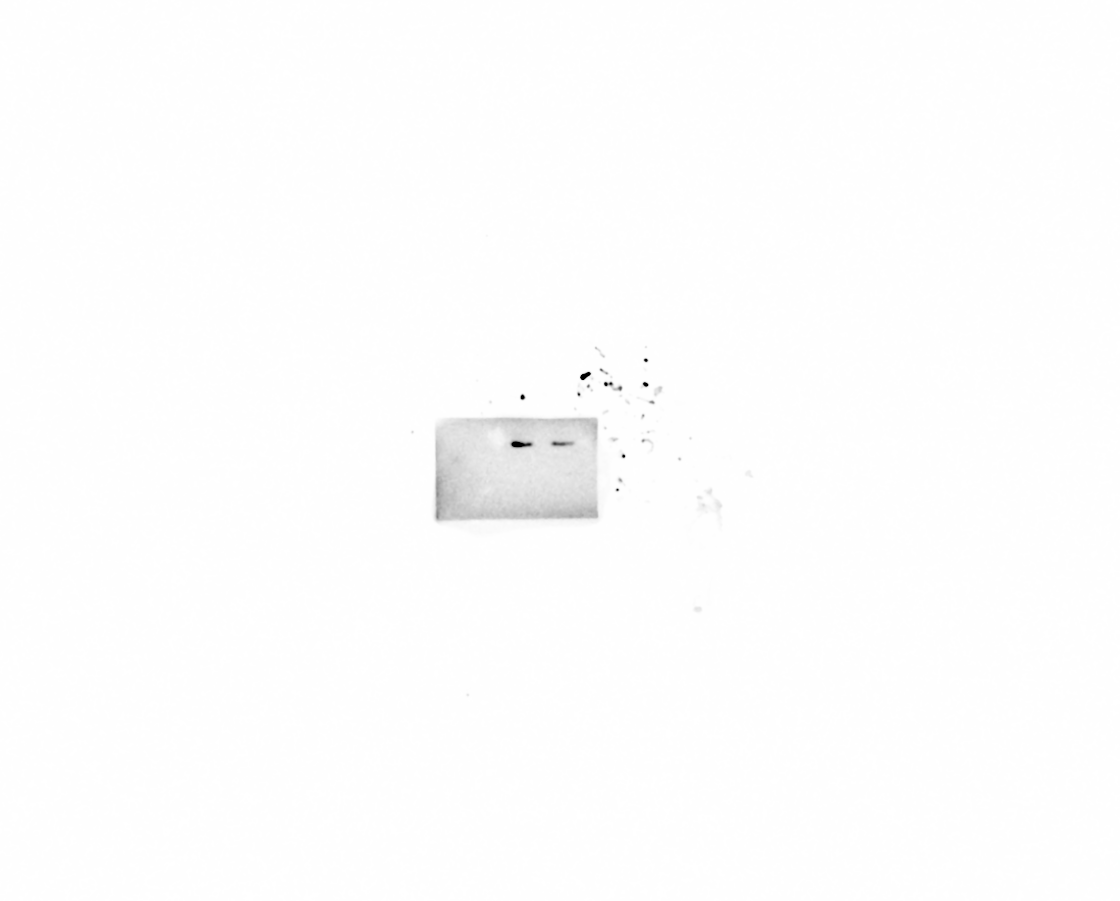

Supplement: Figure 5—source data 1. [file elife-81639-fig5-data1.zip › Figure5-source data/Figure 5L Repeat2/IP-antiRPA32.tif]

Figure 6L  
ATRIP

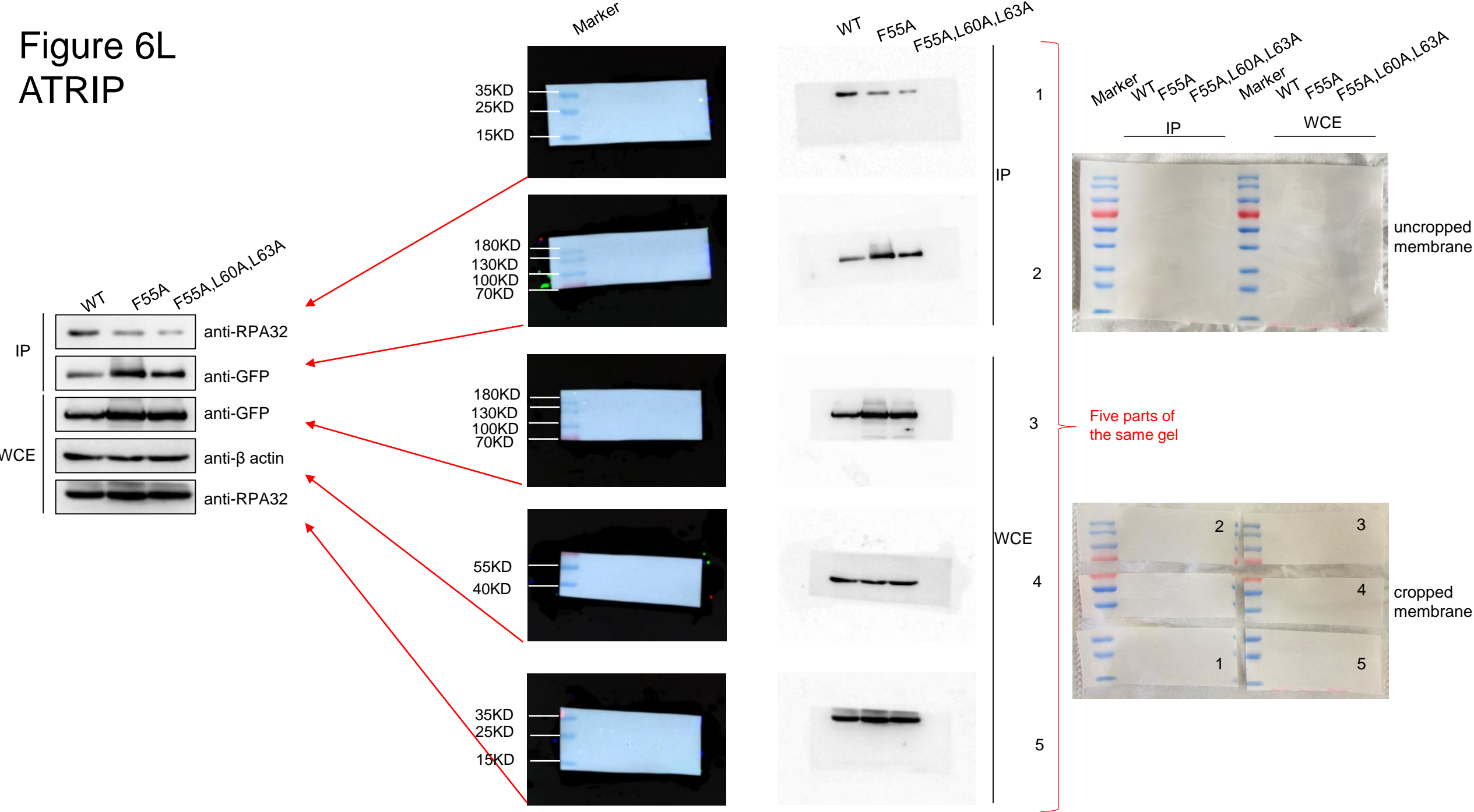

Supplement: Figure 6—source data 1. [file elife-81639-fig6-data1.zip › Figure6-source data/Figure 6L.pdf]

Figure 6L  
ATRIP

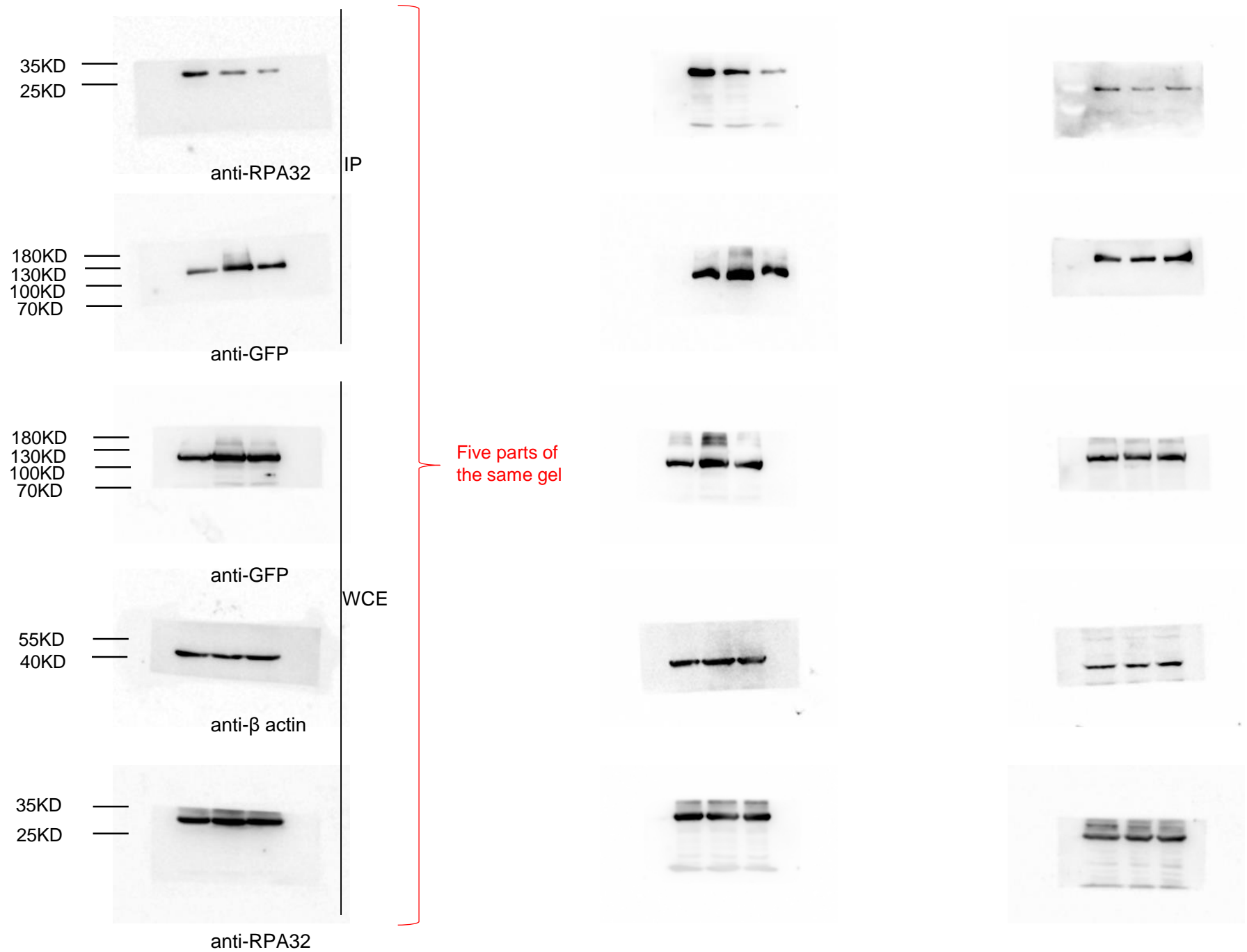

Supplement: Figure 6—source data 1. [file elife-81639-fig6-data1.zip › Figure6-source data/IP-data-Figure 6L.pdf]

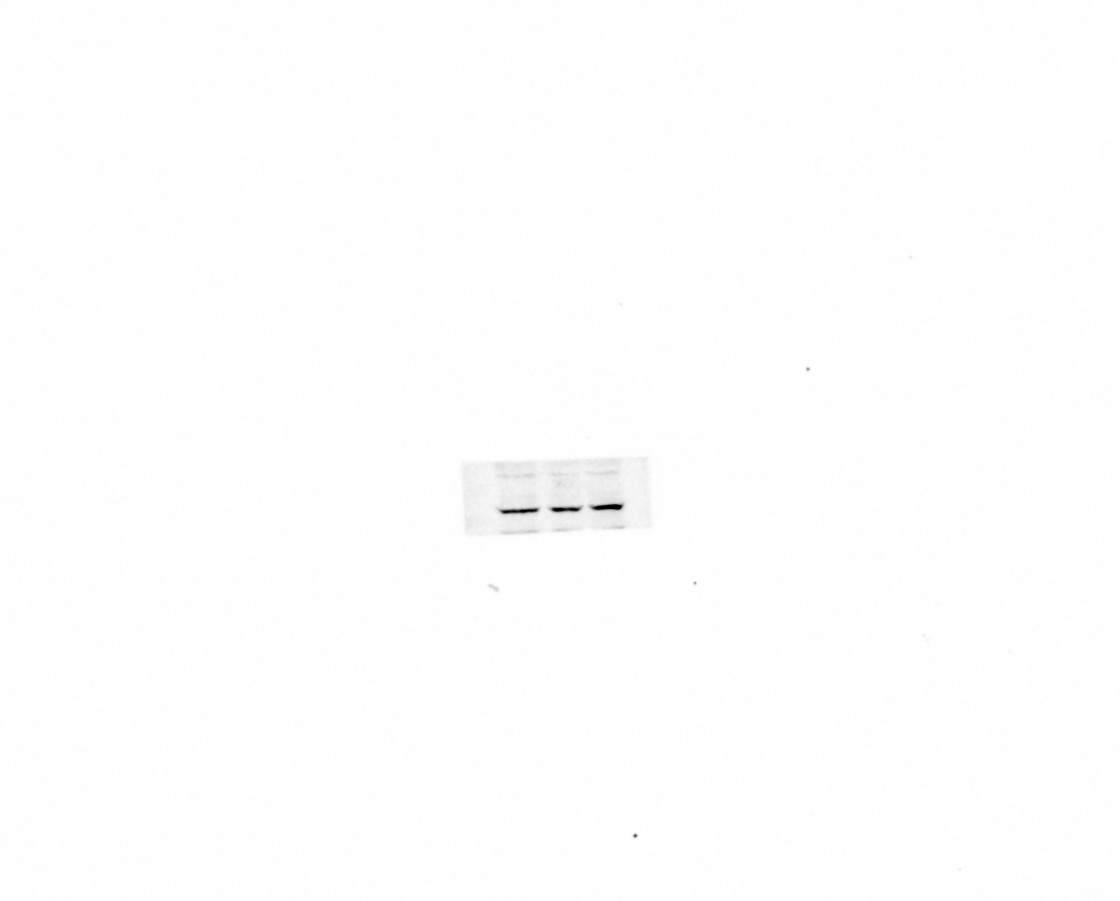

Supplement: Figure 6—source data 1. [file elife-81639-fig6-data1.zip › Figure6-source data/Figure 6L Repeat2/WCE-anti╬▓ actin.tif]

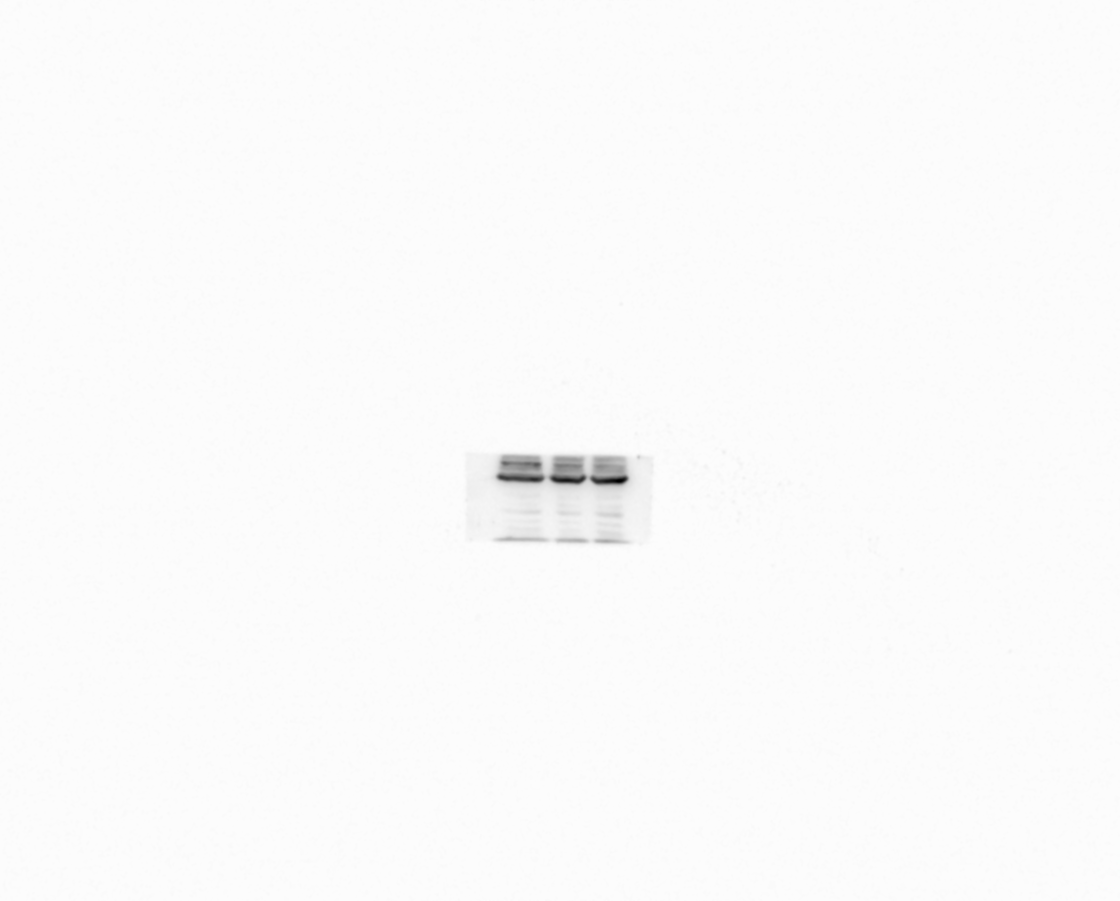

Supplement: Figure 6—source data 1. [file elife-81639-fig6-data1.zip › Figure6-source data/Figure 6L Repeat2/WCE-antiRPA32.tif]

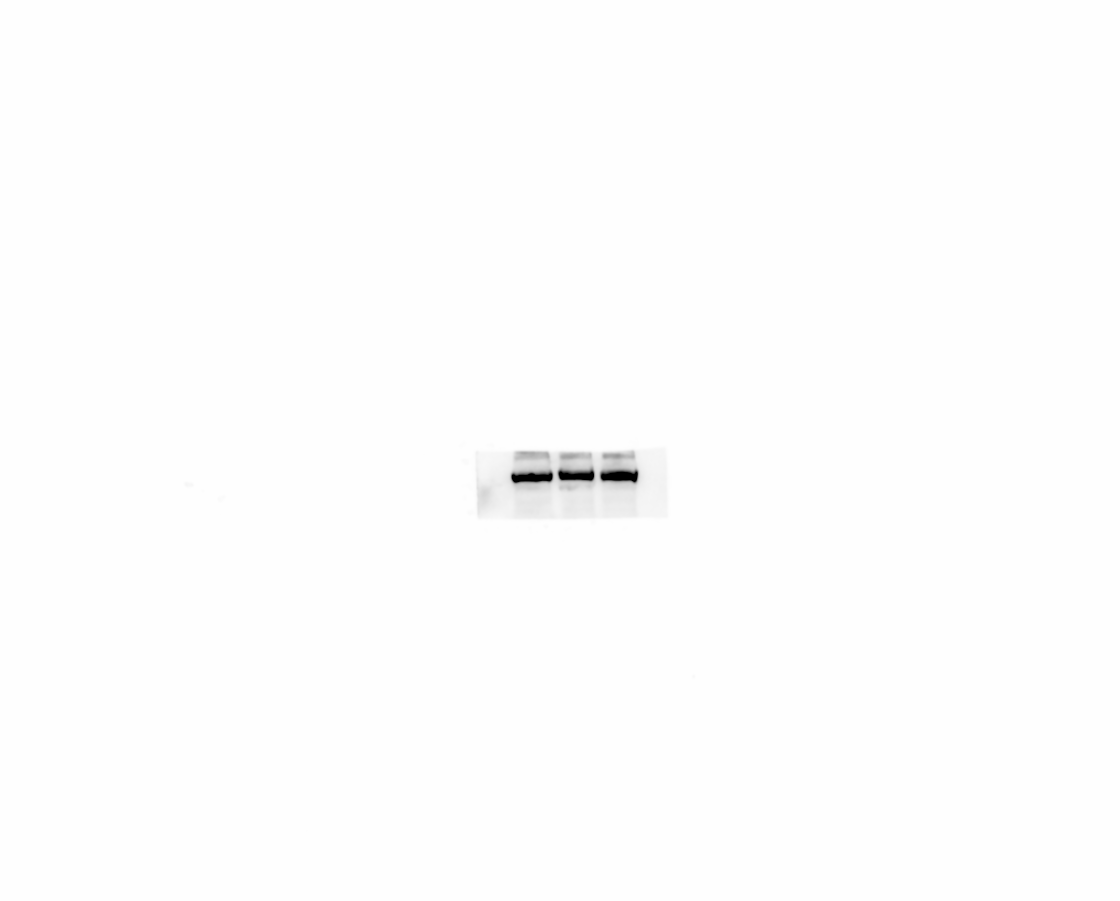

Supplement: Figure 6—source data 1. [file elife-81639-fig6-data1.zip › Figure6-source data/Figure 6L Repeat2/WCE-antiGFP.tif]

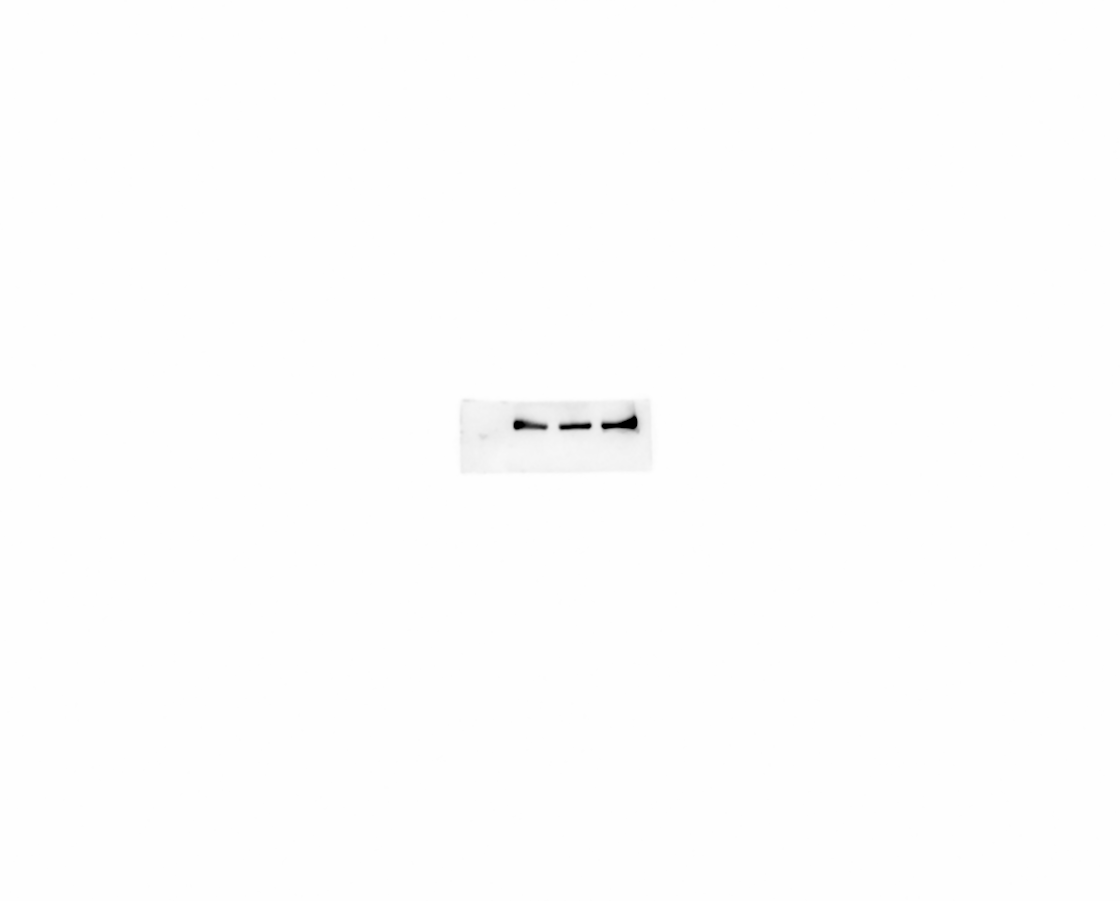

Supplement: Figure 6—source data 1. [file elife-81639-fig6-data1.zip › Figure6-source data/Figure 6L Repeat2/IP-antiGFP.tif]

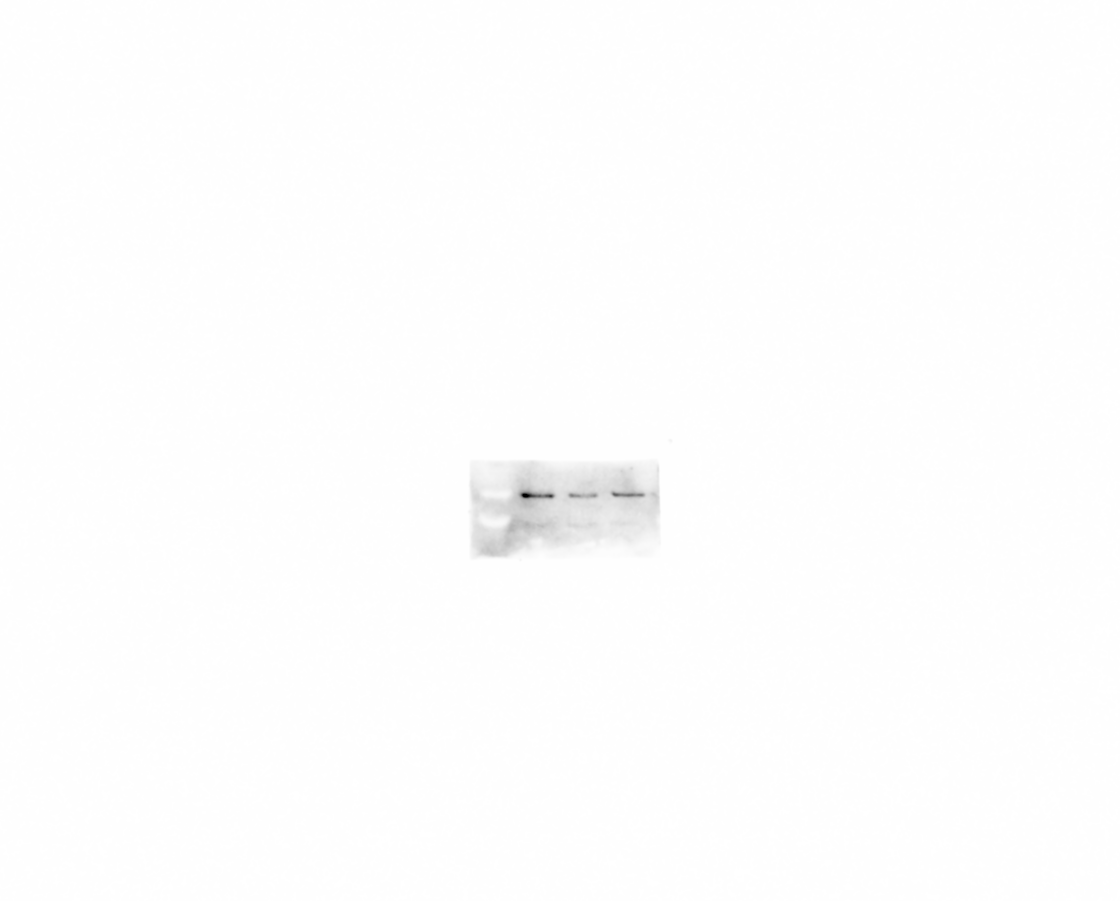

Supplement: Figure 6—source data 1. [file elife-81639-fig6-data1.zip › Figure6-source data/Figure 6L Repeat2/IP-antiRPA32.tif]

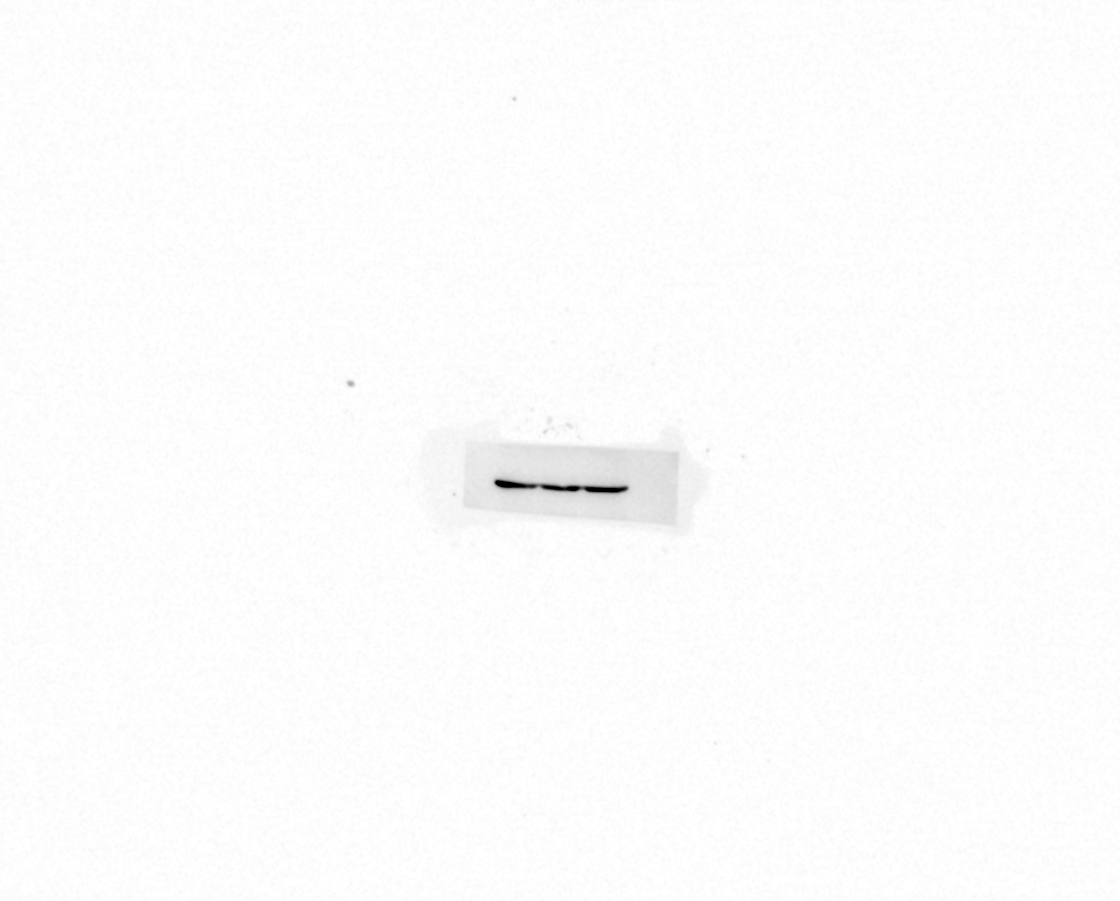

Supplement: Figure 6—source data 1. [file elife-81639-fig6-data1.zip › Figure6-source data/Figure 6L initial trial/WCE-anti╬▓ actin.tif]

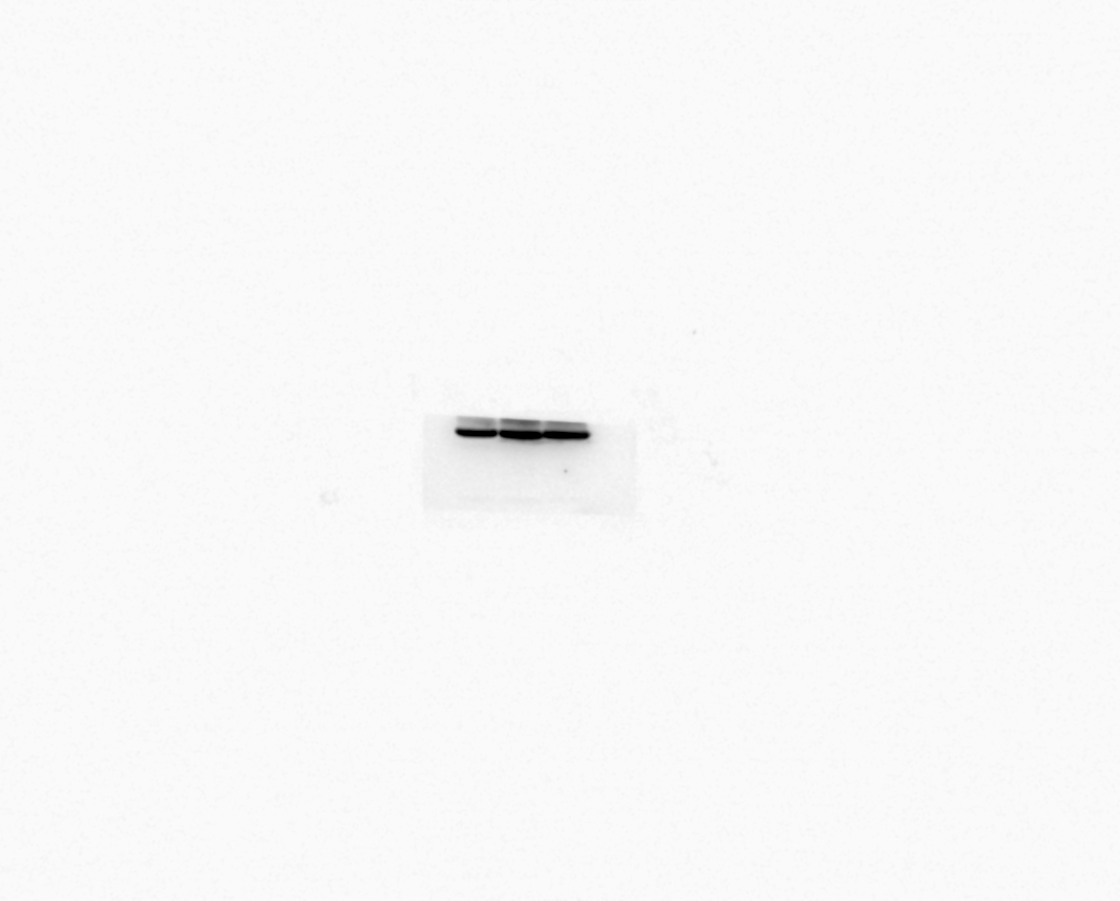

Supplement: Figure 6—source data 1. [file elife-81639-fig6-data1.zip › Figure6-source data/Figure 6L initial trial/WCE-antiRPA32.tif]

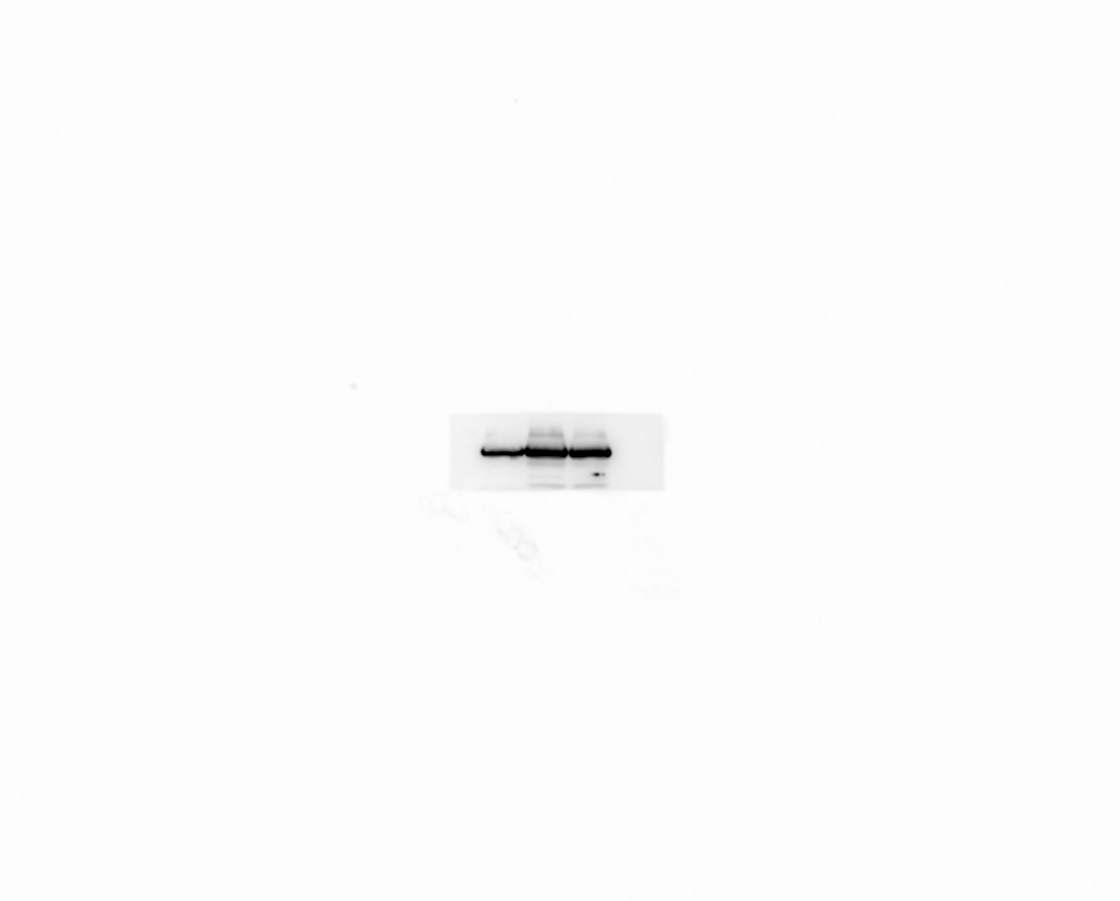

Supplement: Figure 6—source data 1. [file elife-81639-fig6-data1.zip › Figure6-source data/Figure 6L initial trial/WCE-antiGFP.tif]

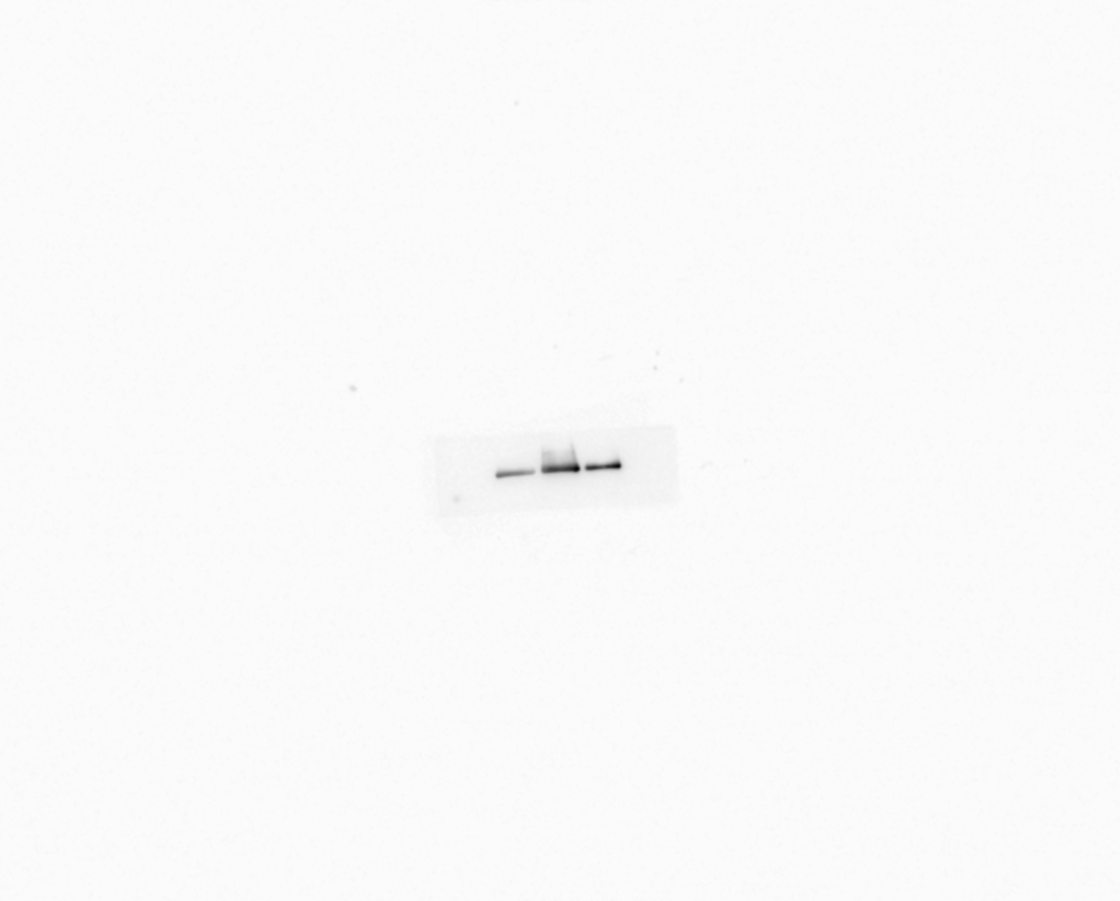

Supplement: Figure 6—source data 1. [file elife-81639-fig6-data1.zip › Figure6-source data/Figure 6L initial trial/IP-antiGFP.tif]

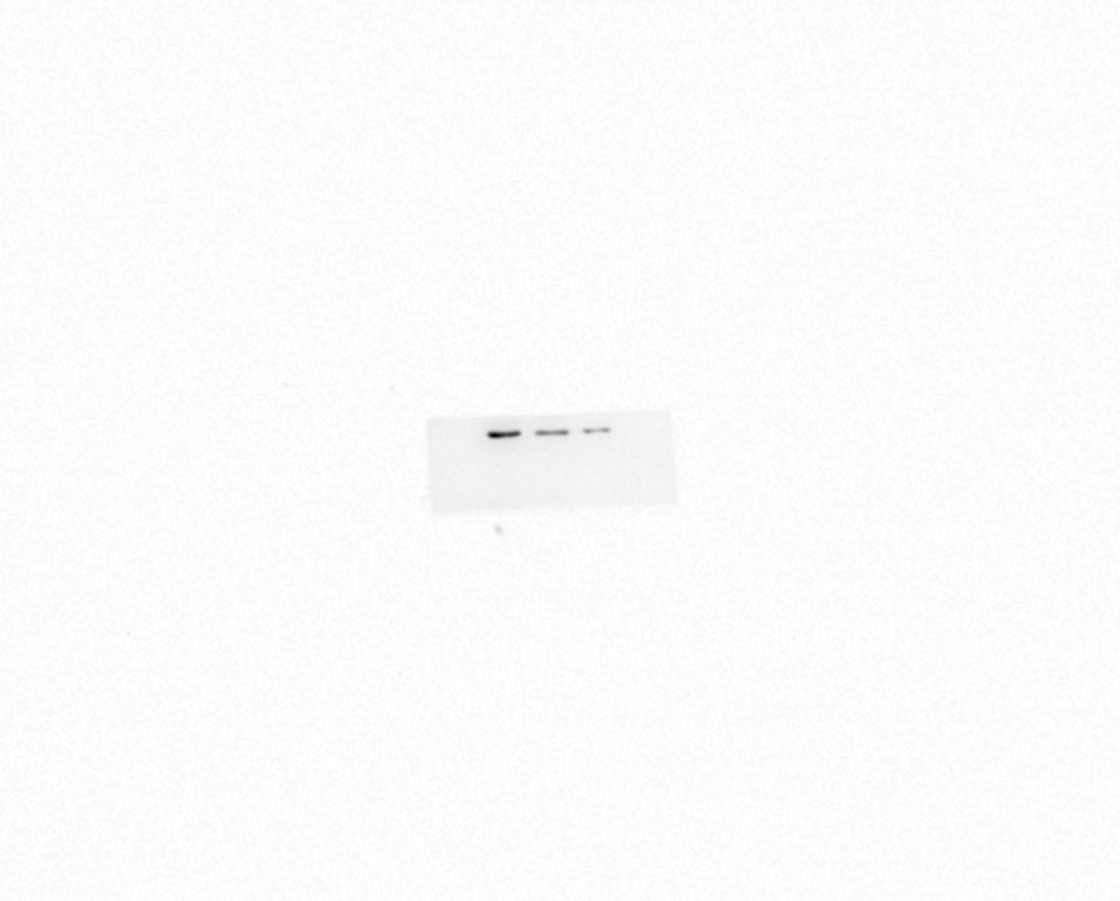

Supplement: Figure 6—source data 1. [file elife-81639-fig6-data1.zip › Figure6-source data/Figure 6L initial trial/IP-antiRPA32.tif]

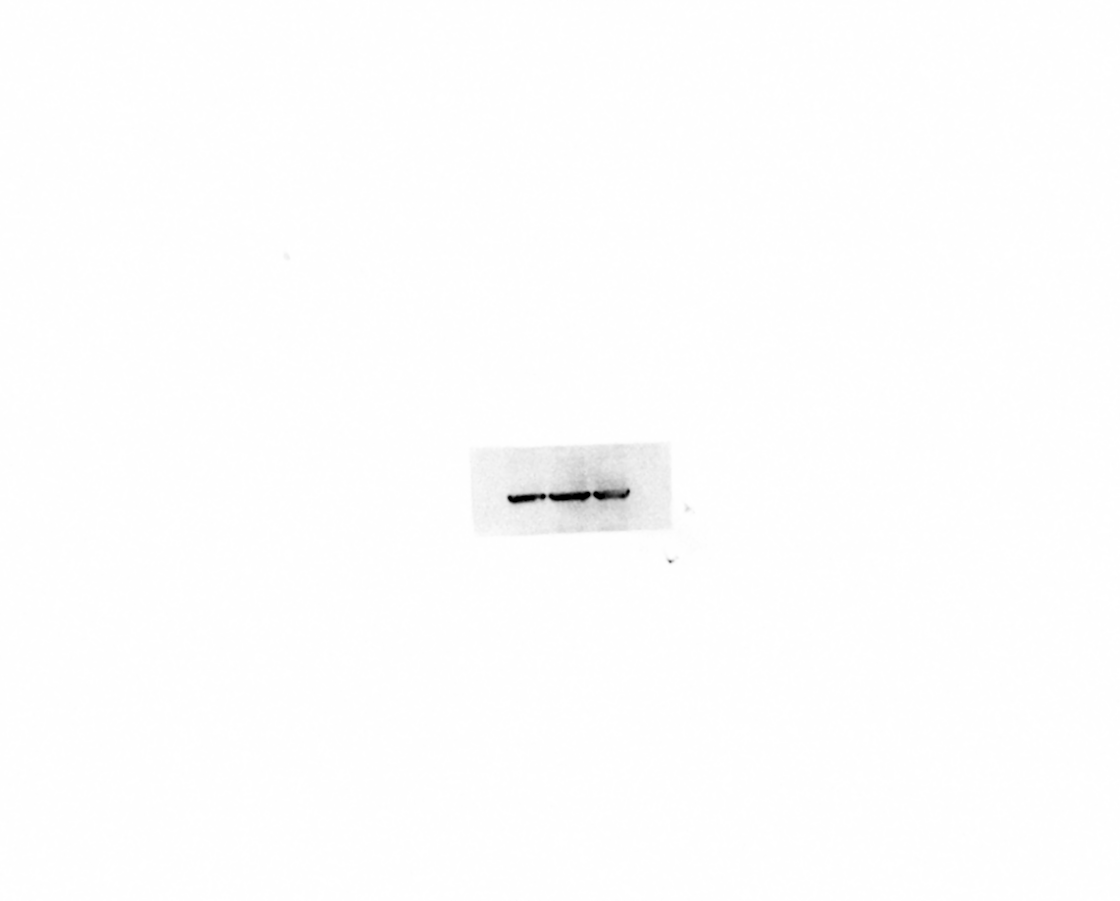

Supplement: Figure 6—source data 1. [file elife-81639-fig6-data1.zip › Figure6-source data/Figure 6L Repeat1/WCE-anti╬▓ actin.tif]

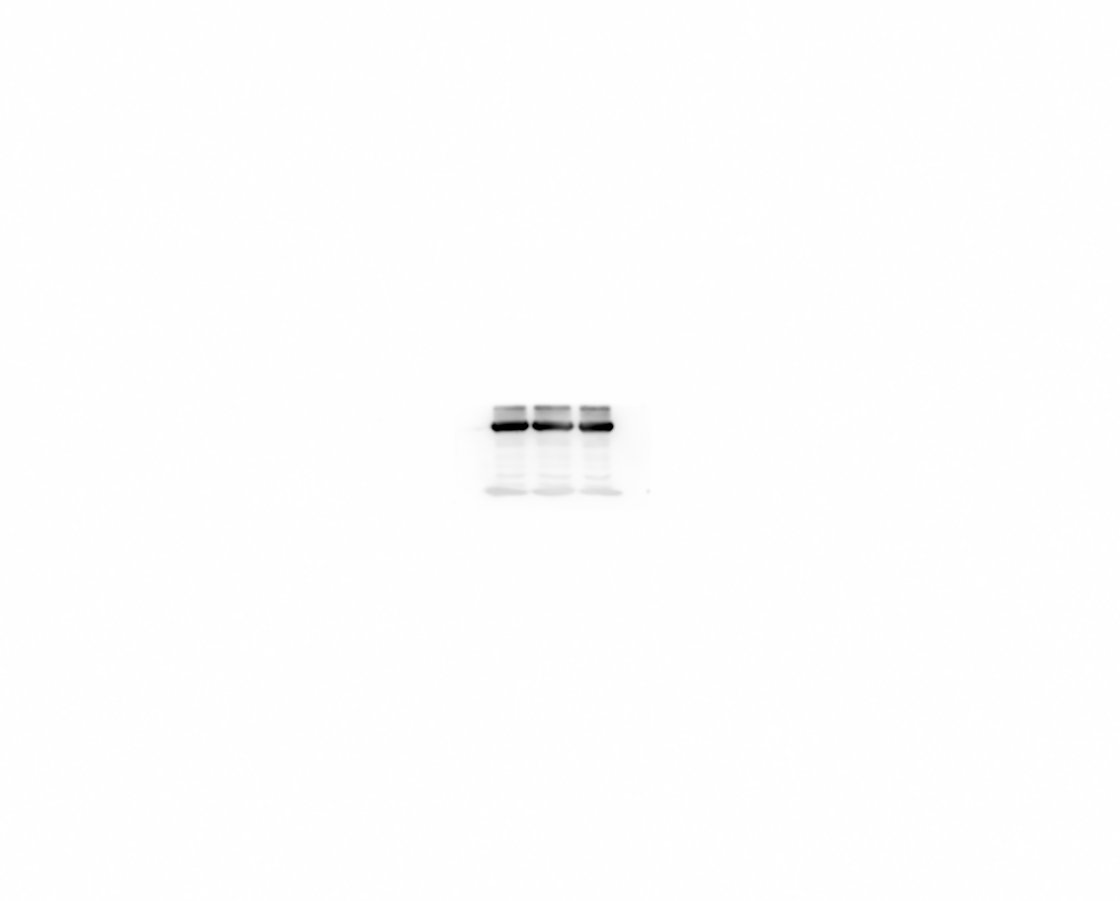

Supplement: Figure 6—source data 1. [file elife-81639-fig6-data1.zip › Figure6-source data/Figure 6L Repeat1/WCE-antiRPA32.tif]

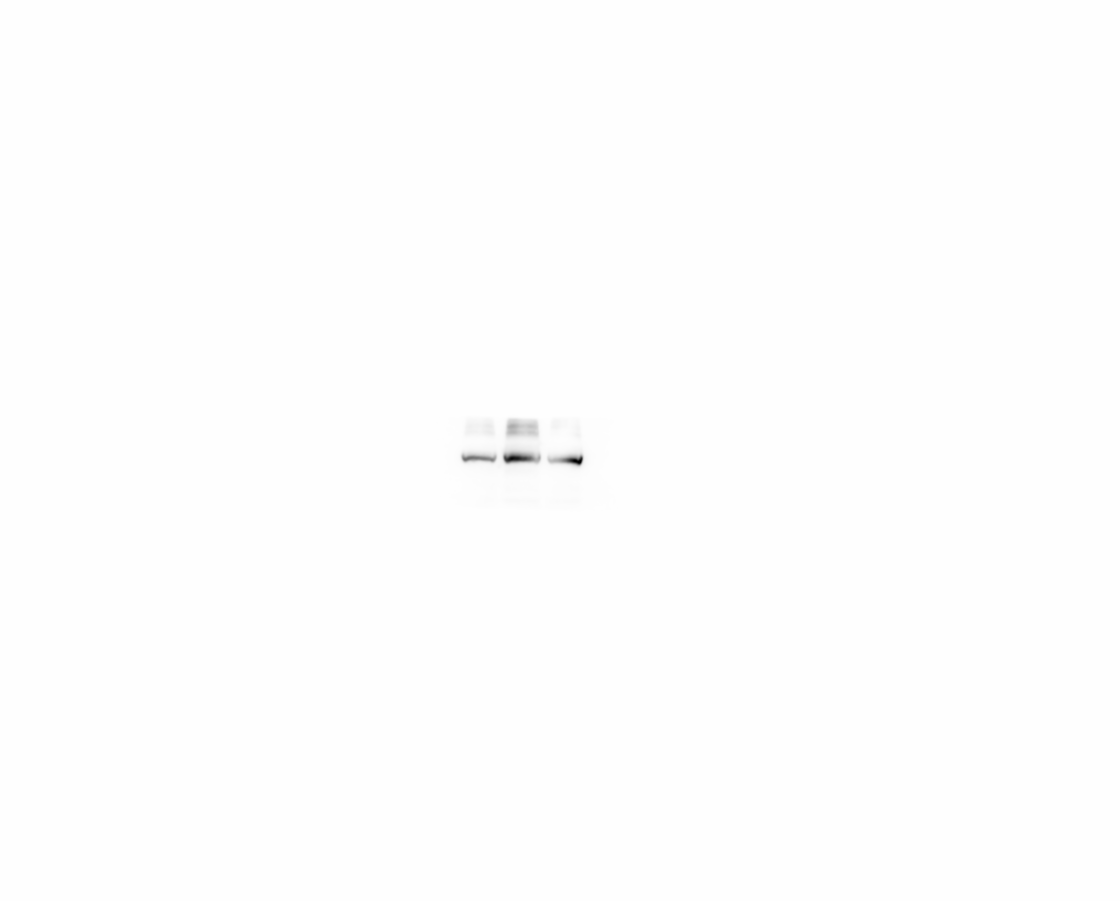

Supplement: Figure 6—source data 1. [file elife-81639-fig6-data1.zip › Figure6-source data/Figure 6L Repeat1/WCE-antiGFP.tif]

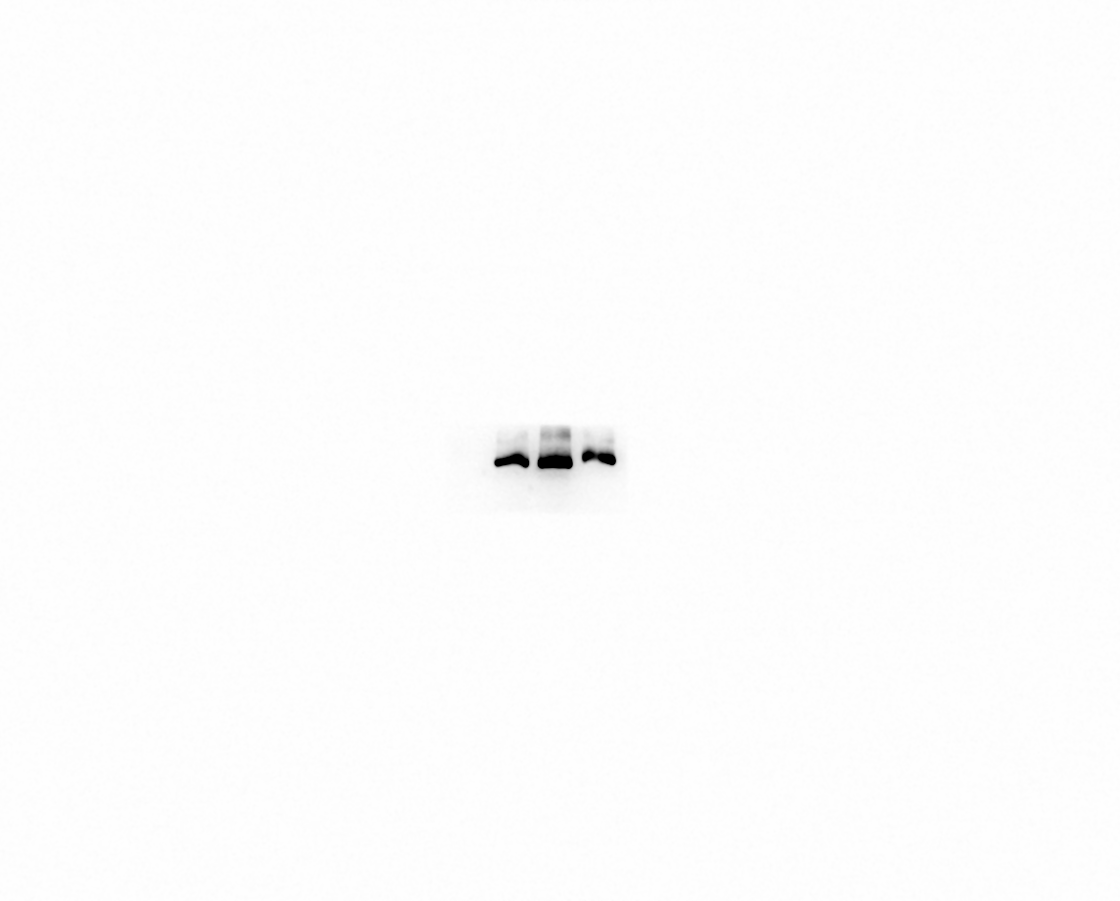

Supplement: Figure 6—source data 1. [file elife-81639-fig6-data1.zip › Figure6-source data/Figure 6L Repeat1/IP-antiGFP.tif]

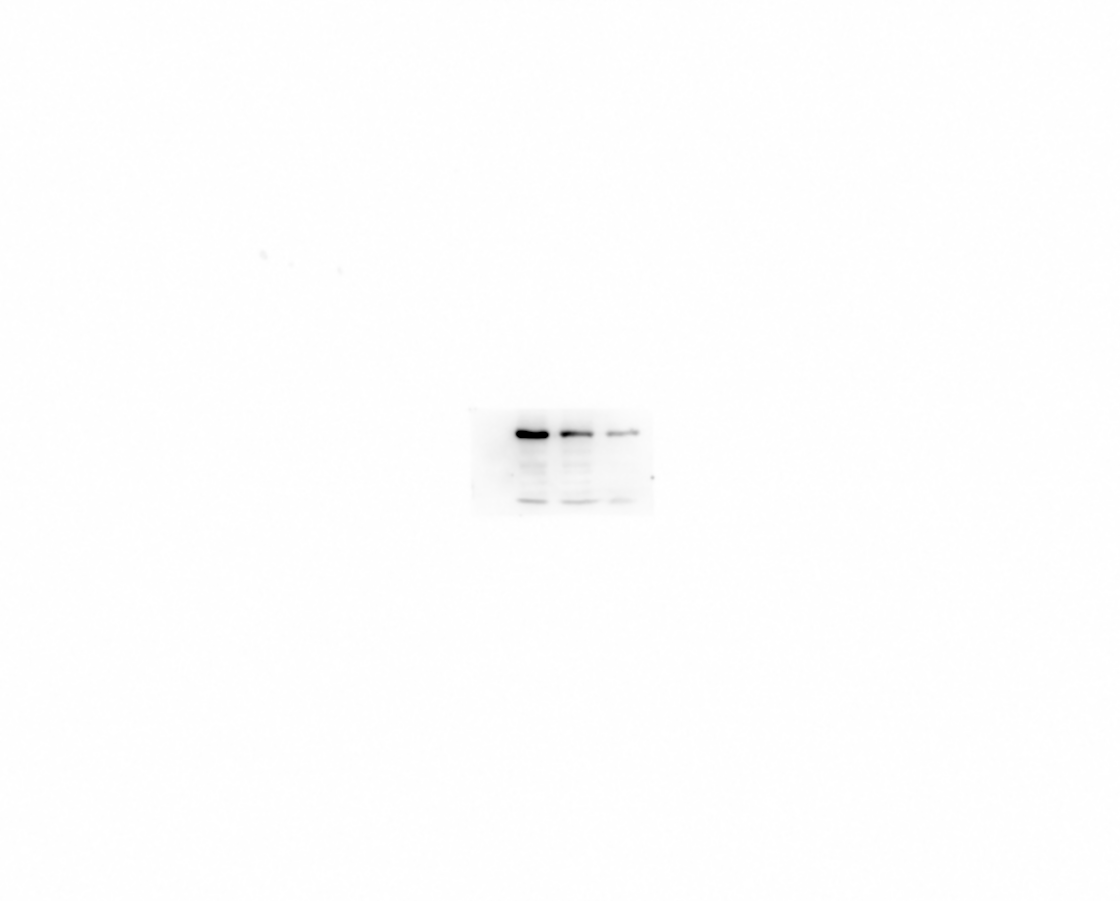

Supplement: Figure 6—source data 1. [file elife-81639-fig6-data1.zip › Figure6-source data/Figure 6L Repeat1/IP-antiRPA32.tif]

Figure 7E  
MRE11

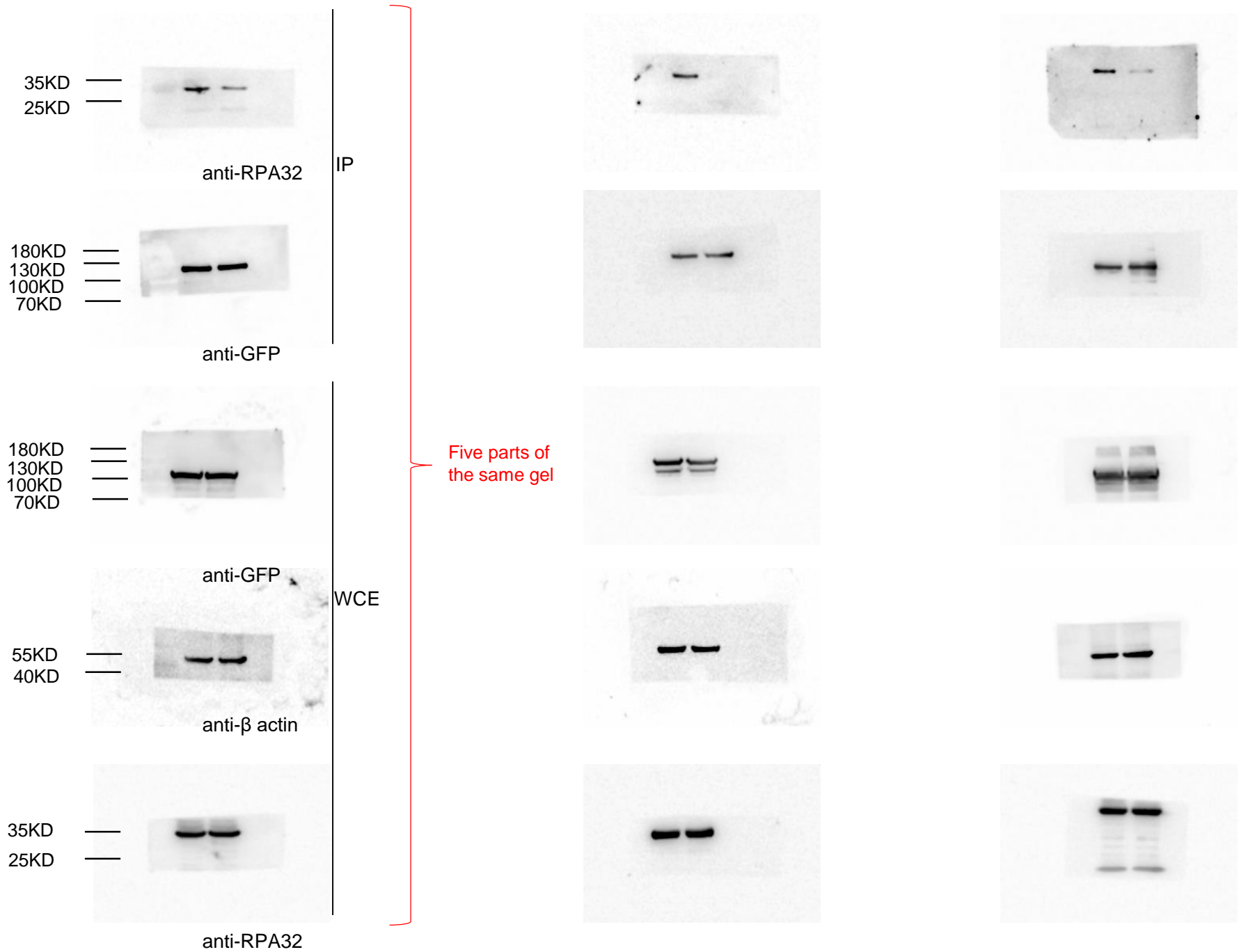

Supplement: Figure 7—source data 1. [file elife-81639-fig7-data1.zip › Figure7-source data/IP-data-Figure 7E.pdf]

Figure 7E  
MRE11

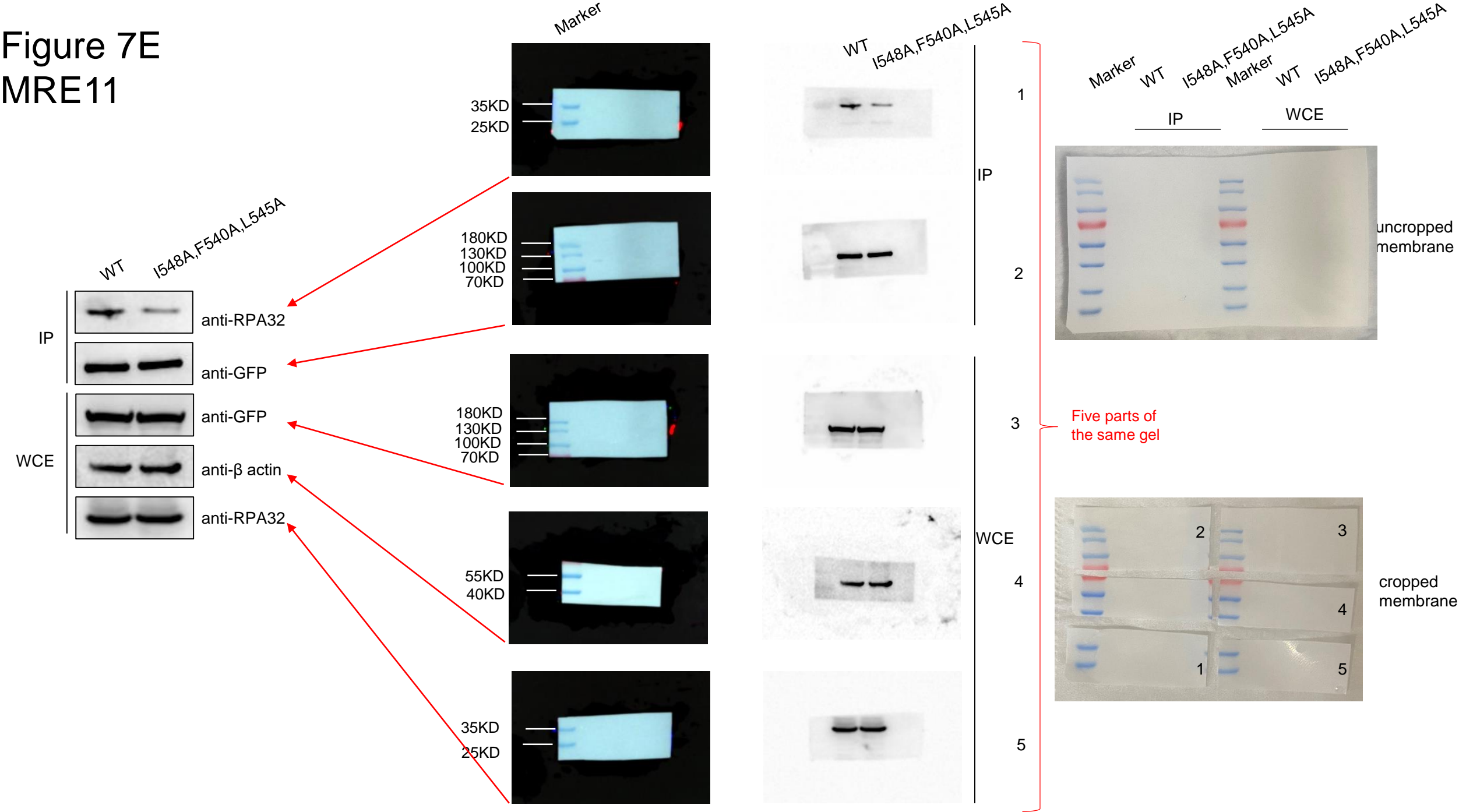

Supplement: Figure 7—source data 1. [file elife-81639-fig7-data1.zip › Figure7-source data/Figure 7E.pdf]

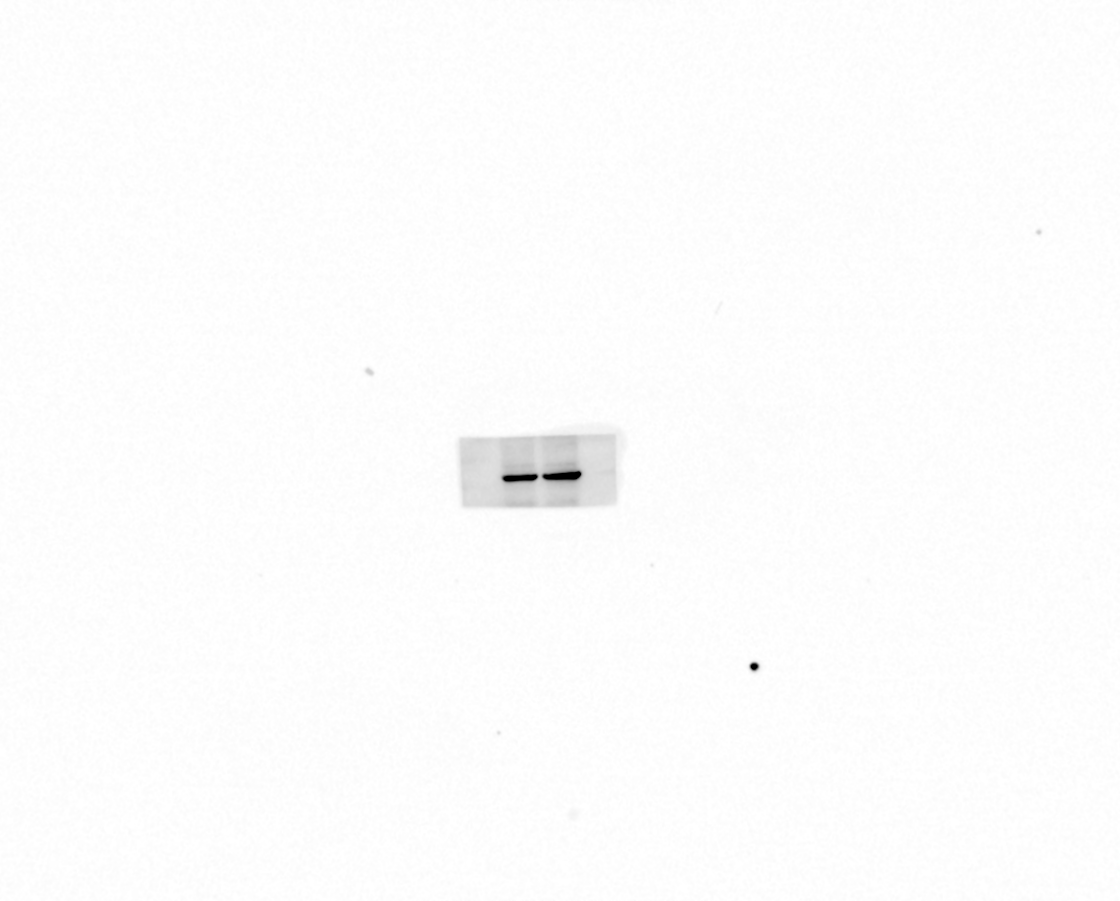

Supplement: Figure 7—source data 1. [file elife-81639-fig7-data1.zip › Figure7-source data/Figure 7E Repeat2/WCE-anti╬▓ actin.tif]

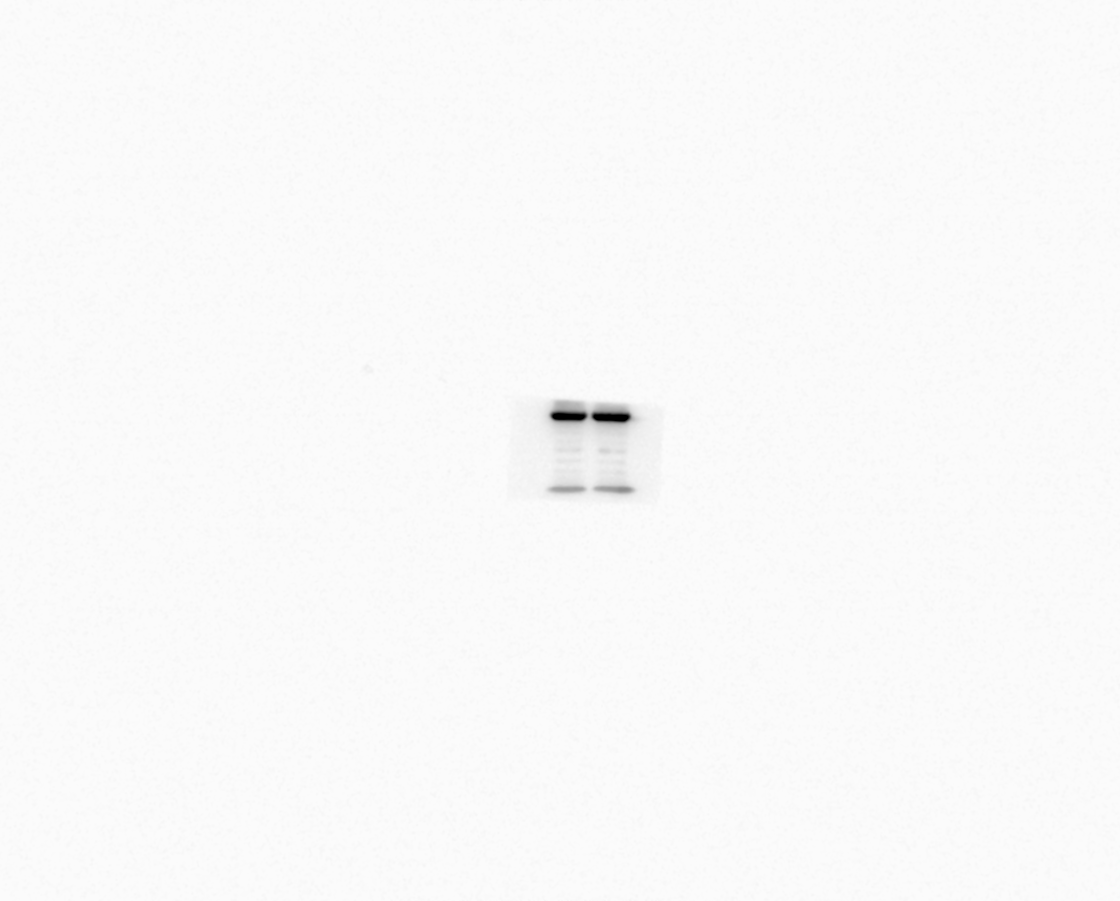

Supplement: Figure 7—source data 1. [file elife-81639-fig7-data1.zip › Figure7-source data/Figure 7E Repeat2/WCE-antiRPA32.tif]

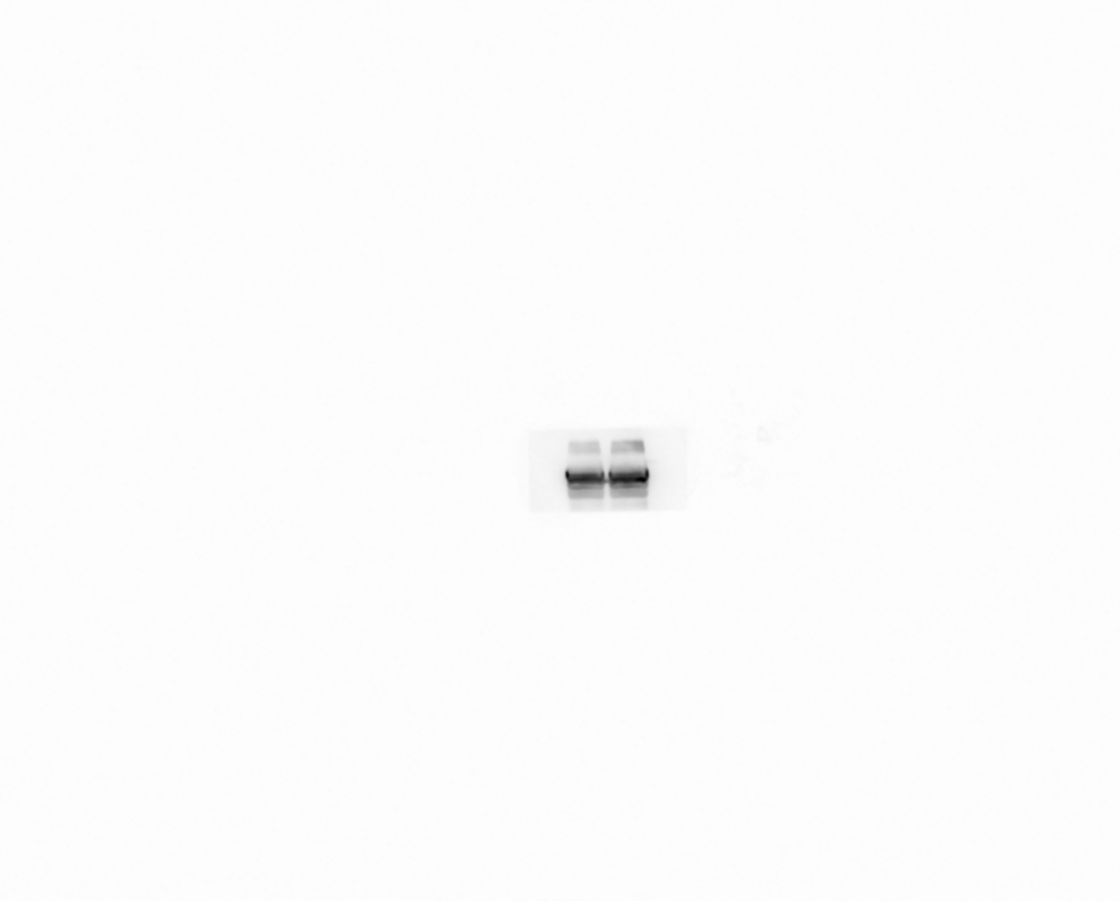

Supplement: Figure 7—source data 1. [file elife-81639-fig7-data1.zip › Figure7-source data/Figure 7E Repeat2/WCE-antiGFP.tif]

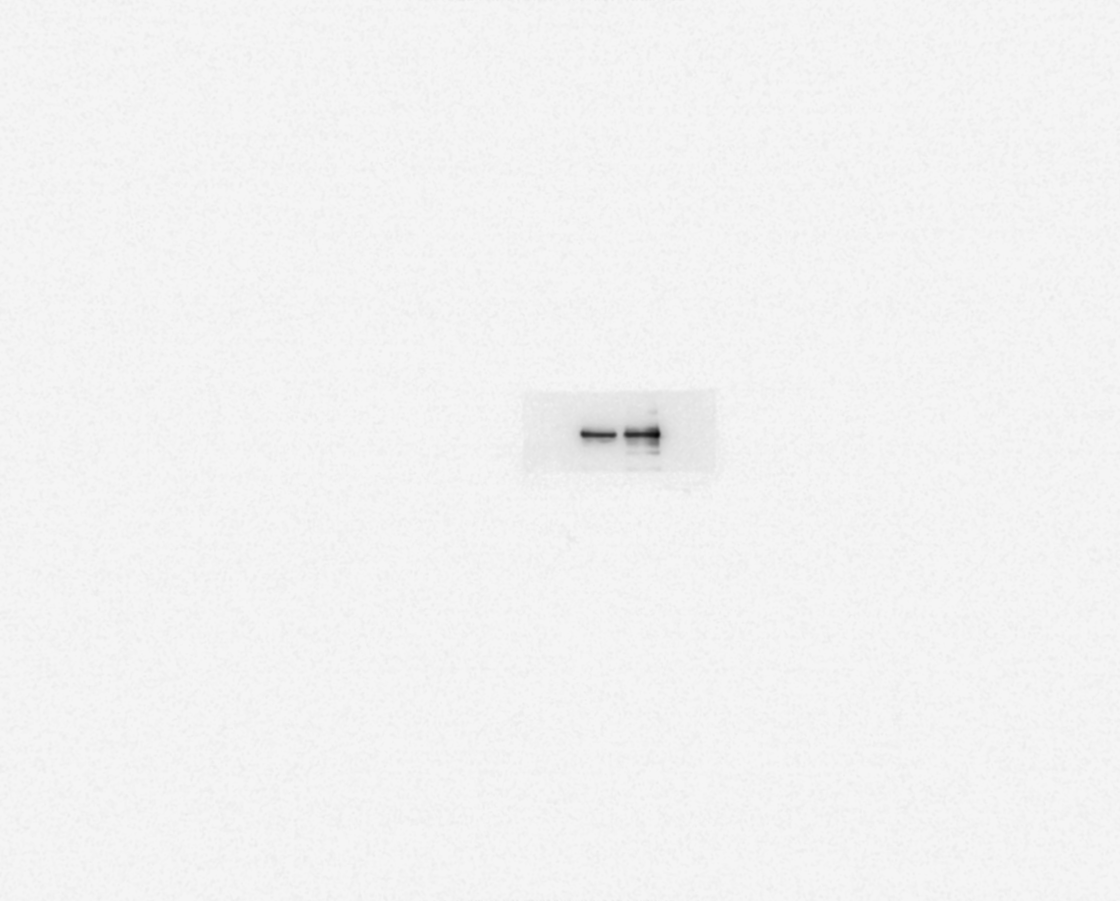

Supplement: Figure 7—source data 1. [file elife-81639-fig7-data1.zip › Figure7-source data/Figure 7E Repeat2/IP-antiGFP.tif]

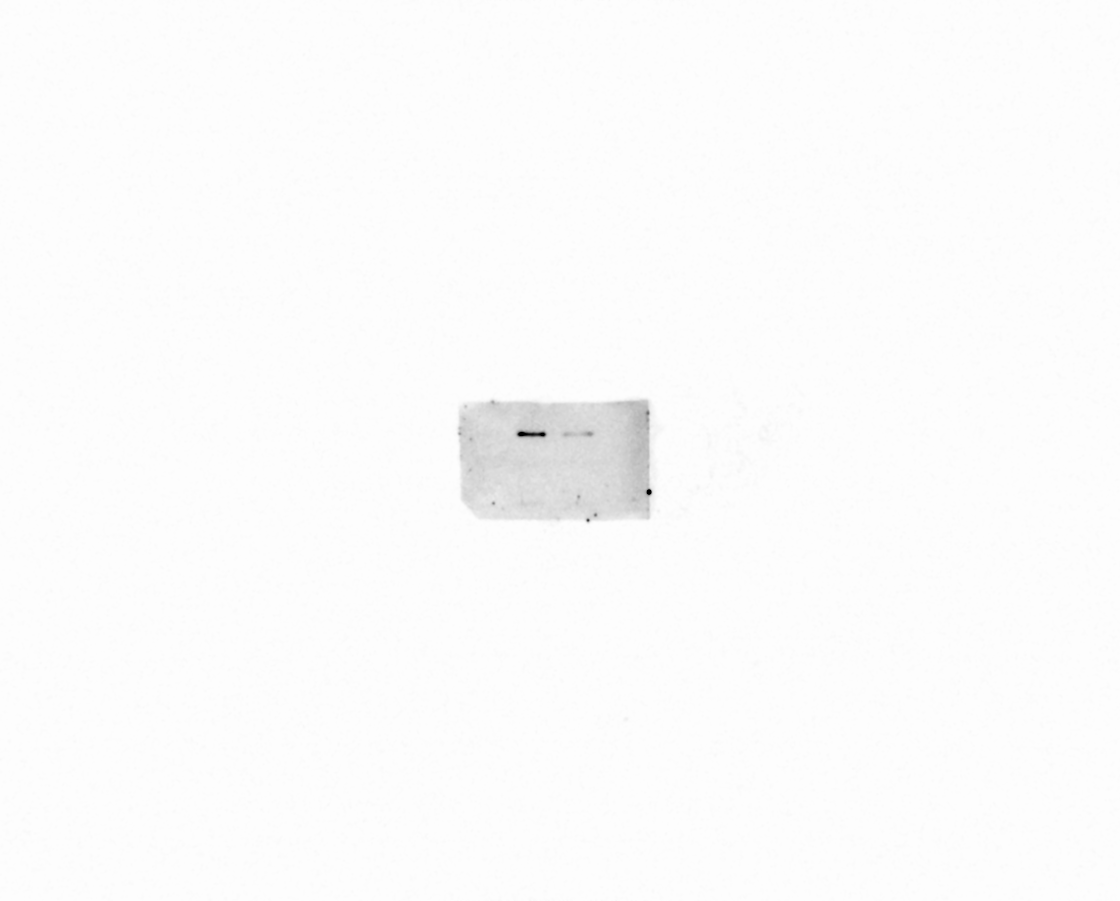

Supplement: Figure 7—source data 1. [file elife-81639-fig7-data1.zip › Figure7-source data/Figure 7E Repeat2/IP-antiRPA32.tif]

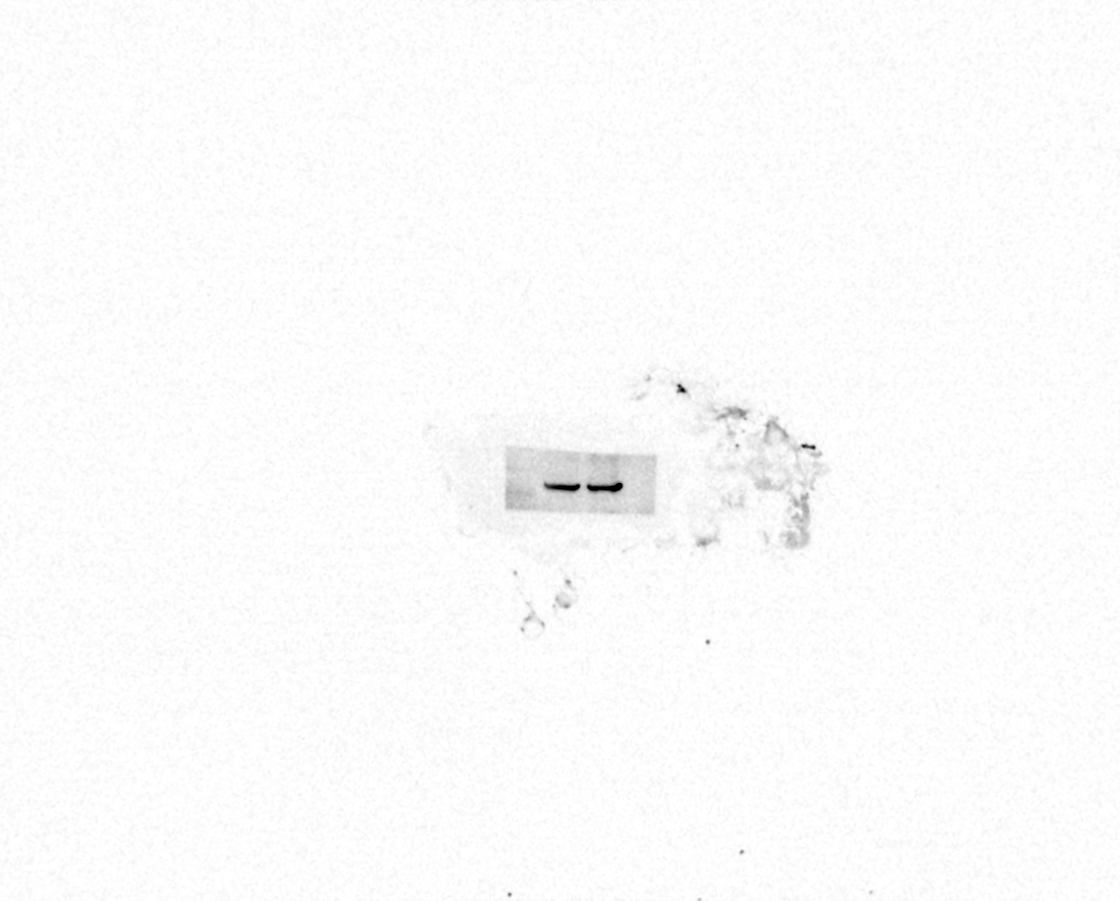

Supplement: Figure 7—source data 1. [file elife-81639-fig7-data1.zip › Figure7-source data/Figure 7E initial trial/WCE-anti╬▓ actin.tif]

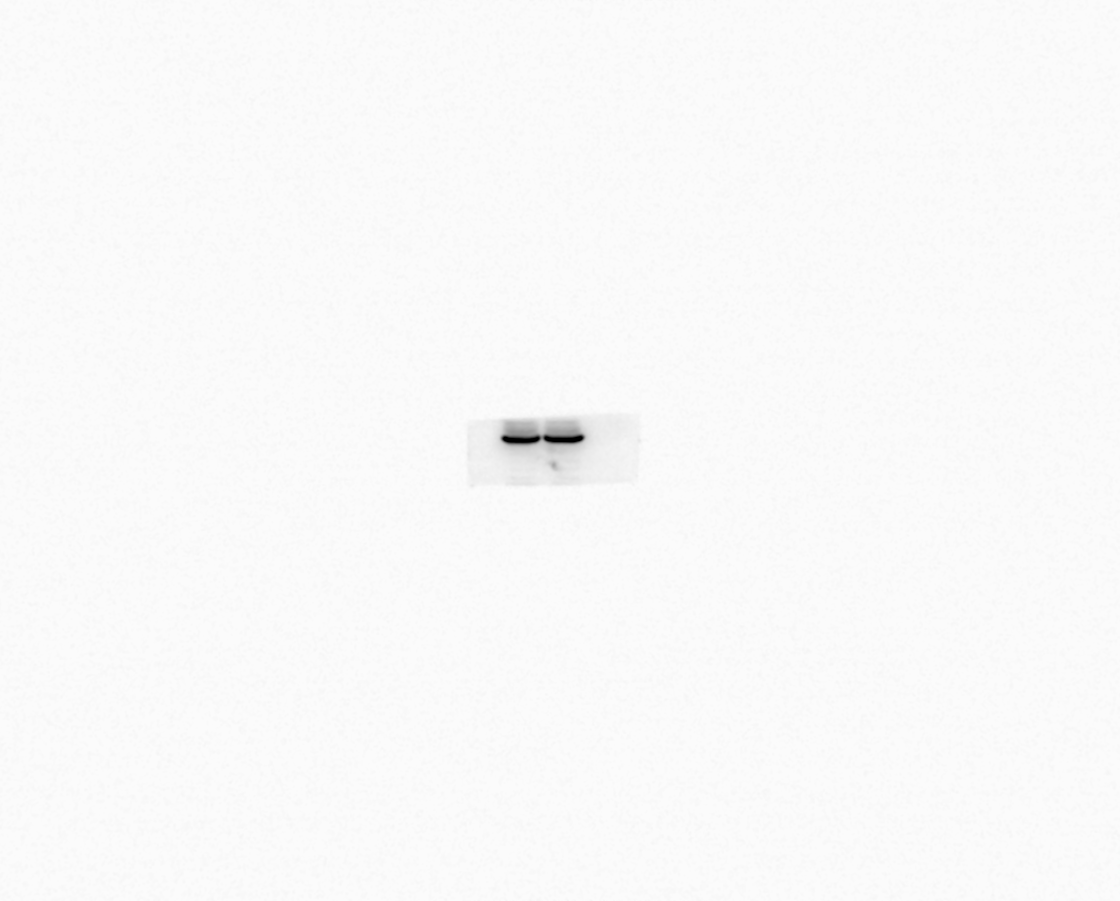

Supplement: Figure 7—source data 1. [file elife-81639-fig7-data1.zip › Figure7-source data/Figure 7E initial trial/WCE-antiRPA32.tif]

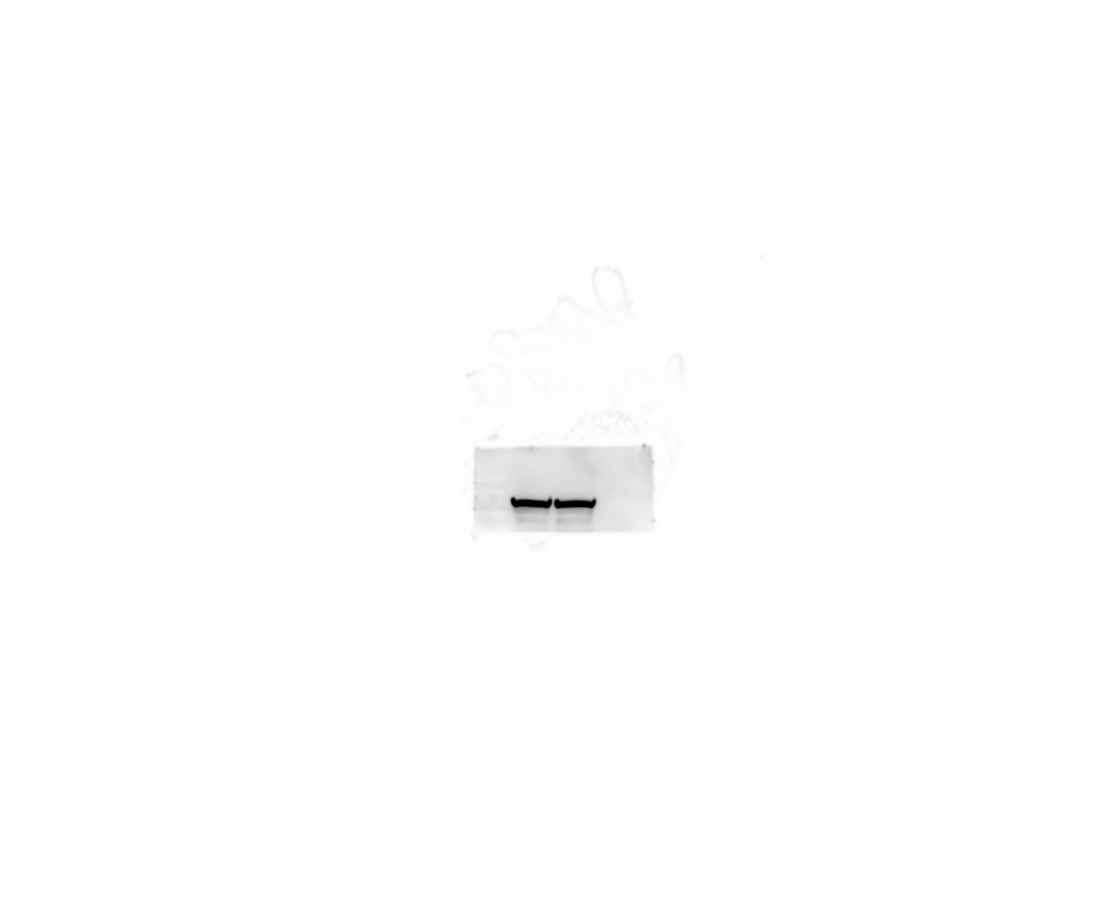

Supplement: Figure 7—source data 1. [file elife-81639-fig7-data1.zip › Figure7-source data/Figure 7E initial trial/WCE-antiGFP.tif]

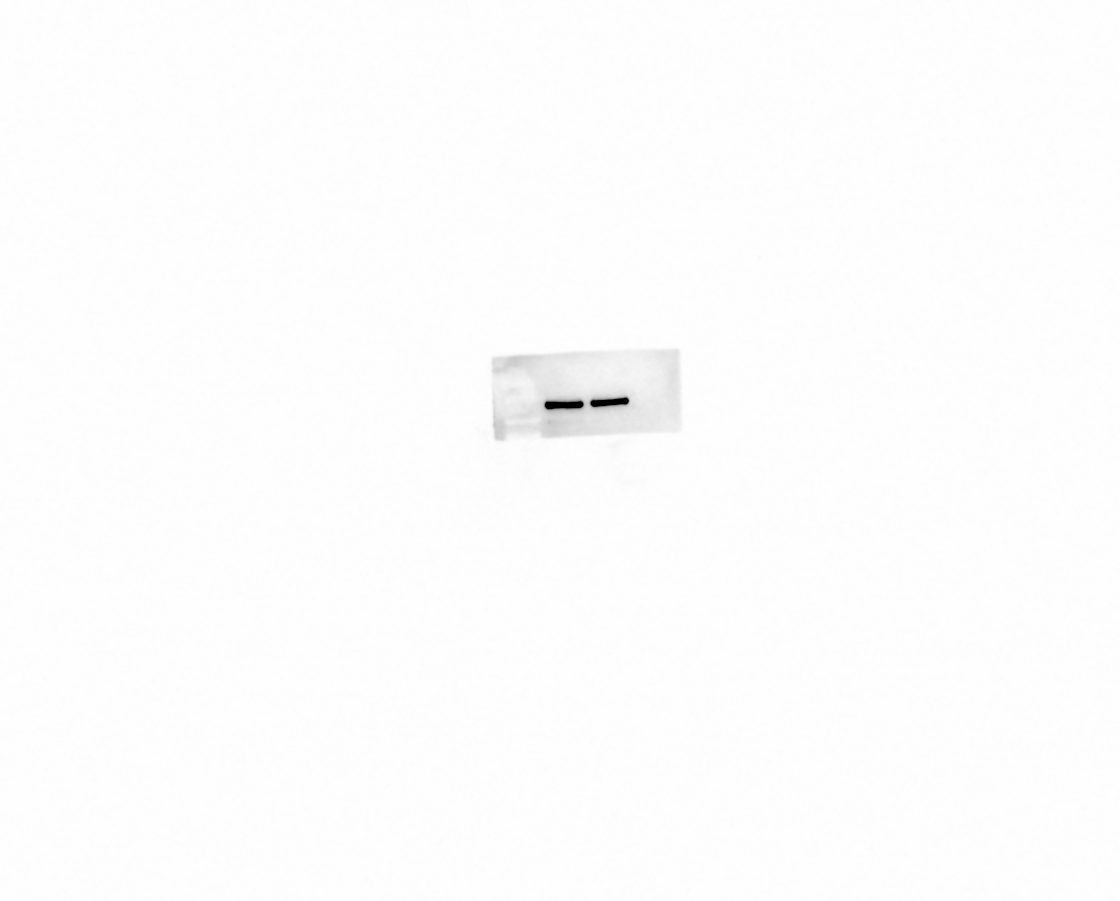

Supplement: Figure 7—source data 1. [file elife-81639-fig7-data1.zip › Figure7-source data/Figure 7E initial trial/IP-antiGFP.tif]

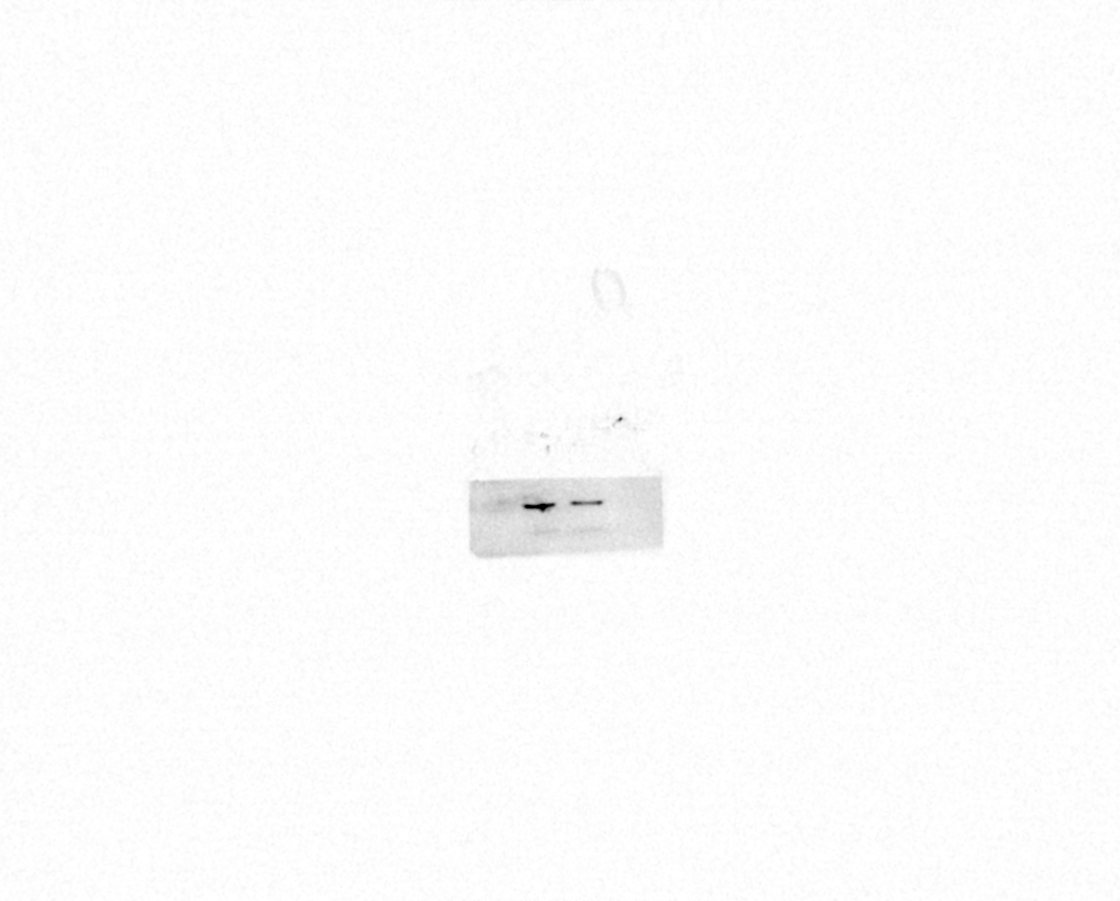

Supplement: Figure 7—source data 1. [file elife-81639-fig7-data1.zip › Figure7-source data/Figure 7E initial trial/IP-antiRPA32.tif]

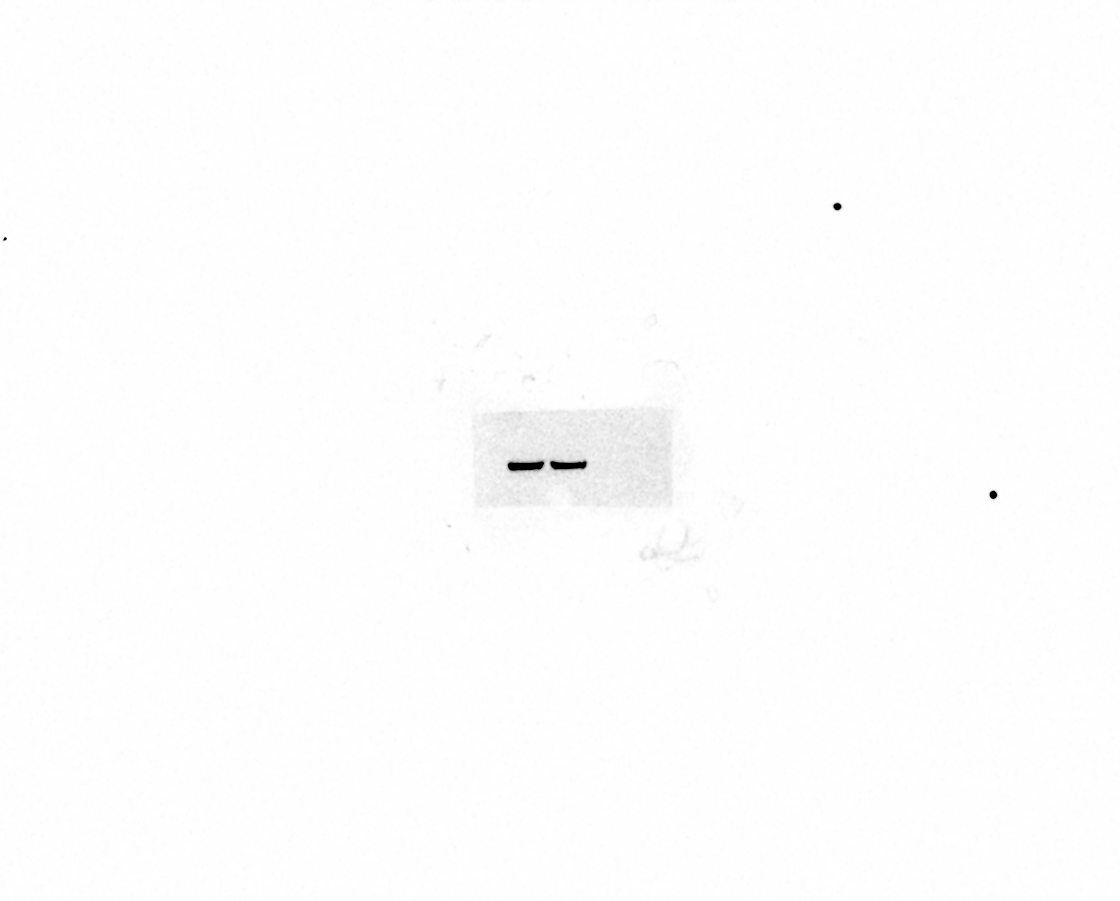

Supplement: Figure 7—source data 1. [file elife-81639-fig7-data1.zip › Figure7-source data/Figure 7E Repeat1/WCE-anti╬▓ actin.tif]

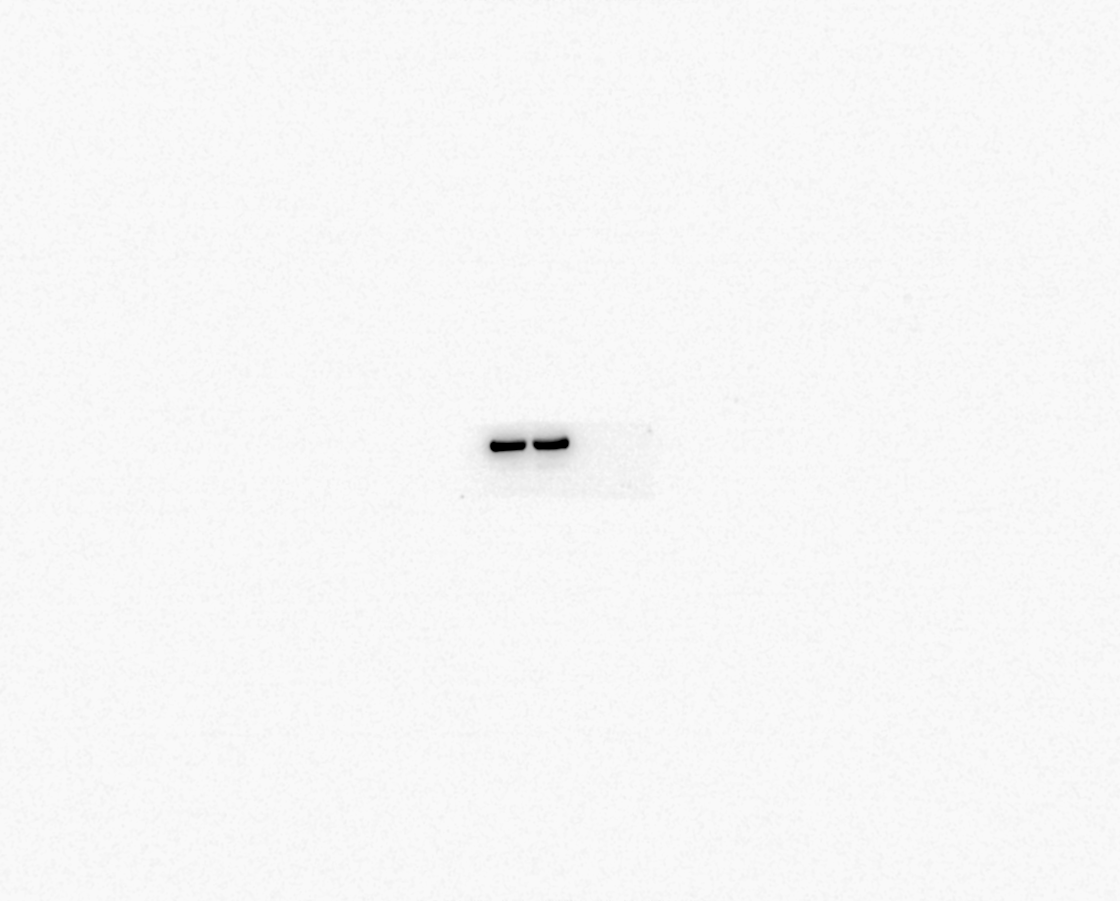

Supplement: Figure 7—source data 1. [file elife-81639-fig7-data1.zip › Figure7-source data/Figure 7E Repeat1/WCE-antiRPA32.tif]

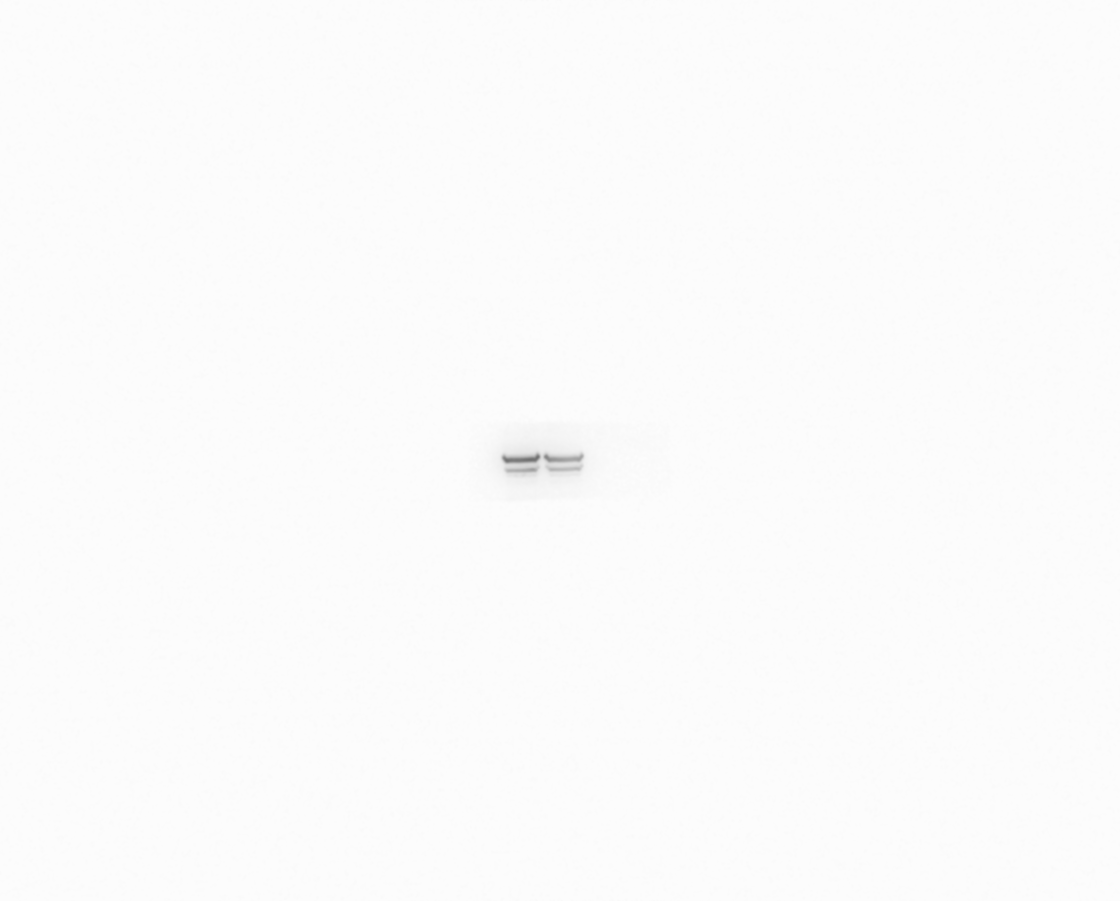

Supplement: Figure 7—source data 1. [file elife-81639-fig7-data1.zip › Figure7-source data/Figure 7E Repeat1/WCE-antiGFP.tif]
