# Supplementary figures and images for: Structural characterization of human RPA70N association with DNA damage response proteins (part 2 of 2)
Source: eLife. 2023 Sep 5;12:e81639. doi: 10.7554/eLife.81639 (PMC10479964; doi:10.7554/eLife.81639)

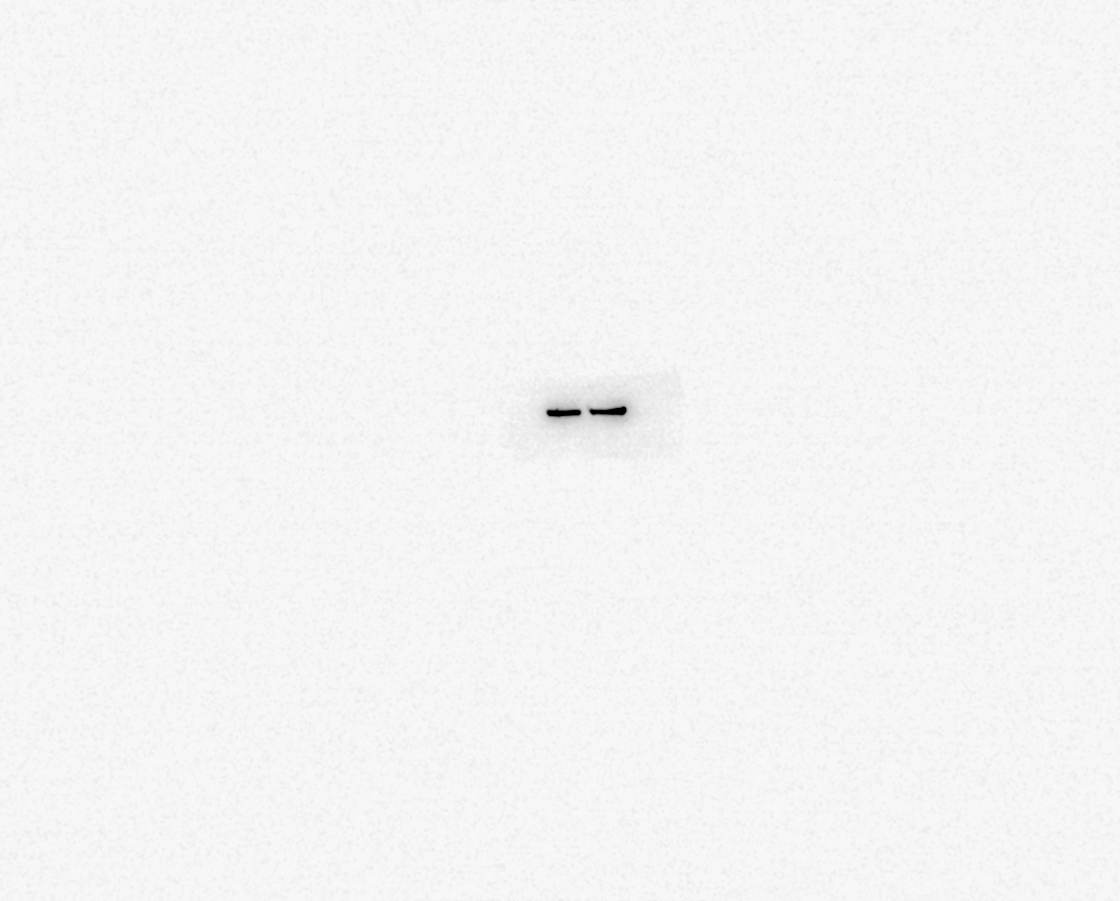

Supplement: Figure 7—source data 1. [file elife-81639-fig7-data1.zip › Figure7-source data/Figure 7E Repeat1/IP-antiGFP.tif]

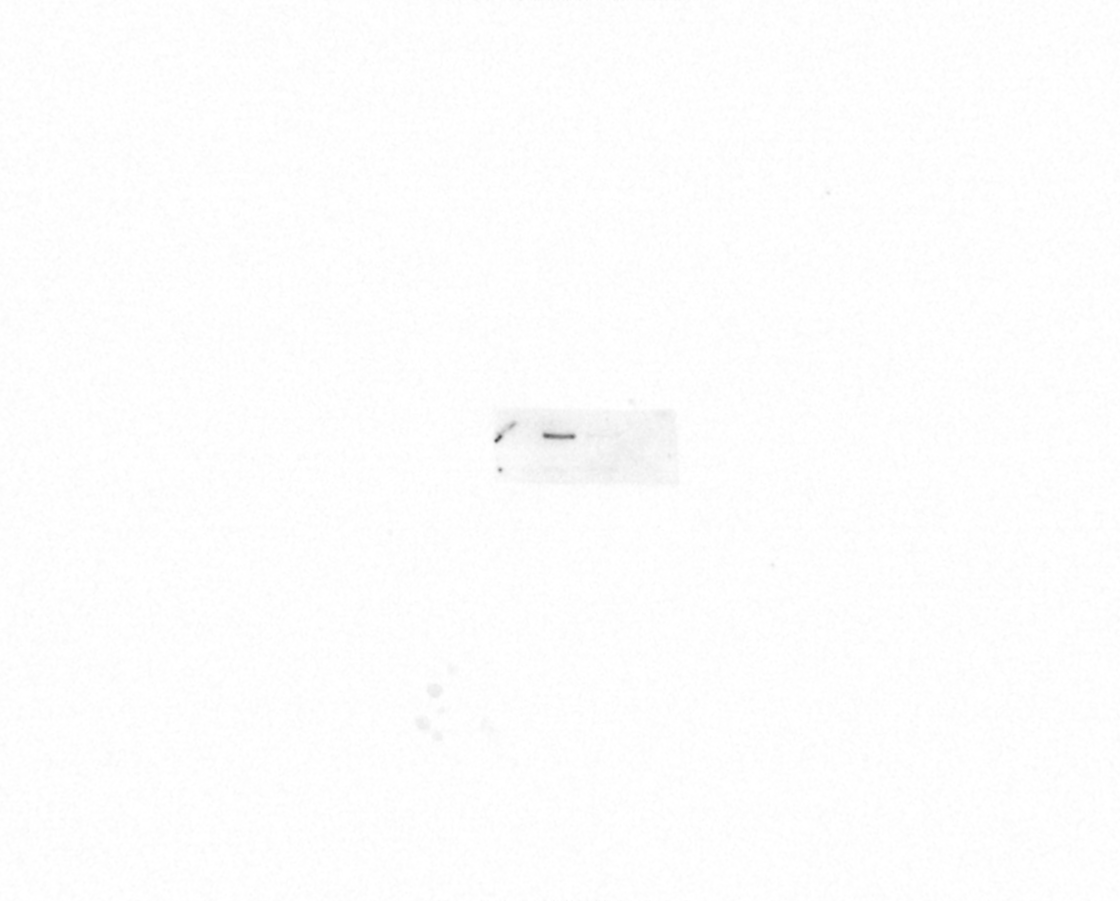

Supplement: Figure 7—source data 1. [file elife-81639-fig7-data1.zip › Figure7-source data/Figure 7E Repeat1/IP-antiRPA32.tif]

Figure 8F  
RAD9

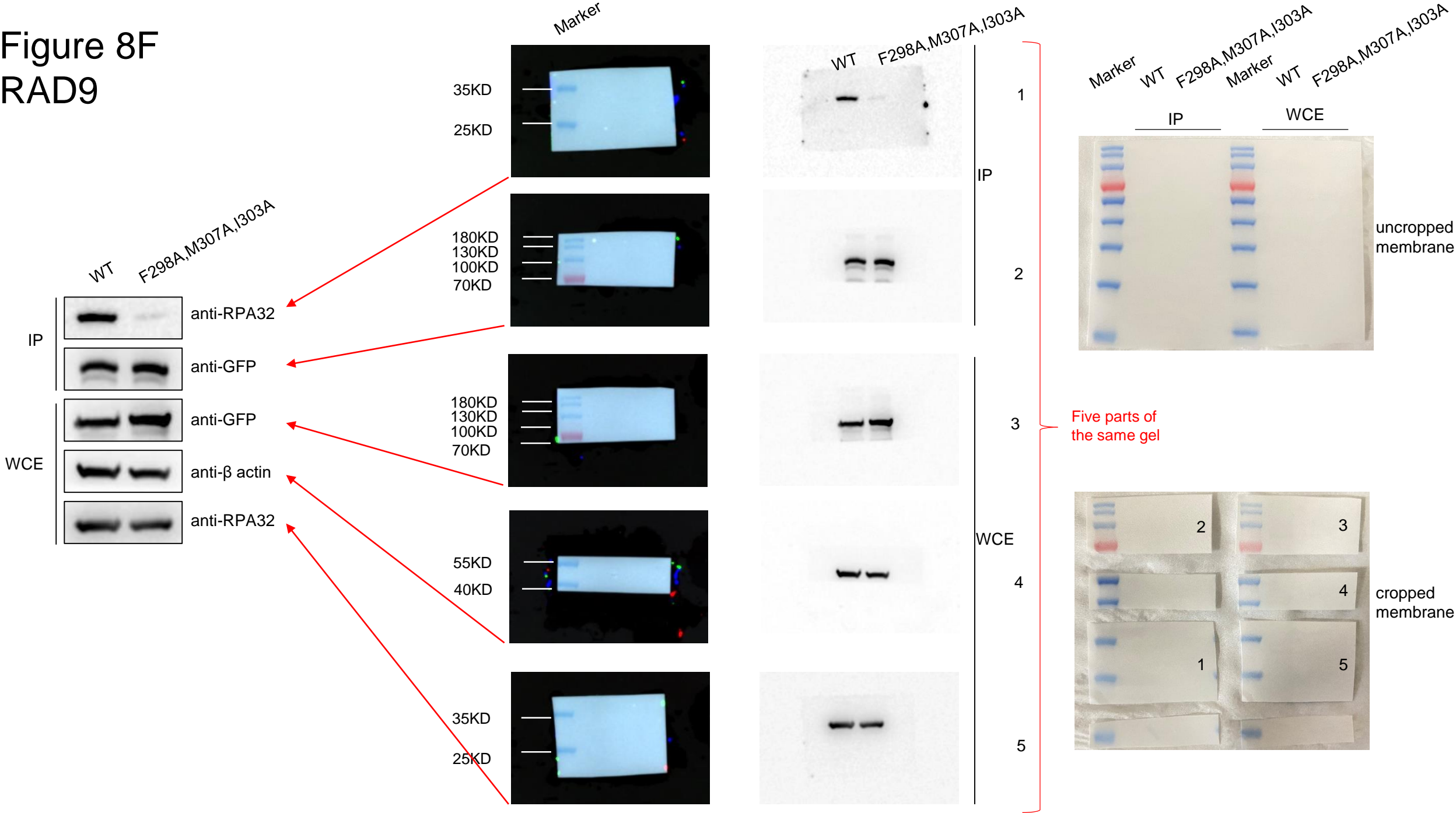

Supplement: Figure 8—source data 1. [file elife-81639-fig8-data1.zip › Figure8-source data/Figure 8F.pdf]

Figure 8F  
RAD9

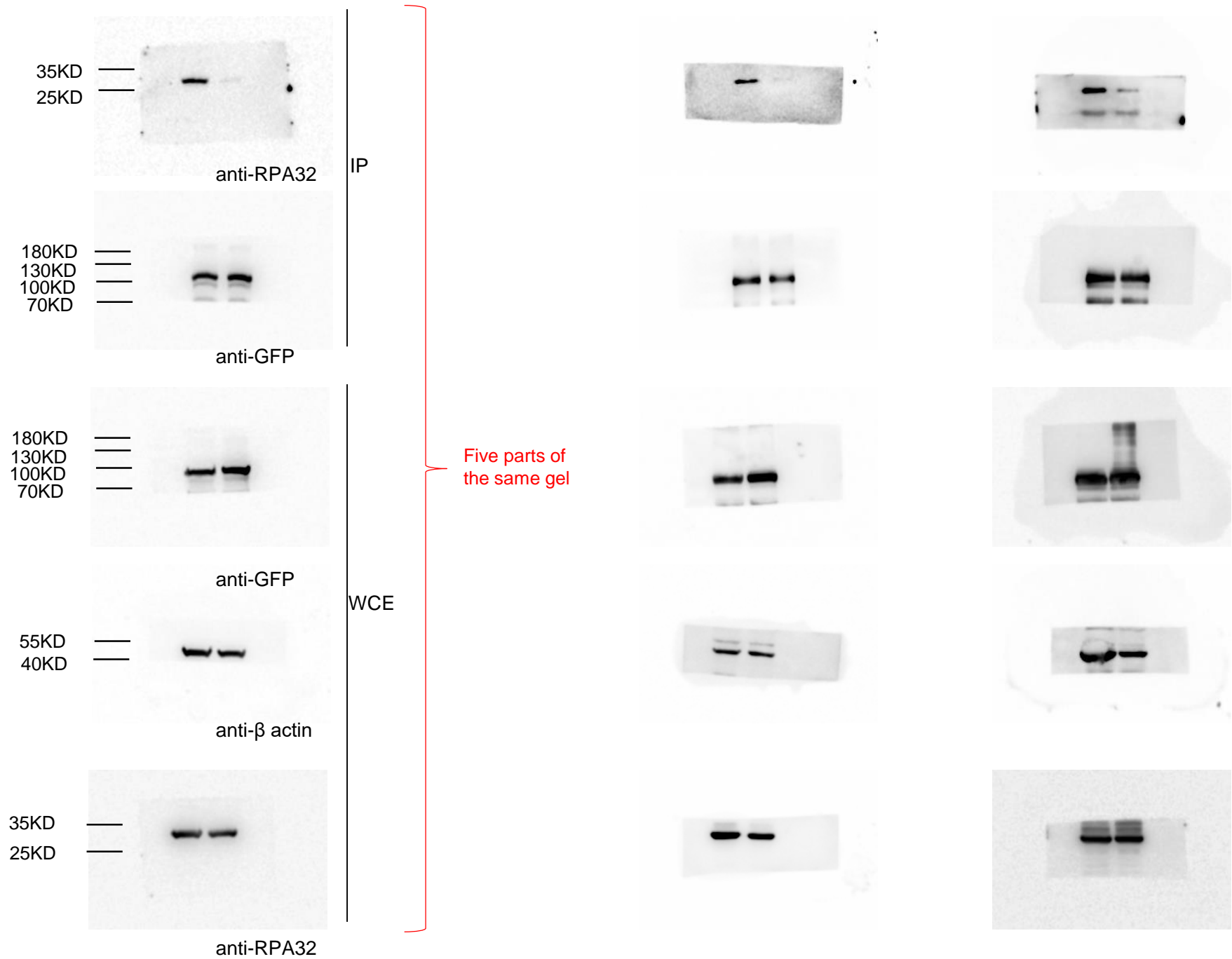

Supplement: Figure 8—source data 1. [file elife-81639-fig8-data1.zip › Figure8-source data/IP-data-Figure 8F.pdf]

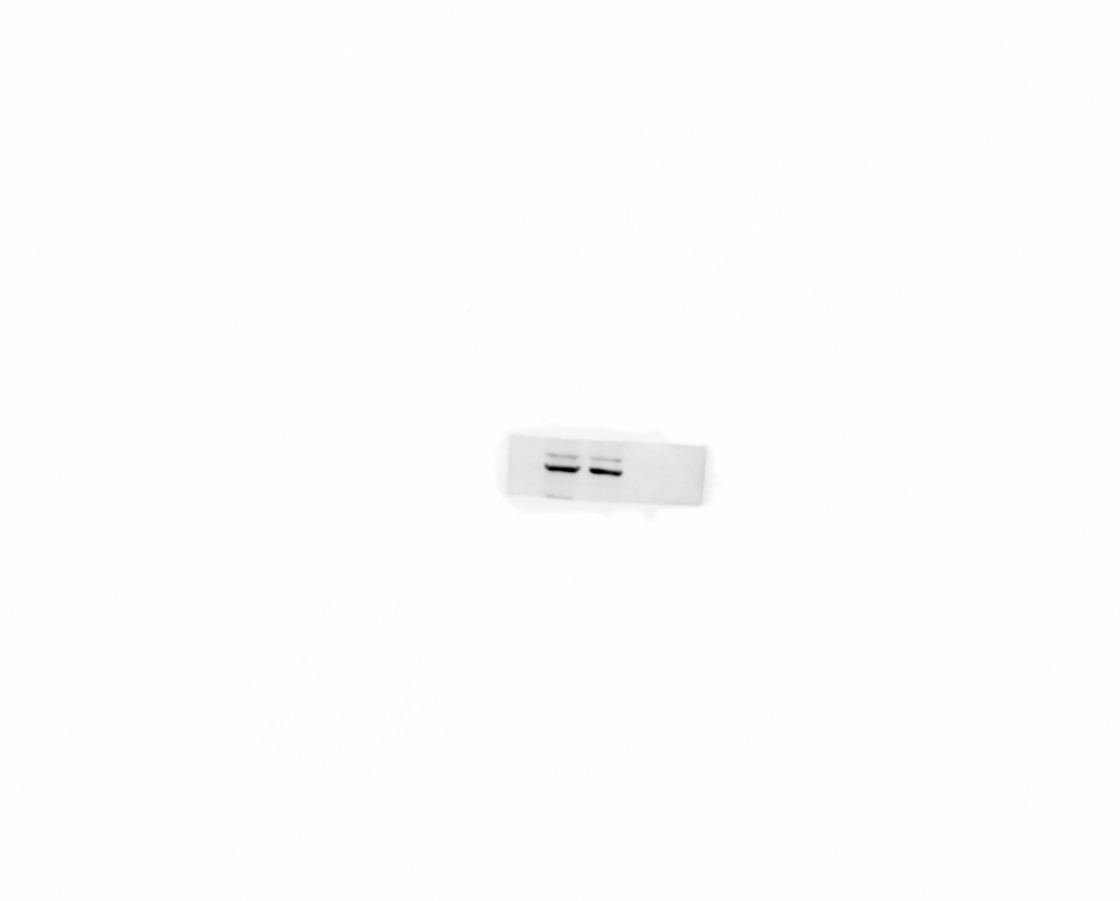

Supplement: Figure 8—source data 1. [file elife-81639-fig8-data1.zip › Figure8-source data/Figure 8F Repeat1/WCE-anti╬▓ actin.tif]

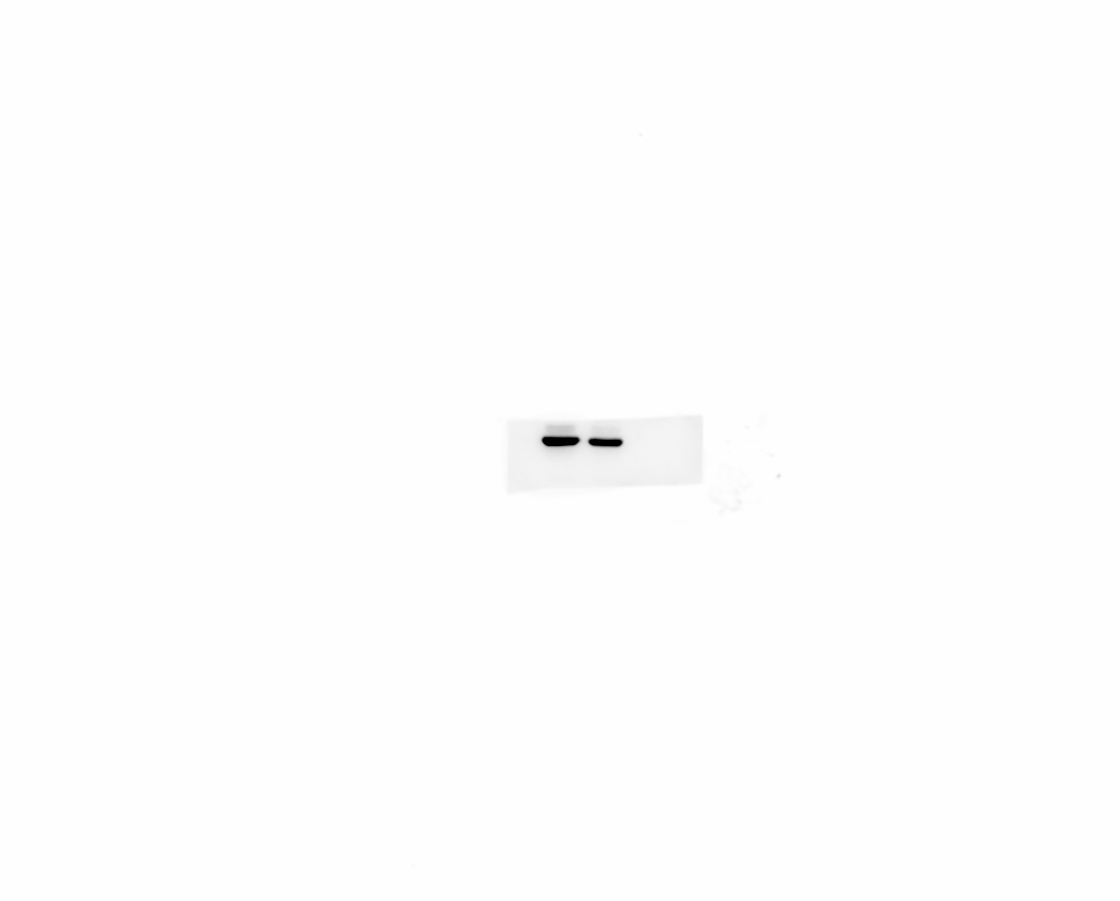

Supplement: Figure 8—source data 1. [file elife-81639-fig8-data1.zip › Figure8-source data/Figure 8F Repeat1/WCE-antiRPA32.tif]

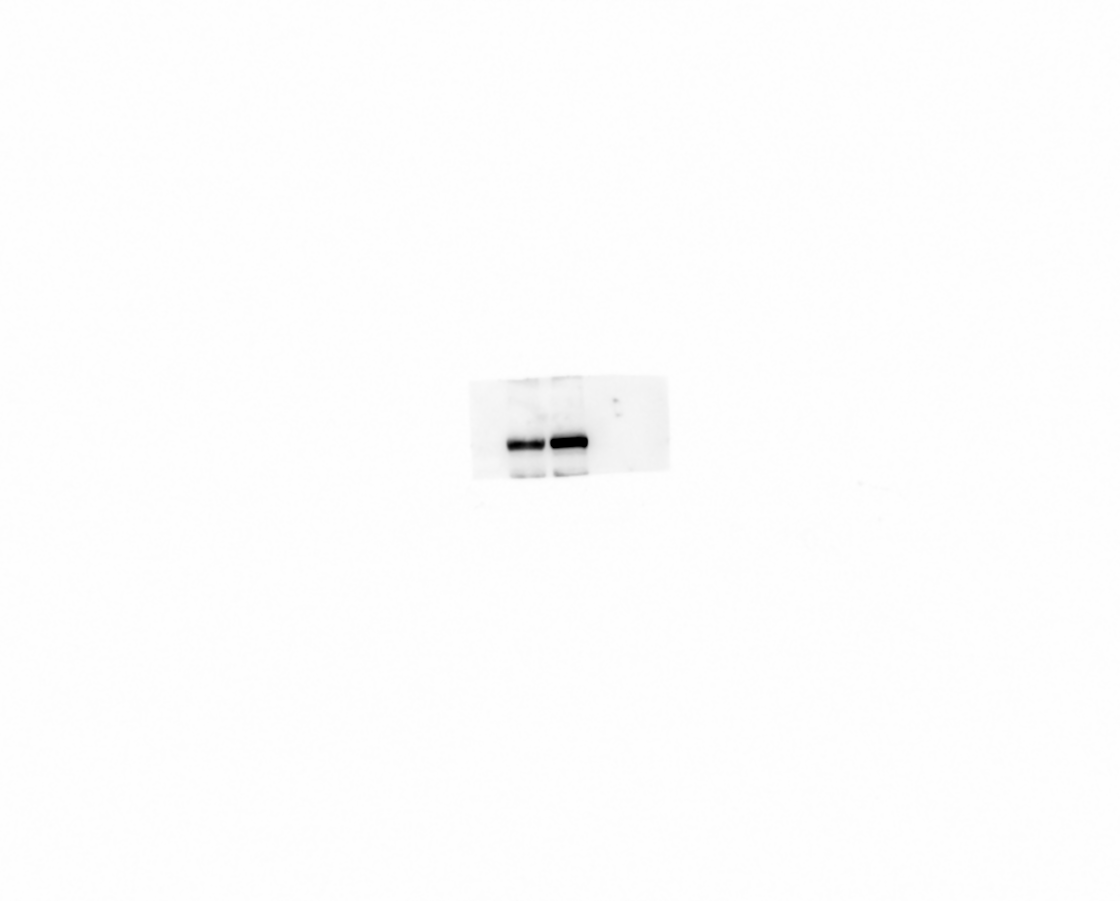

Supplement: Figure 8—source data 1. [file elife-81639-fig8-data1.zip › Figure8-source data/Figure 8F Repeat1/WCE-antiGFP.tif]

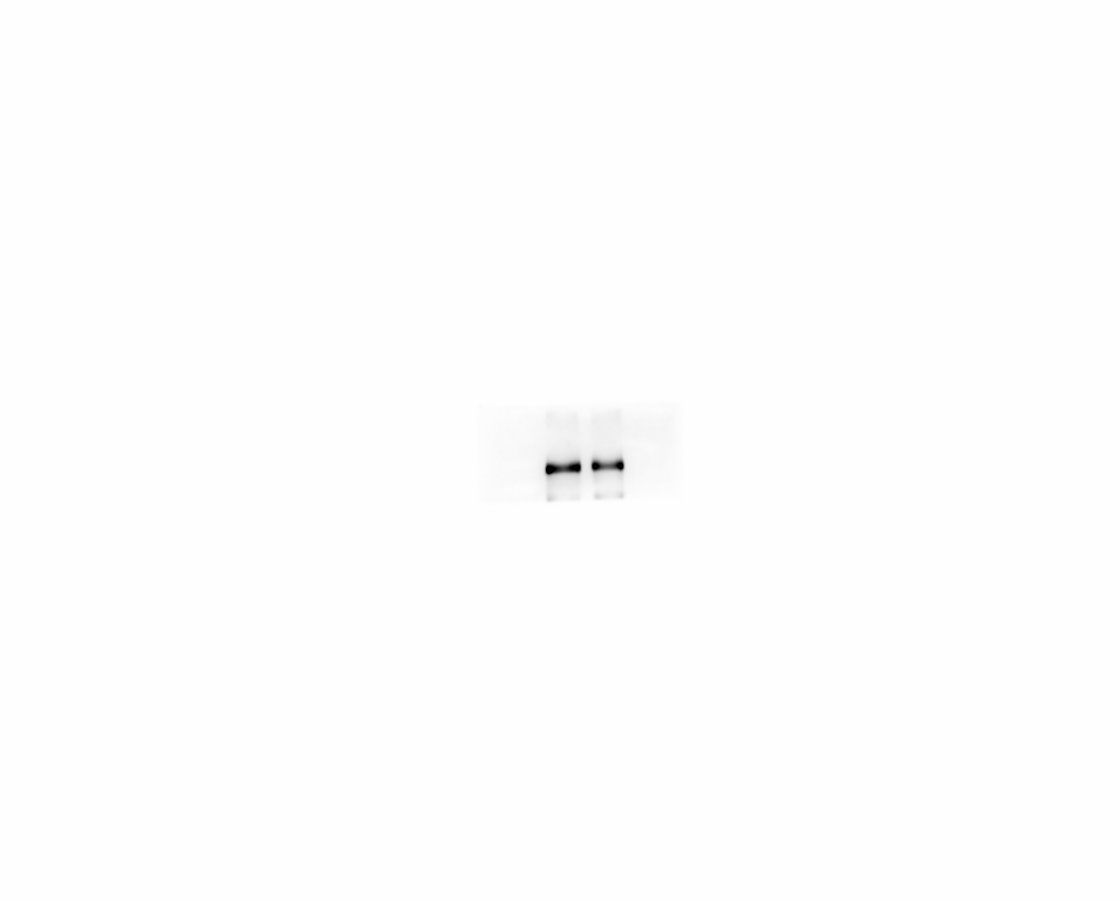

Supplement: Figure 8—source data 1. [file elife-81639-fig8-data1.zip › Figure8-source data/Figure 8F Repeat1/IP-antiGFP.tif]

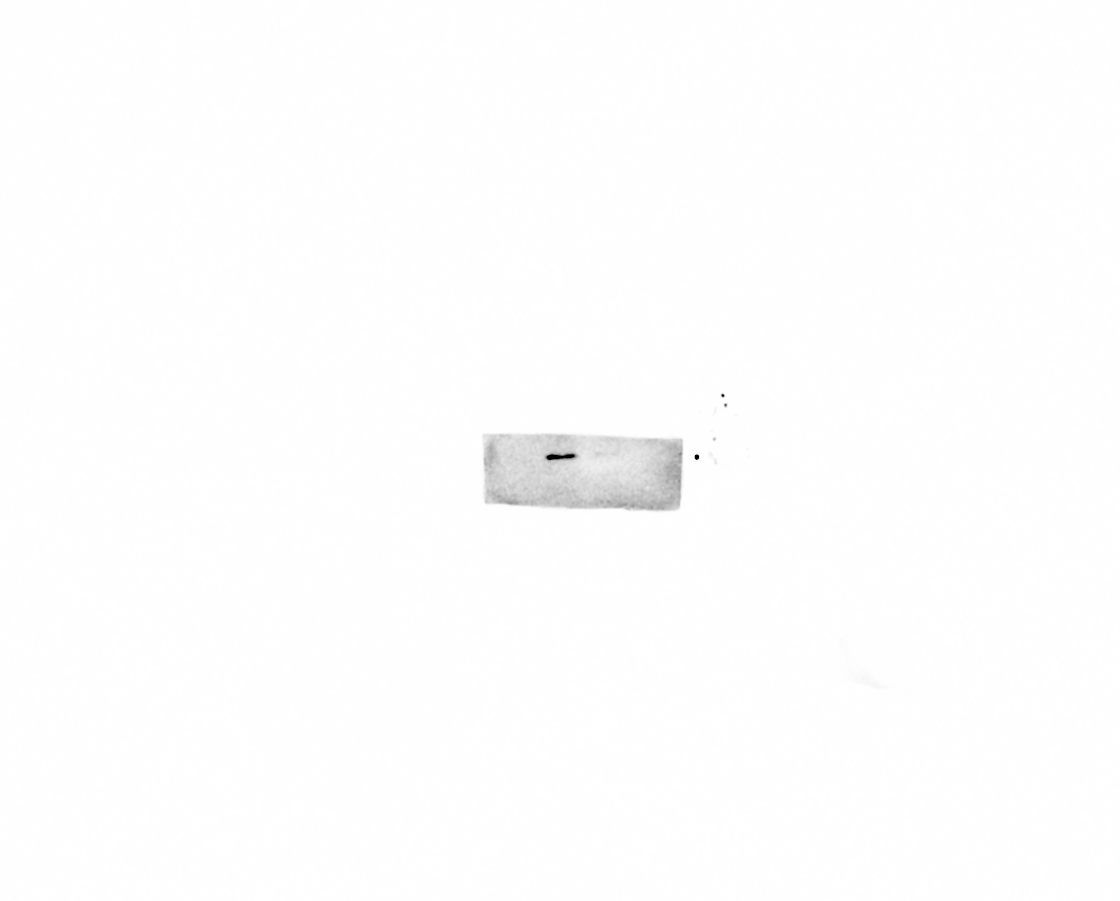

Supplement: Figure 8—source data 1. [file elife-81639-fig8-data1.zip › Figure8-source data/Figure 8F Repeat1/IP-antiRPA32.tif]

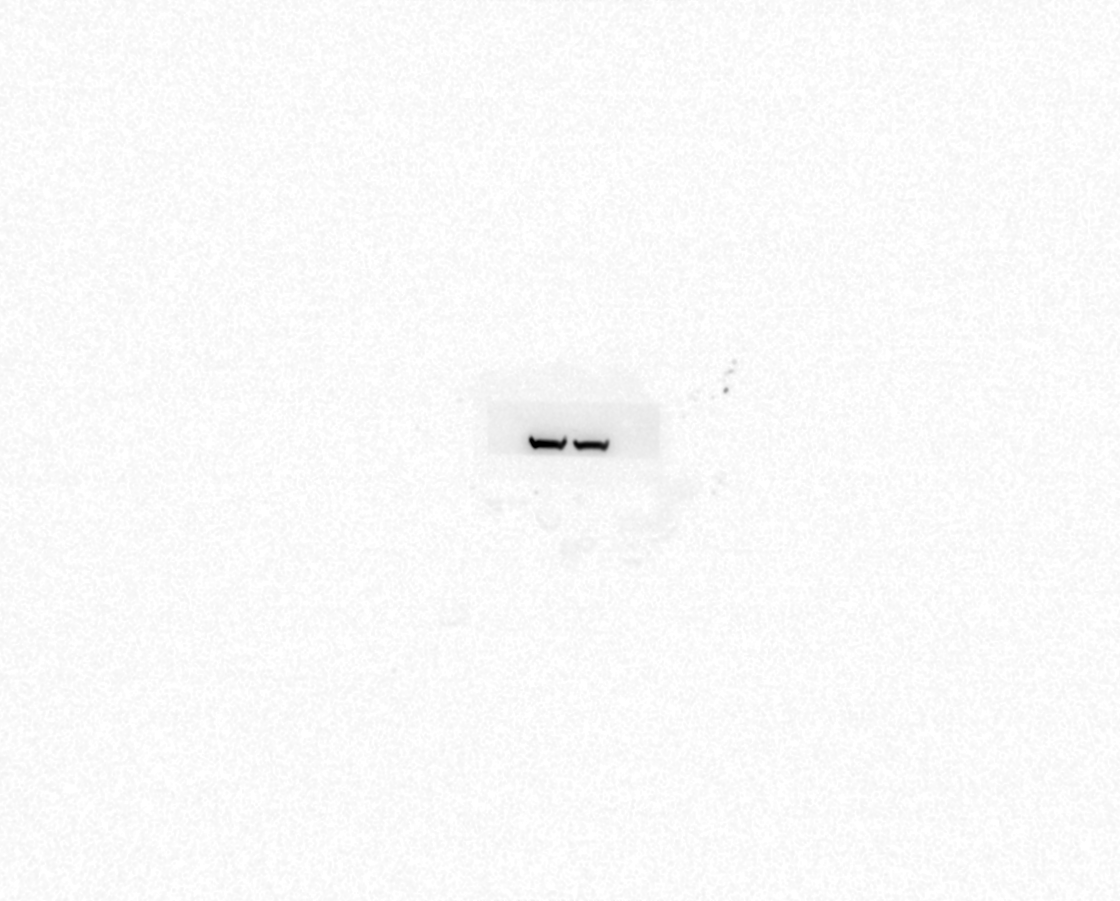

Supplement: Figure 8—source data 1. [file elife-81639-fig8-data1.zip › Figure8-source data/Figure 8F initial trial/WCE-anti╬▓ actin.tif]

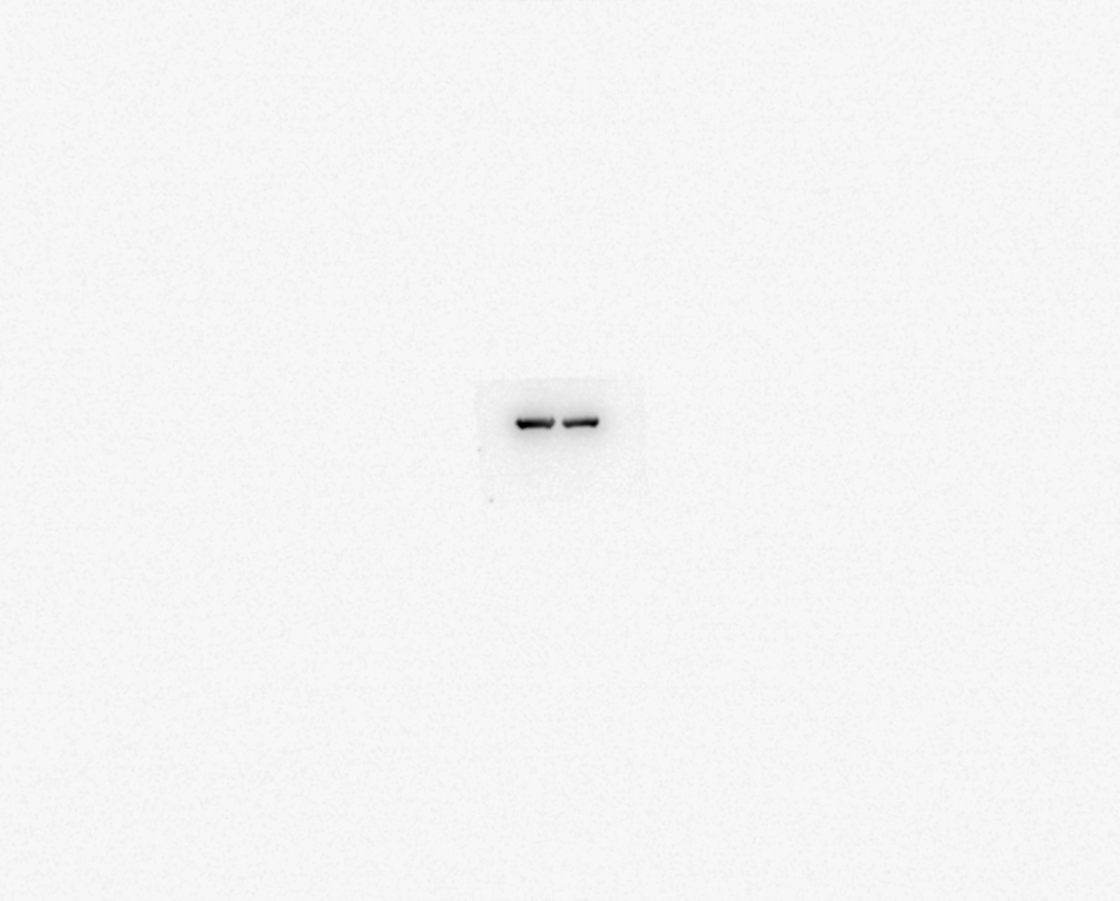

Supplement: Figure 8—source data 1. [file elife-81639-fig8-data1.zip › Figure8-source data/Figure 8F initial trial/WCE-antiRPA32.tif]

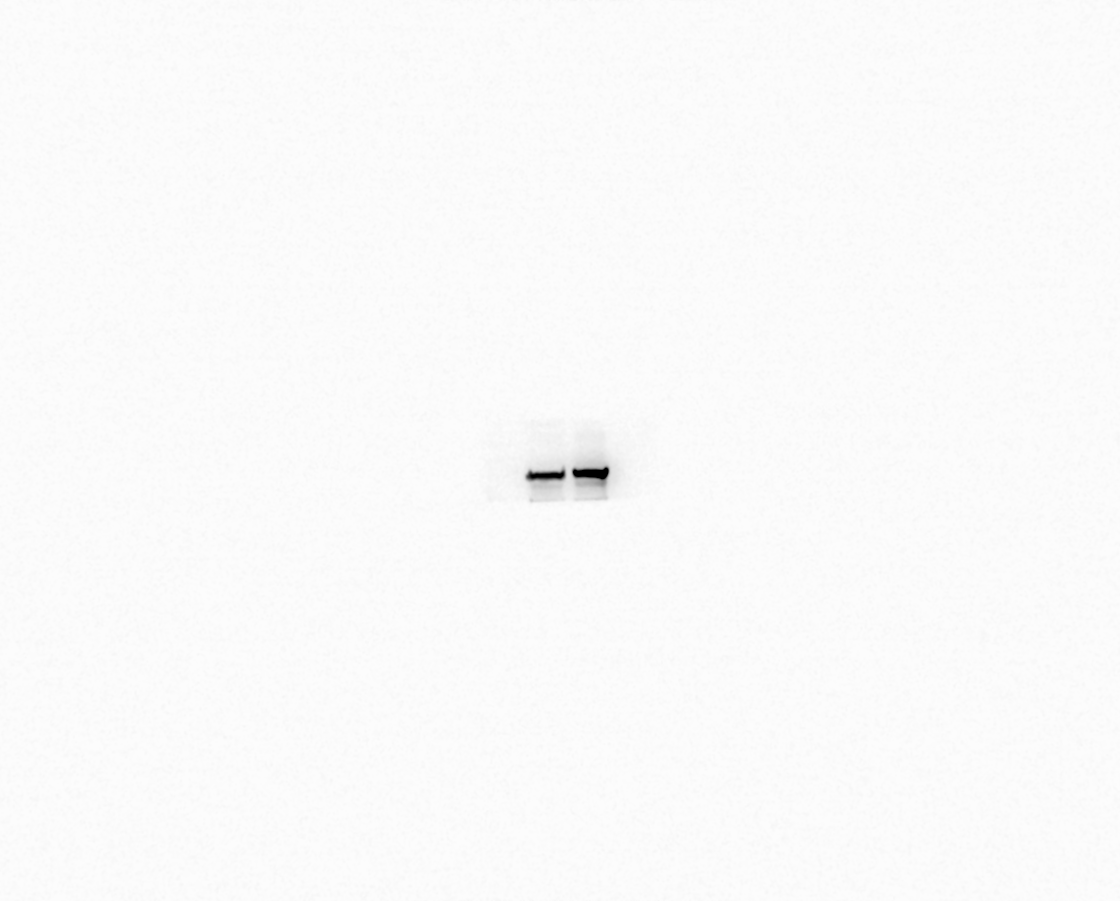

Supplement: Figure 8—source data 1. [file elife-81639-fig8-data1.zip › Figure8-source data/Figure 8F initial trial/WCE-antiGFP.tif]

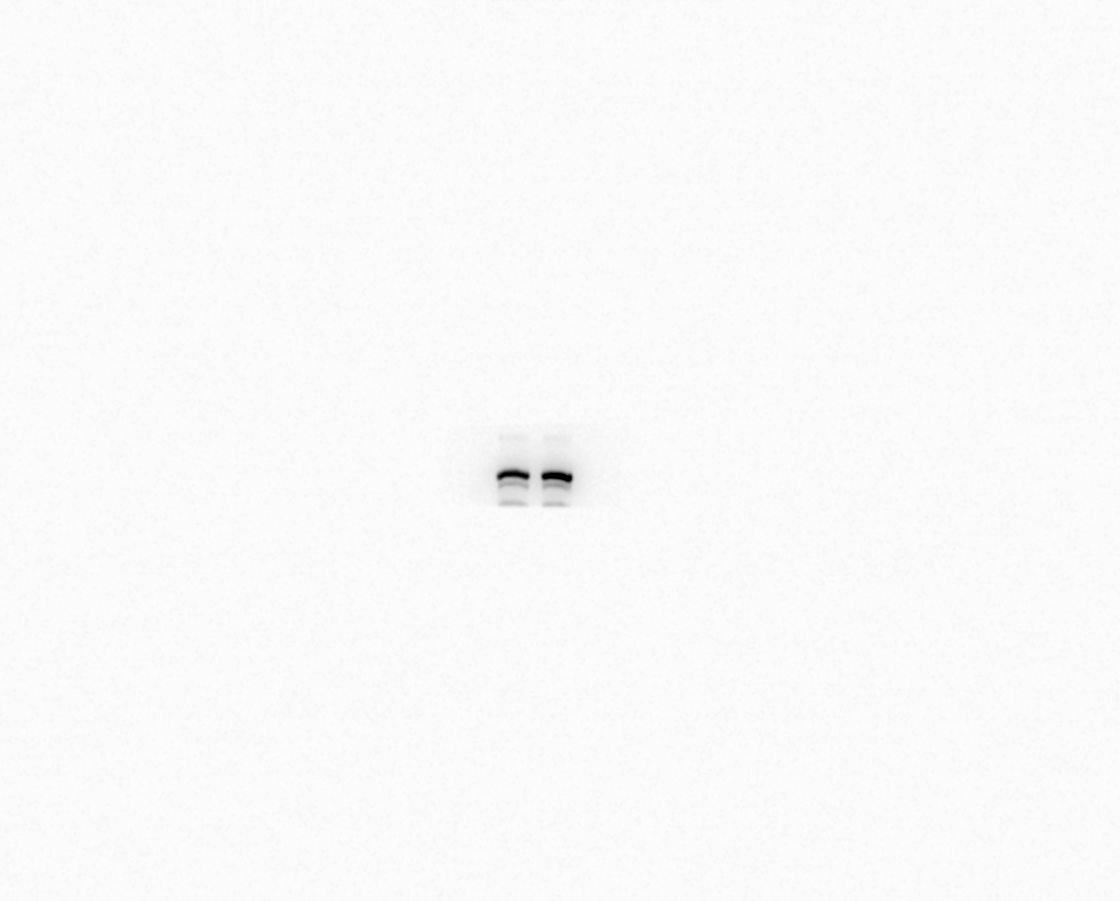

Supplement: Figure 8—source data 1. [file elife-81639-fig8-data1.zip › Figure8-source data/Figure 8F initial trial/IP-antiGFP.tif]

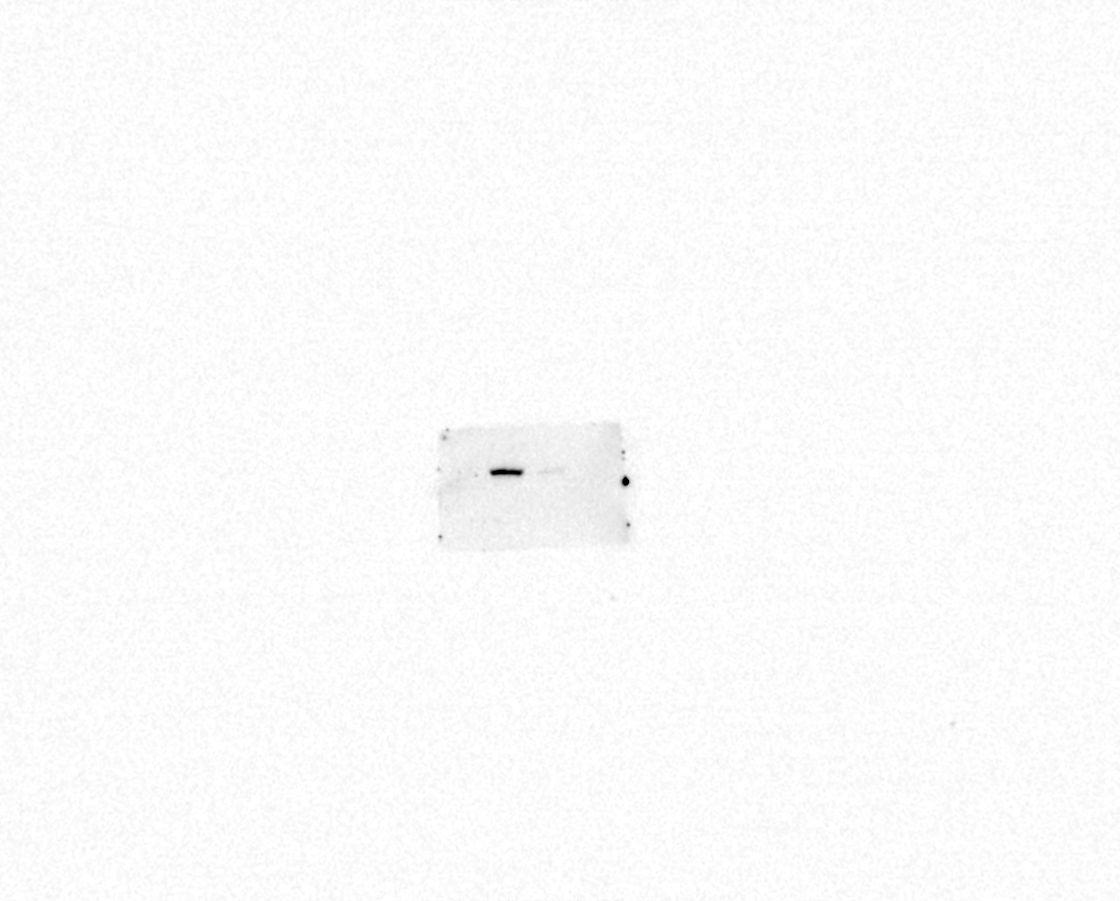

Supplement: Figure 8—source data 1. [file elife-81639-fig8-data1.zip › Figure8-source data/Figure 8F initial trial/IP-antiRPA32.tif]

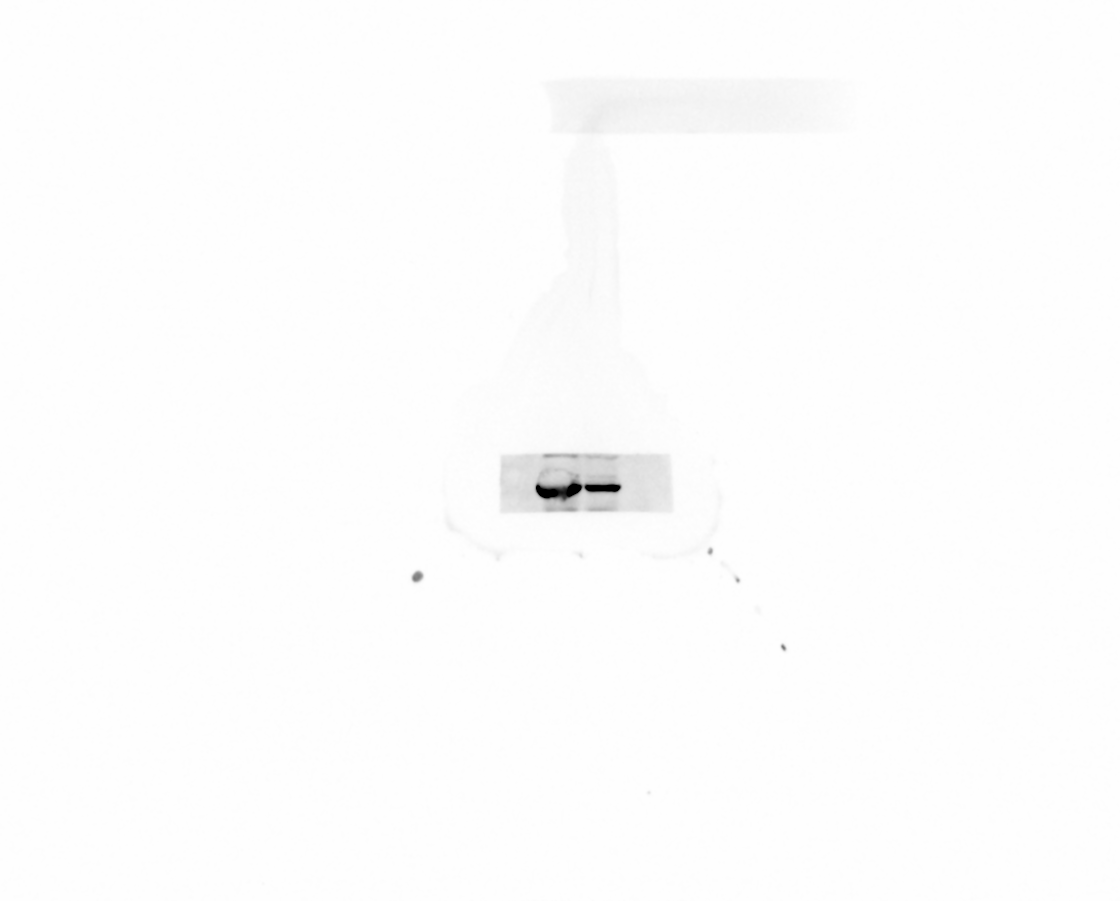

Supplement: Figure 8—source data 1. [file elife-81639-fig8-data1.zip › Figure8-source data/Figure 8F Repeat2/WCE-anti╬▓ actin.tif]

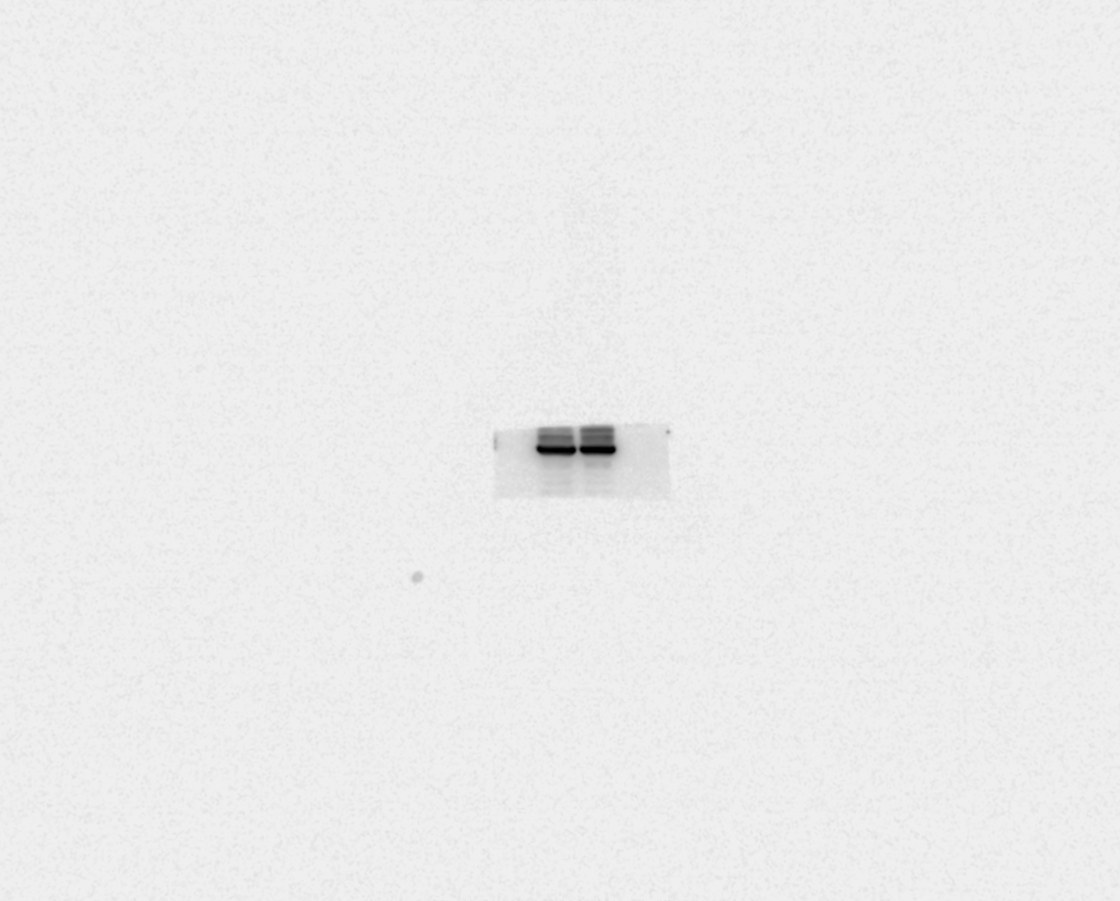

Supplement: Figure 8—source data 1. [file elife-81639-fig8-data1.zip › Figure8-source data/Figure 8F Repeat2/WCE-antiRPA32.tif]

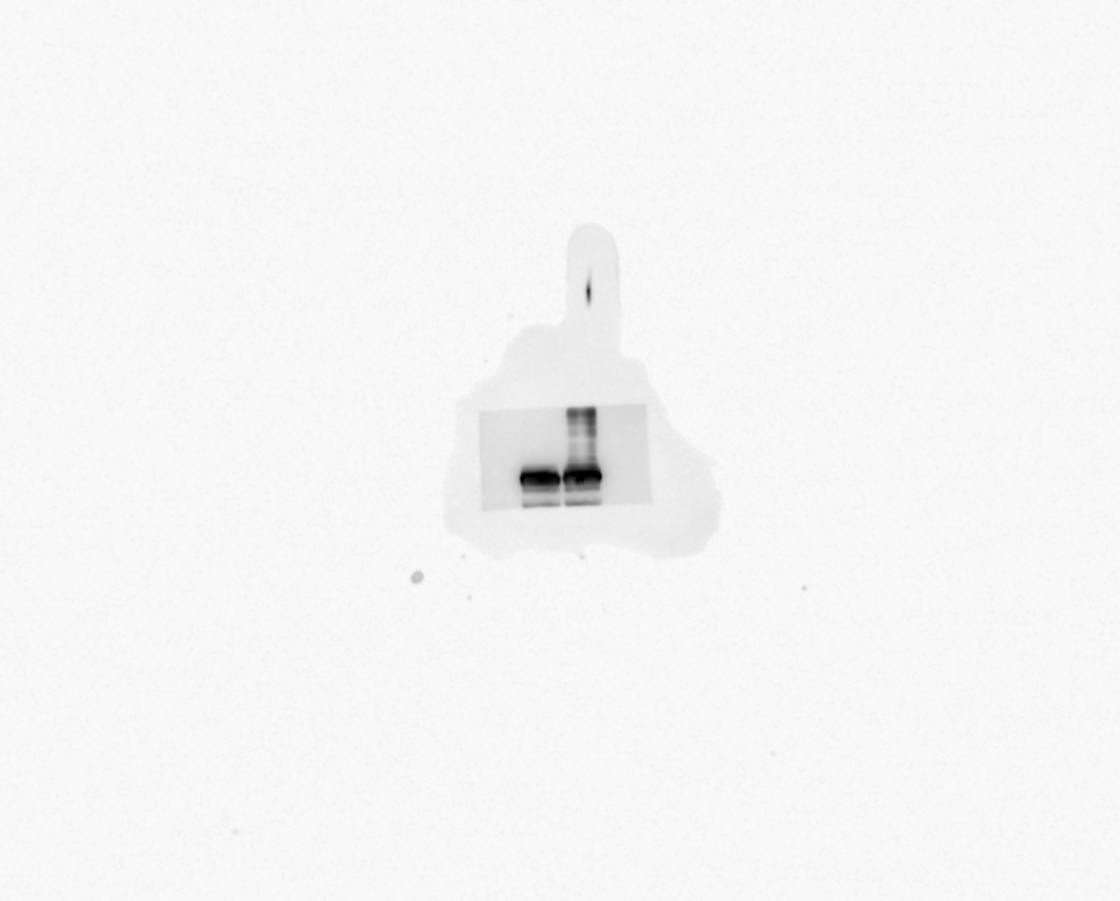

Supplement: Figure 8—source data 1. [file elife-81639-fig8-data1.zip › Figure8-source data/Figure 8F Repeat2/WCE-antiGFP.tif]

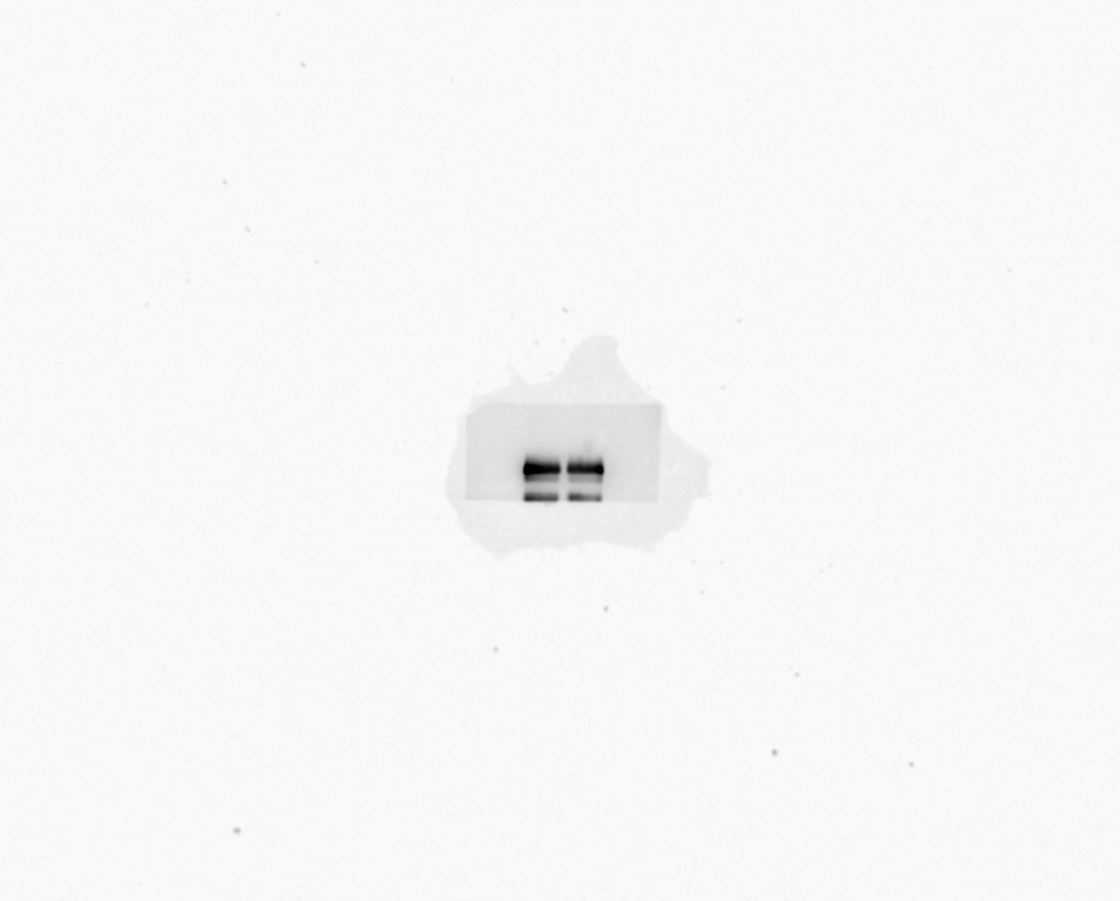

Supplement: Figure 8—source data 1. [file elife-81639-fig8-data1.zip › Figure8-source data/Figure 8F Repeat2/IP-antiGFP.tif]

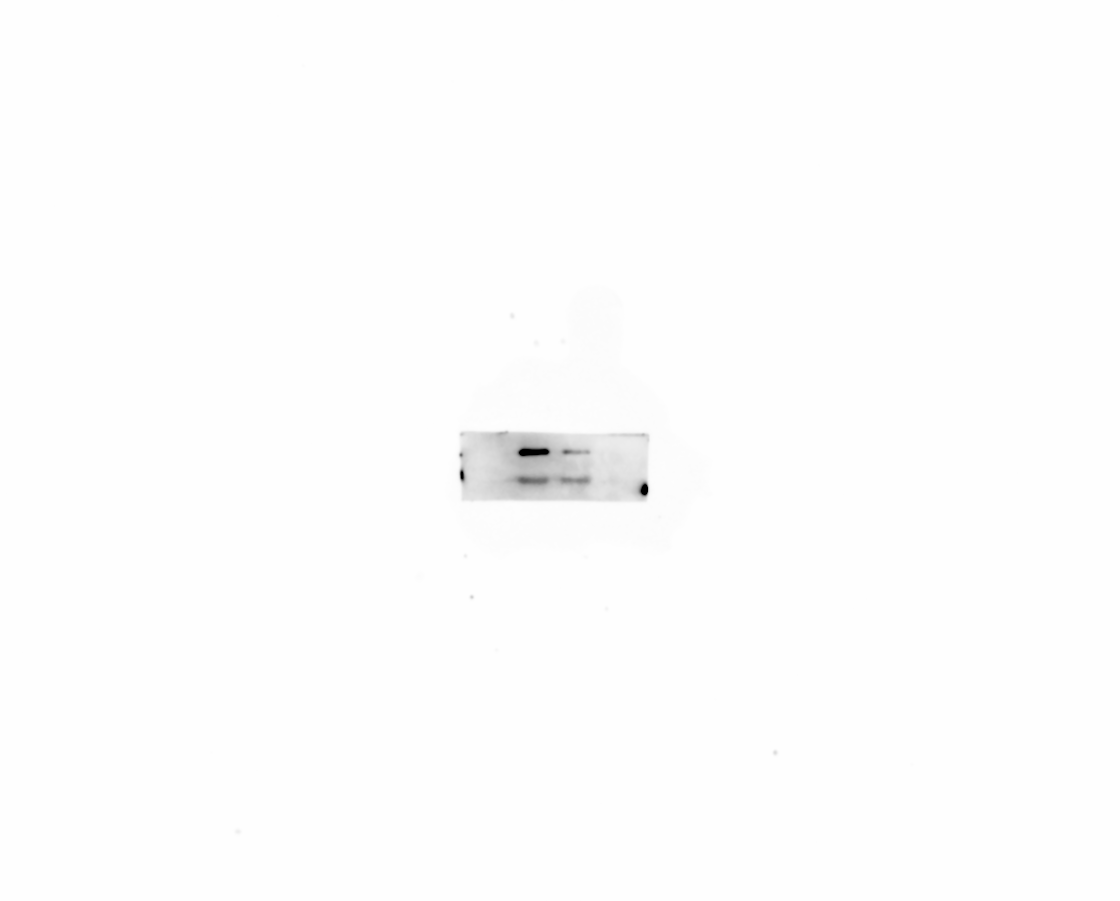

Supplement: Figure 8—source data 1. [file elife-81639-fig8-data1.zip › Figure8-source data/Figure 8F Repeat2/IP-antiRPA32.tif]

Figure 9H  
ETAA1

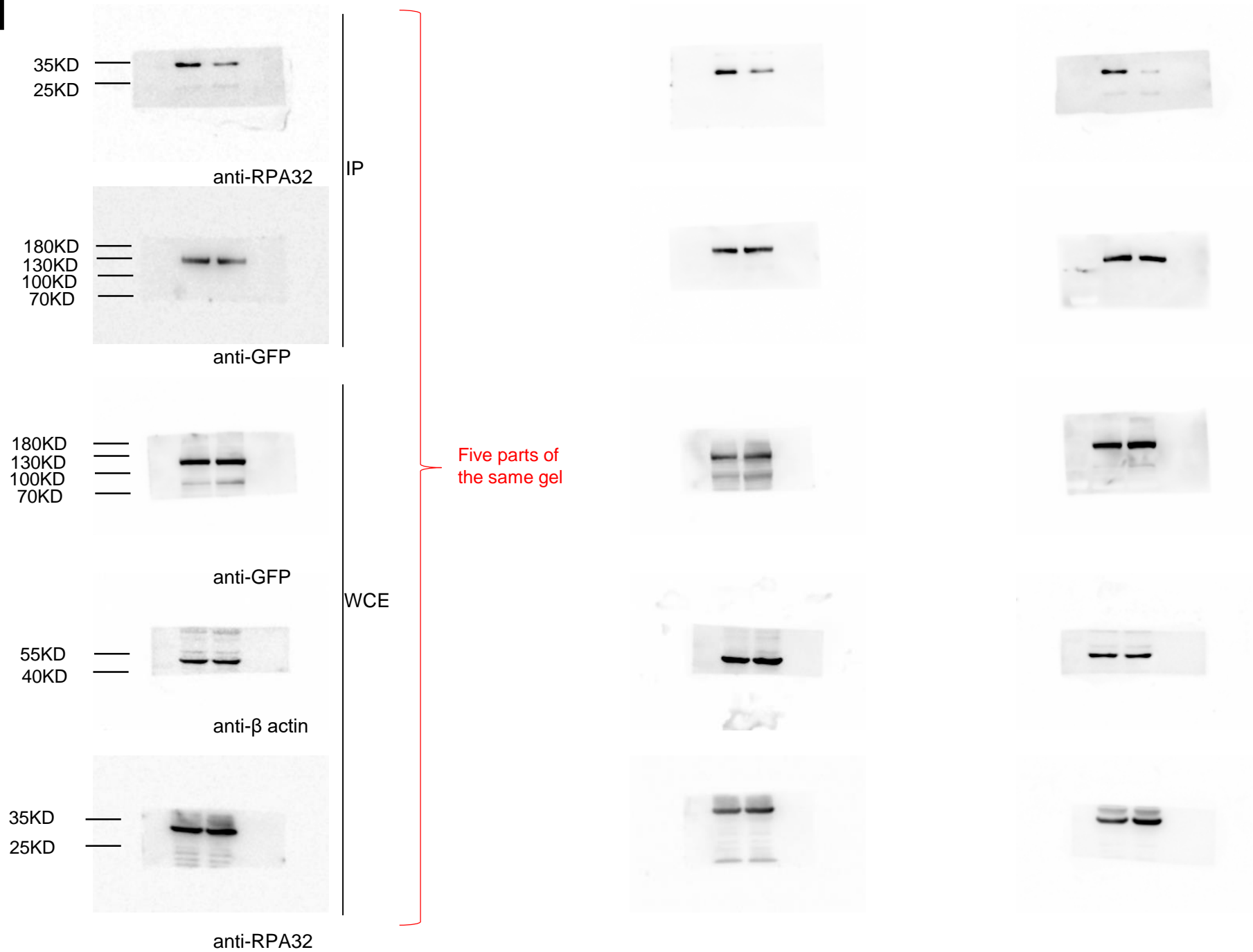

Supplement: Figure 9—source data 1. [file elife-81639-fig9-data1.zip › Figure9-source data/IP-data-Figure 9H.pdf]

Figure 9H  
ETAA1

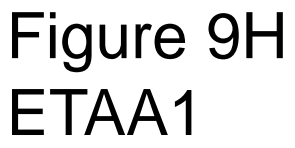

Supplement: Figure 9—source data 1. [file elife-81639-fig9-data1.zip › Figure9-source data/Figure 9H.pdf]

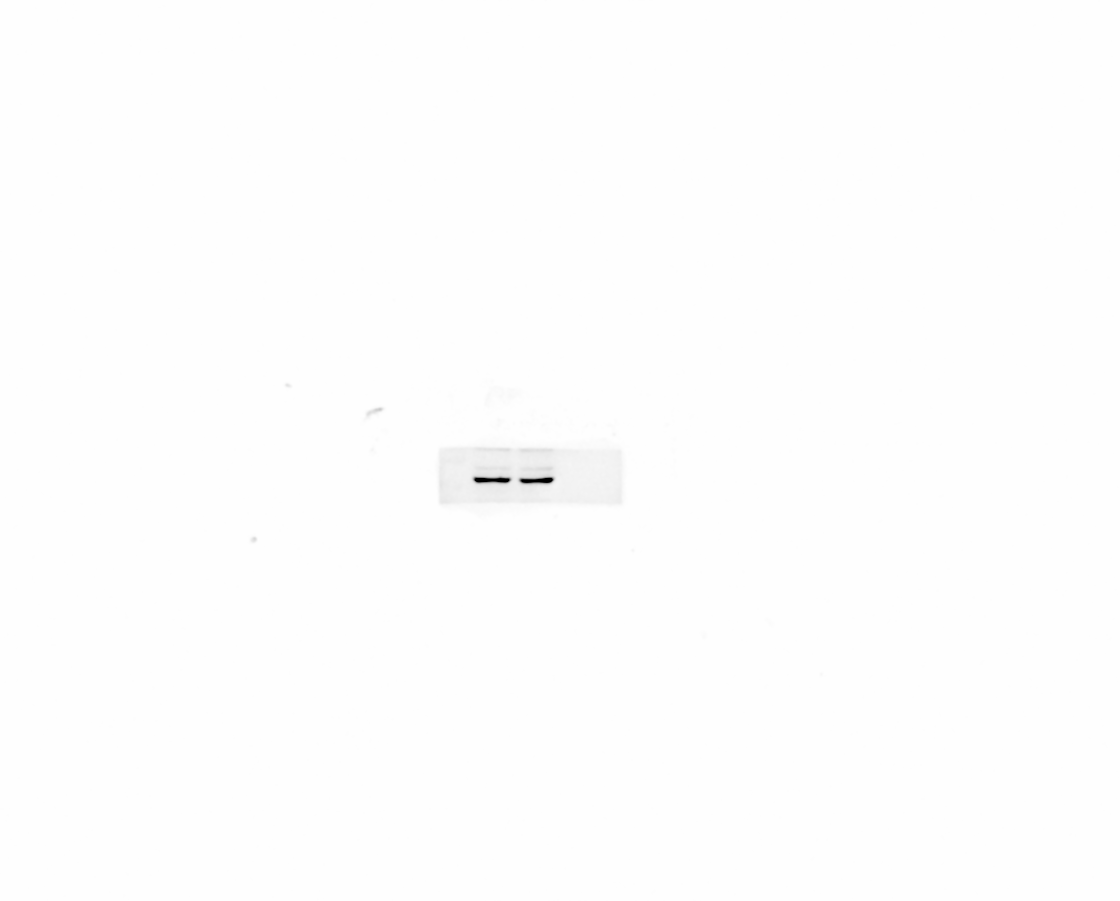

Supplement: Figure 9—source data 1. [file elife-81639-fig9-data1.zip › Figure9-source data/Figure 9H Repeat2/WCE-anti╬▓ actin.tif]

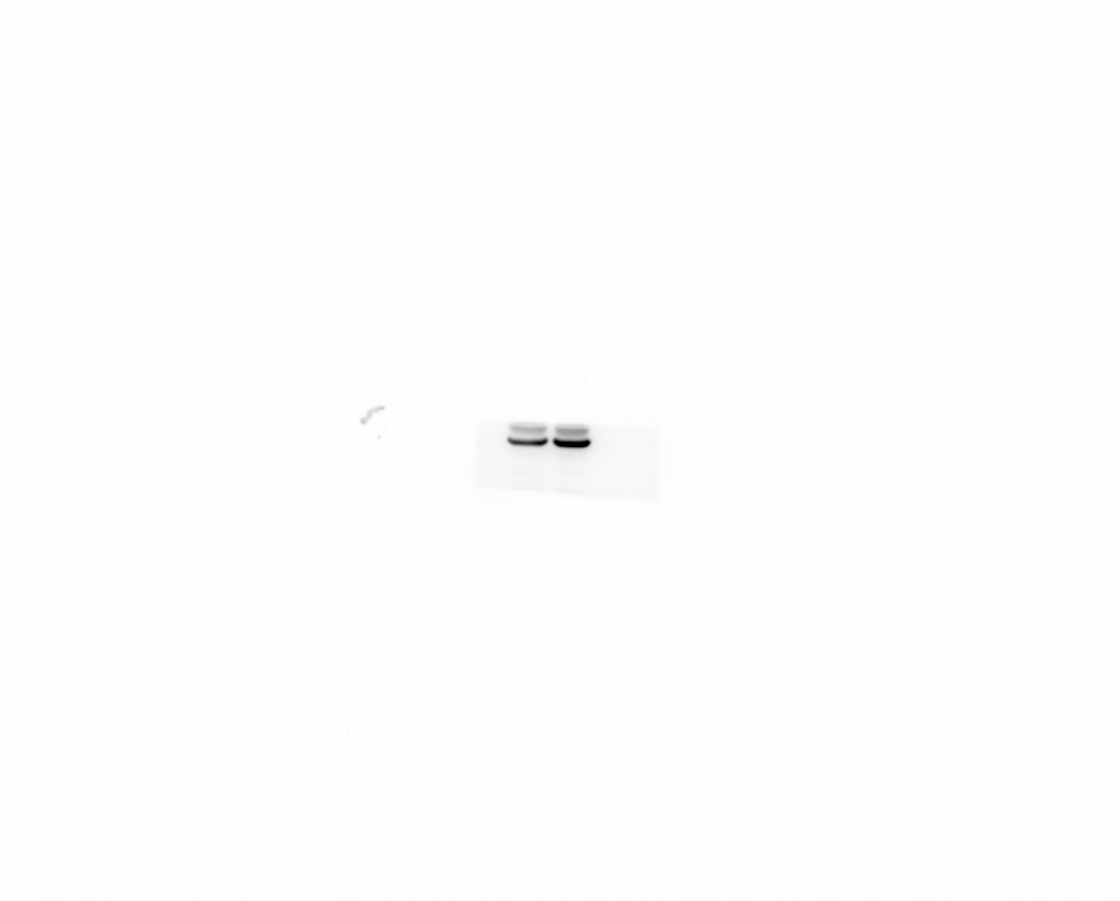

Supplement: Figure 9—source data 1. [file elife-81639-fig9-data1.zip › Figure9-source data/Figure 9H Repeat2/WCE-antiRPA32.tif]

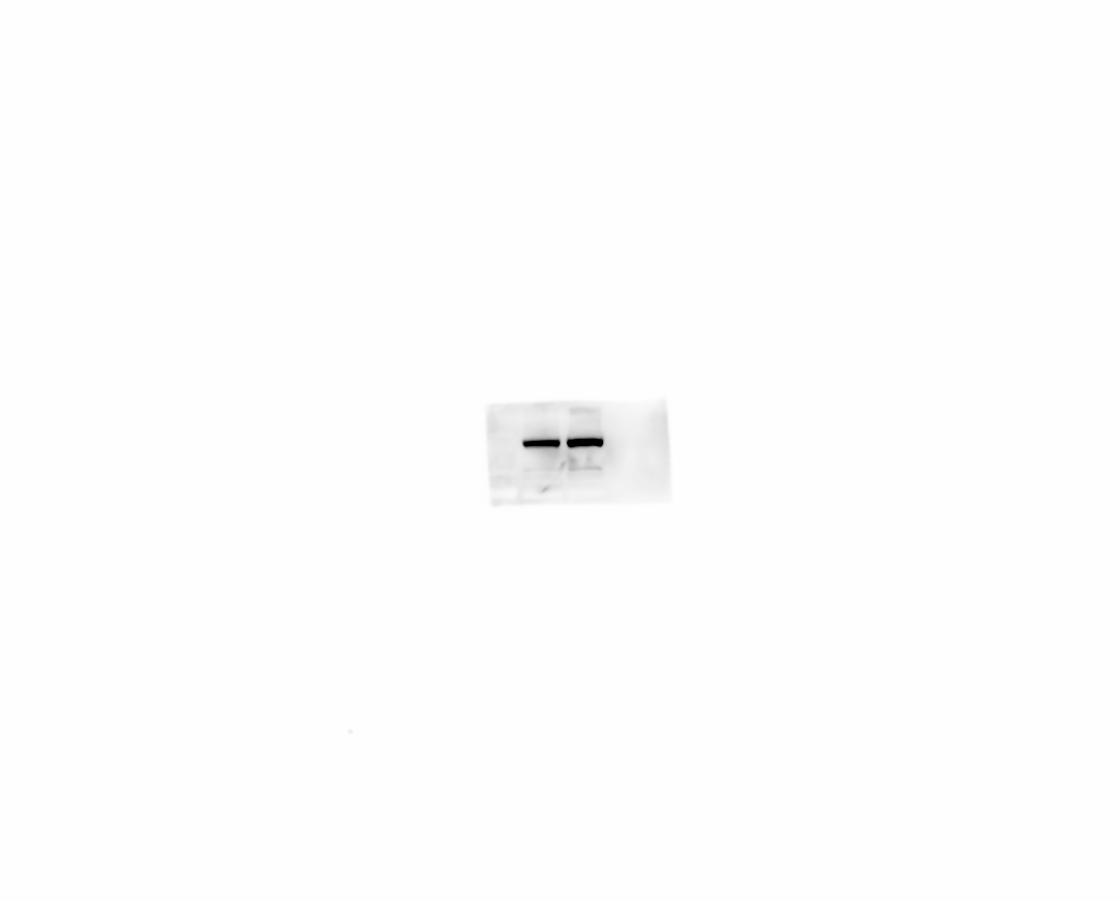

Supplement: Figure 9—source data 1. [file elife-81639-fig9-data1.zip › Figure9-source data/Figure 9H Repeat2/WCE-antiGFP.tif]

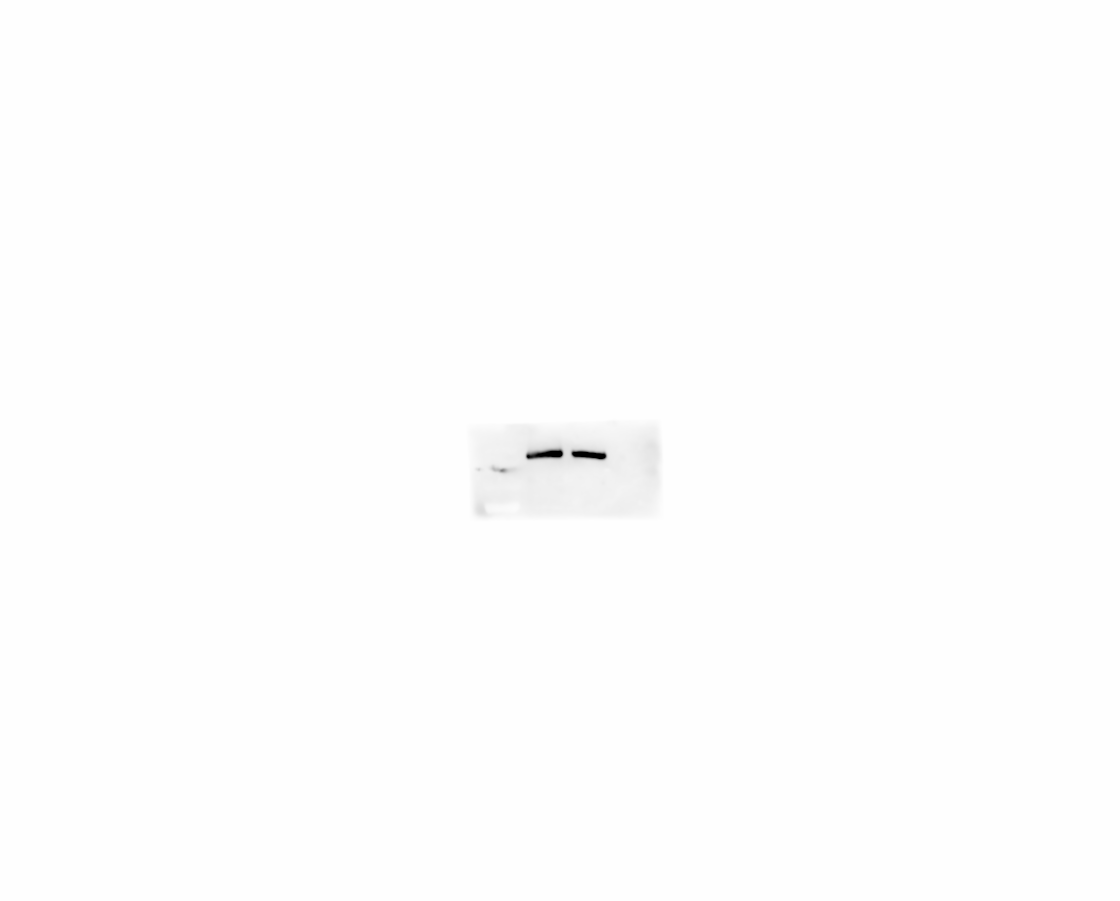

Supplement: Figure 9—source data 1. [file elife-81639-fig9-data1.zip › Figure9-source data/Figure 9H Repeat2/IP-antiGFP.tif]

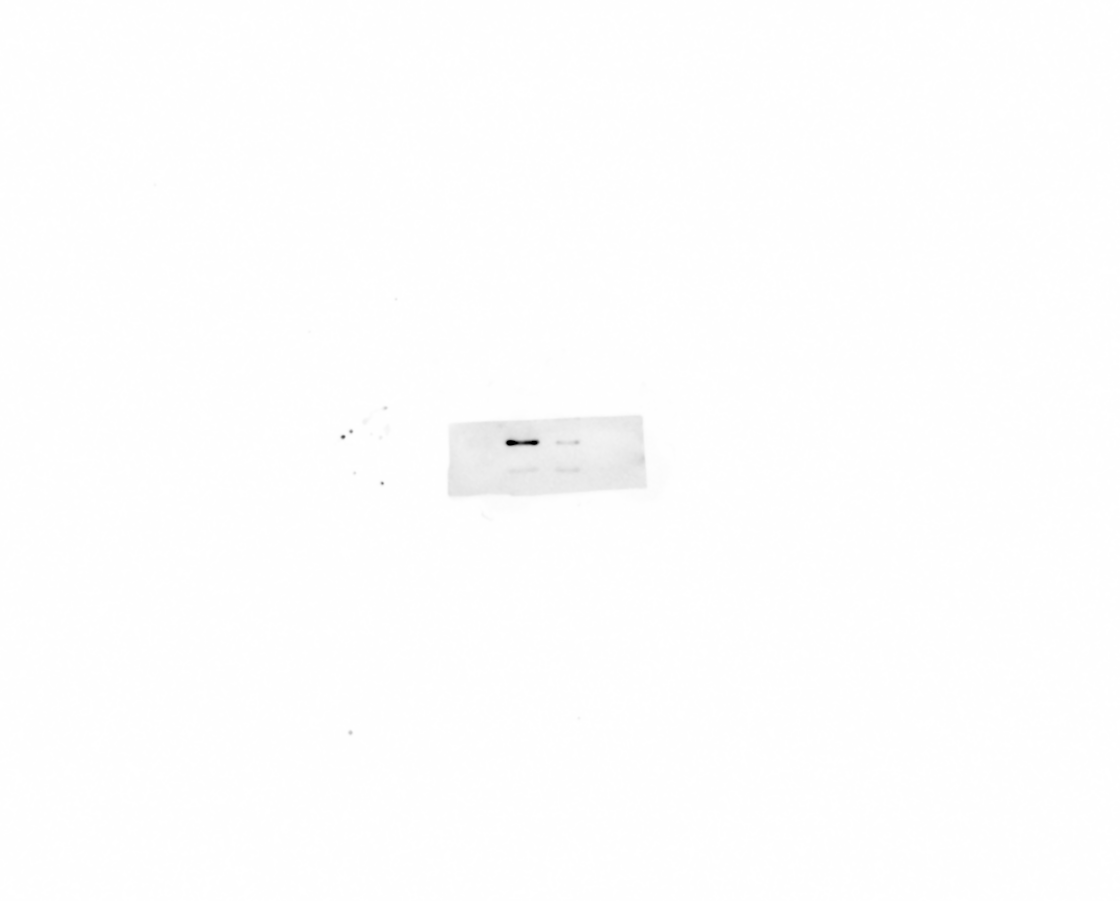

Supplement: Figure 9—source data 1. [file elife-81639-fig9-data1.zip › Figure9-source data/Figure 9H Repeat2/IP-antiRPA32.tif]

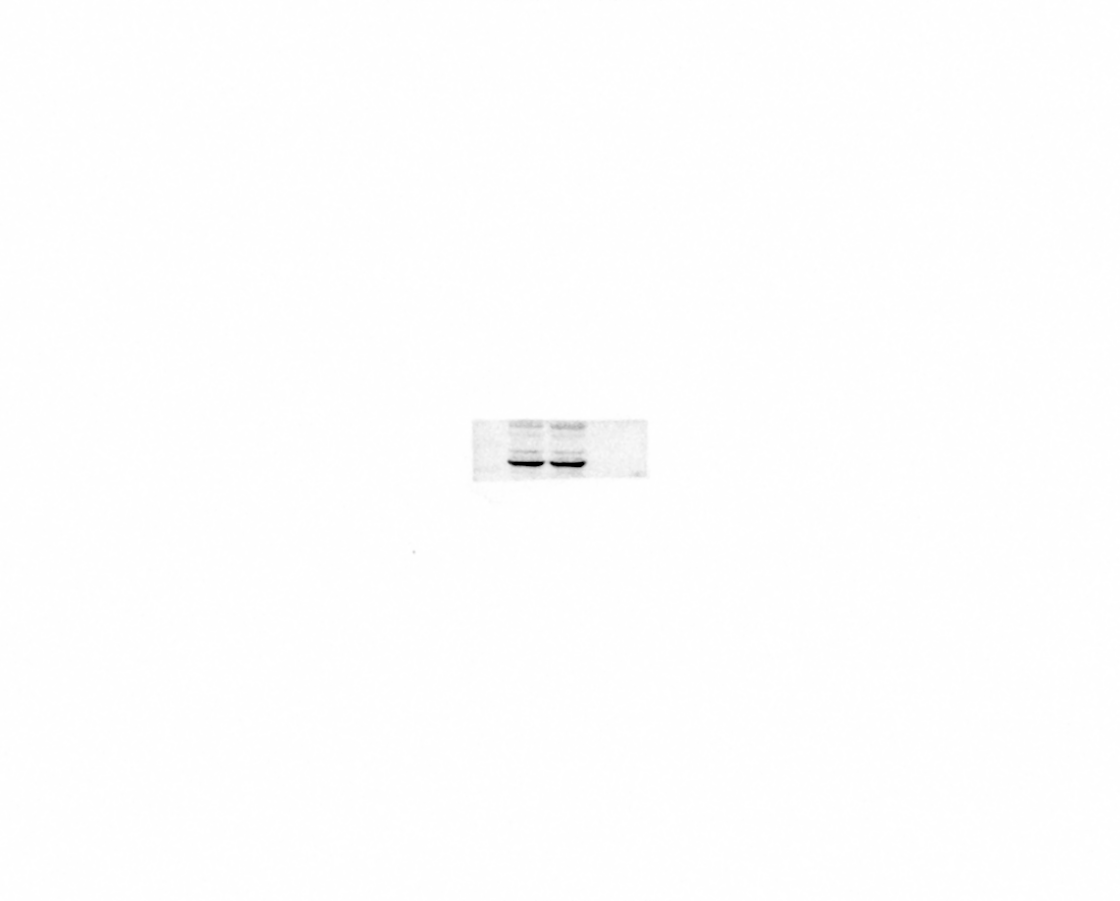

Supplement: Figure 9—source data 1. [file elife-81639-fig9-data1.zip › Figure9-source data/Figure 9H initial trial/WCE-anti╬▓ actin.tif]

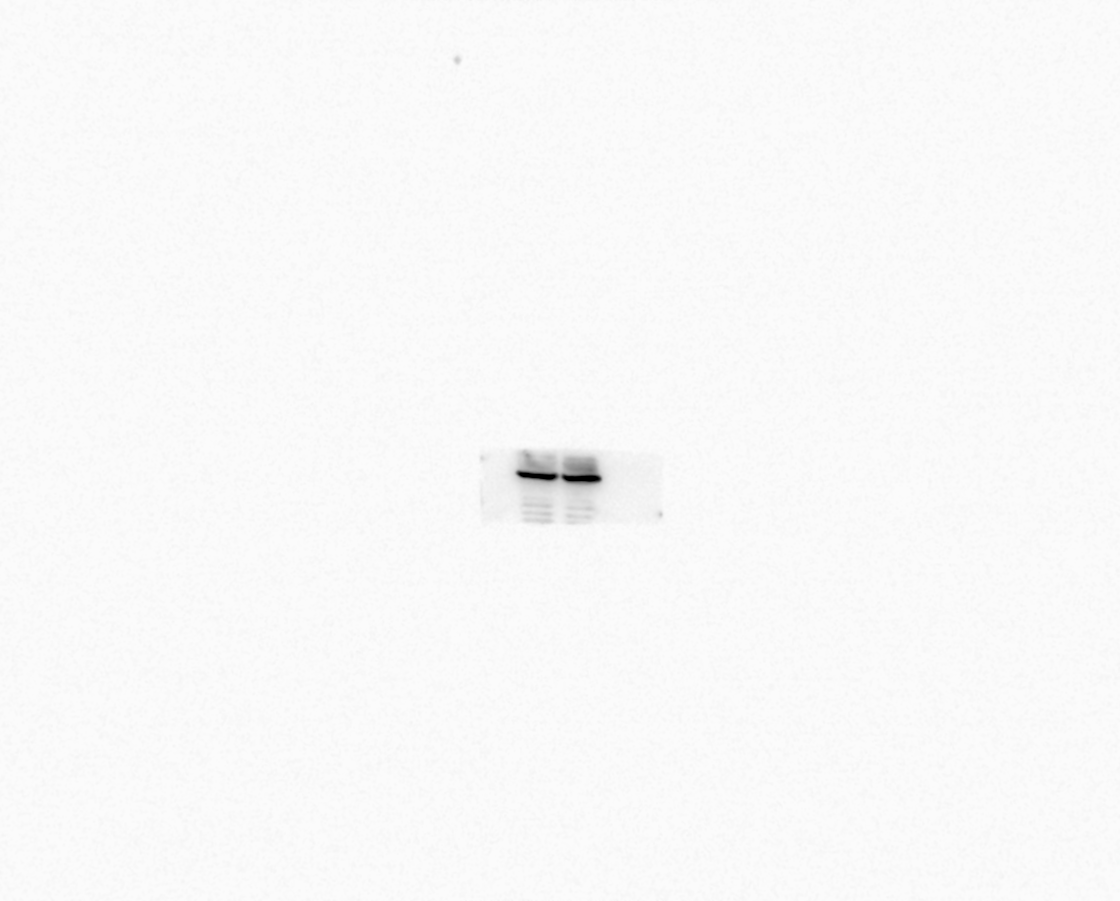

Supplement: Figure 9—source data 1. [file elife-81639-fig9-data1.zip › Figure9-source data/Figure 9H initial trial/WCE-antiRPA32.tif]

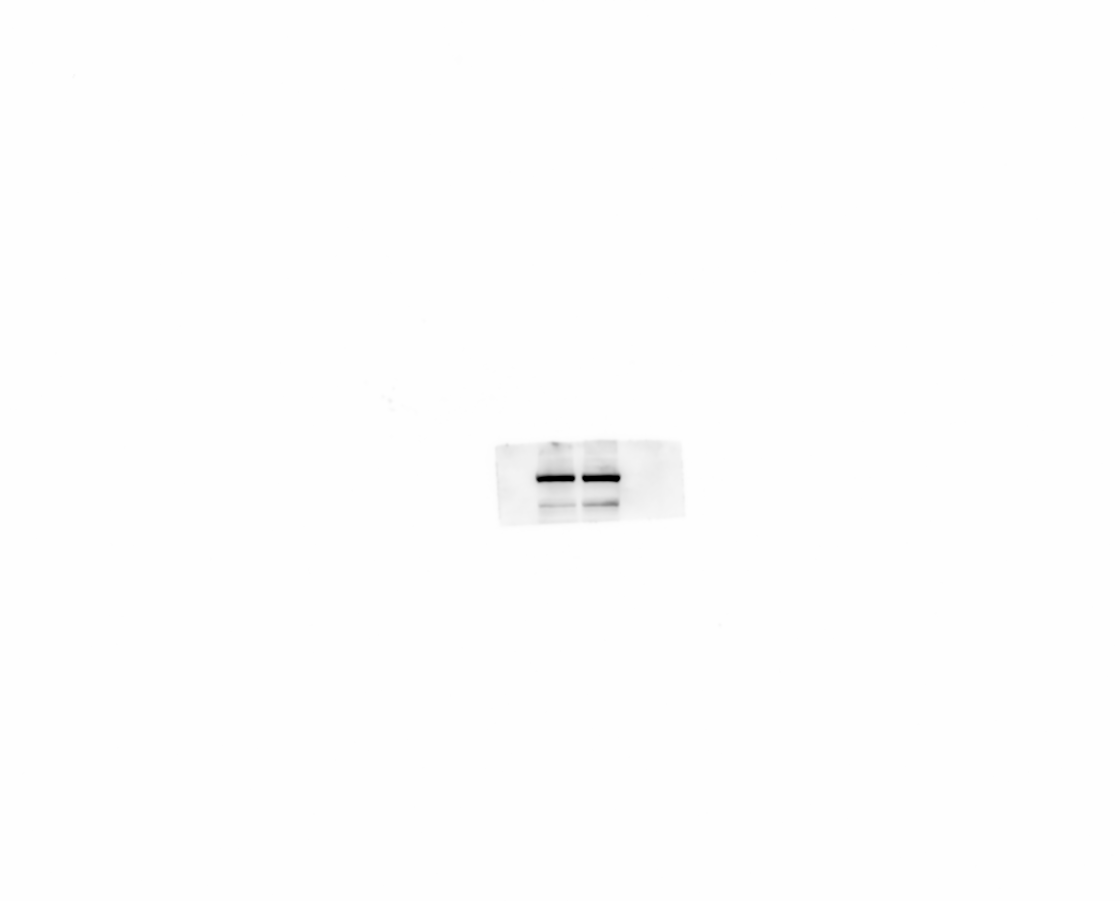

Supplement: Figure 9—source data 1. [file elife-81639-fig9-data1.zip › Figure9-source data/Figure 9H initial trial/WCE-antiGFP.tif]

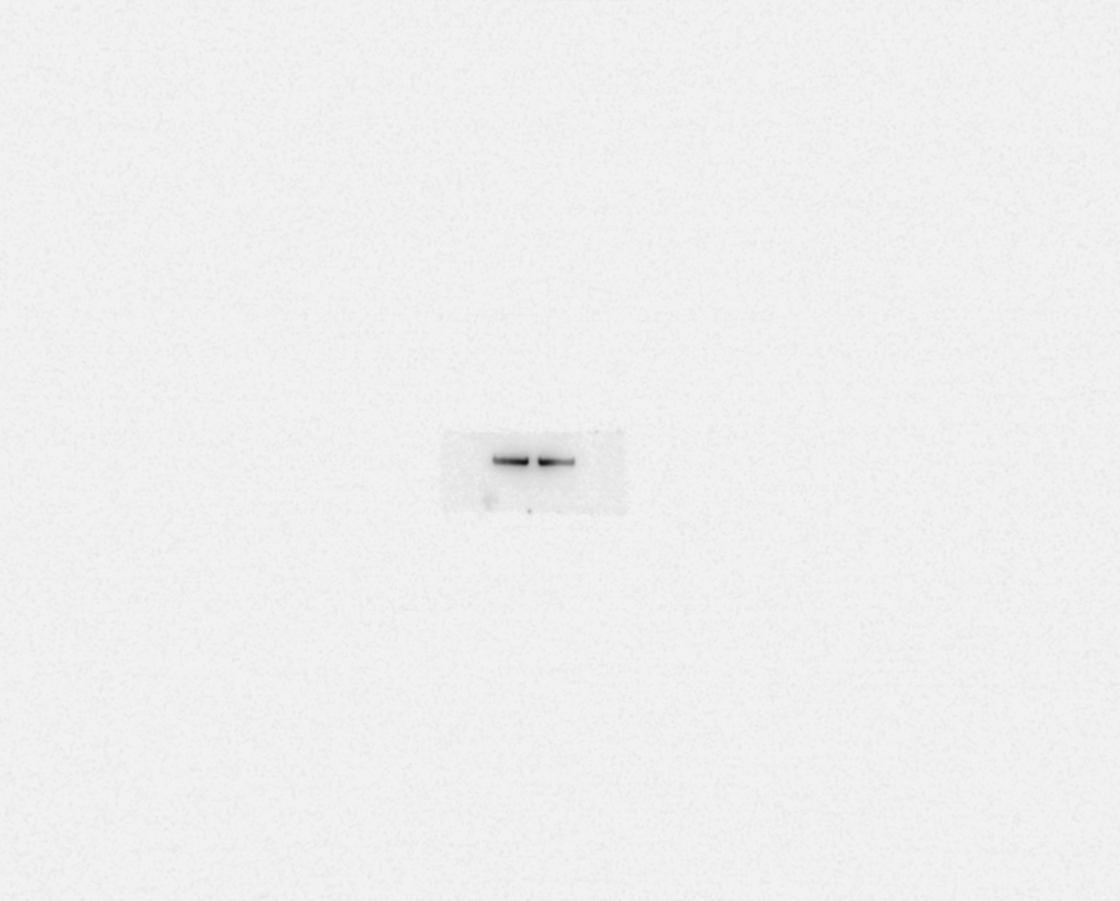

Supplement: Figure 9—source data 1. [file elife-81639-fig9-data1.zip › Figure9-source data/Figure 9H initial trial/IP-antiGFP.tif]

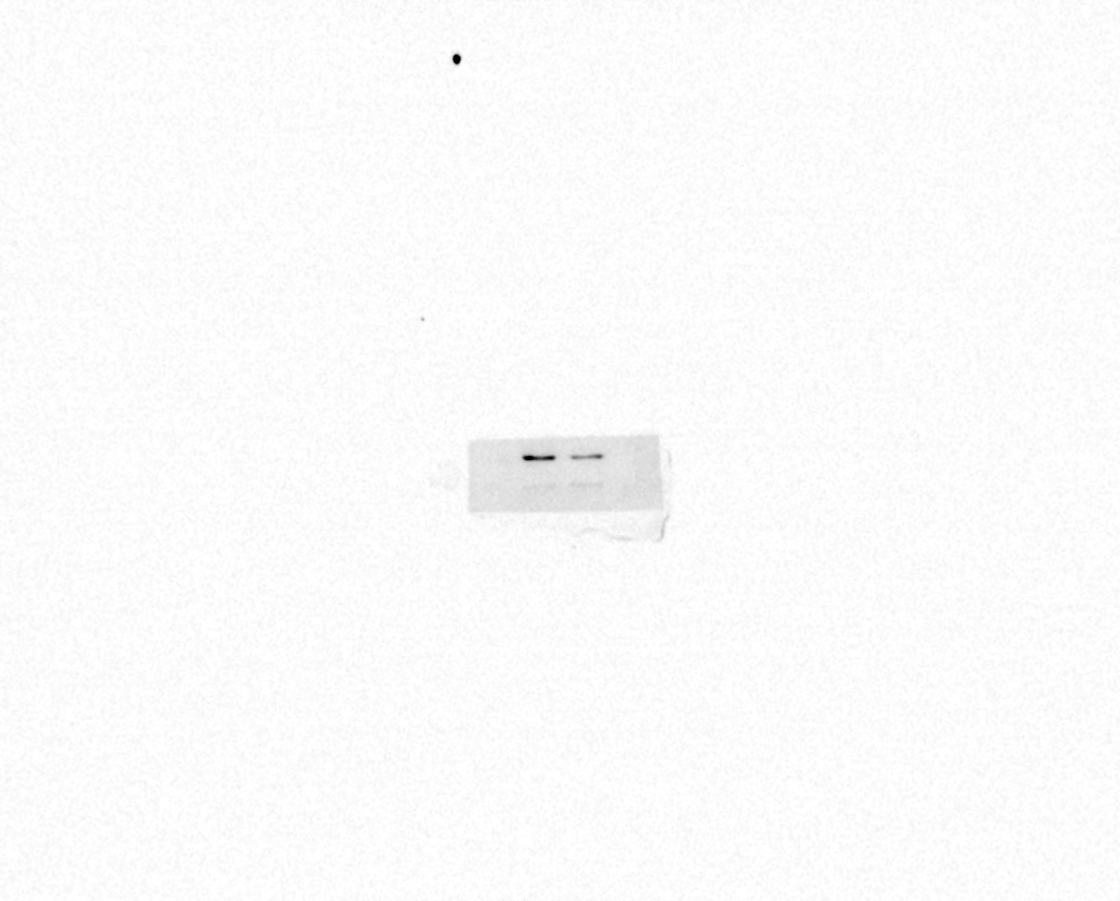

Supplement: Figure 9—source data 1. [file elife-81639-fig9-data1.zip › Figure9-source data/Figure 9H initial trial/IP-antiRPA32.tif]

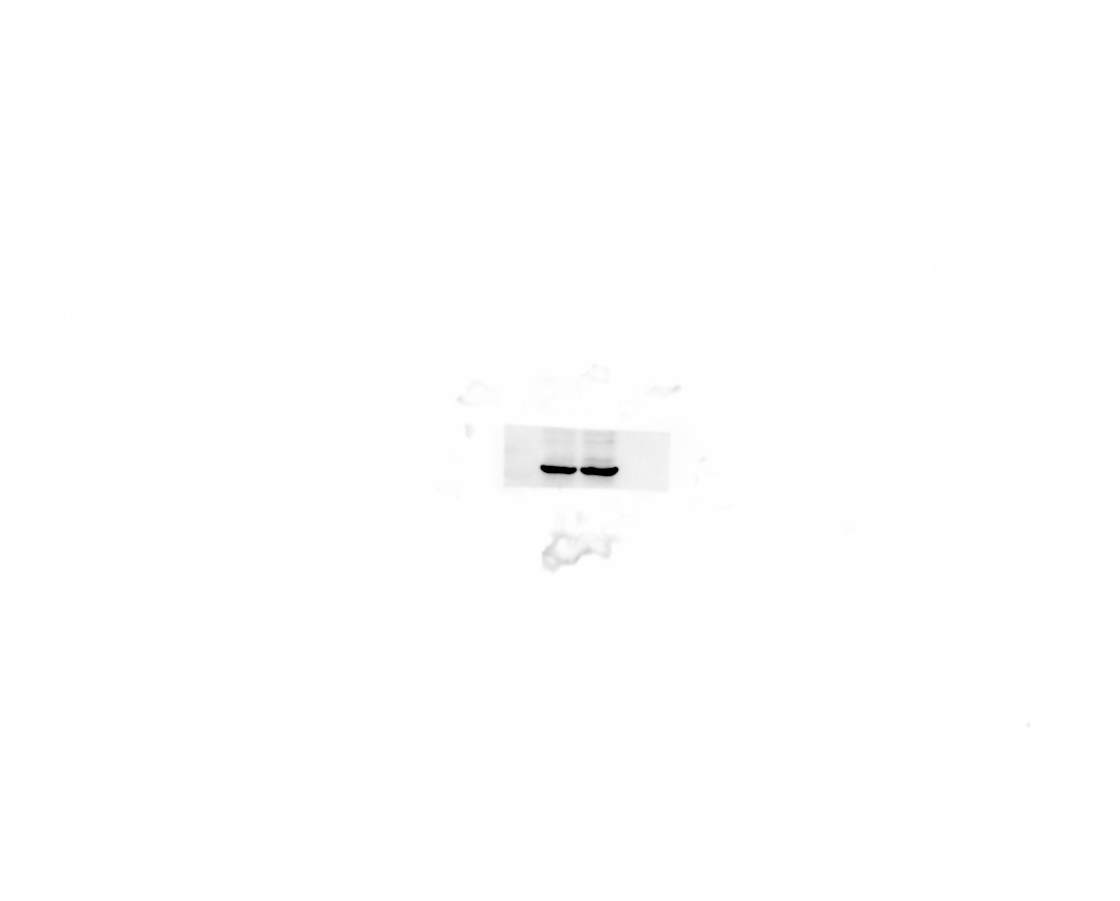

Supplement: Figure 9—source data 1. [file elife-81639-fig9-data1.zip › Figure9-source data/Figure 9H Repeat1/WCE-anti╬▓ actin.tif]

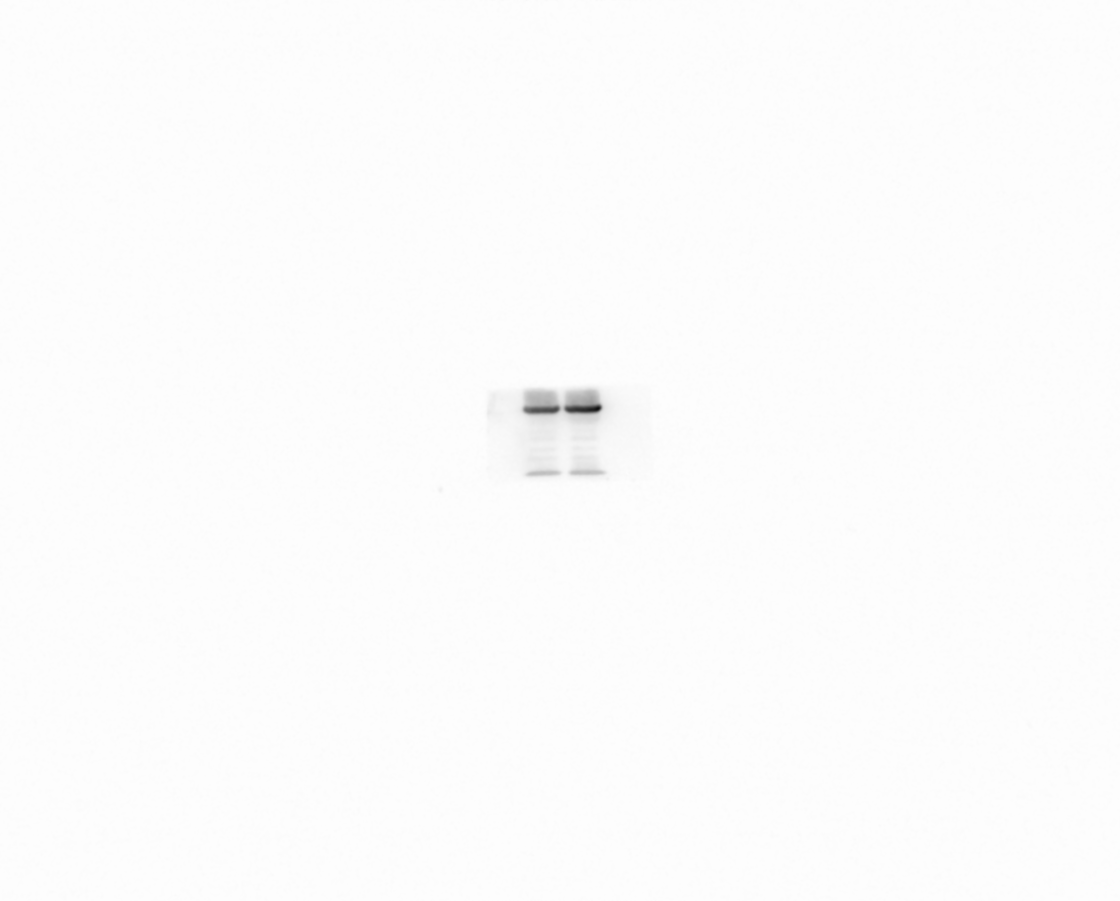

Supplement: Figure 9—source data 1. [file elife-81639-fig9-data1.zip › Figure9-source data/Figure 9H Repeat1/WCE-antiRPA32.tif]

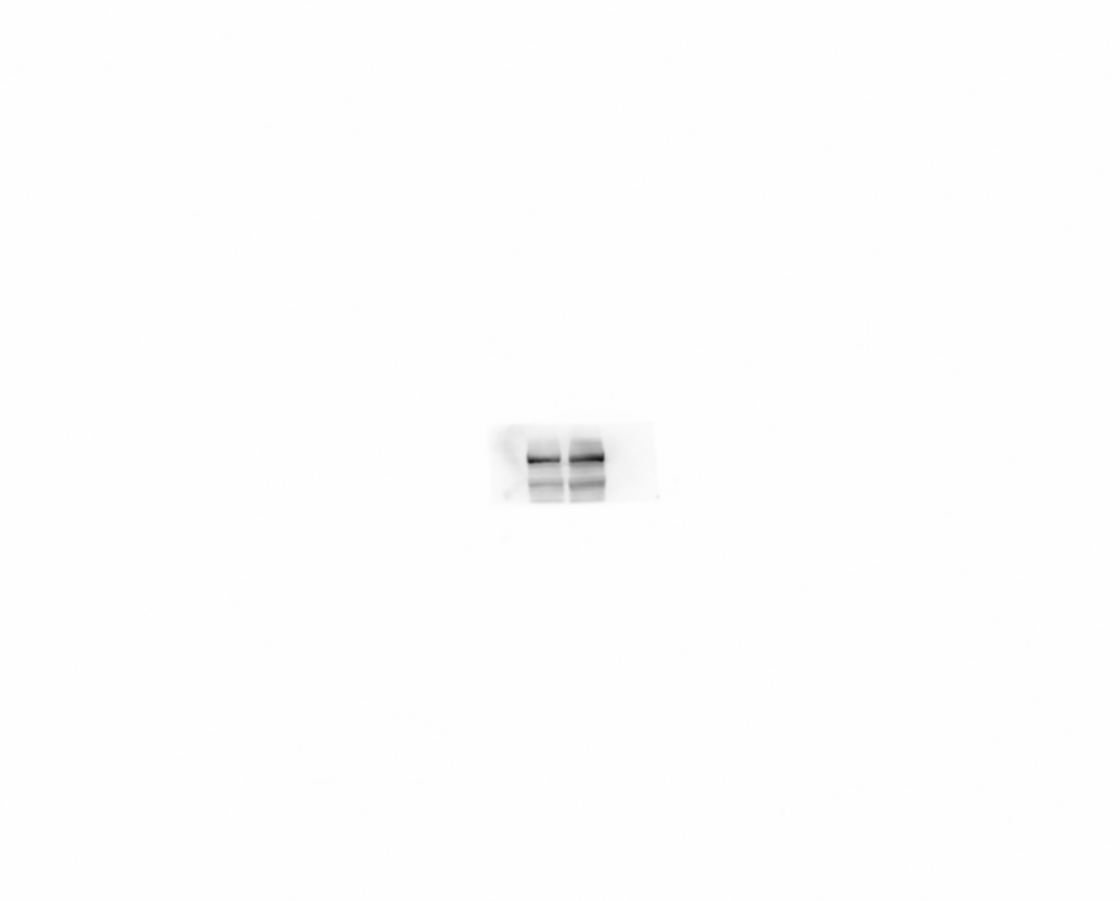

Supplement: Figure 9—source data 1. [file elife-81639-fig9-data1.zip › Figure9-source data/Figure 9H Repeat1/WCE-antiGFP.tif]

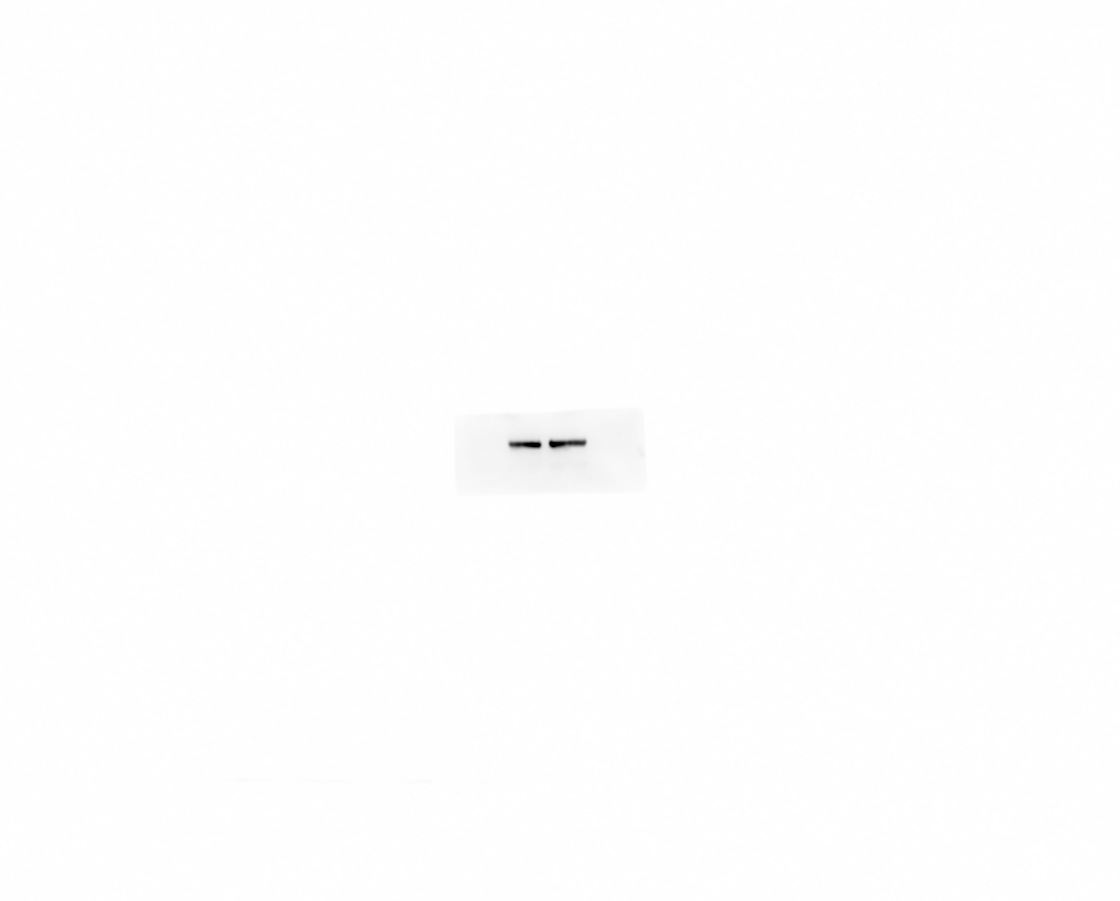

Supplement: Figure 9—source data 1. [file elife-81639-fig9-data1.zip › Figure9-source data/Figure 9H Repeat1/IP-antiGFP.tif]

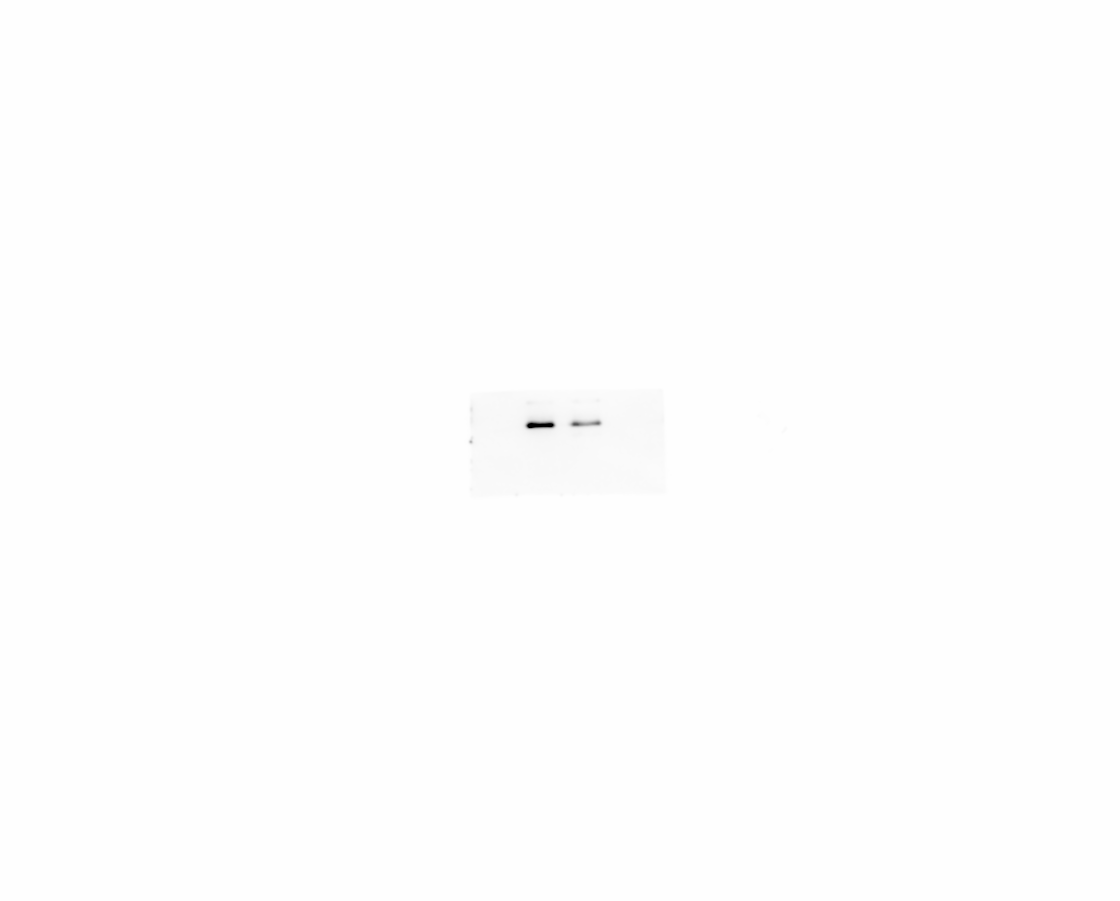

Supplement: Figure 9—source data 1. [file elife-81639-fig9-data1.zip › Figure9-source data/Figure 9H Repeat1/IP-antiRPA32.tif]
