# Supplementary material for: Structural characterization of human RPA70N association with DNA damage response proteins
Source: eLife. 2023 Sep 5;12:e81639. doi: 10.7554/eLife.81639 (PMC10479964; doi:10.7554/eLife.81639)
Supplement: Figure 10—source data 1. [file elife-81639-fig10-data1.docx]

|  | **Protein** | **Basic**  **groove** | **Side**  **pocket** | **Direction** | **Bridging two RPA70N in crystal** | **PDB code** |
| --- | --- | --- | --- | --- | --- | --- |
| **This study** | **HelB** | **Yes** | **Yes** | **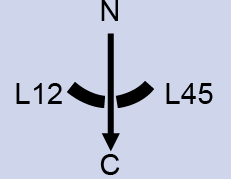** | **No** | **7XUT** |
|  | **BLMp2** | **Yes** | **No** |  | **Yes** | **7XUV** |
|  | **BLMp1** | **Yes** | **Yes** | **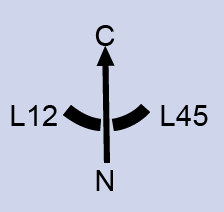** | **Yes** | **7XV0** |
|  | **RMI1** | **Yes** | **Yes** |  | **Yes** | **7XV1** |
|  | **WRN** | **Yes** | **Yes** |  | **Yes** | **7XV4** |
|  | **ATRIP** | **Yes** | **No** |  | **No** | **7XUW** |
|  | **MRE11** | **Yes** | **No** |  | **No** | **8JZY** |
|  | **RAD9** | **Yes** | **Yes** |  | **No** | **8K00** |
|  | **ETAA1** | **Yes** | **No** |  | **No** | **8JZV** |
| **Previous studies** | **p53** | **Yes** | **Yes** | **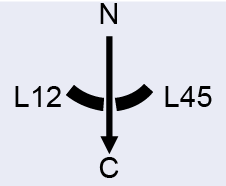** | **Yes** | **2B3G** |
|  | **DNA2** | **Yes** | **Yes** |  | **No** | **5EAY** |
|  | **PrimPol** | **Yes** | **No** | **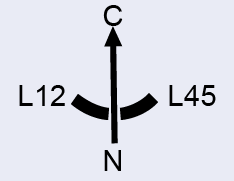** | **No** | **5N85** |
|  | **Ddc2** | **Yes** | **No** |  | **No** | **5OMB** |
